# Photoredox-catalyzed arylation of isonitriles by diaryliodonium salts towards benzamides

**DOI:** 10.3762/bjoc.21.110

**Published:** 2025-07-21

**Authors:** Nadezhda M Metalnikova, Nikita S Antonkin, Tuan K Nguyen, Natalia S Soldatova, Alexander V Nyuchev, Mikhail A Kinzhalov, Pavel S Postnikov

**Affiliations:** 1 Research School of Chemistry and Applied Biomedical Sciences, Tomsk Polytechnic University, Lenina Avenue 30, Tomsk, 634050, Russian Federation; 2 Saint Petersburg State University, 7–9 Universitetskaya Nab., Saint Petersburg, 199034, Russian Federation; 3 Department of Organic Chemistry, Lobachevsky State University of Nizhny Novgorod, Gagarina Avenue 23, Nizhny Novgorod, 603950, Russian Federation

**Keywords:** arylation, benzamides, diaryliodonium salts, isonitriles, photoredox

## Abstract

The arylation of isonitriles by diaryliodonium salts under photoredox conditions has been proposed for the first time. The suggested procedure allows preparing a broad range of benzamides using both symmetric and unsymmetric diaryliodonium salts under mild conditions. A plausible mechanism for the reaction and the selectivity of aryl transfer (in case of unsymmetrical iodonium salts) were studied.

## Introduction

**Scheme 1:**
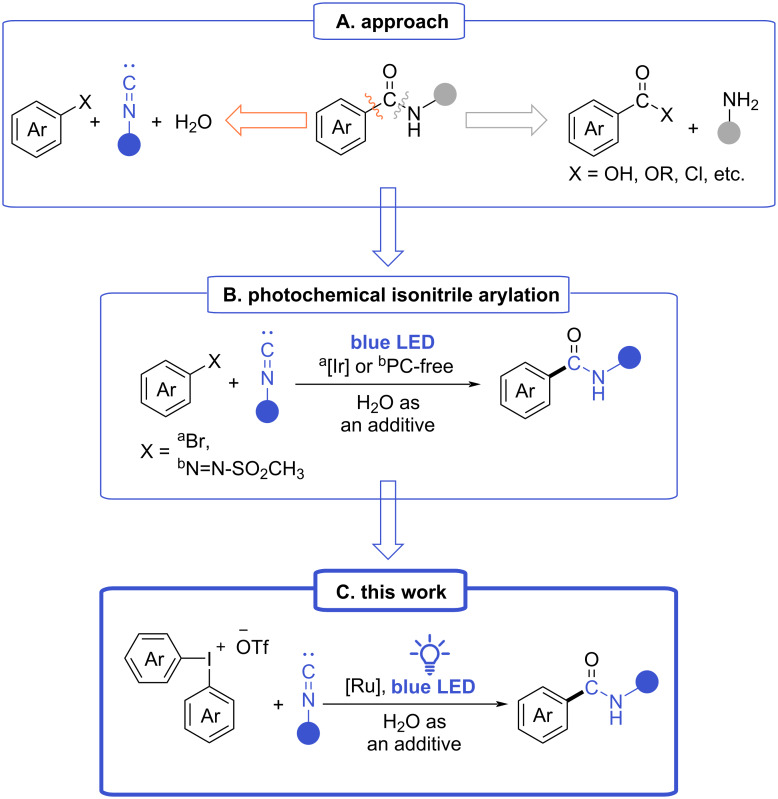
Background and conception.

Amides represent a crucial and ubiquitous structural motif in essential biomolecules including proteins and peptides [1], as well as in a wide array of bioactive compounds. According to the DrugBank there are more than 250 approved drugs classified as amides [2]. Just recently, between February 2021 and June 2022, sixteen anticancer drugs containing an amide bond have been approved by the U.S. FDA [3]. Consequently, the preparation of amides has garnered significant attention within organic and medicinal chemistry. Commonly, amide bonds are formed via the reaction of carboxylic acids or their derivatives with appropriate amines (Scheme 1A) [4]. Although this conventional approach is effective and straightforward, it usually suffers from harsh conditions and low tolerance to sensitive functional groups. Due to these reasons, alternative routes toward the preparation of amides are still in great demand in modern synthetic chemistry [5].

The arylation of isonitriles was introduced nearly a decade ago as an alternative synthetic pathway for the preparation of benzamides (Scheme 1A) [6]. Over the years, the basic reaction has been modified to imply various metal-containing catalysts [7–18], metal-free transformations that employ heteroarenes under harsh conditions [19], or using diazonium salts as arylating agents [20–21]. However, diazonium salts are distinguished by their inherent instability complicating their use in such transformations. Moreover, among the various arylation strategies, photochemical methods remain relatively underexplored, with only a few examples reported [22–23]. These methods often face limitations such as the significant excess of isonitrile [22] or a restricted scope of aryl bromides (Scheme 1B) [23].

In contrast, diaryliodonium salts, representing stable, robust, and efficient arylating agents [24–30], have not been explored yet for the synthesis of benzamides from isonitriles as well as in multicomponent reactions with isonitriles in general. Only a few examples of photochemical cascade arylation–cyclizations of isonitriles with diaryliodonium salts have been published [31–33]. To bridge this gap, we propose a photoredox-mediated strategy for the synthesis of benzamides via the arylation of isonitriles with diaryliodonium salts under blue light irradiation (Scheme 1C).

## Results and Discussion

We commenced our investigation by the optimization of the reaction conditions. During the preliminary experiments we tested different solvents and solvent-to-water ratios to establish initial conditions (Supporting Information File 1, 2.1 Solvent screening). Diphenyliodonium triflate (**1a**) and 1-isocyano-4-methylbenzene were used as model substrates. Taking into account the possibility of iodonium salt decomposition under irradiation we carried out an initial experiment without a catalyst and observed only traces of the benzamide **2aa** (Table 1, entry 1). However, we detected almost half of the salt **1a** remained in the reaction medium after 10 hours of reaction (Supporting Information File 1, Figure S3). Thus, we settled with the similar conditions to the published ones [31–33] introducing the photocatalyst [Ru(bpy)_3_](PF_6_)_2_, which successfully initiated the reaction under blue light irradiation and afforded benzamide **2aa** in 36% yield (Table 1, entry 2). In that case less than 2% of the iodonium salt **1a** remained in the reaction medium according to the ^1^H NMR spectrum of the crude mixture (Supporting Information File 1, Figure S4). Despite its higher reductive potential, *fac*-Ir(ppy)_3_ gave a lower yield of 26% for **2aa** compared to [Ru(bpy)_3_](PF_6_)_2_ (Table 1, entry 3). Inspired by the first positive results, we tested various cyanoarene-based catalytic systems. Unfortunately, 4CzTPN, 4CzIPN, and 3DPAFIPN did not demonstrate increased efficiency, and the yields of the target product **2aa** were slightly lower than for [Ru(bpy)_3_](PF_6_)_2_ (Table 1, entries 4–6). Thus, all further optimization studies were done using [Ru(bpy)_3_](PF_6_)_2_ as a catalyst.

**Table 1:** Optimization of the reaction conditions.^a^

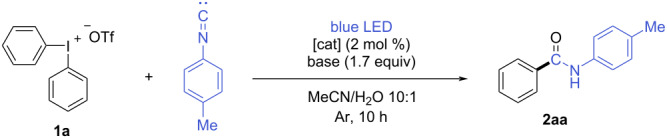					

#	Isonitrile/iodonium salt ratio	*c* (isonitrile) [M]	Catalyst^b^	Base	Yield [%]^c^

variation of catalysts					

1	1:1	0.1	-	Na_2_CO_3_	n.d.
**2**	**1:1**	**0.1**	**[Ru(bpy)** **_3_** **](PF** **_6_** **)** **_2_**	**Na** **_2_** **CO** **_3_**	**36 (31)** **^d^**
3	1:1	0.1	*fac*-Ir(ppy)_3_	Na_2_CO_3_	26
4	1:1	0.1	4CzTPN	Na_2_CO_3_	20
5	1:1	0.1	4CzIPN	Na_2_CO_3_	30
6	1:1	0.1	3DPAFIPN	Na_2_CO_3_	30

variation of bases					

7	1:1	0.1	[Ru(bpy)_3_](PF_6_)_2_	Na_2_HPO_4_	21
8	1:1	0.1	[Ru(bpy)_3_](PF_6_)_2_	NaH_2_PO_4_	24
9	1:1	0.1	[Ru(bpy)_3_](PF_6_)_2_	-	22
10	1:1	0.1	[Ru(bpy)_3_](PF_6_)_2_	Cs_2_CO_3_	30
11	1:1	0.1	[Ru(bpy)_3_](PF_6_)_2_	KOH	11

variation of reagents concentration					

12	1:1	0.2	[Ru(bpy)_3_](PF_6_)_2_	Na_2_CO_3_	32
13	1:1	0.05	[Ru(bpy)_3_](PF_6_)_2_	Na_2_CO_3_	28

variation of isonitrile/iodonium salt ratio					

14	1:2	0.1	[Ru(bpy)_3_](PF_6_)_2_	Na_2_CO_3_	31
15	1:3	0.1	[Ru(bpy)_3_](PF_6_)_2_	Na_2_CO_3_	20
16	1.5:1	0.08	[Ru(bpy)_3_](PF_6_)_2_	Na_2_CO_3_	28
17	2:1	0.1	[Ru(bpy)_3_](PF_6_)_2_	Na_2_CO_3_	34
18	2:1	0.2	[Ru(bpy)_3_](PF_6_)_2_	Na_2_CO_3_	42
19	3:1	0.15	[Ru(bpy)_3_](PF_6_)_2_	Na_2_CO_3_	34
20	4:1	0.2	[Ru(bpy)_3_](PF_6_)_2_	Na_2_CO_3_	36
21	4:1	0.4	[Ru(bpy)_3_](PF_6_)_2_	Na_2_CO_3_	30

variation of iodonium salts					

22^e^	1:1	0.1	[Ru(bpy)_3_](PF_6_)_2_	Na_2_CO_3_	28
23^f^	1:1	0.1	[Ru(bpy)_3_](PF_6_)_2_	Na_2_CO_3_	37

control experiments					

24^g^	1:1	0.1	[Ru(bpy)_3_](PF_6_)_2_	Na_2_CO_3_	traces
25^h^	1:1	0.1	[Ru(bpy)_3_](PF_6_)_2_	Na_2_CO_3_	9

^a^Reaction conditions: MeCN (1 mL), H_2_O (100 μL), irradiation by blue LED (465 nm, 20 W) for 10 h. ^b^The structures of photocatalysts are given in Supporting Information File 1, Figure S2. ^c^Determined by ^1^H NMR spectroscopy using 1,3,5-isopropylbenzene as an internal standard. ^d^Isolated yield (for 0.2 mmol scale). ^e^**1a-BF_4_** was used instead of **1a**. ^f^**1a-TsO** was used instead of **1a**. ^g^In the dark. ^h^Under air.

After, we moved to the screening of bases and their potential role in the arylation process. Firstly, the reaction without a base, or using weaker bases such as sodium phosphates, resulted in reduced yields of product **2aa** (Table 1, entries 7–9) due to the acidic hydrolysis of the isonitrile to formamide [34]. Stronger bases such as Cs_2_CO_3_ (Table 1, entry 10) and KOH (Table 1, entry 11) led to diminished yields, reducing the product formation to 30% and 11%, respectively. In both cases we observed substantial decomposition of the iodonium salt affecting the yield of the desired product. We also performed the reaction with DIPEA as an organic base but the yield of **2aa** was very low and we observed a range of byproducts, mainly amine addition instead of water (see Supporting Information File 1, 2.2 Preliminary and Additional Experiments, Figures S5 and S6).

Afterwards, we evaluated the other crucial parameters for photochemical reactions such as the reagents concentrations and ratio. Surprisingly, both dilution of the reaction mixture to 0.05 M and concentration to 0.2 M led to reduced yields (Table 1, entries 12 and 13) compared to the optimal concentration of 0.1 M. Furthermore, the yield of **2aa** exhibited minimal dependence on the molar ratio of iodonium salt to isonitrile (Table 1, entries 14–21). In case of excess of the isonitrile we observed multiple addition and formation of oligomerized products which hindered the isolation of the product **2aa** (Supporting Information File 1, 2.2 Preliminary and Additional Experiments, supplementary note 1).

It is known that the counteranion of an iodonium salt could be a crucial factor for the reactivity pattern. Thus, we tested iodonium salts with different anions. Reactions with diphenyliodonium tetrafluoroborate **1a-BF_4_** (Table 1, entry 22) and tosylate **1a-TsO** (Table 1, entry 23) gave benzamide **2aa** in 28% and 37% yield, respectively. Diphenyliodonium tosylate **1a-TsO** afforded the product **2aa** in a yield which was comparable to triflate **1a**. However, we proceeded for further experiments with the triflate **1a** since the triflates are generally more synthetically available.

Finally, control experiments without irradiation gave only traces of the benzamide **2aa** showing no activation by the Ru complex at room temperature (Table 1, entry 24). An experiment conducted under an air atmosphere yielded only 9% of **2aa** (Table 1, entry 25) indicating that the presence of atmospheric oxygen significantly inhibited the reaction.

Based on these findings and some additional experiments (see Supporting Information File 1, Table S1), the optimal conditions for further studies were established as the use of equimolar amounts of diaryliodonium salt and isonitrile, Na_2_CO_3_ as the base, and [Ru(bpy)_3_](PF_6_)_2_ as the photocatalyst, under an Ar atmosphere with irradiation by blue LED light (Table 1, entry 2).

**Scheme 2:**
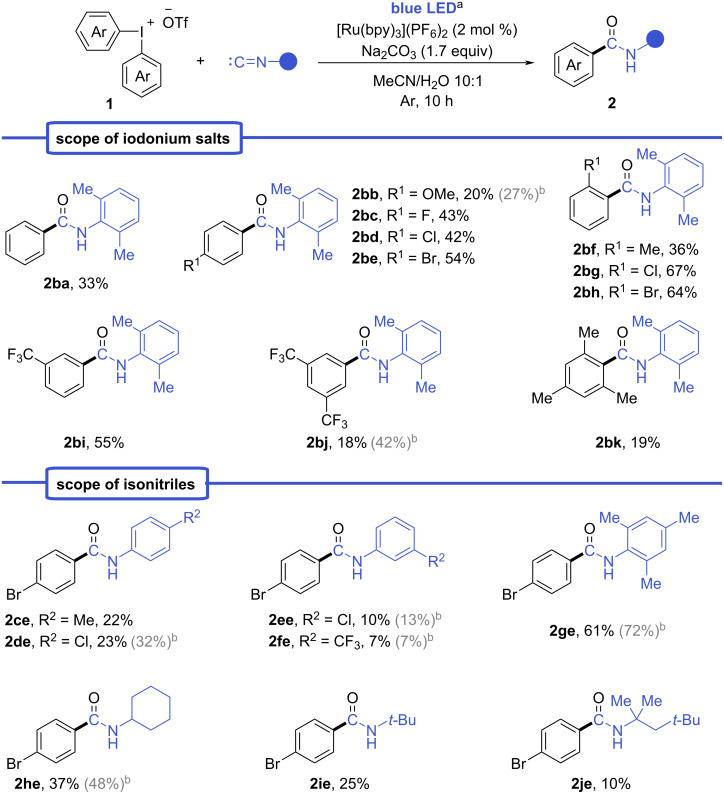
Reaction scope of iodonium salts **1** and isonitriles. ^a^Reaction conditions: isonitrile (0.2 mmol), iodonium salt **1** (0.2 mmol), Na_2_CO_3_ (0.34 mmol), [Ru(bpy)_3_](PF_6_)_2_ (0.004 mmol), MeCN (2 mL), H_2_O (200 μL), irradiation by blue LED (465 nm, 20 W) for 10 h. ^b^Yields in grey correspond to reactions with 2 equiv of iodonium salt **1** and 3.4 equiv of Na_2_CO_3_.

With the optimized conditions in hands, a series of benzamides **2aa**–**je** were synthesized using various symmetrical diaryliodonium salts **1a**–**k** and isonitriles (Scheme 2). The analysis of reaction yields allowed us to establish the dependency from the electronic effects of substituents in the diaryliodonium salts. Diaryliodonium salts containing electron-deficient aryls afforded products **2** in higher yields compared to those bearing electron-donating groups (EDG). Specifically, the reaction with electron-withdrawing groups (EWG)-substituted iodonium salts produced benzamides **2bc**–**be**, **2bg**–**bj** in 18–67% yields. The benzamide **2bj** was isolated in only 18% yield probably due to the low solubility of bis(3,5-ditrifluoromethyl)iodonium triflate (**1j**) in MeCN/H_2_O mixture. The highest yields were achieved in case of *o*-halo-substituted diaryliodonium salts providing 64% and 67% for amides **2bg** and **2bh**, respectively. In contrast, iodonium salts with electron-rich aryls afforded the corresponding benzamides **2aa**, **2ba**, **2bb**, **2bf**, and **2bk** in significantly lower 19–36% yields.

Subsequently, various isonitriles were evaluated in the reaction with bis(4-bromophenyl)iodonium triflate (**1e**) under optimized conditions**.** The corresponding amides were successfully synthesized from both aliphatic and aromatic isonitriles, with yields reaching up to 61%. The highest yields were observed for benzamides **2be** and **2ge** in the reaction with sterically hindered aromatic isonitriles, such as 1-isocyano-2,4,6-trimethylbenzene and 1-isocyano-2,6-dimethylbenzene.

The use of 2 equivalents of iodonium salt **1** further improved the yields of certain amides (**2bb**, **2bj**, **2de**, **2ee**, **2ge** and **2he**), as significant amounts of unreacted isonitrile remained when only 1 equivalent was employed.

**Scheme 3:**
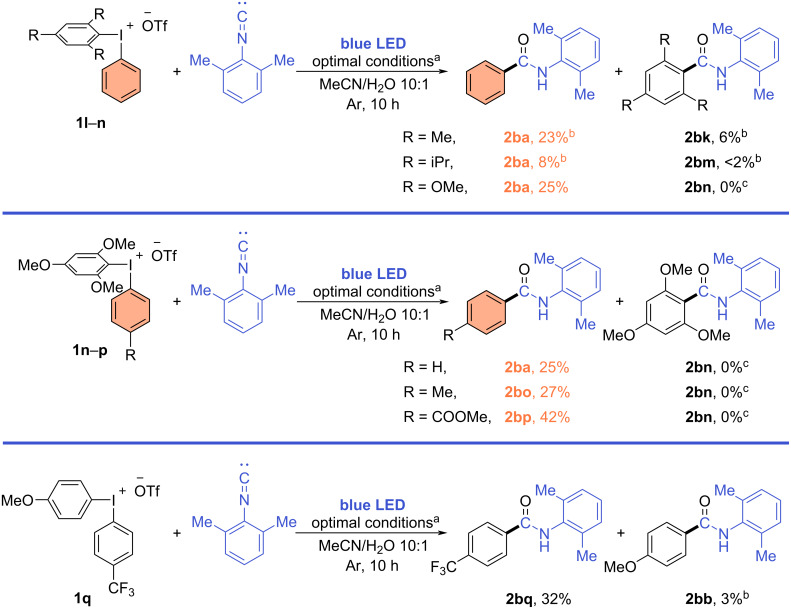
Selectivity experiments and scope of unsymmetrical iodoniums salts. ^a^Reaction conditions: 2-isocyano-1,3-dimethylbenzene (0.2 mmol), iodonium salt **1** (0.2 mmol), Na_2_CO_3_ (0.34 mmol), [Ru(bpy)_3_](PF_6_)_2_ (0.004 mmol), MeCN (2 mL), H_2_O (200 μL), under irradiation by blue LED (465 nm, 20 W) for 10 h. ^b^Determined by ^1^H NMR spectroscopy after isolation as a mixture of amides or a single amide using 1,2-dibromoethane as an internal standard. ^c^Not detected by GC–MS.

The limited scope of iodonium salts for arylations usually arose from the poor range of synthetically accessible symmetrical salts compared to their unsymmetrical analogues. However, the selective transfer of one of the aryl groups is a main challenge for unsymmetrical iodonium salts. Therefore, we moved to these to test the selectivity of aryl transfer under the established conditions. Since iodonium salts are prone to repeat the selectivity pattern of nucleophilic substitution in photoredox processes [35–41], we evaluated iodonium salts with common dummy ligands such as 2,4,6-trimethoxyphenyl (TMP) [42–43], sterically hindered 2,4,6-triisopropylphenyl, and mesityl ligands. The highest selectivity was achieved using aryl(2,4,6-trimethoxyphenyl)iodonium triflates **1n**–**p** yielding the desired amides **2ba**, **2bo**, and **2bp** in 25–42% yield. Competing amide **2bn** was not detected in the reaction mixture even by GC–MS analysis. In contrast, the mesityl-substituted salt **1l** gave both competing products **2ba** and **2bk** in a ≈4:1 ratio, while the 2,4,6-triisopropylphenyl derivative **2bm** yielded a mixture of products **2ba** and **2bm** in low yield (Scheme 3 and Supporting Information File 1, supplementary note 2).

Our experiments clearly demonstrated that electron-poor aryls gave better yields in case of both symmetrical and unsymmetrical iodonium salts. In general, such results cannot be associated with the stability of radical species, which does not significantly change for EWG- or EDG-substituted species except for extreme cases [44]. Additionally, such reactivity pattern cannot be explained only by steric factors since the yield dramatically dropped for *o*-methyl-substituted iodonium salt **1f** compared to *o*-halo-substituted salts **1g** and **1h**, which provided the best yields in the scope. Moreover, the strong correlation of the yield with the electronic effects in aryl rings was clearly shown in the experiment with iodonium salt **1q** (Scheme 3). We believe that the reason for the predominant transfer of the electron-poor ligands under the given conditions is due to a favorable formation of EWG-substituted aryl radicals from the iodonium cation, based on their reduction potentials and bond-dissociation energies calculated by Romanczyk and Kurek [45]. The reduction potential in SET reactions for iodonium salts with EWG-substituted aryls significantly differs from the ones with electron-rich aryls with 0.36 eV gap between (4-NO_2_C_6_H_4_)_2_I^+^ and (4-OMeC_6_H_4_)_2_I^+^ iodonium cations. If unsymmetric iodonium cations are considered where one of the aryls is phenyl and the other is a 4-substituted phenyl the bond-dissociation energy is 4.0 kcal/mol lower in case of (4-NO_2_C_6_H_4_) compared to (4-OMeC_6_H_4_) [45]. Therefore, despite the fact that literature data mostly suggest similar reactivity for aryl radicals with different substituents in the phenyl ring, the formation itself is more favorable for EWG-substituted radicals.

**Scheme 4:**
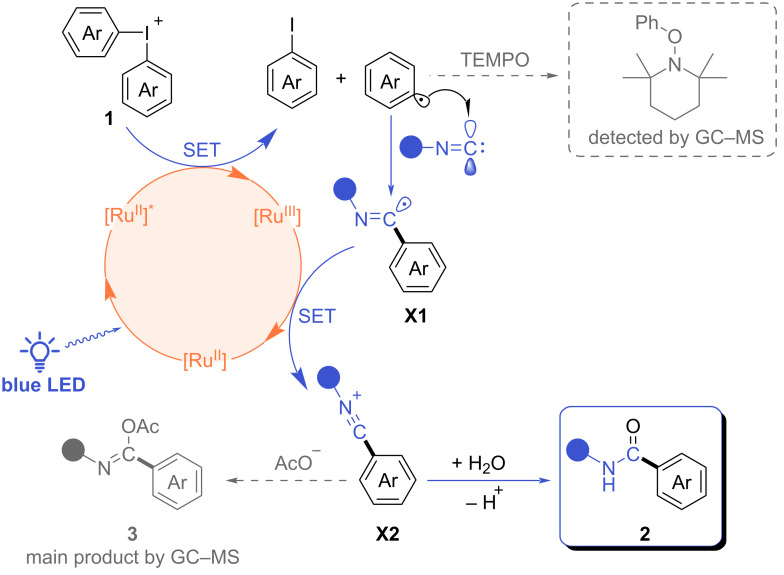
Proposed reaction mechanism.

To gain a deeper understanding of the reactivity pattern in the current transformation, a reaction mechanism was proposed taking into the account the known data and control experiments (Scheme 4). Upon irradiation with blue light, the Ru(II) catalyst undergoes photoexcitation, followed by an oxidative single-electron transfer (SET) process with the iodonium salt, leading to the generation of an aryl radical, aryl iodide, and Ru(III). The formation of the aryl radical was corroborated through a trapping experiment utilizing TEMPO as a radical scavenger (Scheme 4 and Supporting Information File 1, 5. Control experiments, Figure S18). The resulting aryl radical is subsequently captured by an isonitrile molecule, forming an imidoyl radical intermediate **X1**. The intermediate **X1** facilitates the reduction of the Ru(III) species back to Ru(II) thereby completing the photoredox cycle, with the formation of the cationic intermediate **X2**. We propose that bulky isonitriles effectively shield the radical or cationic centers in intermediates **X1** or **X2**, thereby preventing multiple additions of isonitrile to give the highest yields for benzamides **2be** and **2ge** among the scope of isonitriles. In the final step of the reaction, the addition of a water molecule from the reaction medium to **X2** occurs culminating in the formation of the final product **2** after deprotonation and tautomerization. The proposed mechanistic pathway is formally supported by conducting the reaction in the presence of NaOAc as a base, which resulted in the formation of the acetoxy derivative **3** attributable to the addition of acetate instead of water in the final step (Scheme 4 and Supporting Information File 1, 5. Control experiments, Figure S19). Additional control experiments excluded the possible arylation of formamide **4** by the iodonium salt (Supporting Information File 1, 5. Control experiments, Figure S20).

## Conclusion

In conclusion, a novel synthetic methodology for the preparation of benzamides from isonitriles and diaryliodonium salts has been proposed utilizing visible-light photoredox ruthenium-based catalysis. Both symmetrical and unsymmetrical diaryliodonium salts were evaluated and their reactivity was systematically analyzed in relation to the structural features of the iodonium salts and isonitriles. The study revealed that EWG-substituted diaryliodonium salts exhibited superior performance compared to EDG-substituted ones. Furthermore, the potential for selective transfer of a single aryl group from unsymmetrical diaryliodonium salts was demonstrated through the use of dummy ligands, such as 2,4,6-trimethoxyphenyl.

## Supporting Information

File 1 Experimental section, characterization data and control experiments.

## Data Availability

Data generated and analyzed during this study is available from the corresponding author upon reasonable request.
