# Supplementary material for: Photoredox-catalyzed arylation of isonitriles by diaryliodonium salts towards benzamides
Source: Beilstein J Org Chem. 2025 Jul 21;21:1480–8. doi: 10.3762/bjoc.21.110 (PMC12302481; doi:10.3762/bjoc.21.110)

BEILSTEIN JOURNAL OF ORGANIC CHEMISTRY

Supporting Information

for

Photoredox-catalyzed arylation of isonitriles by diaryliodonium salts towards benzamides

Nadezhda M. Metalnikova, Nikita S. Antonkin, Tuan K. Nguyen, Natalia S. Soldatova, Alexander V. Nyuchev, Mikhail A. Kinzhalov and Pavel S. Postnikov

Beilstein J. Org. Chem. **2025,** *21,* 1480–1488. doi:10.3762/bjoc.21.110

Experimental section, characterization data and control experiments

License and Terms: This is a supporting information file under the terms of the Creative Commons Attribution License (https://creativecommons.org/licenses/by/4.0). Please note that the reuse, redistribution and reproduction in particular requires that the author(s) and source are credited and that individual graphics may be subject to special legal provisions.
The license is subject to the *Beilstein Journal of Organic Chemistry* terms and conditions: (https://www.beilstein-journals.org/bjoc/terms)

Table of contents

1. General information S2
2. Initial consideration S4
   2.1 Solvent screening S4
   2.2 Preliminary and additional experiments S5
3. Synthesis of iodonium salts 1 ..... S10
4. Synthesis of amides 2 ..... S21
5. Control experiments S39

References S42

Spectral data S44

1. General information

Reagents were used as received from their commercial supplier (abcr, Acros Organics, Alfa Aesar, Sigma Aldrich, TCI, fluorochem, BLD pharm). MeCN was dried using standard procedures (distillation from CaH_2 under Ar atmosphere). THF was dried over Na and benzophenone until a blue color was reached then distilled under Ar atmosphere.[1] DMF was mixed with an appropriate amount of water and toluene, and fractionally distilled at atm. pressure, then under vacuum directly to 4 Å MS.[2] Unless otherwise stated, all yields refer to isolated yields of compounds estimated to be >95% pure as determined by ^{1}H NMR spectroscopy.

Thin-layer chromatography was performed on fluorescence indicator marked precoated silica gel 60 plates (Macherey-Nagel, ALUGRAM Xtra SIL G/UV254) and visualized by UV light (254 nm/365 nm). Column chromatography was performed on silica gel (0.040–0.063 mm) with the solvents given in the procedures.

^{1}H, ^{13}C and ^{19}F NMR spectra were recorded on Bruker AVANCE III HD (400 MHz), Bruker AVANCE III (500 MHz) spectrometers. The following abbreviations were used to describe splitting patterns: br = broad, s = singlet, d = doublet, t = triplet, q = quartet, quin. = quintet, sext. = sextet, sept. = septet, m = multiplet. Coupling constants *J* are given in hertz. Chemical shifts for ^{1}H NMR spectra were reported as δ (parts per million) relative to the residual signal of $CDCl_3$ at 7.26 ppm (s), DMSO-d_6 at 2.50 ppm (s), acetone-d_6 at 2.05 ppm (quin.). Chemical shifts for ^{13}C NMR spectra were reported as δ (parts per million) relative to the signal of $CDCl_3$ at 77.2 ppm (t), DMSO-d_6 at 39.5 ppm (sept.), acetone-d_6 at 29.8 ppm (sept.).

HR–ESI mass spectra were recorded on a Bruker impact II. EI mass spectra were recorded on an Agilent 7820A. All signals were reported with the quotient from mass to charge *m*/*z*. A solvent for HR–ESI mass spectra measurements is specified for each compound.

Melting points were determined on a Büchi M-5600 Melting Point apparatus or on a melting point apparatus SMP30 with a heating rate of 2 °C/min. The melting points were reported in °C. Most of the iodonium salts underwent changes in appearance (e.g. softening) before final melting/decomposition.

Photochemical experiments were carried out with a homemade setup (Figure S1A). The reaction mixture was irradiated by 20 W blue LED (465 nm) coiled around a glass tube. Water cooled to 14 °C was supplied between the glass tube and the screw cap tube with the reaction mixture. The screw cap tube is inside a glass tube (Figure S1B).

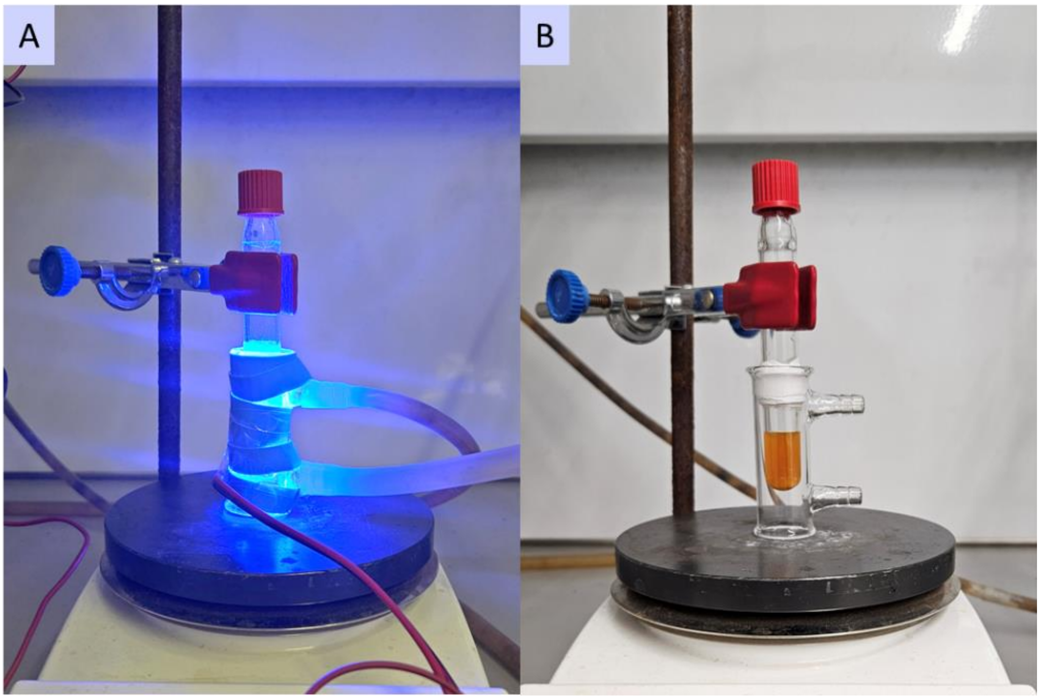

Figure S1. Overview of the reaction setup. (A) Photoreactor with power on. (B) A screw cap tube arrangement inside the glass tube

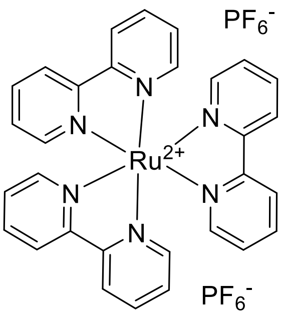

[Ru(bpy)$_3$](PF$_6$)$_2$

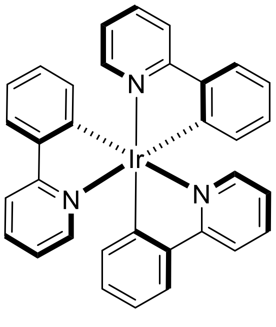

***fac*-Ir(ppy)$_3$**

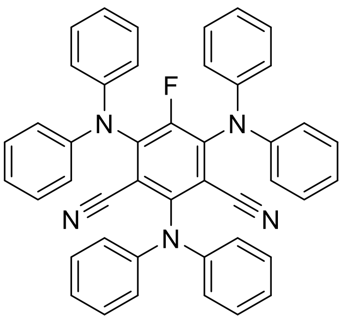

3DPAFIPN

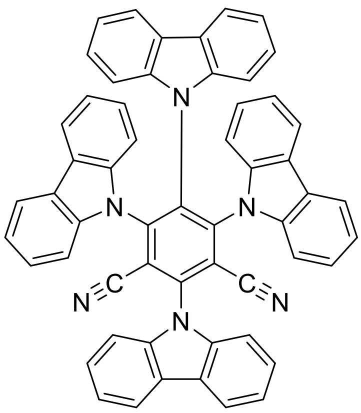

4CzIPN

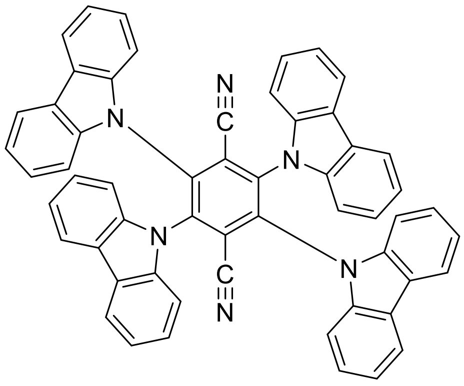

4CzTPN

Figure S2. Structure of photocatalysts

2. Initial consideration

2.1 Solvent screening[a]

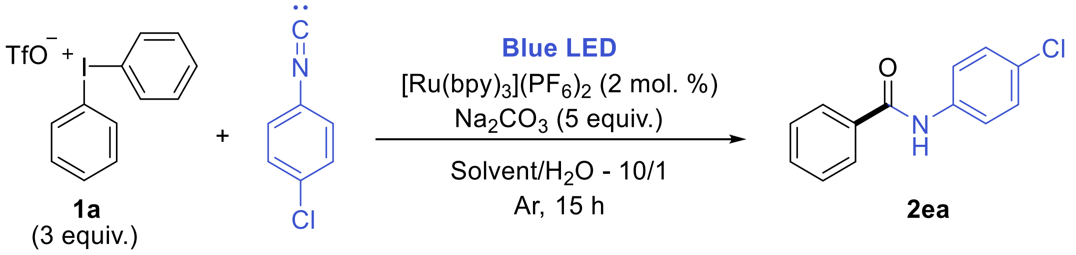

Solvents

**Solvent/H_2O**	**MeCN/H_2O**	**DMF/H_2O[c*]**	**Acetone/H_2O[c*]**	**THF/H_2O[c*]**
**Yield**	37%[b]	15%[d]	23%[d]	traces[e]

MeCN:H_2O ratio

**MeCN/H_2O[f]**	**5/1**	**10/1**	**20/1**
**Yield[b]**	34%	37%	28%

[a]Reaction conditions: 1-chloro-4-isocyanobenzene (0.1 mmol), diphenyliodonium triflate (**1a**, 0.3 mmol), Na_2CO_3 (0.5 mmol), $[Ru(bpy)_3](PF_6)_2$ (0.002 mmol), MeCN (1 mL), H_2O (100 μL) under irradiation by blue LED (465 nm, 20 W) for 15 h. [b]Determined by ^{1}H NMR using 1,3,5-triisopropylbenzene as an internal standard. [c]$[Ru(bpy)_3](PF_6)_2$ (10 mol %, 0.01 mmol) [d]Isolated yield via column chromatography with gradient elution hexane/EtOAc 30:1 → 10:1. [e]According to GC–MS. [f]MeCN (1 mL)/H_2O (50 – 200 μL).

Even reactions with increased amount of the catalyst in other solvents apart from MeCN demonstrated lower yields of the product* **2ea.

All the reactions above afforded *N*-(4-chlorophenyl)benzamide **2ea** as an off-white solid, mp = 190 – 191 °C (lit. 188 – 190 °C).[3] The analytical data is in accordance with previously published ones.[4]

^{1}H NMR (400 MHz, $CDCl_3$) δ 7.86 (d, *J* = 7.6 Hz, 2H), 7.79 (s, 1H), 7.62 – 7.56 (m, 3H), 7.50 (t, *J* = 7.6 Hz, 2H), 7.34 (d, *J* = 8.4 Hz, 2H).

^{13}C NMR (100 MHz, $CDCl_3$) δ 165.8, 136.6, 134.8, 132.2, 129.7, 129.3, 129.0, 127.1, 121.5.

2.2 Preliminary and additional experiments

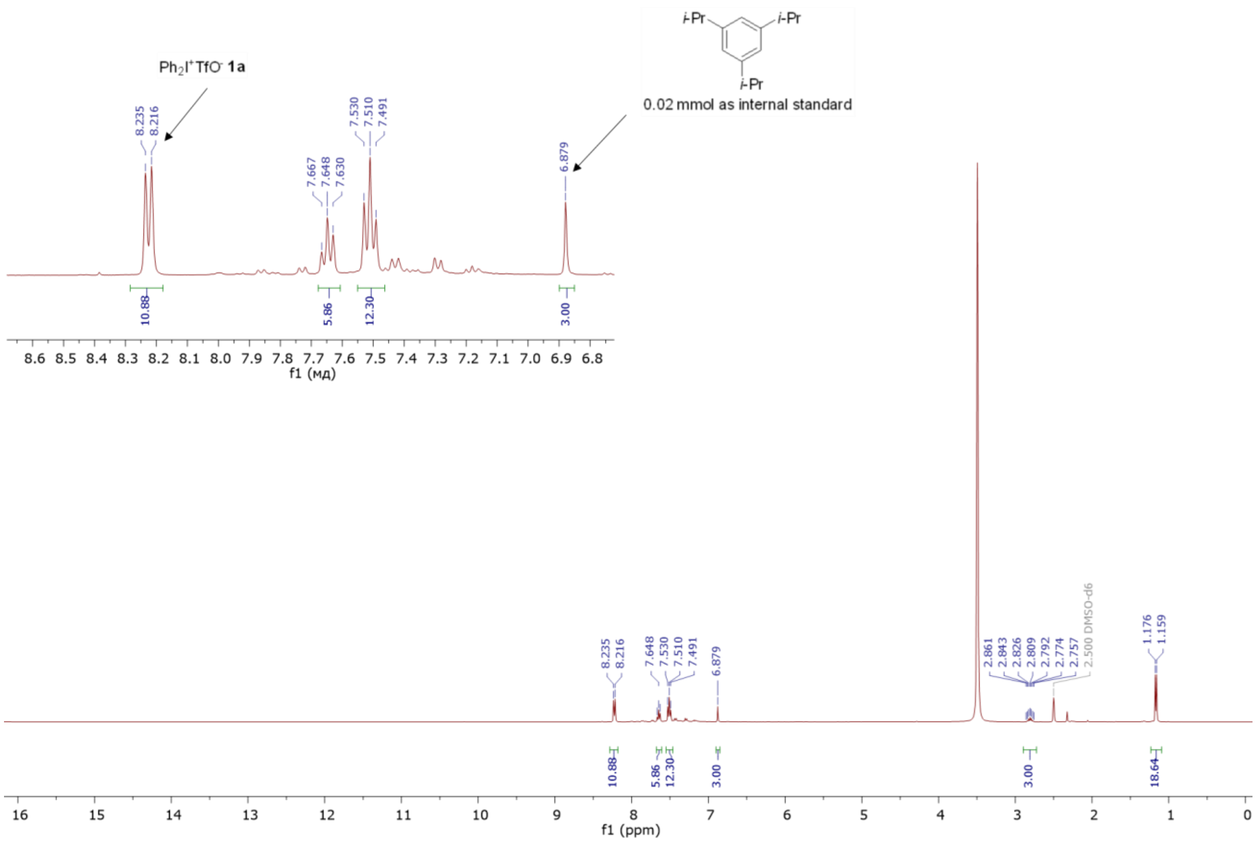

Figure S3. The amount of iodonium salt **1a** remained in the reaction without a catalyst (main text, Table 1, entry 1)

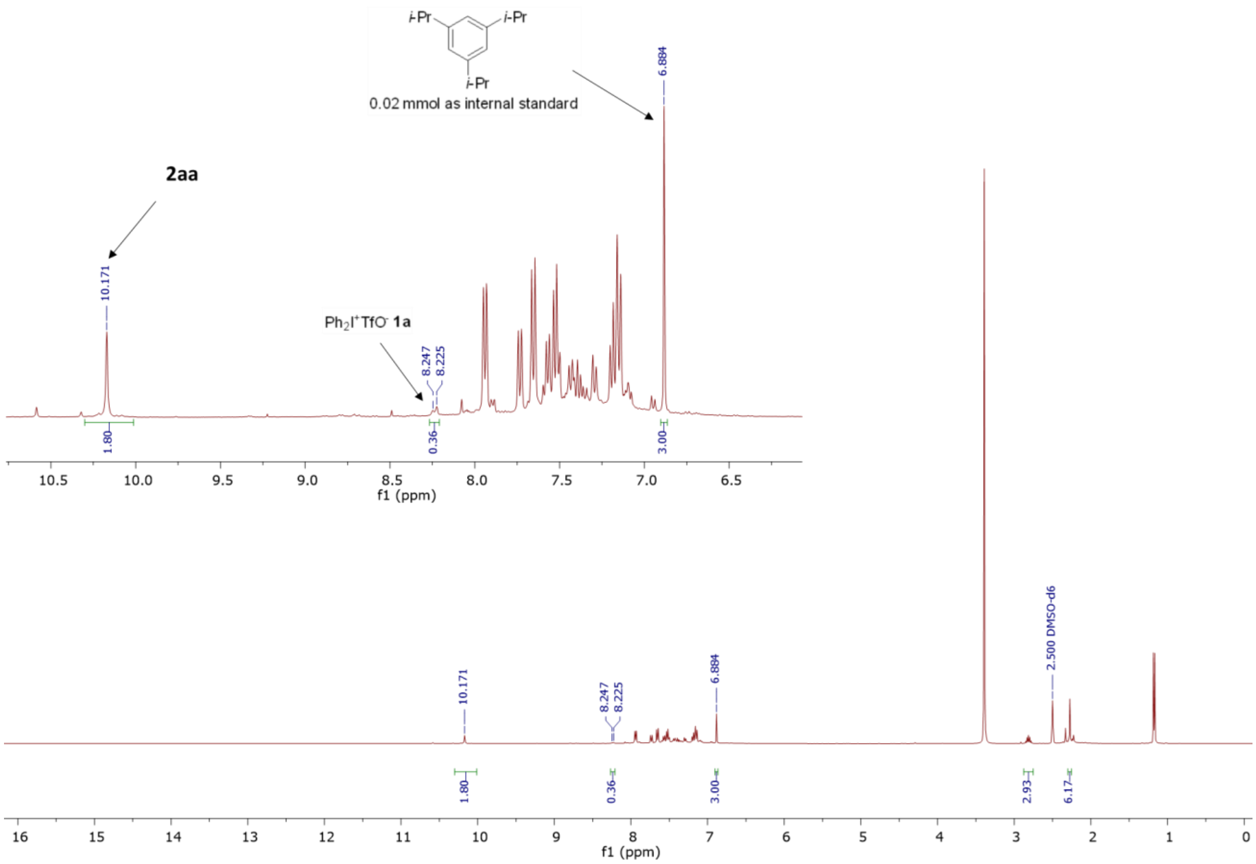

Figure S4. NMR yield and the amount of iodonium salt **1a** remained in the reaction under optimal conditions (Table 1, entry 2)

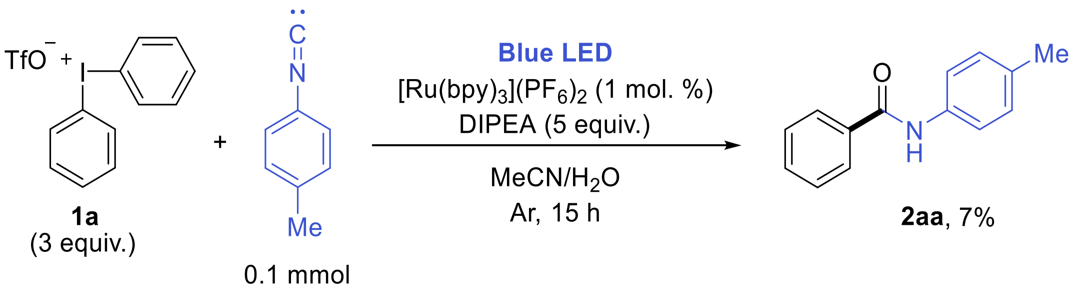

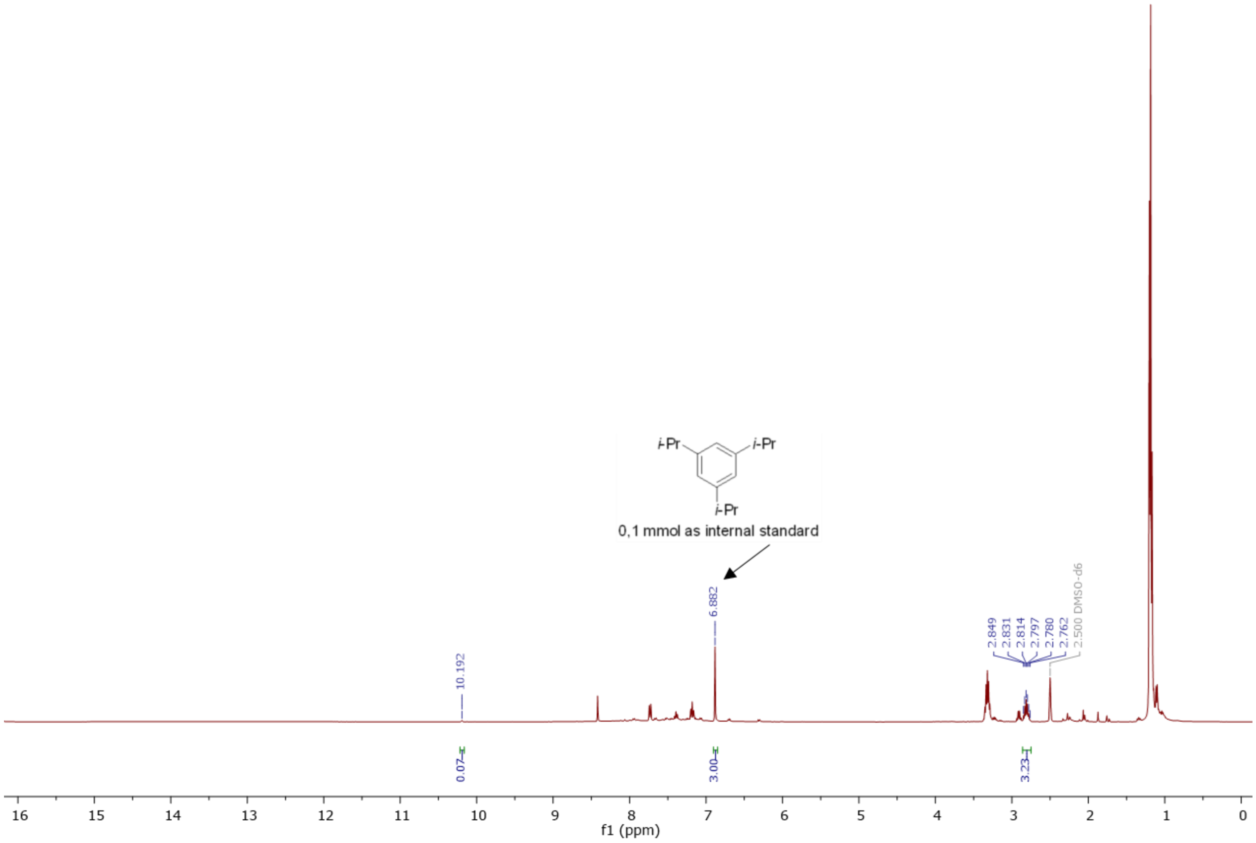

Figure S5. NMR yield of **2aa** with DIPEA as an example of organic base

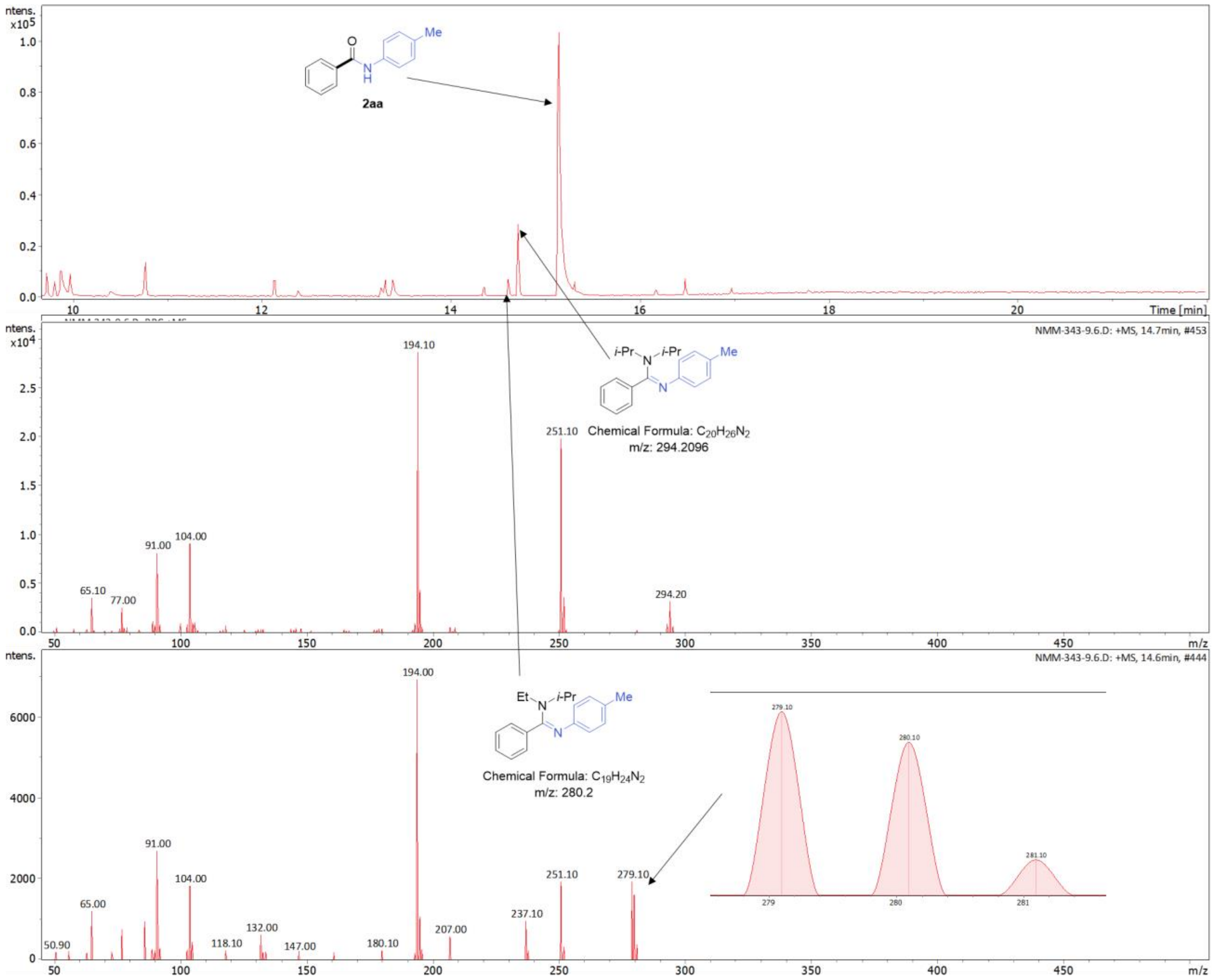

Figure S6. GC–MS data for the reaction in a presence of DIPEA

Supplementary note 1

We observed an insoluble film on the wall of the tube after reactions with substantial excess of isonitrile and the formation of products of multiple addition of isonitrile in such reactions (**Figure S7**). We concluded that higher concentration of isonitrile generally led to oligomerization.

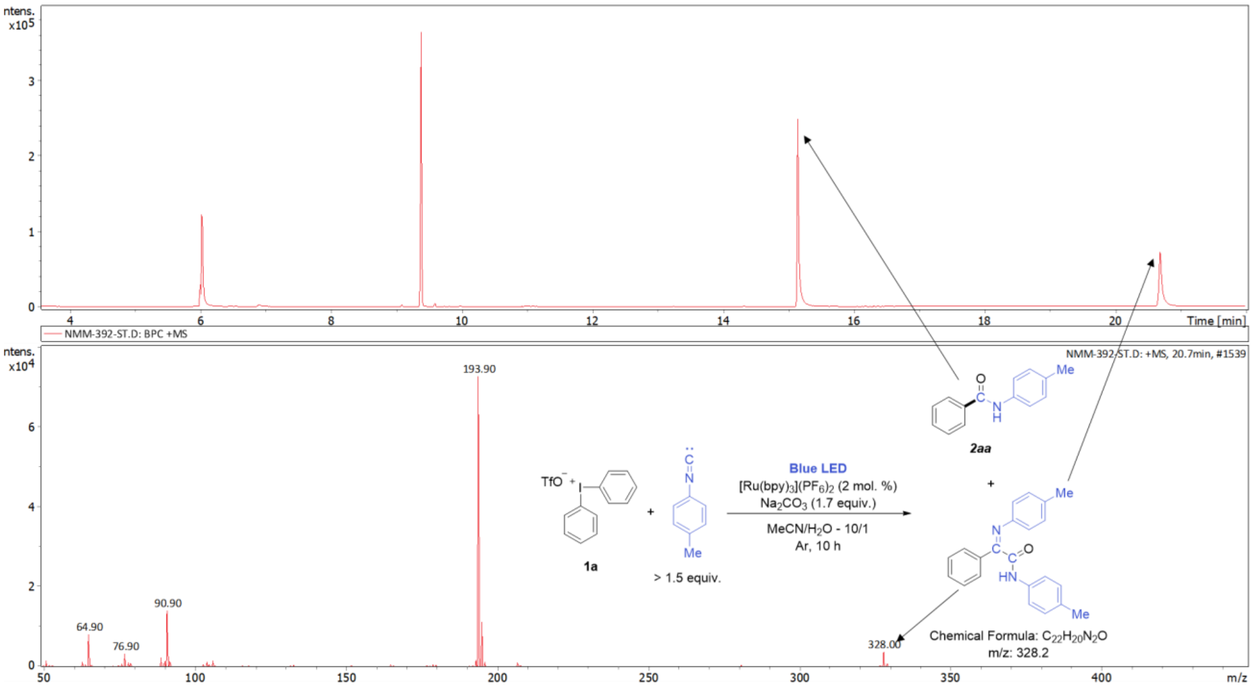

Figure S7. GC–MS data with polyaddition of isonitrile

Table S1. Additional optimization

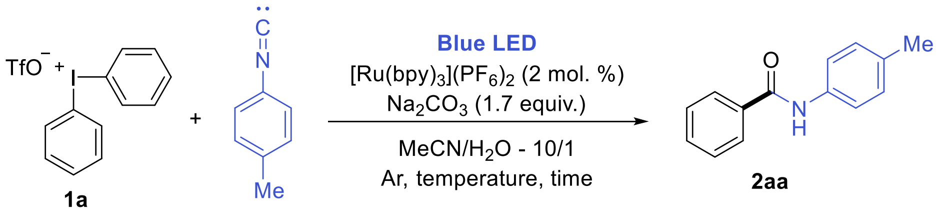

Deviation from standard conditions (Main Text, Table 1, entry 2)	Yield of 2aa
Reaction time – 15 h	37%
Temperature of cooling water – 4 °C	36%
Temperature of cooling water – 35 °C	28%

3. Synthesis of iodonium salts 1

General procedure (GP1): according to the literature procedure.[5] Iodoarene (1.0 equiv, 10.0 mmol) was dissolved in DCM (50 mL) and *m*CPBA was added (1.13 equiv, 11.3 mmol, 2.89 g, 70%) neat. Then, the arene (1.13 equiv, 11.3 mmol) was added and the reaction mixture was stirred for 5 min. Afterwards the reaction mixture was cooled in an ice–water bath and TfOH (3.0 equiv, 30.0 mmol, 2.66 mL) was added dropwise. The mixture was allowed to stir overnight at room temperature. Then, the solvent was removed under reduced pressure. The product was precipitated by addition of Et_2O and stored at −30 °C for 1 h. Then, the precipitate was filtered, washed with cold Et_2O (3 × 15 mL) and dried under vacuum to give diaryliodonium triflate **1**.

General procedure (GP2): according to the modified literature procedure.[6] Iodoarene (1.0 equiv, 10.0 mmol) was dissolved in $CHCl_3$ (10 mL). Then, *m*CPBA (1.1 equiv, 11.0 mmol, 2.71 g, 70%) and *p*-TsOH·H_2O (1.0 equiv, 10.0 mmol, 2.09 g) were added neat. The reaction mixture was allowed to stir for 2 h at room temperature. Then, the solvent was removed under reduced pressure and the product was precipitated by the addition of Et_2O. The precipitate was filtered and washed with Et_2O (3 × 10 mL). The product was dried under vacuum to give [hydroxy(tosyloxy)iodo]arene. Then, the prepared [hydroxy(tosyloxy)iodo]arene (1.0 equiv, 2.0 mmol) was suspended in MeCN. BF_3·Et_2O (4.0 equiv, 8.0 mmol, 1.02 mL) was added dropwise to the mixture. After full dissolution of the substrate arylboronic acid (1.1 equiv, 2.2 mmol) was added and the reaction mixture was stirred overnight. TfOH (3.0 equiv, 6.0 mmol, 0.9 mL) was added dropwise and stirring was continued for 30 min. The solvent was removed under reduced pressure and the product was isolated by column chromatography on silica (gradient elution: DCM/MeOH 100:1 → 10:1) to give diaryliodonium triflate **1**.

General procedure (GP3): according to the literature procedure.[7] Iodoarene (1.0 equiv, 5.0 mmol) and *p*-TsOH·H_2O (1.05 equiv, 5.25 mmol, 0.90 g, 70%) was dissolved in MeCN (50 mL) and *m*CPBA was added (1.13 equiv, 11.3 mmol, 2.89 g, 70%) neat. The reaction mixture was stirred at 77 °C for 30 min. Then, 1,3,5-trimethoxybenzene (1.05 equiv, 5.25 mmol, 0.88 g) was added and the reaction mixture was stirred at 77 °C for 5 min. The solvent was removed under reduced pressure. The product was precipitated by the addition of Et_2O, washed with cold Et_2O (3 × 15 mL) and dried in the air at room temperature to give aryl(2,4,6-trimethoxyphenyl)iodonium tosylate. Afterwards aryl(2,4,6-trimethoxyphenyl)iodonium tosylate was dissolved in DCM (50 mL). The resulted solution was washed with cooled aqueous solution of NaOTf (1 M, 100 mL)

prepared by mixing solutions of NaOH (100 mmol, 4.0 g) in H_2O (50 mL) and TfOH (100 mmol, 8.8 mL) in H_2O (50 mL). The combined organic layer was dried over $MgSO_4$ and the solvent was removed under reduced pressure. The product was precipitated by the addition of Et_2O, filtered, washed with cold Et_2O (3 × 15 mL) and dried in the air at room temperature to give aryl(2,4,6-trimethoxyphenyl)iodonium triflate **1**. *It is not recommended to dry under vacuum since for some salts decomposition occurred!*

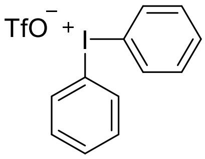

1a: according to ***GP1*** iodobenzene (10.0 mmol, 1.2 mL) reacted with benzene (11.3 mmol, 1.0 mL) to give diphenyliodonium triflate (**1a**) as a white solid, 4.10 g (95%). mp = 170 – 172 °C (lit. 169 – 173 °C).[7] The analytical data is in accordance with previously published.[7]

^{1}H NMR (400 MHz, DMSO-*d₆*) δ 8.25 (d, *J* = 8.0 Hz, 4H), 7.67 (t, *J* = 6.8 Hz, 2H), 7.53 (t, *J* = 6.8 Hz, 4H).

^{13}C NMR (100 MHz, DMSO-d_6) δ 135.2, 132.1, 131.8, 120.7 (q, *J* = 320.0 Hz), 116.5.

^{19}F NMR (376 MHz, DMSO-d_6) δ -77.7.

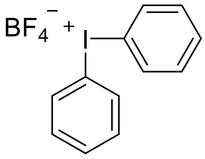

1a-BF₄: according to modified literature procedure,[8] (diacetoxyiodo)benzene (2.0 mmol, 644 mg) was dissolved in DCM (7 mL) and $BF_3 \cdot Et_2O$ (2.75 equiv, 5.5 mmol, 0.7 mL) was added under cooling with an ice–water bath (5–10 °C). After 20 min of stirring phenylboronic acid (1.05 equiv, 2.1 mmol, 256 mg) was added neat under cooling and the reaction was allowed to stir overnight at room temperature. The solvent was removed under reduced pressure, Et_2O (15 mL) was added to precipitate the product. The solid was filtered, washed with Et_2O (3 × 10 mL) and dried under vacuum to give diphenyliodonium tetrafluoroborate (**1a-BF₄**) as an off-white solid, 0.73 g (98%). mp = 124 – 125 °C (lit. 132 – 134 °C).[9] The analytical data is in accordance with previously published.[9]

^{1}H NMR (400 MHz, DMSO-*d6*) δ 8.25 (d, *J* = 8.0 Hz, 4H), 7.67 (t, *J* = 7.6 Hz, 2H), 7.53 (t, *J* = 7.6 Hz, 4H).

^{13}C NMR (100 MHz, DMSO-*d6*) δ 135.2, 132.1, 131.8, 116.5.

^{19}F NMR (376 MHz, DMSO-d_6) δ -148.2.

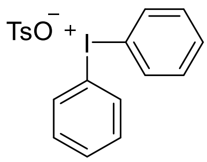

1a-TsO: according to the literature procedure[10] diphenyliodonium tetrafluoroborate (**1a-BF**$_4$, 1.0 mmol, 367 mg) was dissolved in DCM (15 mL) and washed with NaOTs solution in water (3 × 30 mL, 10 equiv each wash) and the organic layer was evaporated without drying to give diphenyliodonium tosylate (**1a-TsO**) as a white solid, 0.104 g (23%). mp = 172 – 173 °C (lit. 178 – 179 °C).[11] The analytical data is in accordance with previously published.[11]

^{1}H NMR (400 MHz, DMSO-*d6*) δ 8.25 (d, *J* = 7.6 Hz, 4H), 7.66 (t, *J* = 7.6 Hz, 2H), 7.53 (t, *J* = 7.6 Hz, 4H), 7.47 (d, *J* = 8.0 Hz, 2H), 7.11 (d, *J* = 8.0 Hz, 2H), 2.28 (s, 3H).

^{13}C NMR (100 MHz, DMSO-*d6*) δ 145.8, 137.5, 135.2, 132.0, 131.7, 128.0, 125.5, 116.5, 20.8.

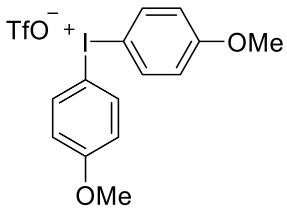

1b: Preparation of **1b** is based on the previously reported procedure.[7] Anisole (3.5 equiv, 20.0 mmol, 2.2 mL), *m*CPBA (70%, 2.0 equiv, 14.3 mmol, 3.53 g) and *p*-TsOH·H_2O (3.4 equiv, 19.4 mmol, 3.69 g) was dissolved in DCM (50 mL). Iodine (1.0 equiv, 5.7 mmol, 1.43 g) was added and the solution was stirred overnight at room temperature. Then, the solvent was removed under reduced pressure, the product was precipitated by the addition of Et_2O, filtered and washed with Et_2O (3 × 15 mL). The obtained bis(4-methoxyphenyl)iodonium tosylate was dried in the air at room temperature. Afterwards bis(4-methoxyphenyl)iodonium tosylate was suspended in DCM (50 mL). The resulted suspension was washed with a cooled aqueous solution of NaOTf (1 M, 100 mL) prepared by mixing solutions of NaOH (100 mmol, 4.0 g) in H_2O (50 mL) and TfOH (100 mmol, 8.8 mL) in H_2O (50 mL). The combined organic layer was dried over $MgSO_4$ and the solvent was removed under reduced pressure. The product was precipitated by the addition of Et_2O, filtered, washed with cold Et_2O (3 × 15 mL) and dried under vacuum to give bis(4-methoxyphenyl)iodonium triflate (**1b**) as a white solid, 3.10 g (63%). mp = 131 – 133 °C (lit. 116 – 125 °C).[7] The analytical data is in accordance with previously published.[7]

^{1}H NMR (400 MHz, DMSO-*d*$_6$) δ 8.13 (d, *J* = 8.4 Hz, 4H), 7.06 (d, *J* = 8.4 Hz, 4H), 3.79 (s, 6H).

^{13}C NMR (100 MHz, DMSO-d_6) δ 161.9, 136.9, 120.7 (q, J = 320.0 Hz), 117.4, 106.0, 55.7.
^{19}F NMR (376 MHz, DMSO-d_6) δ -77.7.

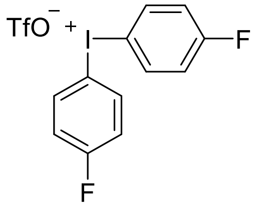

1c: Preparation of **1c** is based on the previously reported procedure.[5] Iodine (1.0 equiv, 10.0 mmol, 1.43 g) and *m*CPBA (3.0 equiv, 30.0 mmol, 3.2 g, 70%,) was dissolved in DCM (84 mL). Afterwards the reaction mixture was cooled in ice–water bath and fluorobenzene (10.0 equiv, 100.0 mmol, 9.39 mL) was added. After 5 min of stirring TfOH (3.8 equiv, 38.0 mmol, 3.36 mL) was added dropwise. The mixture allowed to stir overnight at room temperature. The solvent was removed under reduced pressure. The product was precipitated by the addition of Et_2O, stored at −30 °C for 1 h, filtered, washed with cold Et_2O (3 × 15 mL) and dried under vacuum to give bis(4-fluorophenyl)iodonium triflate (**1c**) as a white solid, 5.25 g (56%). mp = 166 – 168 °C (lit. 168 – 170 °C).[12] The analytical data is in accordance with previously published.[12]
^{1}H NMR (400 MHz, DMSO-d_6) δ 8.32 (dd, J = 9.2, 5.2 Hz, 4H), 7.42 (t, J = 8.8 Hz, 4H).
^{13}C NMR (100 MHz, DMSO-d_6) δ 164.0 (d, J = 250.0 Hz), 138.06 (d, J = 9.0 Hz), 120.7 (q, J = 320.0 Hz), 119.3 (d, J = 23.0 Hz), 111.2 (d, J = 3.0 Hz).
^{19}F NMR (376 MHz, DMSO-d_6) δ -77.8, -106.6 – 106.6 (m).

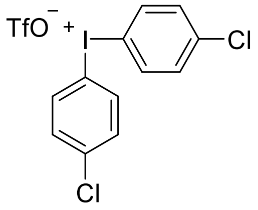

1d: according to ***GP1*** 1-chloro-4-iodobenzene (10.0 mmol, 2.39 g) reacted with chlorobenzene (11.3 mmol, 1.2 mL) to give bis(4-chlorophenyl)iodonium triflate (**1d**) as a white solid, 4.39 g (88%). mp = 189 – 190 °C (lit. 183 – 185 °C).[7] The analytical data is in accordance with previously published.[7]
^{1}H NMR (400 MHz, DMSO-d_6) δ 8.26 (d, J = 8.0 Hz, 4H), 7.63 (d, J = 8.0 Hz, 4H).
^{13}C NMR (100 MHz, DMSO-d_6) δ 137.5, 137.0, 131.8, 120.7 (q, J = 320.0 Hz), 114.7.
^{19}F NMR (376 MHz, DMSO-d_6) δ -77.8.

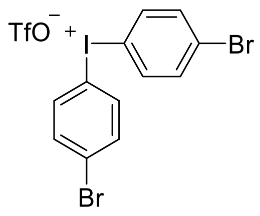

1e: according to ***GP1*** 1-bromo-4-iodobenzene (10.0 mmol, 2.83 g) reacted with bromobenzene (11.3 mmol, 1.2 mL) for 2 h to give bis(4-bromophenyl)iodonium triflate (**1e**) as a white solid, 5.30 g (90%). mp = 210 – 212 °C (lit. 181 – 184 °C).[7] The analytical data is in accordance with previously published.[7]

^{1}H NMR (400 MHz, DMSO-d_6) δ 8.18 (d, *J* = 8.4 Hz, 4H), 7.77 (d, *J* = 8.4 Hz, 4H).

^{13}C NMR (100 MHz, DMSO-d_6) δ 137.1, 134.7, 126.4, 120.7 (q, *J* = 320.3 Hz), 115.4.

^{19}F NMR (376 MHz, DMSO-d_6) δ -77.8.

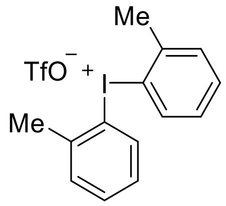

1f: according to ***GP2*** 1-iodo-2-methylbenzene (10.0 mmol, 2.18 g) reacted with *m*CPBA (70%, 11.0 mmol, 2.71 g) and *p*-TsOH·H_2O (11.0 mmol, 2.09 g) to give hydroxy(2-methylphenyl)iodonium tosylate as a white solid 3.98 g (98%). Reaction of hydroxy(2-methylphenyl)iodonium tosylate (2.0 mmol, 0.81 g) with (2-methylphenyl)boronic acid (2.2 mmol, 0.30 g) afforded bis(2-methylphenyl)iodonium triflate (**1f**) as a white solid, 0.44 g (48%). mp = 175 – 178 °C (lit. 170 – 171 °C).[13] The analytical data is in accordance with previously published.[13]

^{1}H NMR (400 MHz, DMSO-d_6) δ 8.32 (d, *J* = 8.4 Hz, 2H), 7.60 – 7.56 (m, 4H), 7.32 – 7.28 (m, 2H), 2.61 (s, 6H).

^{13}C NMR (100 MHz, DMSO-d_6) δ 140.6, 137.2, 132.8, 131.6, 129.3, 120.7 (q, *J* = 320.0 Hz), 120.6, 25.0.

^{19}F NMR (376 MHz, DMSO-d_6) δ -77.8.

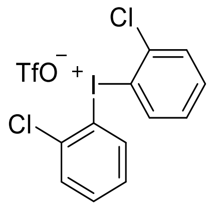

1g: according to ***GP2*** 1-chloro-2-iodobenzene (10.0 mmol, 2.38 g) reacted with *m*CPBA (70%, 11.0 mmol, 2.71 g) and *p*-TsOH·H_2O (11.0 mmol, 2.09 g) to give hydroxy(2-chlorophenyl)iodonium tosylate as a white solid 4.14 g (97%). Reaction of hydroxy(2-chlorophenyl)iodonium tosylate (2.0 mmol, 0.85 g) with (2-chlorophenyl)boronic acid (2.2 mmol, 0.34 g) afforded bis(2-chlorophenyl)iodonium triflate (**1g**) as a white solid, 0.67 g (67%). mp = 193 – 195 °C.

^{1}H NMR (400 MHz, DMSO-d_6) δ 8.53 (d, J = 8.0 Hz, 1H), 7.84 (d, J = 8.0 Hz, 1H), 7.70 (t, J = 7.6 Hz, 1H), 7.50 (t, J = 7.6 Hz, 1H).
^{13}C NMR (100 MHz, DMSO-d_6) δ 139.0, 136.0, 134.8, 130.5, 130.2, 120.7 (q, J = 320.0 Hz), 119.6.
^{19}F NMR (376 MHz, DMSO-d_6) δ -77.8.
HRMS (positive mode, MeCN) calcd. for $C_{12}H_8Cl_2I^+$ ($[M+H]^+$) 348.9043 (found 348.9043).
HRMS (negative mode, MeCN) calcd. for $CF_3O_3S^-$ ($[M+H]^-$) 148.9525 (found 148.9524).

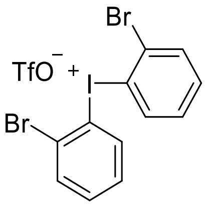

1h: according to ***GP2*** 1-bromo-2-iodobenzene (10.0 mmol, 2.38 g) reacted with *m*CPBA (70%, 11.0 mmol, 2.71 g) and *p*-TsOH·H_2O (11.0 mmol, 2.09 g) to give hydroxy(2-bromophenyl)iodonium tosylate as a white solid 4.62 g (98%). Reaction of hydroxy(2-bromophenyl)iodonium tosylate (2.0 mmol, 0.94 g) with (2-bromophenyl)boronic acid (2.2 mmol, 0.44 g) afforded bis(2-bromophenyl)iodonium triflate (**1h**) as a white solid, 0.76 g (65%) mp = 209 – 210 °C.
^{1}H NMR (400 MHz, DMSO-d_6) δ 8.50 (d, J = 7.6 Hz, 2H), 7.98 (d, J = 7.6 Hz, 2H), 7.62 (t, J = 7.6 Hz, 2H), 7.54 (t, J = 7.6 Hz, 2H).
^{13}C NMR (100 MHz, DMSO-d_6) δ 139.2, 134.7, 133.9, 130.6, 127.0, 122.9, 120,7 (q, J = 320.0 Hz).
^{19}F NMR (376 MHz, DMSO-d_6) δ -77.8.
HRMS (positive mode, MeCN) calcd. for $C_{12}H_8Br_2I^+$ ($[M+H]^+$) 438.8012 (found 438.8014).
HRMS (negative mode, MeCN) calcd. for $CF_3O_3S^-$ ($[M+H]^-$) 148.9525 (found 148.9527).

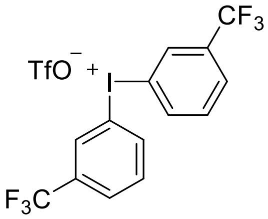

1i: Preparation of **1i** is based on the previously reported procedure.[7] Iodine (1.0 equiv, 2.84 mmol, 0.72 g) and $NaIO_4$ (1.5 equiv, 4.3 mmol, 0.92 g) were added to $H_2SO_{4conc.}$ (10 mL) and stirred at 75 °C for 1 h. Reaction mixture was cooled with ice-water bath and benzotrifluoride (9.1 equiv, 26 mmol, 3.8 mL) was added dropwise and obtained mixture was stirred overnight at room temperature. Then ice was added to the reaction mixture (total volume after melting was approximately 50 mL). TfOH (7.0 equiv, 20.0 mmol, 1.77 mL) was added. Resulted mixture was extracted with DCM (3 × 20 mL) and combined organic phase was dried over Mg_2SO_4 and solvent was

removed under reduced pressure. The product was precipitated by Et_2O (2 mL) and hexane (18 mL), filtered and washed with hexane (3 × 10 mL). Afterwards the precipitate dried under vacuum to give bis(3-(trifluoromethyl)phenyl)iodonium triflate (**1i**) as a white solid, 1.83 g (45%). mp = 109 – 110 °C (lit. 95 – 102 °C).[7] The analytical data is in accordance with previously published.[7]

^{1}H NMR (400 MHz, DMSO-*d*$_6$) δ 8.83 (s, 2H), 8.62 (d, *J* = 8.0 Hz, 2H), 8.07 (d, *J* = 8.0 Hz, 2H), 7.79 (t, *J* = 8.0 Hz, 2H).

^{13}C NMR (100 MHz, DMSO-*d*$_6$) δ 139.4, 132.8, 132.0 (q, *J* = 4.0 Hz), 131.3 (q, *J* = 33.0 Hz), 129.1 (q, *J* = 3.0 Hz), 122.9 (q, *J* = 271.7 Hz), 120.7 (q, *J* = 320.0 Hz), 117.2.

^{19}F NMR (376 MHz, DMSO-*d*$_6$) δ -61.3, -77.8.

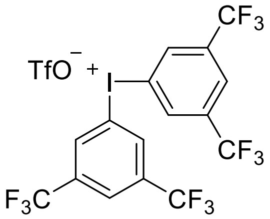

1j: Preparation of **1j** is based on the previously reported procedure.[14] A 100 mL round-bottomed flask was charged with the mixture of TfOH (3 mL) and iodine (1.0 equiv, 0.66 mmol, 0.17 g). Then $NaIO_4$ (1.5 equiv, 1 mmol, 0.2 g) was added to the mixture under stream of argon, reaction vessel was flushed with argon and stirred at room temperature for 48 h. Afterwards, the reaction mixture was cooled in ice-water bath and 1,3-bis(trifluoromethyl)benzene (9.1 equiv, 6.0 mmol, 0.95 mL) was added dropwise. The obtained mixture was allowed to stir at room temperature for 24 h. Then ice was added to the reaction mixture (total volume after melting was approximately 50 mL). The reaction mixture was extracted with EtOAc (3 × 20 mL). The combined organic phase was dried over Na_2SO_4 and the solvent was removed under reduced pressure. A mixture of hexane (10 mL) and Et_2O (2 mL) was added to the residue. The product was filtered, washed with hexane (3 × 10 mL) and dried under vacuum to give bis(3,5-bis(trifluoromethyl)phenyl)iodonium triflate as a white solid, 0.79 mg (49%). mp = 254 – 255 °C (dec.) (lit. 198 – 203 °C).[14] The analytical data is in accordance with previously published.[14]

^{1}H NMR (400 MHz, Acetone-*d*$_6$) δ 9.21 (s, 4H), 8.43 (s, 2H).

^{13}C NMR (100 MHz, Acetone-*d*$_6$) δ 137.8 (q, *J* = 4.0 Hz), 134.4 (q, *J* = 34.0 Hz), 127.7 (sept. *J* = 3.8 Hz), 123.1 (q, *J* = 271.3 Hz), 121.8 (q, *J* = 318.3 Hz), 116.5.

^{19}F NMR (376 MHz, Acetone *d*$_6$) δ -63.4, -79.1.

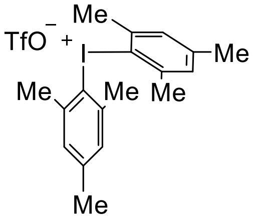

1k: Preparation of **1k** is based on the previously reported procedure.[7] A 100 mL round-bottomed flask was charged with iodine (1.0 equiv, 2.5 mmol, 0.64 g), Oxone (4.0 equiv, 10.0 mmol, 6.17 g) and mesitylene (4.3 equiv, 10.8 mmol, 1.5 mL). MeCN was added (10 mL). Afterwards, $H_2SO_{4conc.}$ (2 mL) was added to the mixture. The reaction mixture was allowed to stir overnight. Then, a solution of TfOH (4.0 equiv, 10.0 mmol, 0.88 mL) in water (10 mL) was added and the reaction mixture was extracted with DCM (3 × 20 mL) and the combined organic phase was dried over Mg_2SO_4.The solvent was removed under reduced pressure. The product was precipitated by the addition of Et_2O (2 mL) and hexane (18 mL), filtered and washed with hexane (3 × 5 mL). Afterwards, the precipitate was dried under vacuum to give dimesityliodonium triflate (**1k**) as an off-white solid, 1.20 g (47%). mp = 197 – 198 °C (dec.) (lit. 189 – 191 °C).[7] The analytical data is in accordance with previously published.[7]

^{1}H NMR (400 MHz, DMSO-*d6*) δ 7.19 (s, 4H), 2.46 (s, 12H), 2.29 (s, 6H).

^{13}C NMR (100 MHz, DMSO-*d6*) δ 142.8, 141.9, 130.3, 120.7 (*J* = 320.0 Hz), 118.9, 25.3, 20.4.

^{19}F NMR (376 MHz, DMSO-*d6*) δ -77.8.

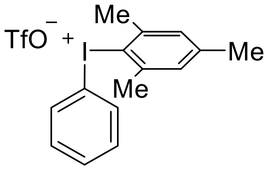

1l: Preparation of **1l** is based on the previously reported procedure.[15] Mesitylene (1.1 equiv, 11.0 mmol, 1,53 mL) was added to the suspension of iodosobenzene diacetate (1.0 equiv, 10.0 mmol, 3.22 g) in DCM (20 mL). Then the reaction mixture was cooled with an ice–water bath and TfOH (1.1 equiv, 11.0 mmol, 0.97 mL) was added dropwise to the stirred solution. The cooling bath was removed and the reaction mixture was stirred for 2 h at room temperature. The solvent was removed under reduced pressure and the product was precipitated by Et_2O. The solid was filtered and washed with Et_2O (3 × 15 mL). The solid was dried under vacuum to give mesityl(phenyl)iodonium triflate (**1l**) as a white solid, 4.60 g (97%). mp = 150 – 151 °C (lit. 149 – 150 °C).[16] The analytical data is in accordance with previously published.[15]

^{1}H NMR (400 MHz, DMSO-*d6*) δ 7.98 (d, *J* = 7.2 Hz, 2H), 7.65 – 7.61 (m, 1H), 7.52 – 7.48 (m, 2H), 7.22 (s, 2H), 2.60 (s, 6H), 2.29 (s, 3H).

^{13}C NMR (100 MHz, DMSO-d_6) δ 143.1, 141.6, 134.5, 131.9, 131.8, 129.8, 122.6, 120.7 (q, J = 320.3 Hz), 114.5, 26.3, 20.5.
^{19}F NMR (376 MHz, DMSO-d_6) δ -77.7.

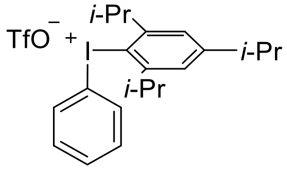

1m: according to ***GP1*** iodobenzene (10 mmol, 1.2 mL) reacted with 1,3,5-triisopropylbenzene (11.0 mmol, 2.7 mL) to give phenyl(2,4,6-triisopropylphenyl)iodonium triflate (**1m**) as a white solid, 3.00 g (54%). mp = 171 – 172 °C (lit. 169 – 170 °C).[15] The analytical data is in accordance with previously published.[15]
^{1}H NMR (400 MHz, $CDCl_3$) δ 7.67 (d, J = 8.0 Hz, 2H), 7.53 (t, J = 7.2 Hz, 1H), 7.42 (t, J = 7.6 Hz, 2H), 7.17 (s, 2H), 3.26 (sept., J = 6.8 Hz, 2H), 2.96 (sept., J = 6.8 Hz, 1H), 1.27 (d, J = 6.8 Hz, 6H), 1.23 (d, J = 6.8 Hz, 12H).
^{13}C NMR (100 MHz, $CDCl_3$) δ 155.8, 152.5, 132.7, 132.5, 132.0, 125.4, 120.7, 120.5 (q, J = 318.3 Hz), 113.0, 39.7, 34.3, 24.4, 23.8.
^{19}F NMR (376 MHz, $CDCl_3$) δ -78.3.

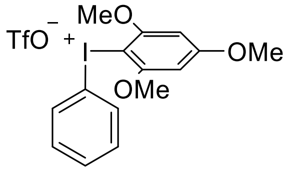

1n: according to ***GP3*** iodobenzene (5.0 mmol, 0.56 mL) reacted with 1,3,5-trimethoxybenzene (5.25 mmol, 0.88 g) to give phenyl(2,4,6-trimethoxyphenyl)iodonium triflate (**1n**) as a white solid, 2.00 g (77%). mp = 107 – 108 °C (dec.) (lit. 114 – 116 °C)[17]. The analytical data is in accordance with previously published.[17]
^{1}H NMR (400 MHz, DMSO-d_6) δ 7.92 (d, J = 8.0 Hz, 2H), 7.61 (t, J = 7.2 Hz, 1H), 7.47 (t, J = 7.6 Hz, 2H), 6.47 (s, 2H), 3.95 (s, 6H), 3.87 (s, 3H).
^{13}C NMR (100 MHz, DMSO-d_6) δ 166.2, 159.4, 134.3, 131.7, 131.6, 120.7 (q, J = 320.0 Hz), 116.1, 92.1, 87.0, 57.3, 56.1.
^{19}F NMR (376 MHz, DMSO-d_6) δ -77.8.

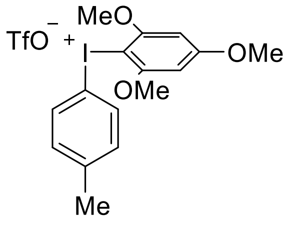

1o: according to ***GP3*** 1-iodo-4-methylbenzene (5.0 mmol, 1.09 g) reacted with 1,3,5-trimethoxybenzene (5.25 mmol, 0.88 g) to give 4-methylphenyl(2,4,6-

trimethoxyphenyl)iodonium tosylate as a white solid, 2.67 g (96%). 4-Methylphenyl(2,4,6-trimethoxyphenyl)iodonium tosylate (4.8 mmol ,2.66 g) was washed with the solution of NaOTf to give 4-methylphenyl(2,4,6-trimethoxyphenyl)iodonium triflate (**1o**) as a white solid, 2.20 g (85%). mp = 73 – 74 °C (dec.). The analytical data is in accordance with previously published.[18]

^{1}H NMR (400 MHz, DMSO-d_6) δ 7.80 (d, *J* = 8.4 Hz, 2H), 7.27 (d, *J* = 8.0 Hz, 2H), 6.45 (s, 2H), 3.94 (s, 6H), 3.86 (s, 3H), 2.32 (s, 3H).

^{13}C NMR (100 MHz, DMSO-d_6) δ 166.1, 159.3, 142.0, 134.5, 132.2, 120.7 (q, *J* = 320.3 Hz), 112.5, 92.0, 87.2, 57.3, 56.2, 20.8.

^{19}F NMR (376 MHz, DMSO-d_6) δ -77.8.

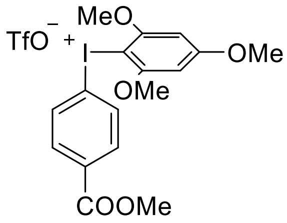

1p: according to ***GP3*** methyl 4-iodobenzoate (5.0 mmol, 1.31 g) reacted with 1,3,5-trimethoxybenzene (5.25 mmol, 0.88 g) to give (4-(methoxycarbonyl)phenyl)(2,4,6-trimethoxyphenyl)iodonium tosylate as a white solid, 2.64 g (88%). (4-(Methoxycarbonyl)phenyl)(2,4,6-trimethoxyphenyl)iodonium tosylate (4.4 mmol, 2.63 g) was washed with the solution of NaOTf to give (4-(methoxycarbonyl)phenyl)(2,4,6-trimethoxyphenyl)iodonium triflate (**1p**) as a white solid, 2.10 g (83%). mp = 98 – 100 °C (dec.) (lit. 120 – 122 °C).[19] The analytical data is in accordance with previously published.[19]

^{1}H NMR (400 MHz, DMSO-d_6) δ 8.05 (d, *J* = 8.0 Hz, 2H), 7.97 (d, *J* = 8.0 Hz, 2H), 6.48 (s, 2H), 3.94 (s, 6H), 3.87 (s, 3H), 3.85 (s, 3H).

^{13}C NMR (100 MHz, DMSO-d_6) δ 166.4, 165.2, 159.4, 134.6, 132.2, 131.9, 120.9, 120.7 (q., *J* = 320.3 Hz), 107.1, 92.2, 86.9, 57.4, 56.2, 55.0, 52.7, 15.2.

^{19}F NMR (376 MHz, DMSO-d_6) δ -77.8.

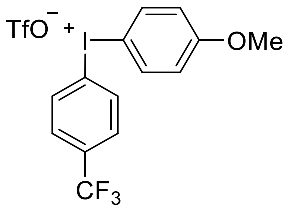

1q: according to ***GP1*** 4-iodobenzotrifluoride (5.0 mmol, 0.75 mL) reacted with anisole (5.0 mmol, 0.55 mL) to give (4-methoxyphenyl)(4-(trifluoromethyl)phenyl)iodonium triflate (**1q**) as an off-green solid, 0.76 g (29%). mp = 148 – 149 °C (lit. 149.6 °C).[20] The analytical data is in accordance with previously published.[20]

^1H NMR (400 MHz, DMSO-d_6) δ 8.39 (d, J = 8.0 Hz, 2H), 8.22 (d, J = 8.4 Hz, 2H), 7.91 (d, J = 8.4 Hz, 2H), 7.10 (d, J = 8.4 Hz, 2H), 3.80 (s, 3H).

^{13}C NMR (100 MHz, DMSO-d_6) δ 162.2, 137.6, 135.6, 131.7 (q., J = 31.3 Hz), 128.3 (q., J = 3.0 Hz), 123.5 (q., J = 271.7 Hz), 121.3, 120.7 (q., J = 320.0 Hz), 117.6, 55, 105.6, 55.8.

^{19}F NMR (376 MHz, DMSO-d_6) δ -61.6, -77.8.

4. Synthesis of amides 2

General procedure for 1 equiv of iodonium salt 1 (GP4): a transparent tube with a screw cap was charged with iodonium salt **1** (1.0 equiv, 0.2 mmol), Na_2CO_3 (1.7 equiv, 0.34 mmol, 36 mg), isonitrile (1.0 equiv, 0.2 mmol) and $[Ru(bpy)_3](PF_6)_2$ (2.0 mol %, 0.002 mmol, 2 mg). Afterwards MeCN (2 mL) and H_2O (200 µL) were added. The suspension was bubbled with argon for 20 min and closed under stream of argon. Then, the tube was allowed to stir under 20 W blue LED (465 nm) irradiation for 10 h under water cooling (14 °C). After the reaction was completed, water was added (15 mL) and the aqueous layer was extracted with DCM (4 × 15 mL). The combined organic layers were dried over Mg_2SO_4, filtered and the solvent was removed under reduced pressure. The product was isolated by column chromatography on silica to afford the crude product **2**. The crude product **2** was refluxed with hexane (1 mL), cooled to −30 °C, decanted and washed with hexane (2 × 1 mL). The solid was dried under vacuum to give amide **2**.

General procedure for 2 equiv of iodonium salt 1 (GP5): a transparent tube with a screw cap was charged with iodonium salt **1** (2.0 equiv, 0.4 mmol), Na_2CO_3 (3.4 equiv, 0.68 mmol, 72 mg), isonitrile (1.0 equiv, 0.2 mmol) and $[Ru(bpy)_3](PF_6)_2$ (2.0 mol %, 0.002 mmol, 2 mg). Afterwards, MeCN (2 mL) and H_2O (200 µL) were added. The suspension was bubbled with argon for 20 min and closed under stream of argon. Then the tube was allowed to stir under 20 W blue LED (465 nm) irradiation for 10 h under water cooling (14 °C). After the reaction was completed, water was added (15 mL) and the aqueous layer was extracted with DCM (4 × 15 mL). The combined organic layers were dried over Mg_2SO_4, filtered and the solvent was removed under reduced pressure. The product was isolated by column chromatography on silica to afford the crude product **2**. The crude product **2** was refluxed with hexane (1 mL), cooled to −30 °C, decanted and washed with hexane (2 × 1 mL). The solid was dried under vacuum to give amide **2**.

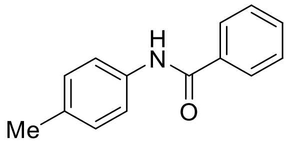

2aa: according to ***GP4*** 1-isocyano-4-methylbenzene (0.2 mmol, 24 mg) reacted with diphenyliodonium triflate (**1a**, 0.2 mmol, 86 mg) followed by column chromatography (gradient elution: hexane → hexane:EtOAc – 10:1) to give *N*-(4-tolyl)benzamide (**2aa**) as a beige solid, 13 mg (31%). mp = 155 – 157 °C (lit. 158 – 159 °C).[21] The analytical data is in accordance with previously published.[21]

^{1}H NMR (400 MHz, $CDCl_3$) δ 7.86 (d, *J* = 7.6 Hz, 3H(Ar+NH)), 7.55 – 7.51 (m, 3H), 7.46 (t, *J* = 7.6 Hz, 2H), 7.16 (d, *J* = 8.0 Hz, 2H), 2.34 (s, 3H).

^{13}C NMR (100 MHz, $CDCl_3$) δ 165.8, 135.5, 135.2, 134.4, 131.9, 129.7, 128.9, 127.1, 120.4, 21.1.

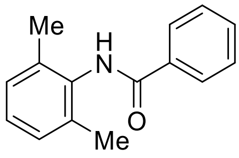

2ba: according to ***GP4*** 2-isocyano-1,3-dimethylbenzene (0.2 mmol, 26 mg) reacted with diphenyliodonium triflate (**1a**, 0.2 mmol, 86 mg) followed by column chromatography (gradient elution: hexane:EtOAc – 10:1 → 7:1) to give *N*-(2,6-dimethylphenyl)benzamide (**2ba**) as an yellowish solid, 15 mg (33%). mp = 156 – 157 °C (lit. 155 – 157 °C).[22] The analytical data is in accordance with previously published.[23]

^{1}H NMR (400 MHz, $CDCl_3$) δ 7.91 (d, *J* = 7.6 Hz, 2H), 7.56 (t, *J* = 7.2 Hz, 1H), 7.50 – 7.47 (m, 3H), 7.17 – 7.10 (m, 3H), 2.27 (s, 6H).

^{13}C NMR (100 MHz, $CDCl_3$) δ 166.0, 135.7, 134.6, 134.0, 131.9, 128.9, 128.4, 127.5, 127.4, 18.6.

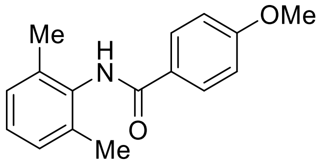

2bb: according to ***GP5*** 2-isocyano-1,3-dimethylbenzene (0.2 mmol, 26 mg) reacted with bis(4-methoxyphenyl)iodonium triflate (**1b**, 0.4 mmol, 196 mg) followed by column chromatography (gradient elution: hexane:EtOAc – 10:1 → 7:1) to give *N*-(2,6-dimethylphenyl)-4-methoxybenzamide (**2bb**) as a beige solid, 14 mg (27%). mp = 169 – 170 °C (lit. 168 – 170 °C).[24] The analytical data is in accordance with previously published.[24]

^{1}H NMR (400 MHz, $CDCl_3$) δ 7.89 (d, *J* = 8.0 Hz, 2H), 7.36 (br s, 1H), 7.16 – 7.09 (m, 3H), 6.97 (d, *J* = 8.0 Hz, 2H), 3.88 (s, 3H), 2.27 (s, 6H).

^{13}C NMR (100 MHz, $CDCl_3$) δ 165.5, 162.6, 135.7, 134.2, 129.2, 128.4, 127.4, 126.8, 114.0, 55.6, 18.6.

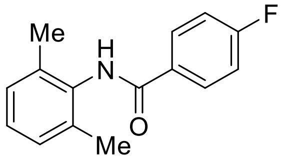

2bc: according to ***GP4*** 2-isocyano-1,3-dimethylbenzene (0.2 mmol, 26 mg) reacted with bis(4-fluorophenyl)iodonium triflate (**1c**, 0.2 mmol, 93 mg) followed by column chromatography (gradient elution: hexane:EtOAc – 10:1 → 7:1) to give *N*-(2,6-dimethylphenyl)-4-fluorobenzamide (**2bc**) as a pinkish solid, 21 mg (43%). mp = 180 – 181 °C (lit. 179 – 181 °C).[24] The analytical data is in accordance with previously published.[24]

^{1}H NMR (400 MHz, $CDCl_3$) δ 7.90 (dd, *J* = 8.0, 5.6 Hz, 2H), 7.47 (br s, 1H), 7.17 – 7.10 (m, 5H), 2.25 (s, 6H).
^{13}C NMR (100 MHz, $CDCl_3$) δ 165.0 (d, *J* = 251.0 Hz), 165.0, 135.7, 133.9, 130.7 (d, *J* = 3.0 Hz), 129.7 (d, *J* = 9.0 Hz), 128.4, 127.7, 115.9 (d, *J* = 21.0 Hz), 18.6.
^{19}F NMR (376 MHz, $CDCl_3$) δ -107.6 – -107.7 (m).
HRMS (positive mode, MeCN) calcd. for $C_{15}H_{15}FNO^+$ ($[M+H]^+$) 244.1133 (found 244.1135).

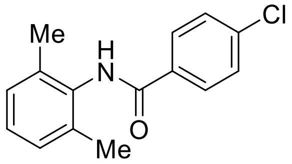

2bd: according to ***GP4*** 2-isocyano-1,3-dimethylbenzene (0.2 mmol, 26 mg) reacted with bis(4-chlorophenyl)iodonium triflate (**1d**, 0.2 mmol, 100 mg) followed by column chromatography (gradient elution: hexane:EtOAc – 10:1 → 7:1) to give 4-chloro-*N*-(2,6-dimethylphenyl)benzamide (**2bd**) as an yellowish solid, 22 mg (42%). mp = 180 – 181 °C.
^{1}H NMR (400 MHz, $CDCl_3$) δ 7.83 (d, *J* = 8.0 Hz, 2H), 7.50 (br s, 1H), 7.43 (d, *J* = 8.0 Hz, 2H), 7.17 – 7.10 (m, 3H), 2.24 (s, 6H).
^{13}C NMR (100 MHz, $CDCl_3$) δ 165.1, 138.2, 135.6, 133.8, 132.9, 129.1, 128.8, 128.5, 127.7, 18.6.
HRMS (positive mode, MeCN) calcd. for $C_{15}H_{15}ClNO^+$ ($[M+H]^+$) 260.0837 (found 260.0834).

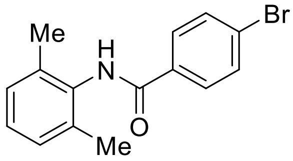

2be: according to ***GP4*** 2-isocyano-1,3-dimethylbenzene (0.2 mmol, 26 mg) reacted with bis(4-bromophenyl)iodonium triflate (**1e**, 0.2 mmol, 118 mg) followed by column chromatography (gradient elution: hexane:EtOAc – 10:1 → 7:1) to give 4-bromo-*N*-(2,6-dimethylphenyl)benzamide (**2be**) as a white solid, 33 mg (54%). mp = 189 – 191 °C (lit. 190 – 192 °C).[24] The analytical data is in accordance with previously published.[24]
^{1}H NMR (400 MHz, $CDCl_3$) δ 7.74 (d, *J* = 8.4 Hz, 2H), 7.59 – 7.56 (m, 3H), 7.17 – 7.09 (m, 3H), 2.23 (s, 6H).
^{13}C NMR (100 MHz, $CDCl_3$) δ 165.2, 135.6, 133.8, 133.3, 132.1, 129.0, 128.4, 127.7, 126.6, 18.6.

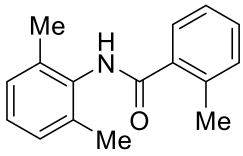

2bf: according to ***GP4*** 2-isocyano-1,3-dimethylbenzene (0.2 mmol, 26 mg) reacted with bis(2-methylphenyl)iodonium triflate (**1f**, 0.2 mmol, 92 mg) followed by column chromatography (gradient elution: hexane:EtOAc – 10:1 → 7:1) to give *N*-(2,6-dimethylphenyl)-2-methylbenzamide (**2bf**) as an yellowish solid, 17 mg (36%). mp = 135 – 136 °C (lit. 138 – 140 °C).[24] The analytical data is in accordance with previously published.[24]

^{1}H NMR (400 MHz, $CDCl_3$) δ 7.56 (d, *J* = 7.6 Hz, 1H), 7.38 (t, *J* = 7.2 Hz 1H), 7.30 – 7.25 (m, 2H), 7.18 – 7.11 (m, 3H), 7.08 (br s, 1H), 2.54 (s, 3H), 2.34 (s, 6H).

^{13}C NMR (100 MHz, $CDCl_3$) δ 168.6, 136.7, 136.4, 135.7, 133.7, 131.4, 130.3, 128.5, 127.7, 126.8, 126.0, 20.1, 18.8.

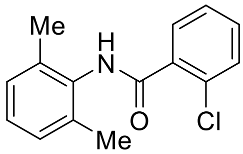

2bg: according to ***GP4*** 2-isocyano-1,3-dimethylbenzene (0.2 mmol, 26 mg) reacted with bis(2- chlorophenyl)iodonium triflate (**1g**, 0.2 mmol, 100 mg) followed by column chromatography (gradient elution: hexane:EtOAc – 10:1 → 7:1) to give 2-chloro-*N*-(2,6-dimethylphenyl)benzamide (**2bg**) as a white solid, 35 mg (67%). mp = 148 – 150 °C. The analytical data is in accordance with previously published.[25]

^{1}H NMR (400 MHz, $CDCl_3$) δ 7.79 (d, *J* = 7.2 Hz, 1H), 7.49 – 7.36 (m, 4H), 7.17 – 7.11 (m, 3H), 2.35 (s, 6H).

^{13}C NMR (100 MHz, $CDCl_3$) δ 165.0, 135.8, 135.3, 133.5, 131.7, 130.7, 130.6, 130.5, 128.5, 127.8, 127.4, 18.9.

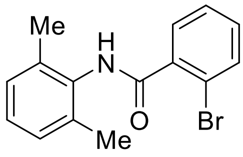

2bh: according to ***GP4*** 2-isocyano-1,3-dimethylbenzene (0.2 mmol, 26 mg) reacted with bis(2-bromophenyl)iodonium triflate (**1h**, 0.2 mmol, 118 mg) followed by column chromatography (gradient elution: hexane:EtOAc – 10:1 → 7:1) to give 2-bromo-*N*-(2,6-dimethylphenyl) benzamide (**2bh**) as a white solid, 39 mg (64%). mp = 172 – 173 °C (lit. 166 – 168 °C).[26] The analytical data is in accordance with previously published.[26]

^{1}H NMR (400 MHz, $CDCl_3$) δ 7.67 (t, *J* = 8.4 Hz, 2H), 7.42 (t, *J* = 7.6 Hz, 1H), 7.35 – 7.31 (m, 2H), 7.18 – 7.11 (m, 3H), 2.37 (s, 6H).

^{13}C NMR (100 MHz, $CDCl_3$) δ 166.0, 138.0, 135.9, 133.7, 133.4, 131.6, 130.0, 128.5, 127.9, 127.8, 119.4, 19.0.

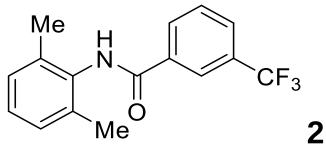

2bi: according to ***GP4*** 2-isocyano-1,3-dimethylbenzene (0.2 mmol, 26 mg) reacted with bis(3-(trifluoromethyl)phenyl)iodonium triflate (**1i**, 0.2 mmol, 113 mg) followed by column chromatography (gradient elution: hexane → hexane:EtOAc – 10:1) to give *N*-(2,6-dimethylphenyl)-3-(trifluoromethyl)benzamide (**2bi**) as an yellowish solid, 32 mg (55%). mp = 234 – 236 °C.

^{1}H NMR (400 MHz, $CDCl_3$) δ 8.17 (s, 1H), 8.07 (d, *J* = 7.6 Hz, 1H), 7.82 (d, *J* = 8.0 Hz, 1H), 7.63 – 7.58 (m, 2H), 7.18 – 7.10 (m, 3H), 2.25 (s, 6H).

^{13}C NMR (100 MHz, $CDCl_3$) δ 164.7, 135.6, 135.4, 133.5, 131.5 (q, *J* = 32.6 Hz), 130.6, 129.5, 128.6 (q, *J* = 4.0 Hz), 128.5, 127.9, 124.4 (q, *J* = 3.7 Hz), 123.8 (q, *J* = 271.0 Hz), 18.6.

^{19}F NMR (376 MHz, $CDCl_3$) δ -62.7.

HRMS (positive mode, MeCN) calcd. for $C_{16}H_{15}F_3NO^+$ ($[M+H]^+$) 294.1100 (found 294.1105).

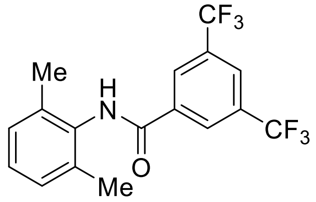

2bj: according to ***GP5*** 2-isocyano-1,3-dimethylbenzene (0.2 mmol, 26 mg) reacted with bis(3,5-bis(trifluoromethyl)phenyl)iodonium triflate (**1j**, 0.4 mmol, 281 mg) followed by column chromatography (gradient elution: hexane → hexane:EtOAc – 10:1) to give *N*-(2,6-dimethylphenyl)-3,5-bis(trifluoromethyl)benzamide (**2bj**) as a white solid, 30 mg (42%). mp = 176 – 177 °C.

^{1}H NMR (400 MHz, $CDCl_3$) δ 8.36 (s, 2H), 8.07 (s, 1H), 7.59 (br s, 1H), 7.19 – 7.11 (m, 3H), 2.26 (s, 6H).

^{13}C NMR (100 MHz, $CDCl_3$) δ 163.2, 136.6, 135.5, 133.1, 132.8 (q, *J* = 33.6 Hz), 128.6, 128.2, 127.7 (d, *J* = 2.0 Hz), 125.5 (p, *J* = 3.5 Hz), 123.0 (q, *J* = 271.3 Hz), 18.6.

^{19}F NMR (376 MHz, $CDCl_3$) δ -62.9.

HRMS (positive mode, MeCN) calcd. for $C_{17}H_{14}F_6NO^+$ ($[M+H]^+$) 360.0975 (found 360.0983).

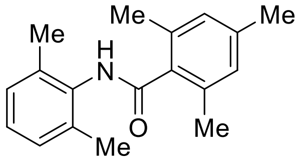

2bk: according to ***GP4*** 2-isocyano-1,3-dimethylbenzene (0.2 mmol, 26 mg) reacted with dimesityliodonium triflate (**1k**, 0.2 mmol, 103 mg) followed by column chromatography (gradient elution: hexane:EtOAc – 10:1 → 7:1) to give *N*-(2,6-dimethylphenyl)-2,4,6-trimethylbenzamide (**2bk**) as a beige solid, 10 mg (19%). mp = 192 – 193 °C (lit. 197 – 199 °C).[27] The analytical data is in accordance with previously published.[27]

^{1}H NMR (400 MHz, $CDCl_3$) δ 7.17 – 7.11 (m, 3H), 6.96 (br s, 1H), 6.91 (s, 2H), 2.47 (s, 6H), 2.39 (s, 6H), 2.31 (s, 3H).

^{13}C NMR (100 MHz, $CDCl_3$) δ 168.8, 138.9, 135.3, 135.0, 134.7, 133.6, 128.8, 128.7, 127.6, 21.2, 20.1, 19.7.

^{1}H NMR (400 MHz, DMSO-*d6*) δ 9.67 (s, 1H), 7.10 (s, 3H), 6.92 (s, 2H), 2.37 (s, 6H), 2.29 (s, 6H), 2.26 (s, 3H).

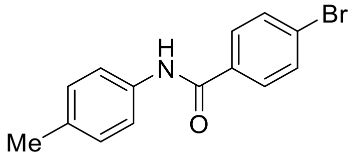

2ce: according to ***GP4*** 1-isocyano-4-methylbenzene (0.2 mmol, 24 mg) reacted with bis(4-bromophenyl)iodonium triflate (**1e**, 0.2 mmol, 118 mg) followed by column chromatography (gradient elution: hexane:EtOAc – 10:1 → 7:1) to give 4-bromo-*N*-(*p*-tolyl)benzamide (**2ce**) as a brown solid, 13 mg (22%). mp = 186 – 187 °C (lit. 180 – 181 °C).[28] The analytical data is in accordance with previously published.[28]

^{1}H NMR (400 MHz, DMSO-d_6) δ 10.23 (s, 1H), 7.90 (d, J = 8.0 Hz, 2H), 7.74 (d, J = 8.0 Hz, 2H), 7.64 (d, J = 8.0 Hz, 2H), 7.15 (d, J = 8.0 Hz, 2H), 2.28 (s, 3H).

^{13}C NMR (100 MHz, DMSO-d_6) δ 164.3, 136.4, 134.1, 132.8, 131.4, 129.8, 129.1, 125.2, 120.4, 20.5.

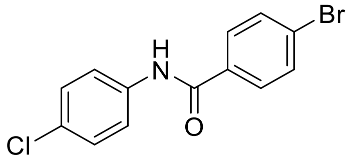

2de: according to ***GP5*** 1-chloro-4-isocyanobenzene (0.2 mmol, 27 mg) reacted with bis(4-bromophenyl)iodonium triflate (**1e**, 0.4 mmol, 235 mg) followed by column chromatography (gradient elution: hexane:EtOAc – 10:1 → 7:1) to give 4-bromo-*N*-(4-chlorophenyl)benzamide (**2de**) as an off-white solid, 20 mg (32%). mp = 219 – 220 °C. The analytical data is in accordance with previously published.[28]

^{1}H NMR (400 MHz, $CDCl_3$) δ 7.80 (br s, 1H), 7.73 (d, J = 8.0 Hz, 2H), 7.63 (d, J = 8.0 Hz, 2H), 7.58 (d, J = 8.4 Hz, 2H), 7.34 (d, J = 8.4 Hz, 2H).

^{13}C NMR (100 MHz, $CDCl_3$) δ 164.9, 136.3, 133.5, 132.3, 130.0, 129.3, 128.8, 127.0, 121.6.

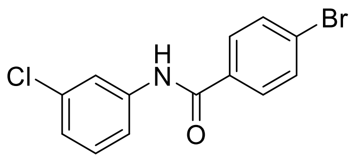

2ee: according to ***GP5*** 1-isocyano-3-chlorobenzene (0.2 mmol, 27 mg) reacted with bis(4-bromophenyl)iodonium triflate (**1e**, 0.4 mmol, 235 mg) followed by column chromatography (hexane:EtOAc – 15:1) to give 4-bromo-*N*-(3-chlorophenyl)benzamide (**2ee**) as a white solid, 8 mg (13%). mp = 120 – 121 °C.

^{1}H NMR (400 MHz, $CDCl_3$) δ 7.80 (br s, 1H), 7.75 – 7.72 (m, 3H), 7.63 (d, J = 8.0 Hz, 2H), 7.47 (d, J = 8.0 Hz, 1H), 7.29 (t, J = 8.0 Hz, 1H), 7.14 (d, J = 8.0 Hz, 1H).

^{13}C NMR (100 MHz, $CDCl_3$) δ 164.8, 138.9, 135.0, 133.5, 132.3, 130.3, 128.8, 127.1, 125.0, 120.5, 118.3.

HRMS (positive mode, MeCN) calcd. for $C_{13}H_9BrClNNaO^+$ ($[M+H]^+$) 331.9454 (found 331.9444).

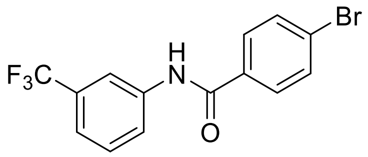

2fe: according to ***GP5*** 1-isocyano-3-(trifluoromethyl)benzene (0.2 mmol, 34 mg) reacted with bis(4-bromophenyl)iodonium triflate (**1e**, 0.2 mmol, 118 mg) followed by column chromatography (hexane:EtOAc – 15:1) to give 4-bromo-*N*-(3-(trifluoromethyl)phenyl)benzamide (**2fe**) as a white solid, 5 mg (7%). mp = 98 – 100 °C.

^{1}H NMR (400 MHz, $CDCl_3$) δ 7.92 (s, 1H), 7.85 (d, J = 6.4 Hz, 2H(Ar+NH)), 7.76 (d, J = 8.4 Hz, 2H), 7.65 (d, J = 8.4 Hz, 2H), 7.51 (t, J = 8.0 Hz, 1H), 7.43 (d, J = 7.6 Hz, 1H).

^{13}C NMR (125 MHz, $CDCl_3$) δ 165.0, 138.3, 133.3, 132.4, 131.7 (q, J = 32.5 Hz), 129.9, 128.8, 127.2, 123.4, 122.8 (q, J = 271.5 Hz), 121.5 (q, J = 3.8 Hz), 117.1 (q, J = 3.8 Hz).

^{19}F NMR (376 MHz, $CDCl_3$) δ -62.8.

HRMS (positive mode, MeCN) calcd. for $C_{14}H_9BrF_3NNaO^+$ ($[M+H]^+$) 365.9717 (found 365.9715).

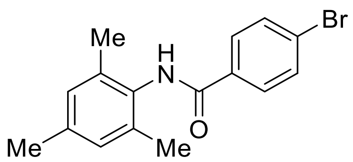

2ge: according to ***GP4*** 2-isocyano-1,3,5-trimethylbenzene (0.2 mmol, 29 mg) reacted with bis(4-bromophenyl)iodonium triflate (**1e**, 0.4 mmol, 235 mg) followed by column chromatography (gradient elution: hexane:EtOAc – 10:1 →

7:1) to give 4-bromo-*N*-mesitylbenzamide (**2ge**) as a white solid, 46 mg (72%). mp = 222 – 223 °C.

^{1}H NMR (400 MHz, $CDCl_3$) δ 7.76 (d, *J* = 8.0 Hz, 2H), 7.60 (d, *J* = 8.0 Hz, 2H), 7.37 (br s, 1H), 6.93 (s, 2H), 2.30 (s, 3H), 2.21 (s, 6H).

^{13}C NMR (100 MHz, $CDCl_3$) δ 165.3, 137.4, 135.4, 133.5, 132.1, 131.1, 129.2, 129.0, 126.6, 21.1, 18.5.

HRMS (positive mode, MeCN) calcd. for $C_{16}H_{17}BrNO^+$ ($[M+H]^+$) 318.0489 (found 244.1135).

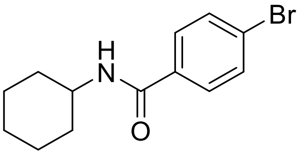

2he: according to ***GP4*** isocyanocyclohexane (0.2 mmol, 22 mg) reacted with bis(4-bromophenyl)iodonium triflate (**1e**, 0.4 mmol, 235 mg) followed by column chromatography (gradient elution: hexane → hexane:EtOAc – 10:1) to give 4-bromo-*N*-cyclohexylbenzamide (**2he**) as an off-white solid, 27 mg (48%). mp = 200 – 201 °C (lit. 180 – 181 °C).[29] The analytical data is in accordance with previously published.[29]

^{1}H NMR (400 MHz, $CDCl_3$) δ 7.61 (d, *J* = 8.4 Hz, 2H), 7.55 (d, *J* = 8.4 Hz, 2H), 5.96 (d, *J* = 4.4 Hz, 1H), 4.00 – 3.90 (m, 1H), 2.02 (d, *J* = 12.0 Hz, 2H), 1.78 – 1.73 (m, 2H), 1.66 (s, 1H), 1.42 (q, *J* = 12.4 Hz, 2H), 1.27 – 1.18 (m, 3H).

^{13}C NMR (100 MHz, $CDCl_3$) δ 165.8, 134.0, 131.8, 128.6, 126.0, 49.0, 33.3, 25.7, 25.0.

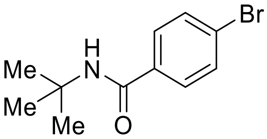

2ie*:* according to ***GP4*** 2-isocyano-2-methylpropane (0.2 mmol, 17 mg) reacted with bis(4-bromophenyl)iodonium triflate (**1e**, 0.2 mmol, 118 mg) followed by column chromatography (hexane:EtOAc – 15:1) to give 4-bromo-*N*-(*tert*-butyl)benzamide (**2ie**) as a white solid, 13 mg (25%). mp = 135 – 136 °C (lit. 132 – 133 °C).[30] The analytical data is in accordance with previously published.[30]

^{1}H NMR (400 MHz, $CDCl_3$) δ 7.58 (d, *J* = 8.4 Hz, 2H), 7.54 (d, *J* = 8.8 Hz, 2H), 5.88 (br s, 1H), 1.46 (s, 9H).

^{13}C NMR (100 MHz, $CDCl_3$) δ 166.1, 134.9, 131.8, 128.5, 125.8, 52.0, 29.0.

HRMS (positive mode, MeCN) calcd. for $C_{11}H_{15}BrNNaO^+$ ($[M+H]^+$) 278.0156 (found 278.0151).

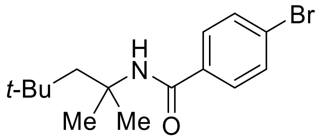

2je*:* according to ***GP4*** 2-isocyano-2,4,4-trimethylpentane (0.2 mmol, 28 mg) reacted with bis(4-bromophenyl)iodonium triflate (**1e**, 0.2 mmol, 118 mg) followed by column chromatography (hexane:EtOAc – 15:1) to give 4-bromo-*N*-(2,4,4-trimethylpentan-2-yl)benzamide (**2je**) as a white solid, 6 mg (10%). mp = 85 – 87 °C.

^{1}H NMR (400 MHz, $CDCl_3$) δ 7.60 – 7.52 (m, 4H), 5.88 (s, 1H), 1.85 (s, 2H), 1.52 (s, 6H), 1.04 (s, 9H).

^{13}C NMR (125 MHz, $CDCl_3$) δ 165.8, 135.2, 131.9, 128.4, 125.8, 55.9, 51.8, 31.9, 31.7, 29.4.

HRMS (positive mode, MeCN) calcd. for $C_{15}H_{22}BrNNaO^+$ ($[M+H]^+$) 334.0774 (found 334.0782).

Supplementary note 2

Some of the benzamides **2** in the reactions in Scheme 3 (main article) could not be isolated by column chromatography as individual compounds. Thus, for amides **2ba** (isolated with symmetrical iodonium salt), **2bk** (isolated with symmetrical iodonium salt), **2bm** and **2bb** yields were determined via ^{1}H NMR with internal standards for a mixture of amides or a mixture of a single amide with unidentified impurities after column chromatography.

Here we also provide a crude ^{1}H NMR spectrum for a reaction of 2-isocyano-1,3-dimethylbenzene with iodonium salt **1l** to make sure that we obtained reliable data after isolation. In order to ensure correct identification of the amides **2ba** and **2bk** we recorded ^{1}H NMR in DMSO-d_6 for amide **2bk** and compared it with a literature spectrum of amide **2ba**.[31]

Phenyl(mesityl)iodonium triflate (1l)

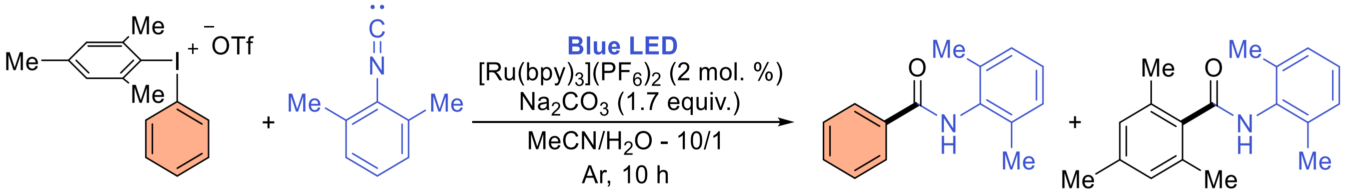

1l

Crude ^{1}H NMR:	**2ba**, 22 %	**2bk,** 8 %
^{1}H NMR after isolation of the **2ba**/**2bk** mixture:	**2ba**, 23 %	**2bk,** 6 %

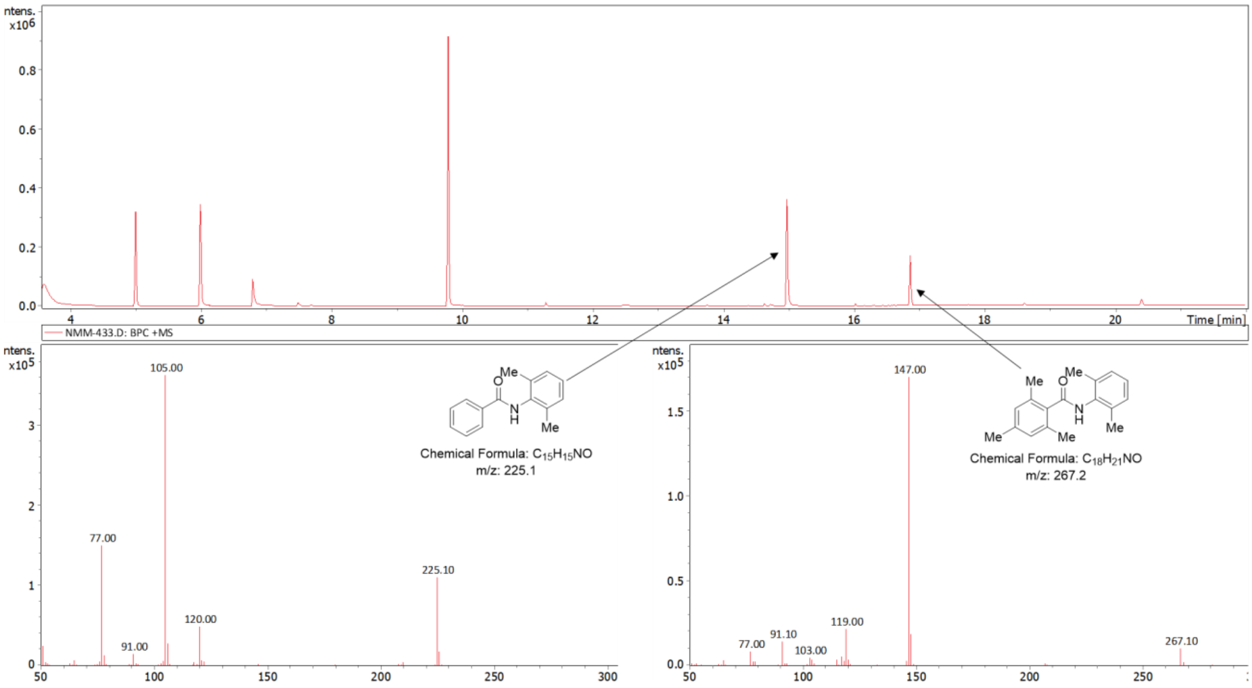

Figure S8. GC–MS data from the reaction mixture with iodonium salt **1l**

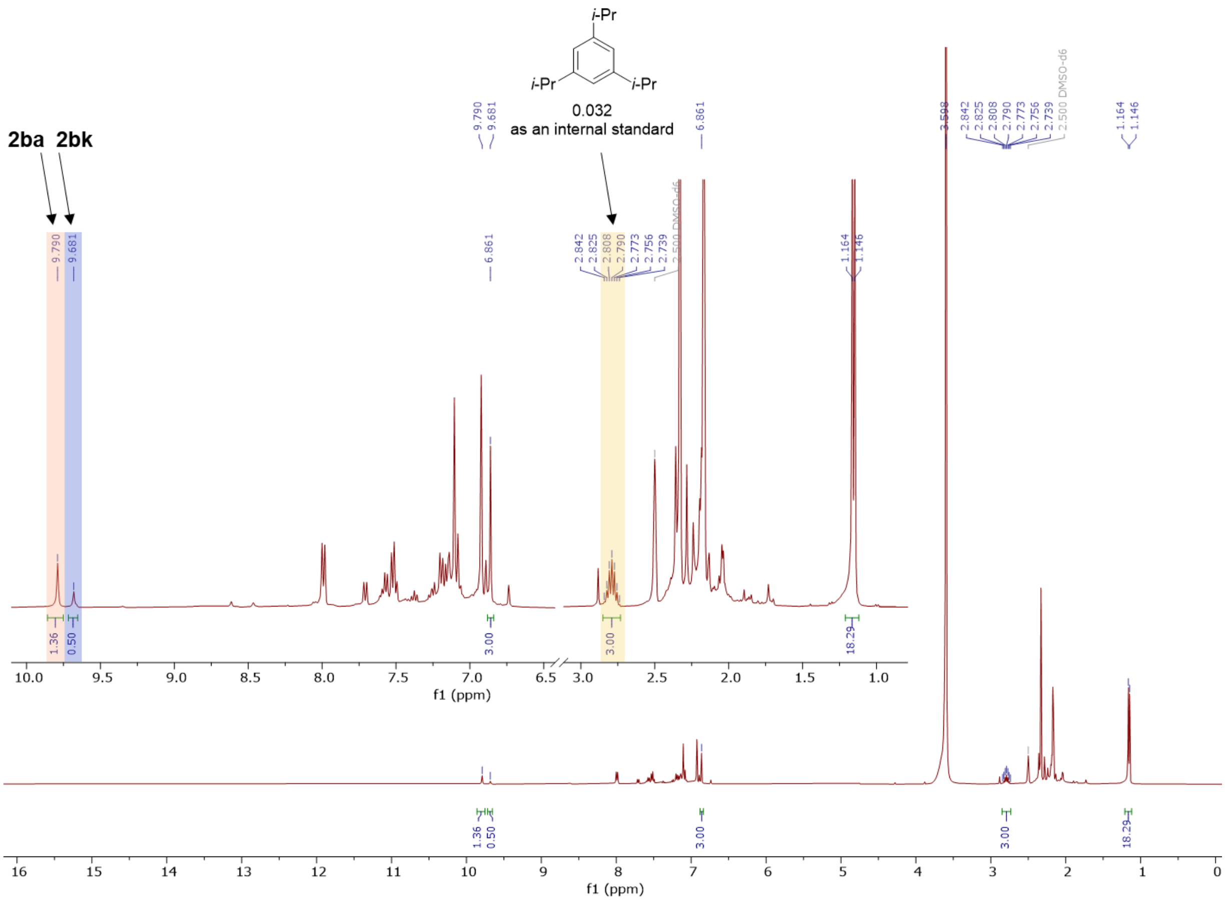

Figure S9. Crude ^{1}H NMR after the reaction with iodomium salt **1l**

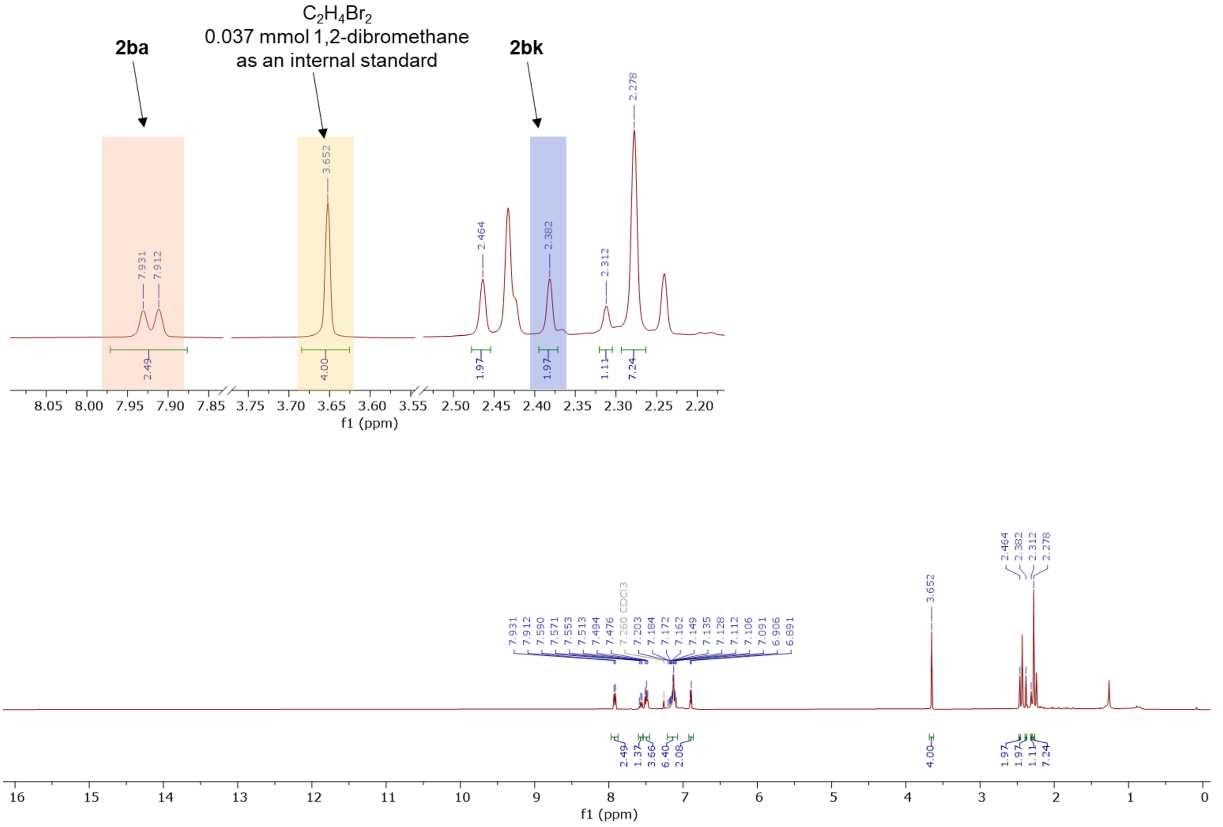

Figure S10. Mixture of amides **2ba** and **2bk** after isolation with column chromatography

Phenyl(2,4,6-triisopropylphenyl)iodonium triflate (1m)

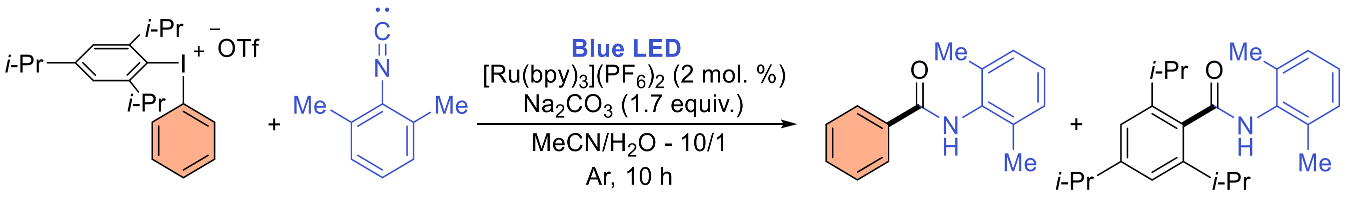

1m **2ba**, 8 % **2bm**, <2 %

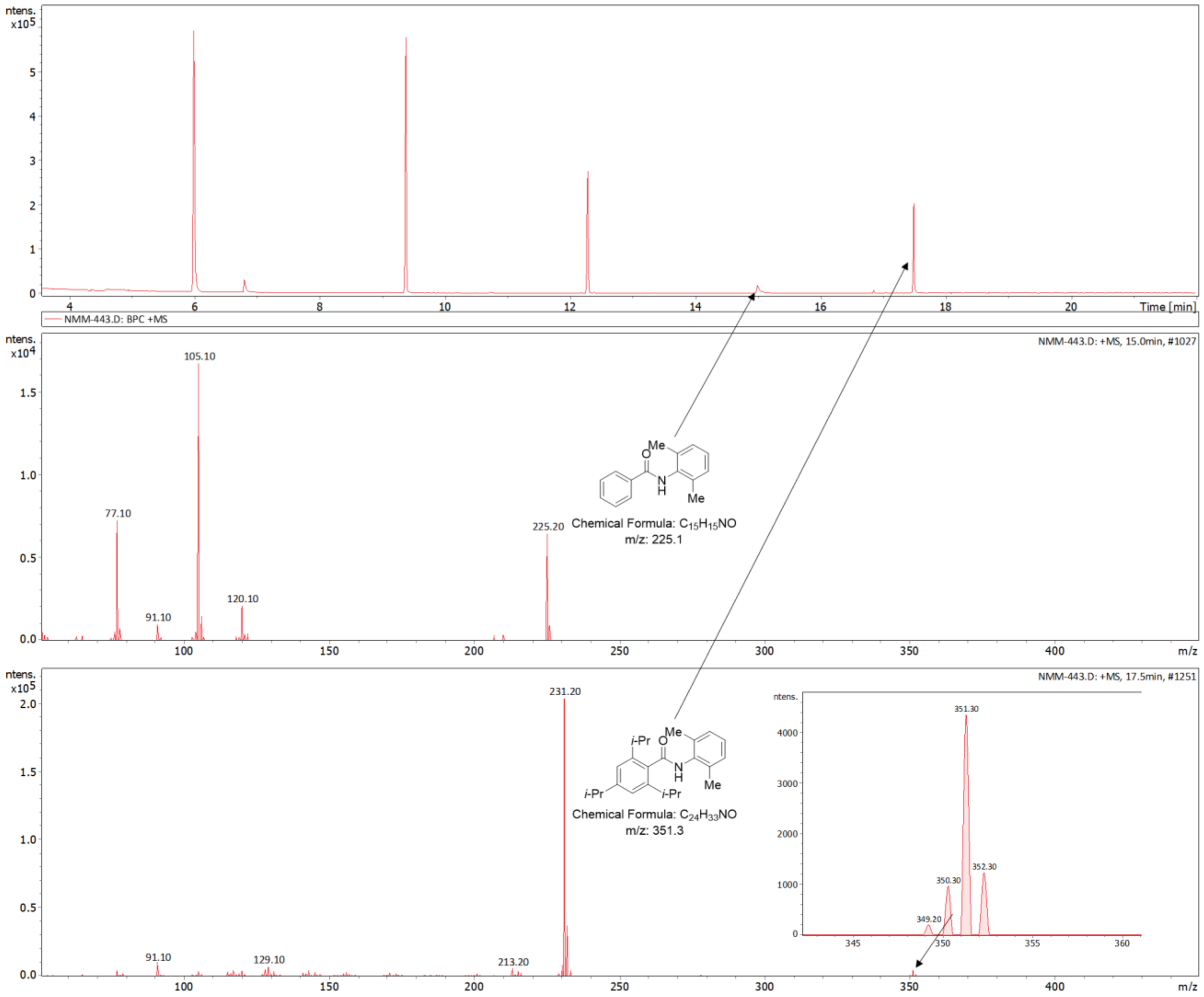

Figure S11. GC–MS data from the reaction mixture with iodonium salt **1m**

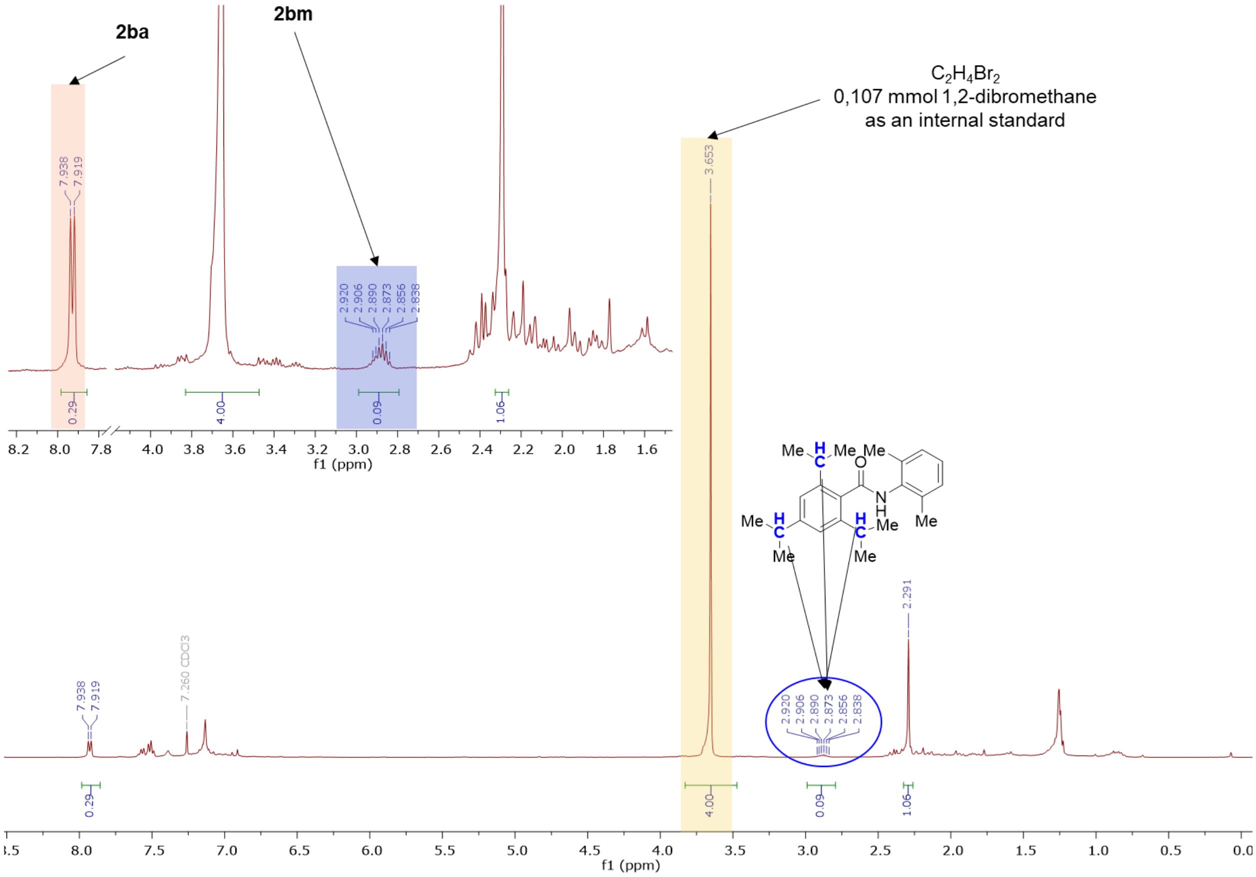

Figure S12. Mixture of amides **2ba** and **2bm** after isolation with column chromatography

Phenyl(2,4,6-trimethoxyphenyl)iodonium triflate (1n)

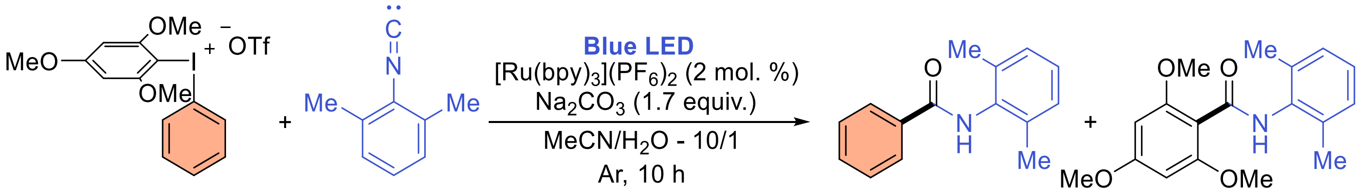

1n **2ba**, 25 % **2bn**, 0 %

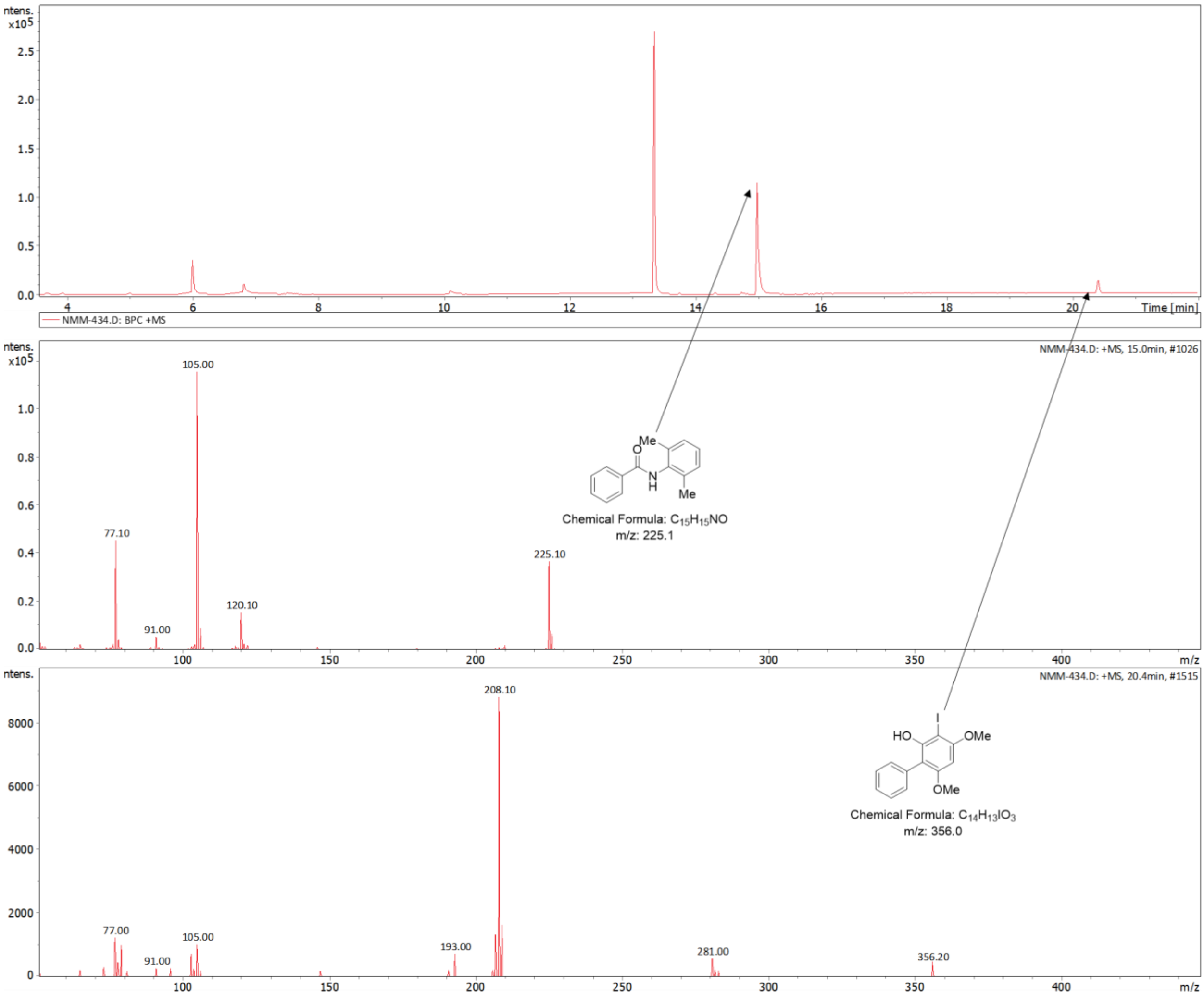

Figure S13. GC–MS data from the reaction mixture with iodonium salt **1n**

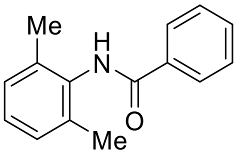

2ba: according to ***GP4*** 2-isocyano-1,3-dimethylbenzene (0.2 mmol, 26 mg) reacted with phenyl(2,4,6-trimethoxyphenyl)iodonium triflate (**1n**, 0.2 mmol, 104 mg) followed by column chromatography (gradient elution: hexane:EtOAc – 10:1 → 7:1) to give *N*-(2,6-dimethylphenyl)benzamide (**2ba**) as a yellowish solid, 11 mg (25%). The analytical data is in accordance with amide **2ba** synthesized from symmetrical iodonium salt **1a**.

4-Methylphenyl(2,4,6-trimethoxyphenyl)iodonium triflate (1o)

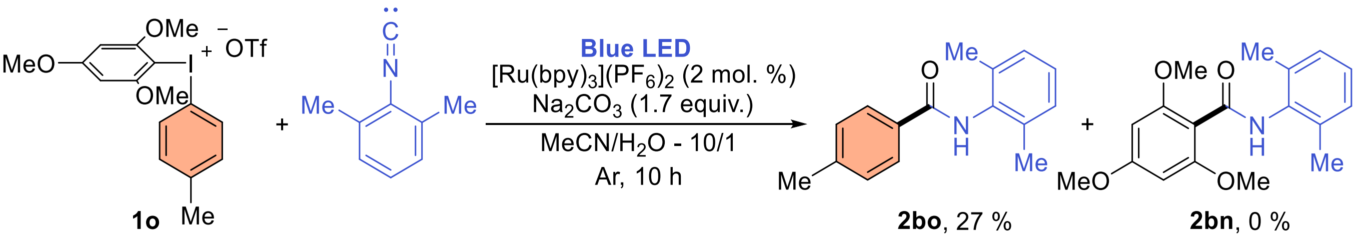

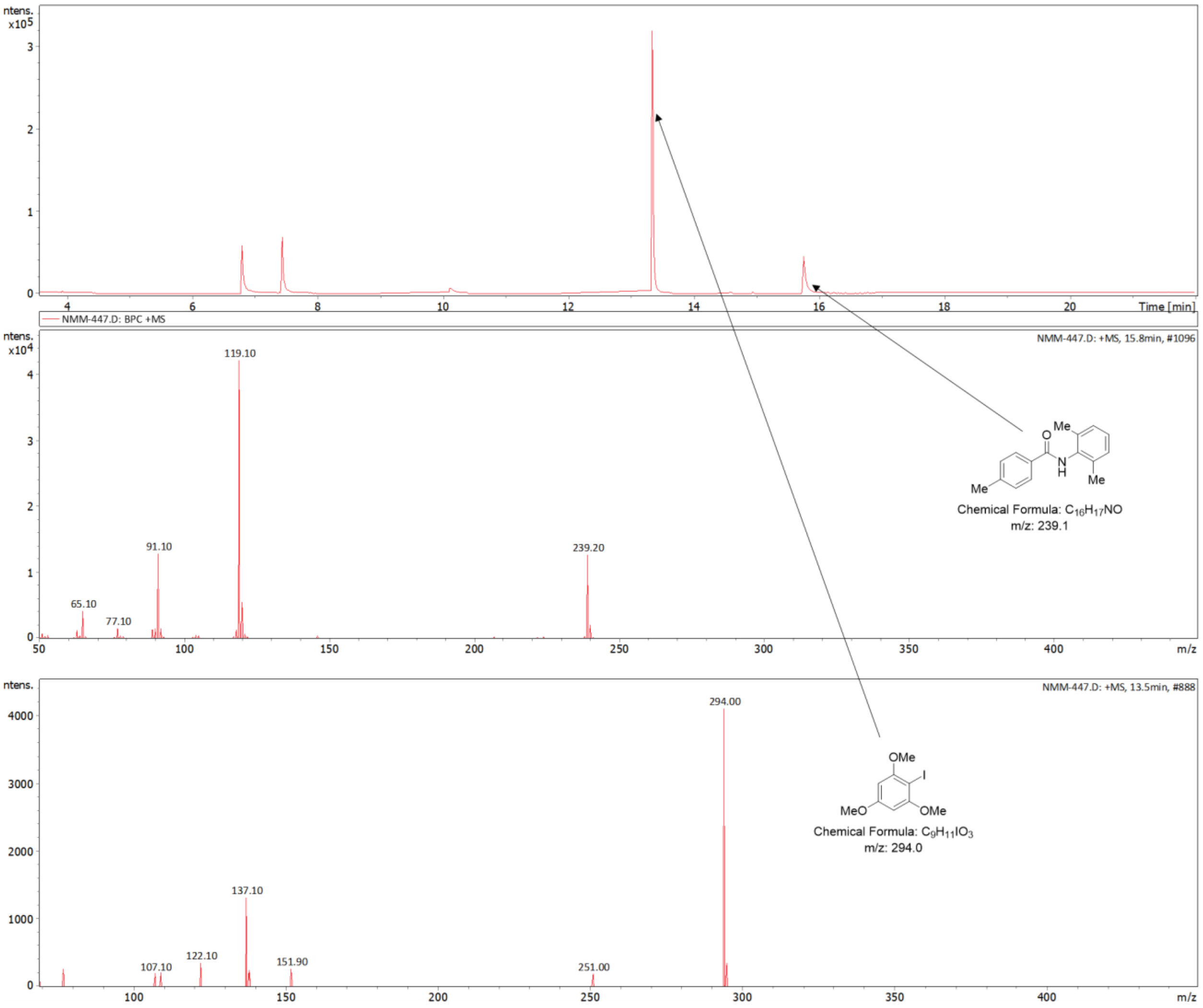

Figure S14. GC–MS data for the reaction mixture with iodonium salt **1o**

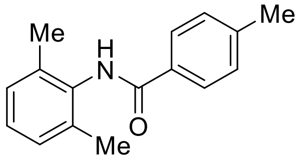

2bo: according to ***GP4*** 2-isocyano-1,3-dimethylbenzene (0.2 mmol, 26 mg) reacted with 4-methylphenyl(2,4,6-trimethoxyphenyl)iodonium triflate (**1o**, 0.2 mmol, 107 mg) followed by column chromatography (gradient elution: hexane → hexane:EtOAc – 10:1 → 7/1) to give *N*-(2,6-dimethylphenyl)-4-methylbenzamide (**2bo**) as an off-white solid, 13 mg (27%). mp = 158 – 159 °C (lit. 162 – 164 °C).[24] The analytical data is in accordance with previously published.[32]

^{1}H NMR (400 MHz, CDCl$_3$) δ 7.83 (d, J = 7.6 Hz, 2H), 7.35 (br s, 1H), 7.30 (d, J = 8.0 Hz, 2H), 7.18 – 7.10 (m, 3H), 2.44 (s, 3H), 2.28 (s, 6H).

^{13}C NMR (100 MHz, CDCl$_3$) δ 165.9, 142.5, 135.7, 134.1, 131.8, 129.6, 128.4, 127.5, 127.4, 21.7, 18.7.

(4-(Methoxycarbonyl)phenyl)(2,4,6-trimethoxyphenyl)iodonium triflate (1p)

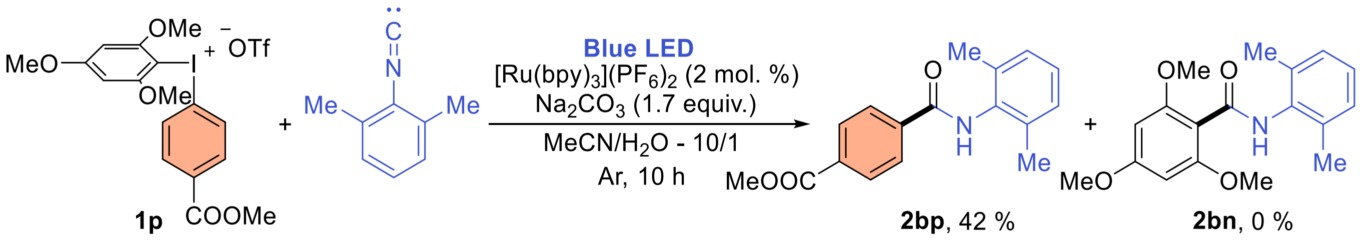

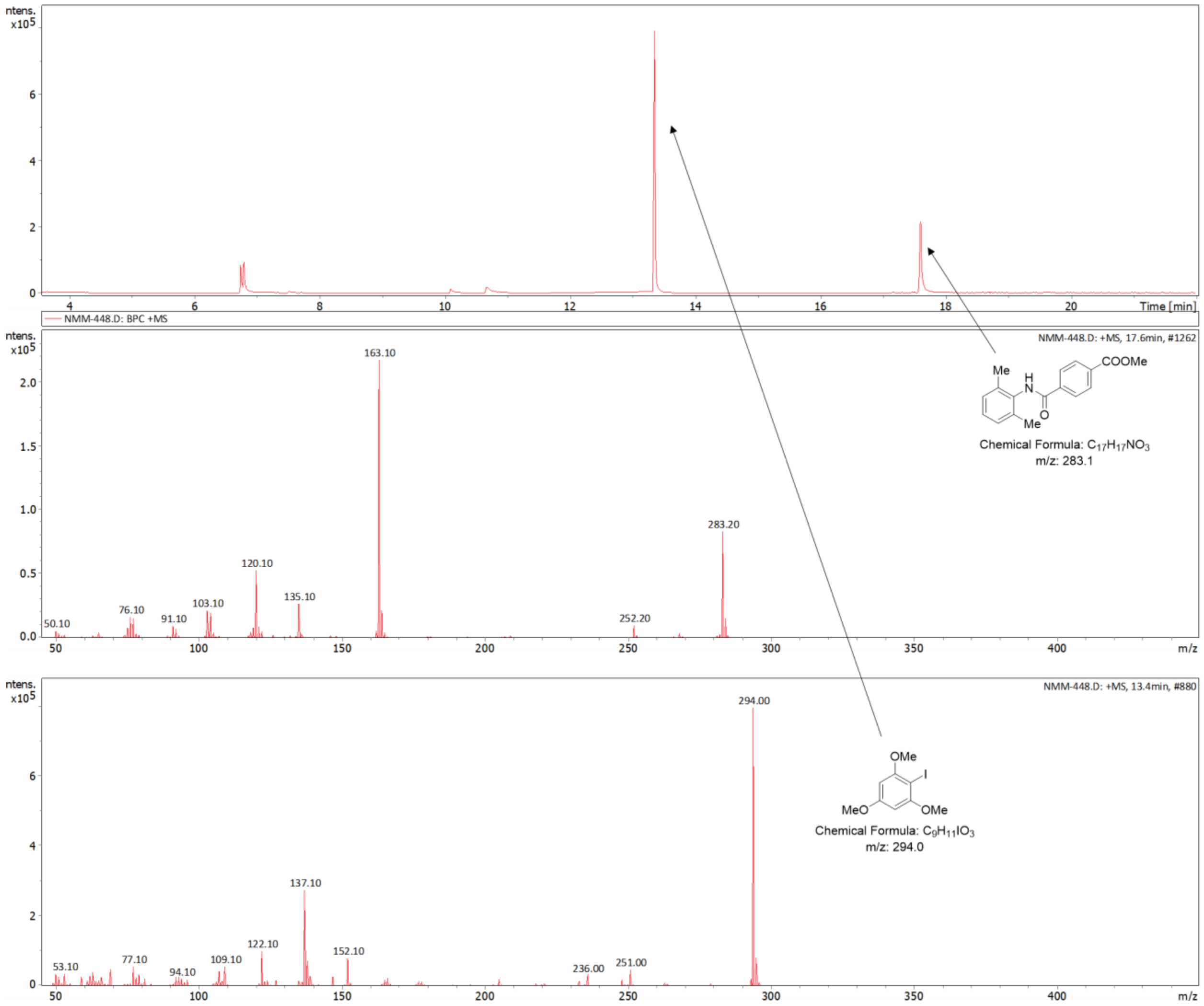

Figure S15. GC–MS data from the reaction mixture with iodonium salt **1p**

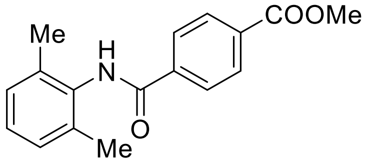

2bp: according to ***GP4*** 2-isocyano-1,3-dimethylbenzene (0.2 mmol, 26 mg) reacted with (4-(methoxycarbonyl)phenyl)(2,4,6-trimethoxyphenyl)iodonium triflate (**1p**, 0.2 mmol, 116 mg) followed by column chromatography (gradient elution: hexane → hexane:EtOAc – 10:1 → 7/1) to give methyl 4-((2,6-dimethylphenyl)carbamoyl)benzoate (**2bp**) as an off-white solid, 24 mg (42%). mp = 198 – 199 °C (lit. 192 – 194 °C).[33] The analytical data is in accordance with previously published.[33]

^{1}H NMR (400 MHz, $CDCl_3$) δ 8.14 (d, *J* = 8.0 Hz, 2H), 7.96 (d, *J* = 8.0 Hz, 2H), 7.54 (br s, 1H), 7.18 – 7.12 (m, 3H), 3.96 (s, 3H), 2.27 (s, 6H).

^{13}C NMR (100 MHz, $CDCl_3$) δ 166.4, 165.2, 138.5, 135.6, 133.6, 133.1, 130.1, 128.5, 127.8, 127.4, 52.6, 18.6.

(4-Methoxyphenyl)(4-(trifluoromethyl)phenyl)iodonium triflate (1q)

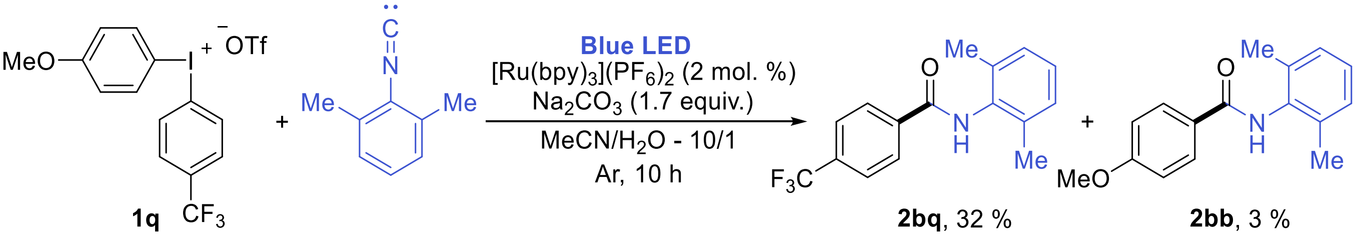

1q **2bq**, 32 % **2bb**, 3 %

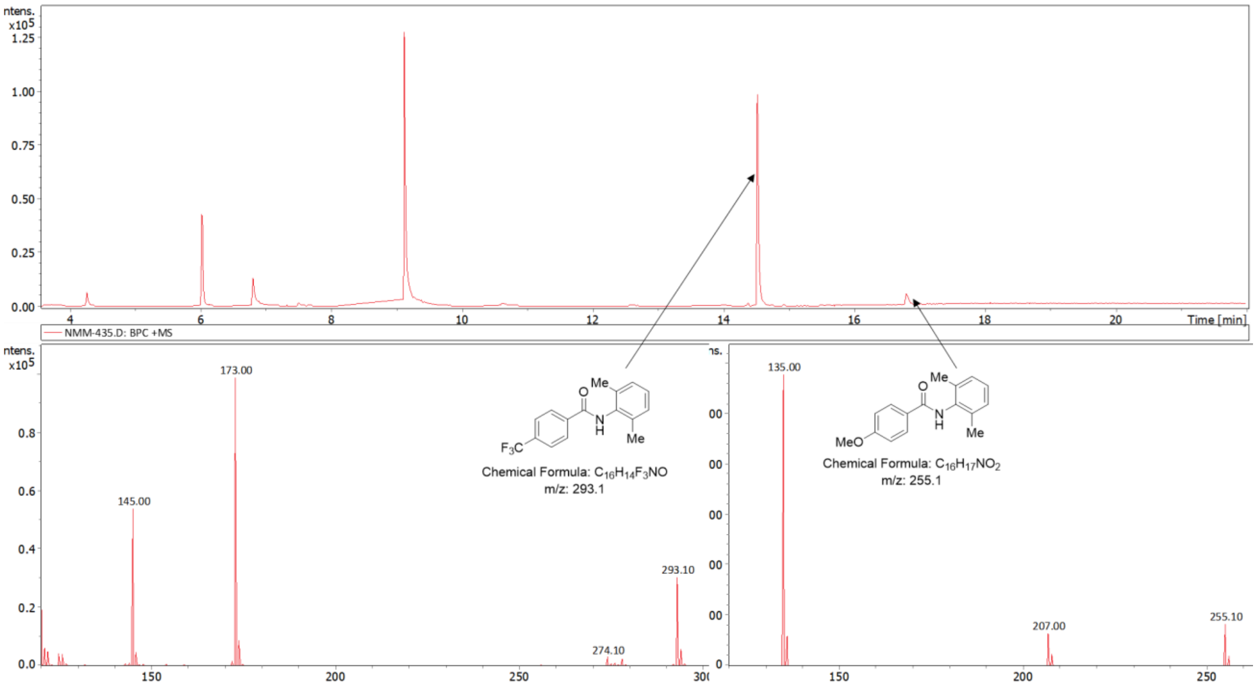

Figure S16. GC–MS data from the reaction mixture with iodonium salt **1q**

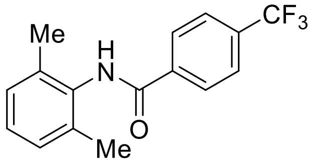

2bq: according to ***GP4*** 2-isocyano-1,3-dimethylbenzene (0.2 mmol, 26 mg) reacted with (4-methoxyphenyl)(4-(trifluoromethyl)phenyl)iodonium triflate (**1q**, 0.2 mmol, 106 mg) followed by column chromatography (hexane → hexane:EtOAc – 10:1) to give *N*-(2,6-dimethylphenyl)-4-(trifluoromethyl)benzamide (**2bq**) as a white solid, 19 mg (32%). mp = 206 – 207 °C (lit. 205 – 207 °C).[24] The analytical data is in accordance with previously published.[24]

^{1}H NMR (400 MHz, $CDCl_3$) δ 7.97 (d, *J* = 8.0 Hz, 2H), 7.71 – 7.68 (m, 3H), 7.20 – 7.14 (m, 1H), 7.11 (d, *J* = 7.2 Hz, 2H), 2.24 (s, 6H).

^{13}C NMR (100 MHz, $CDCl_3$) δ 164.9, 137.7, 135.6, 133.6 (q, *J* = 32.6 Hz), 133.6, 128.5, 127.9, 127.8, 125.9 (q, *J* = 3.6 Hz), 123.8 (q, *J* = 271.0 Hz), 18.54.

^{19}F NMR (376 MHz, $CDCl_3$) δ -62.9

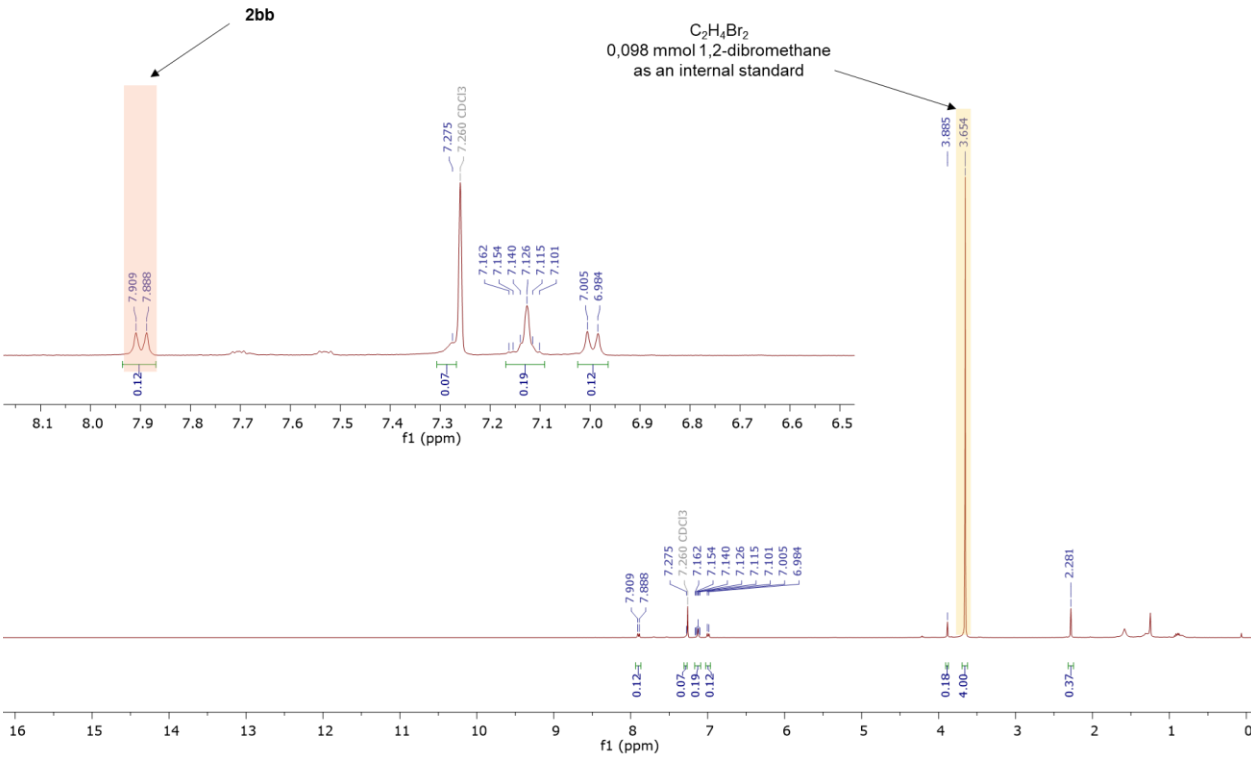

Figure S17. ^{1}H NMR of **2bb** with internal standard after column chromatography (the analytical data is in accordance with amide **2bb** synthesized from symmetrical iodonium salt **1b**)

5. Control experiments

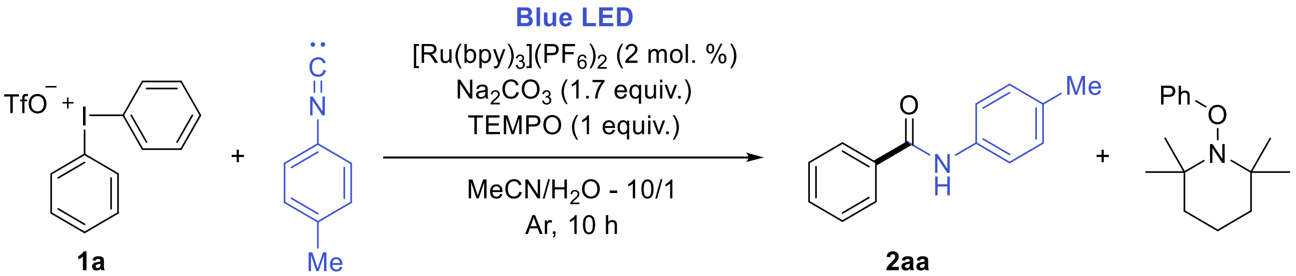

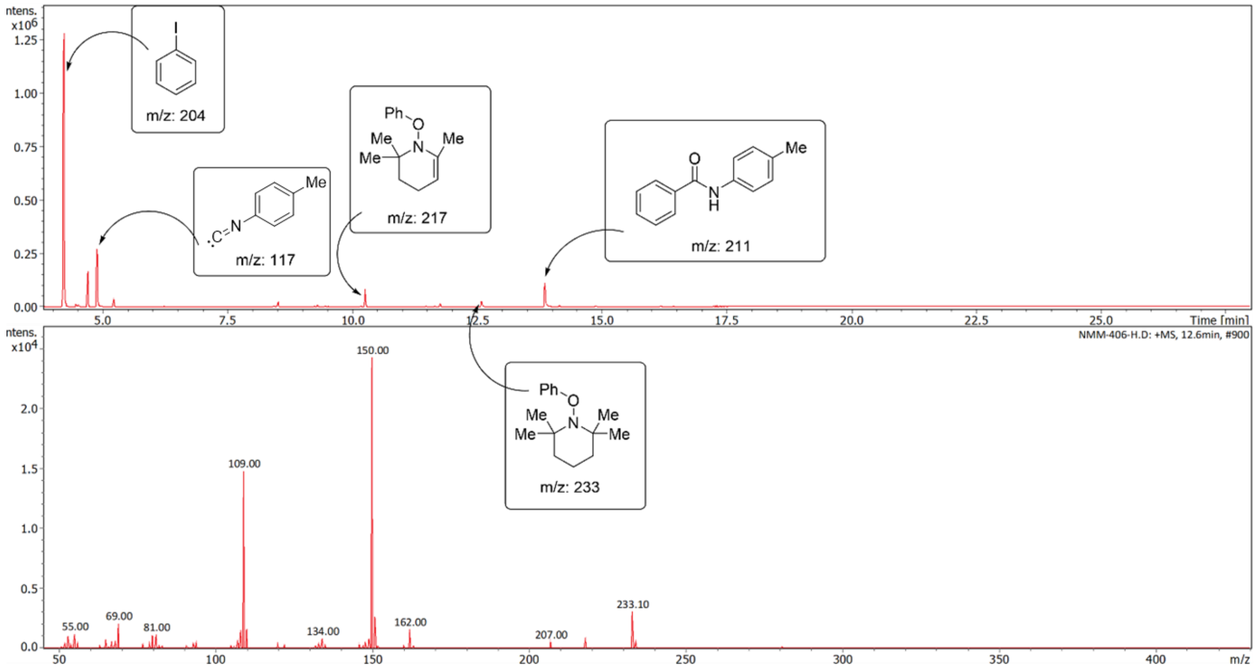

Figure S18. Radical trapping experiment.

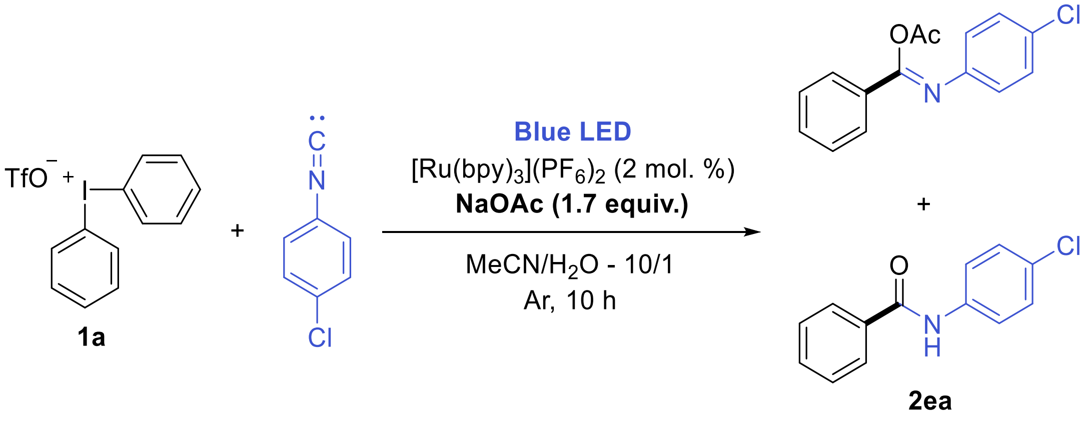

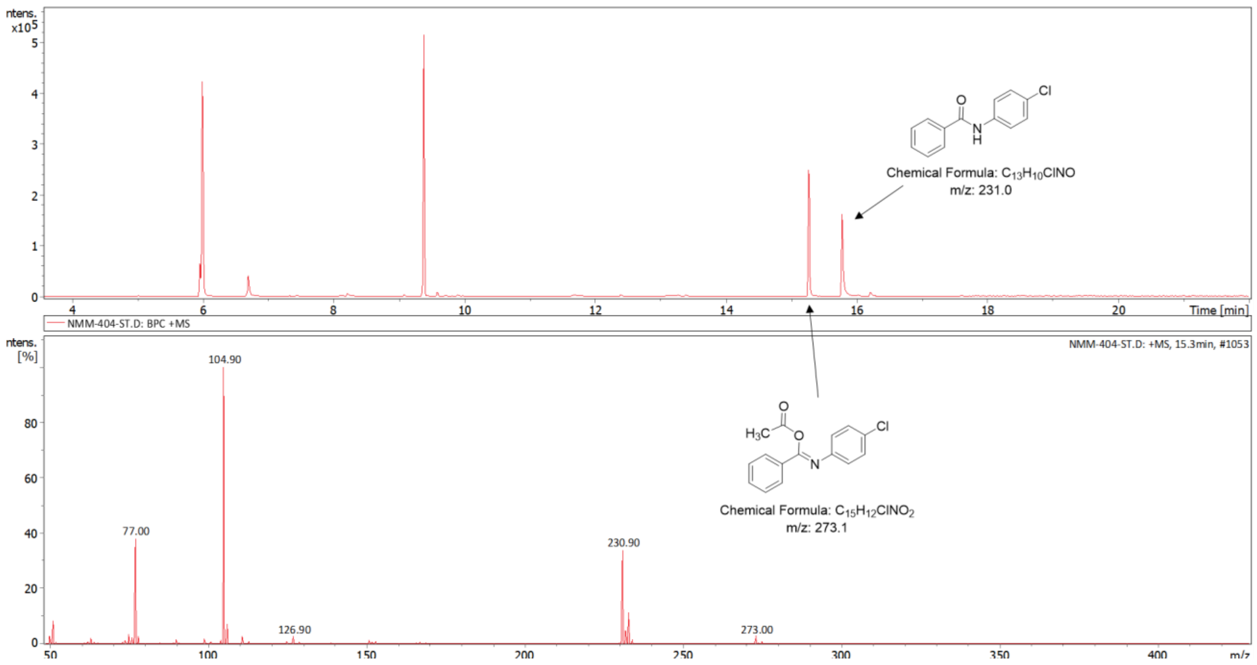

Figure S19. Experiment with sodium acetate as a base

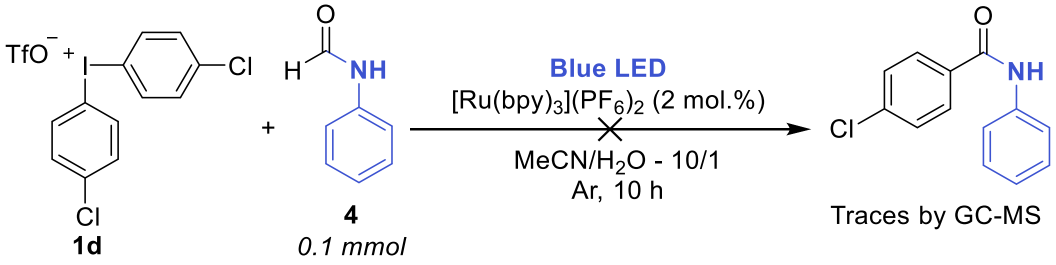

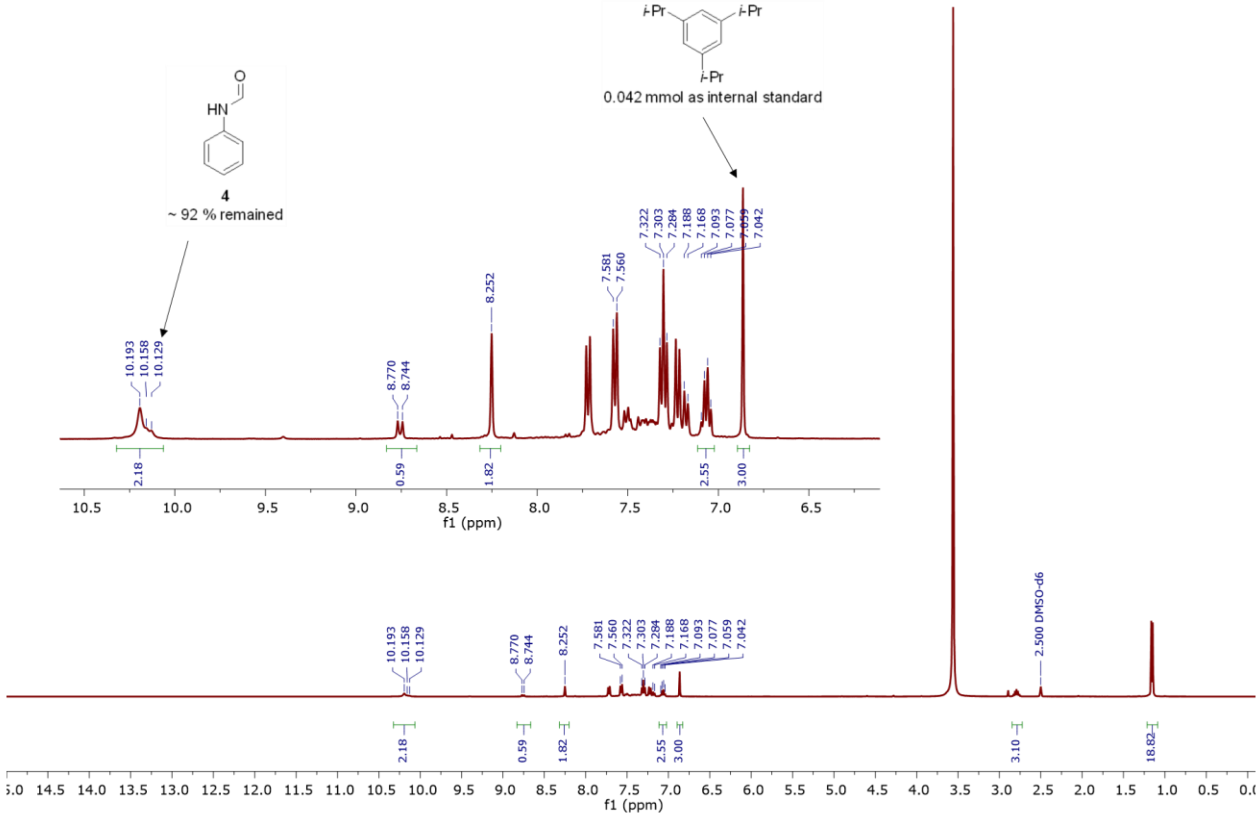

Figure S20. Experiment with formamide **4** as a substrate.

References

1. Armarego, W. L., Purification of Laboratory Chemicals (6th Edition); Elsevier Inc., London, 2009.
2. Becker, H.; Berger, W., Domschke, G. Organicum: Practical Handbook of Organic Chemistry; Elsevier, 2013.
3. Zhang, Z.; Ying, Y., Liebeskind, L. S. *Org. Lett.*, **2008**, *10*, 3005–3008.
4. Lew T. T., Lim D. S., Zhang Y. *Green Chem.*, **2015**, *17*, 5140-5143.
5. Bielawski, M.; Zhu, M.; Olofsson, B. *Adv. Synth. Catal*. **2007**, *349*, 2610–2618.
6. Vlasenko, Y. A.; Kuczmera, T. J.; Antonkin, N. S.; Valiev, R. R.; Postnikov, P. S.; Nachtsheim, B. *J. Adv. Synth. Catal*. **2023**, *365*, 535–543.
7. Podrezova, E. V.; Okhina, A. A.; Rogachev, A. D.; Baykov, S. V.; Kirschning, A.; Yusubov, M. S.; Soldatova, N. S.; Postnikov, P. S. *Org. Biomol. Chem*. **2023**, *21*, 1952–1957.
8. Soldatova, N.; Postnikov, P.; Kukurina, O.; Zhdankin, V. V; Yoshimura, A.; Wirth, T.; Yusubov, M. S. *Beilstein J. Org. Chem*. **2018**, *14*, 849–855
9. Bielawski, M.; Aili, D.; Olofsson, B. *J. Org. Chem*. **2008**, *73,* 4602-4607.
10. Petersen, T. B.; Khan, R.; Olofsson B. *Org. Lett*. **2011**, *13*, 3462-3465.
11. Hossain, M. D.; Kitamura, T. *Bull. Chem. Soc. Jpn.*, **2007**, *80*, 2213-2219.
12. Laudadio, G.; Gemoets, H. P.; Hessel, V.; Noël, T. *J. Org. Chem*. **2017**, *82*, 11735–11741
13. Kuriyama, M.; Hamaguchi, N.; Onomura, O. *Chem. - Eur. J*. **2012**, *6*, 1591-1594.
14. Guselnikova, O.; Fraser, J. P.; Soldatova, N.; Sviridova, E.; Ivanov, A.; Rodriguez, R.; Ganin, A. Y.; Postnikov, P. *Mater. Today Chem*. **2022**, *24*, 100846.
15. Phipps, R. J.; Grimster, N. P.; Gaunt, M. J. *J. Am. Chem. Soc*. **2008**, *130*, 8172-8174.
16. Reay, A. J.; Williams, T. J.; Fairlamb, I. *J. Org. Biomol. Chem*. **2015**, *13*, 8298-8309.
17. Malmgren, J.; Santoro, S.; Jalalian, N.; Himo, F.; Olofsson B. *Chem. - Eur. J*. **2013**, *19*, 10334-10342.
18. Gallagher, R. T.; Basu, S.; Stuart, D. R. *Adv. Synth. Catal*. **2020**, *362*, 320-325.
19. Seidl, T. L.; Stuart, D. R. *J. Org. Chem*. **2017**, *82*, 11765-11771.
20. Kervefors, G.; Kersting, L.; Olofsson, B. *Chem. - Eur. J*. **2021**, *27*, 5790-5795.
21. Chang, R. K.; Clairmont, B. P.; Lin, S.; MacArthur, A. H. *Organometallics*. **2019**, *38*, 4448-4454.
22. Gockel, S. N.; Hull, K. L. *Org. Lett*. **2015**, *17*, 3236-3239.
23. Shi, S.; Szostak, M. *ChemComm*. **2017**, *53*, 10584-10587.

24. Guo, W.; Huang, J.; Wu, H.; Liu, T.; Luo, Z.; Jian, J.; Zeng, Z. *Org. Chem. Front*. **2018**, *5*, 2950-2954.
25. Quesnel, J. S.; Arndtsen, B. A. *J. Am. Chem. Soc*. **2013**, *135*, 16841-16844.
26. Huy, P. H.; Mbouhom, C. *Chem. Sci*., **2019**, *10*, 7399-7406.
27. Zhu, Y. P.; Sergeyev, S.; Franck, P.; Orru, R. V.; Maes, B. U. *Org. Lett*. **2016**, *18*, 4602-4605.
28. Wang, S. P.; Cheung, C. W.; M,a J. A. *J. Org. Chem*. **2019**, *84*, 13922-13934.
29. Pelletier, G.; Bechara, W. S.; Charette, A. B. *J. Am. Chem. Soc*. **2010**, *132*, 12817-12819.
30. Singh, G.; Dada, R.; Yaragorla, S. *Tetrahedron Lett*. **2016**, *57*, 4424-4427.
31. Wang, S. M.; Zhao, C.; Zhang, X.; Qin, H.L. *Org. Biomol. Chem.* **2019**, *17*, 4087-4101.
32. Meng, G.; Lei P.; Szostak, M. *Org. Lett*. **2017**, *19*, 2158-2161.
33. Huang**,** P. Q.; Chen**,** H. *ChemComm*. **2017**, *53*, 12584-12587.

Spectral data

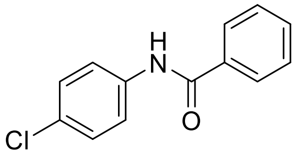

2ea, ^{1}H NMR
400 MHz, $CDCl_3$

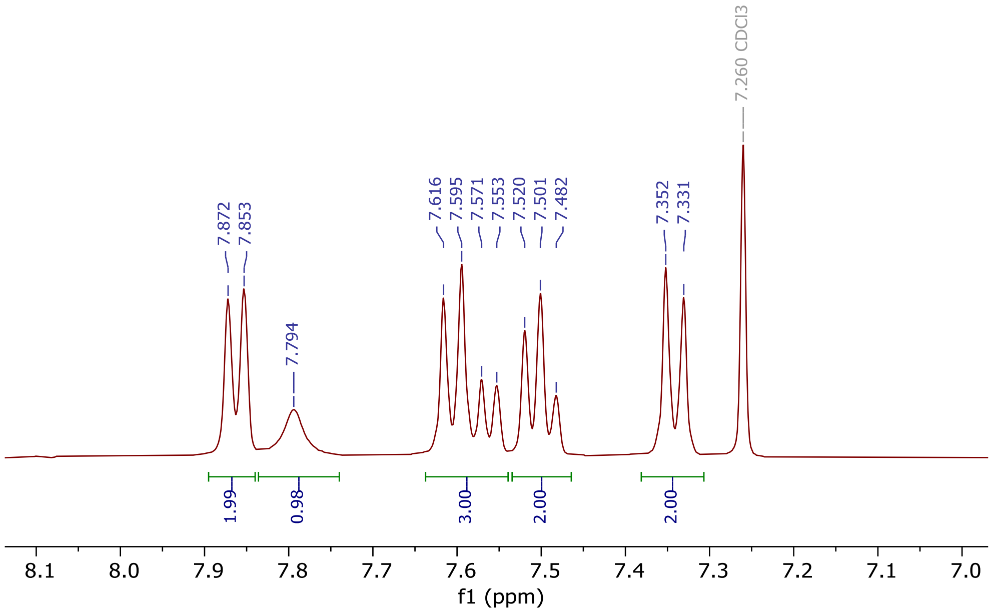

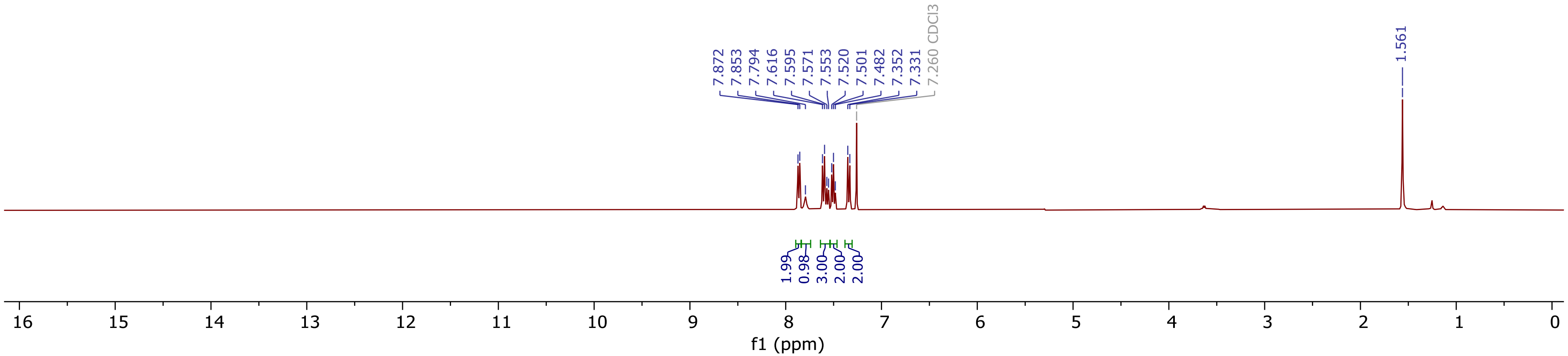

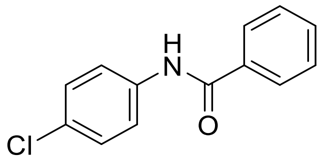

2ea, ^{13}C NMR
100 MHz, $CDCl_3$

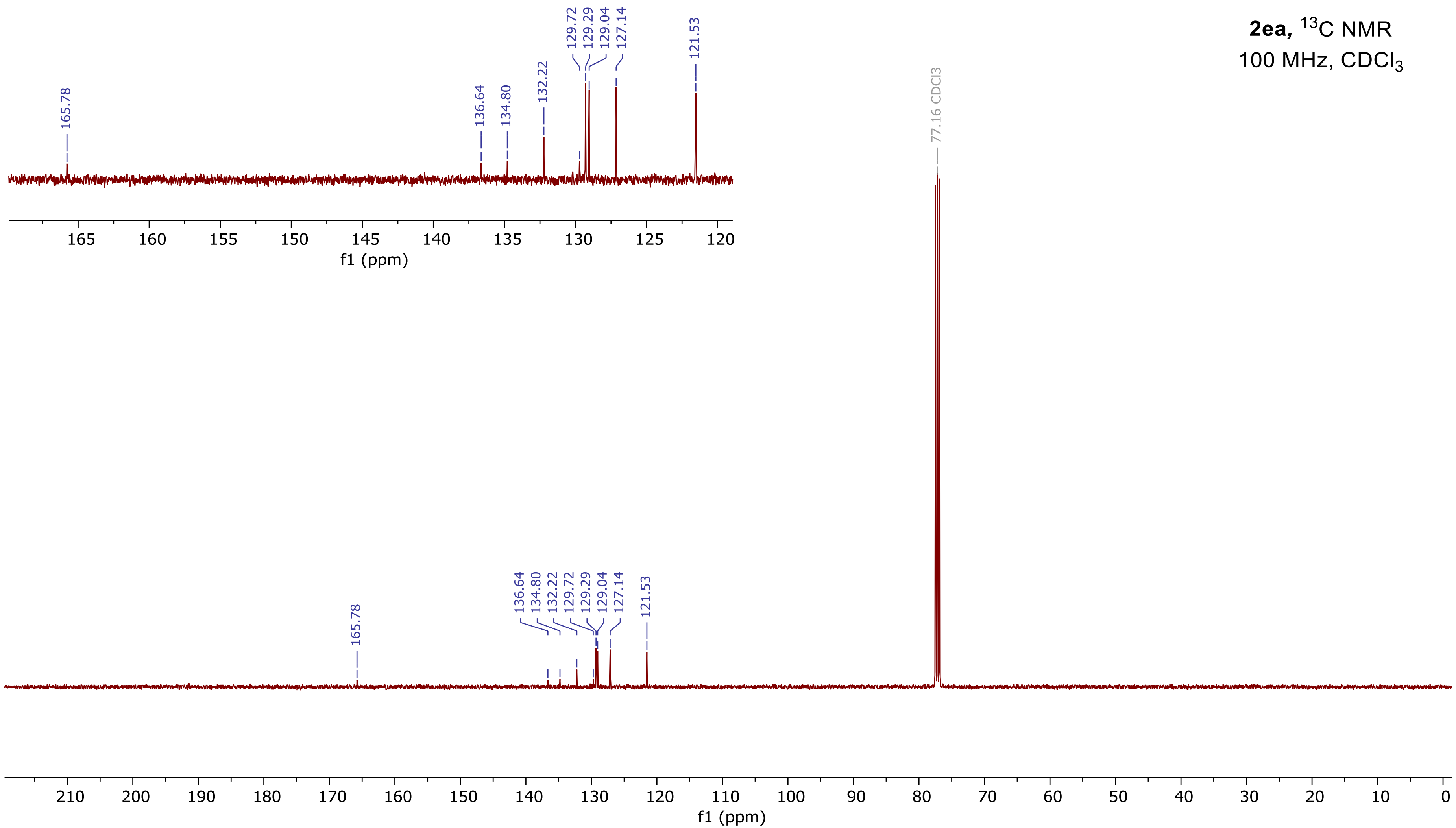

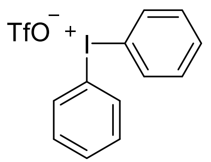

1a, ^{1}H NMR
400 MHz, DMSO-d_6

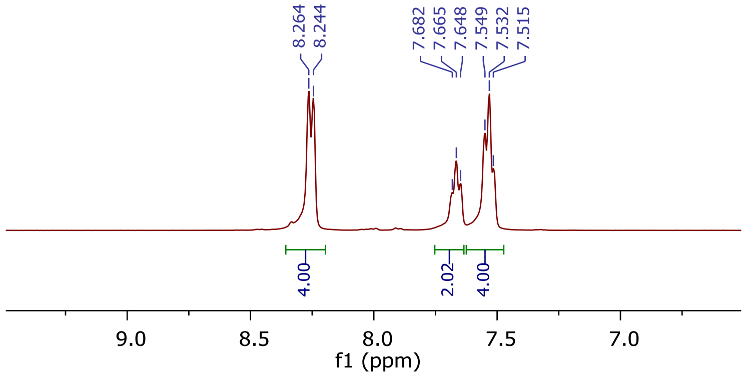

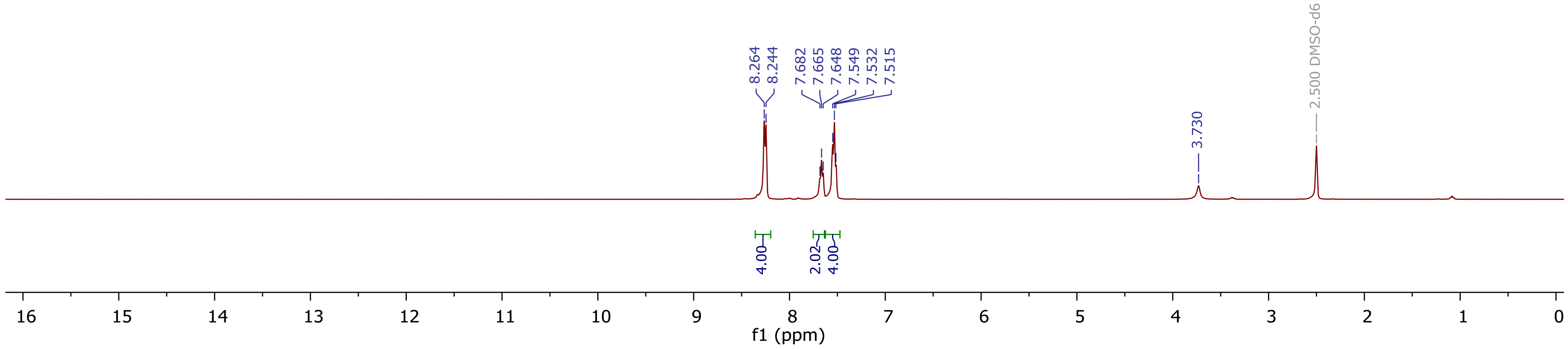

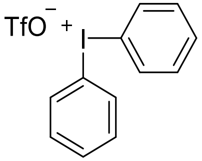

1a, ^{13}C NMR
100 MHz, DMSO-d_6

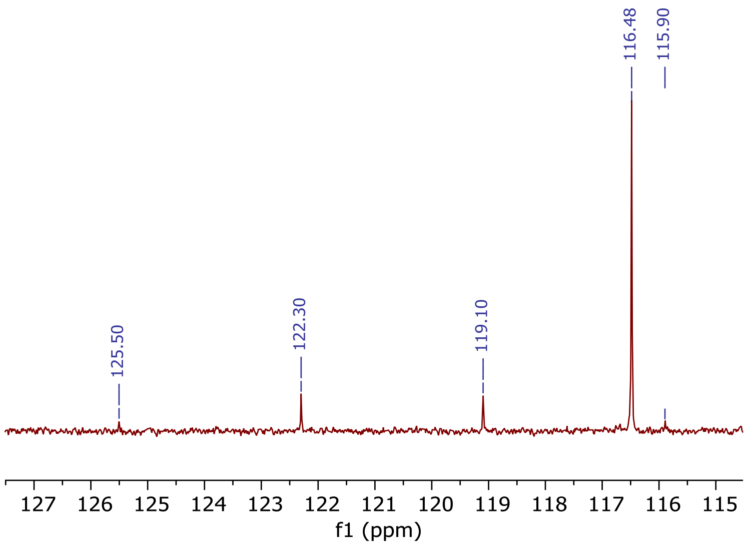

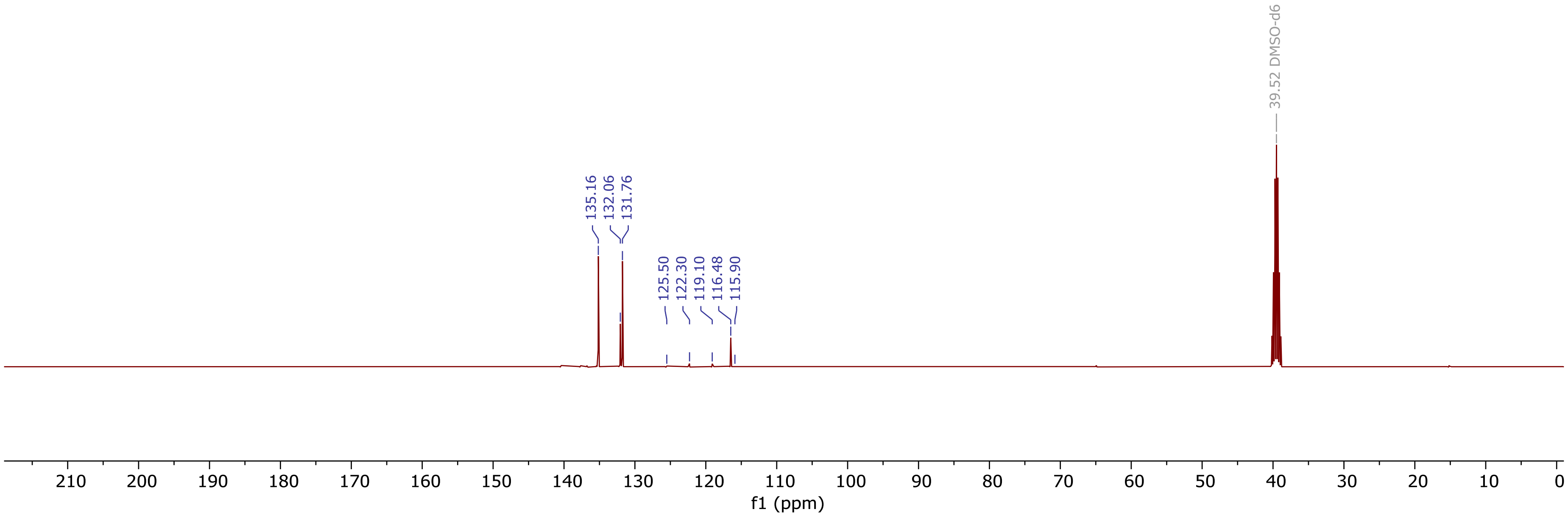

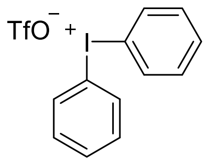

1a, ^{19}F NMR
376 MHz, DMSO-d_6

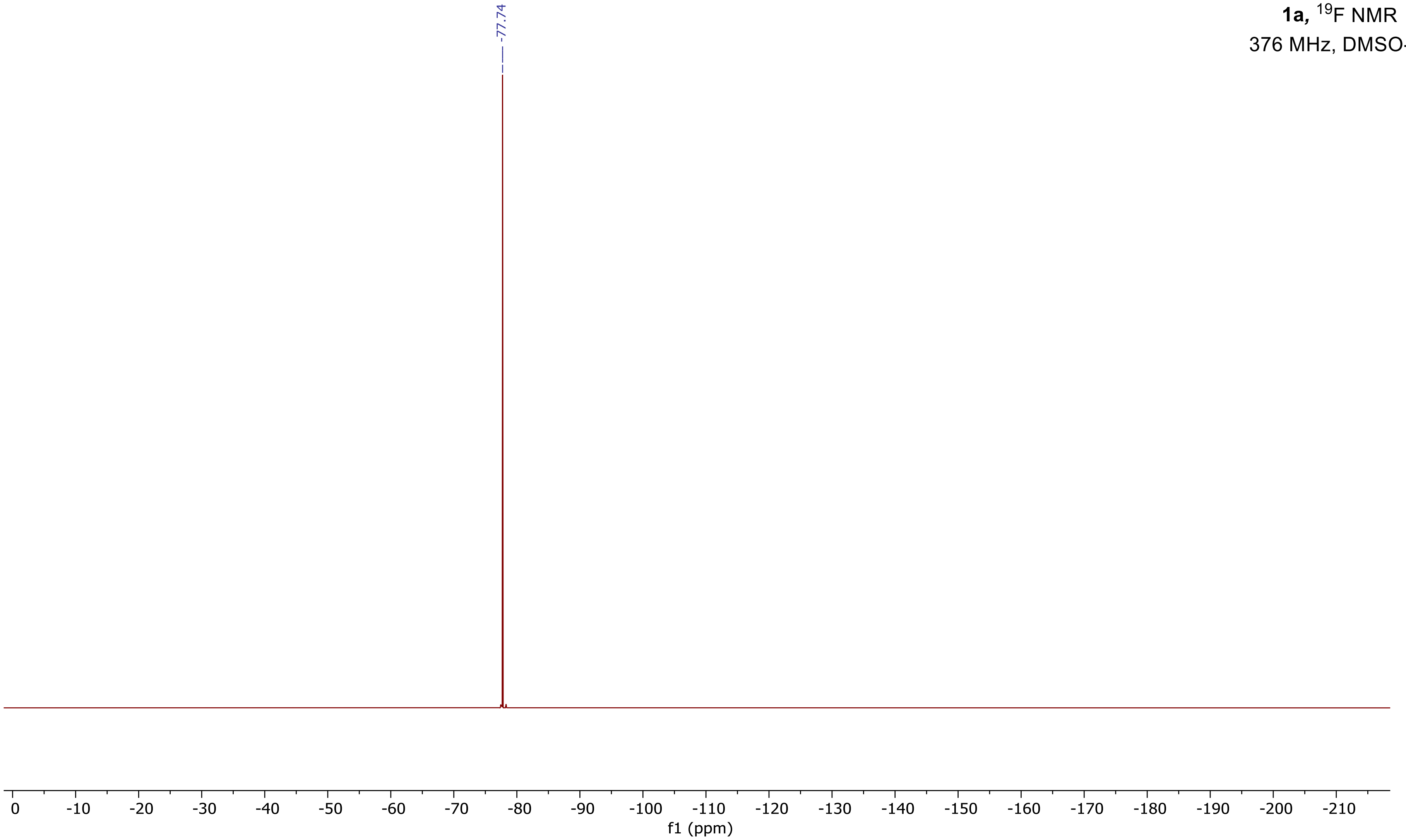

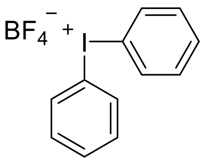

1a-BF$_4$, ^{1}H NMR
400 MHz, DMSO-d_6

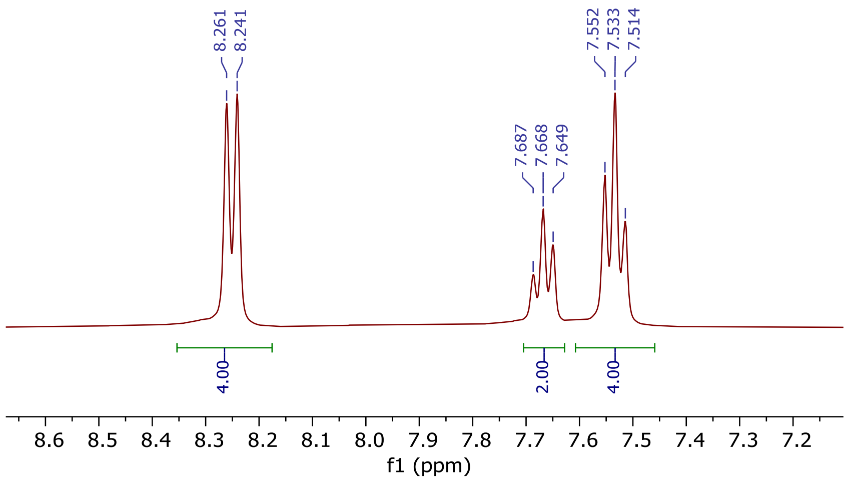

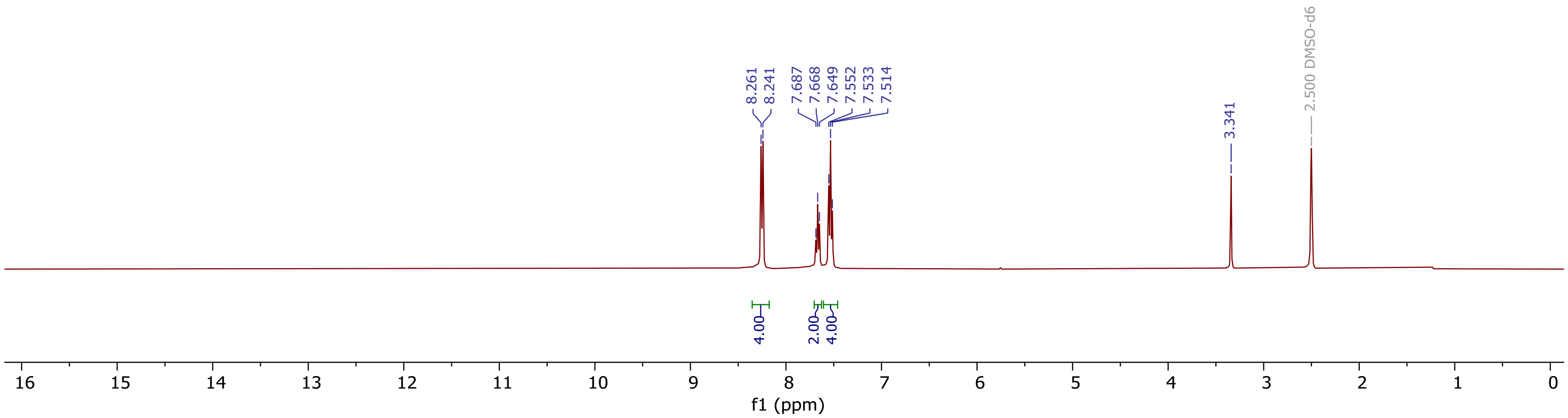

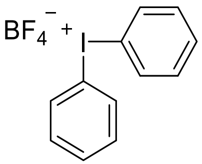

1a-BF$_4$, ^{13}C NMR
100 MHz, DMSO-d_6

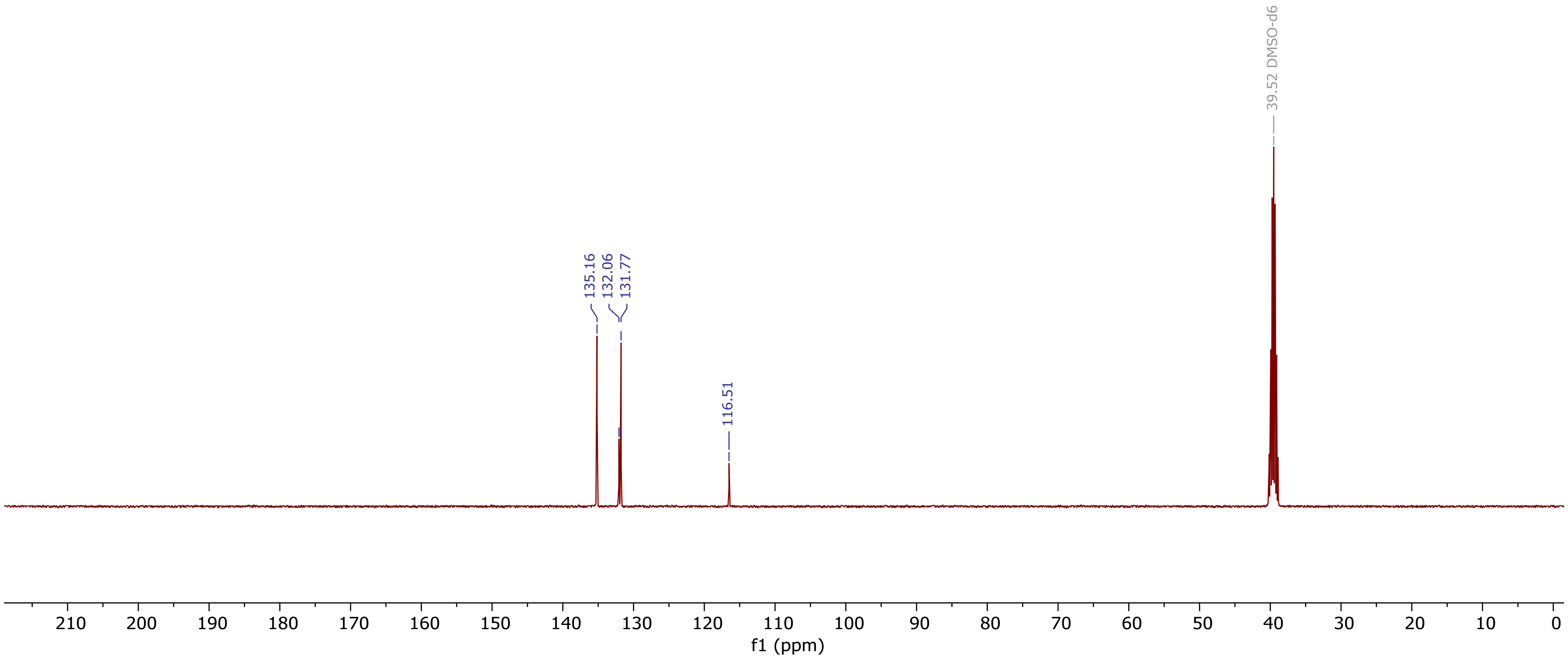

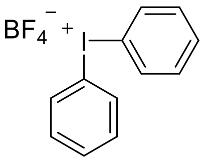

1a-BF_4, ^{19}F NMR
376 MHz, DMSO-d_6

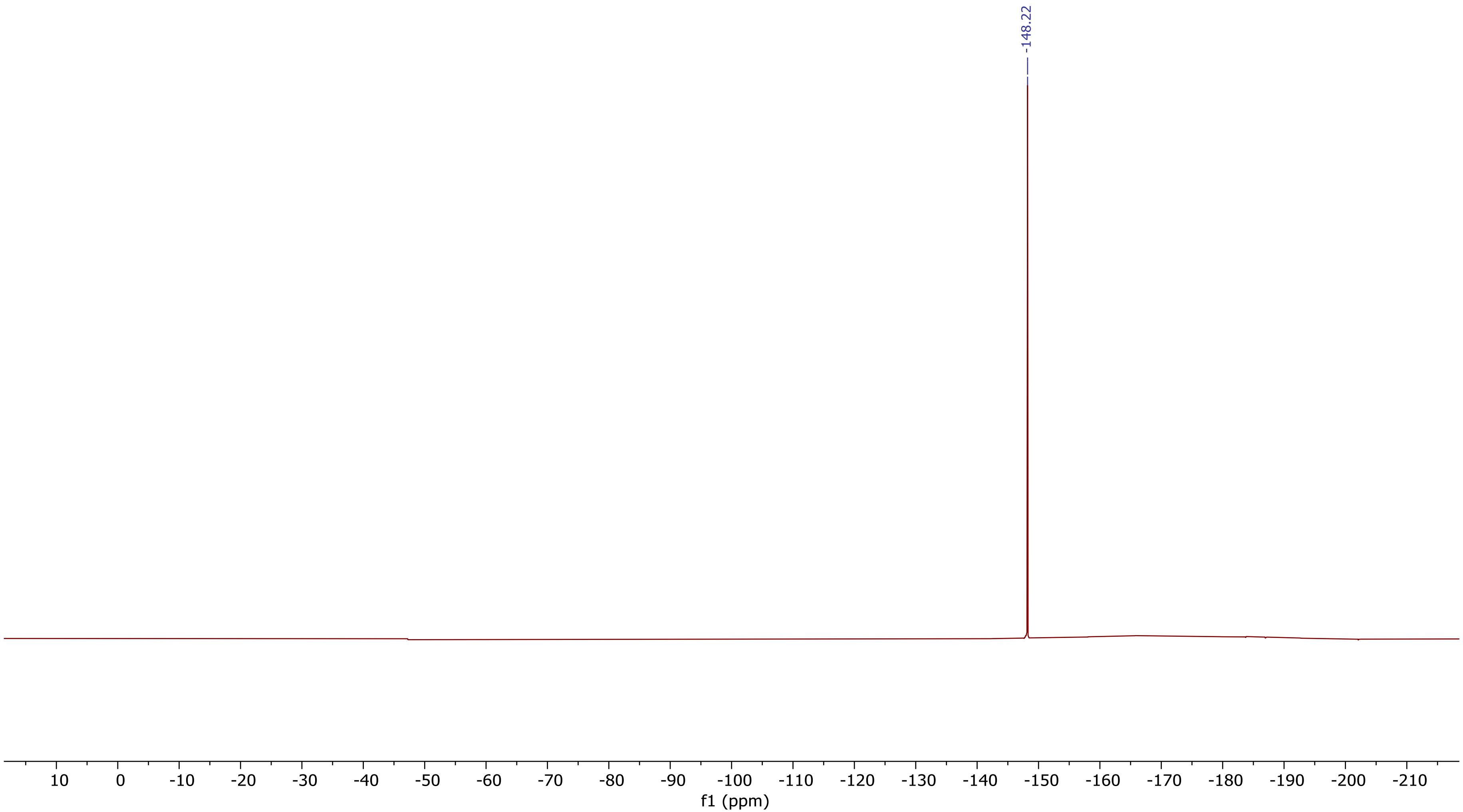

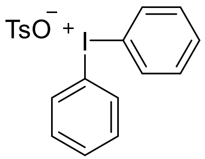

1a-TsO, ^{1}H NMR
400 MHz, DMSO-d_6

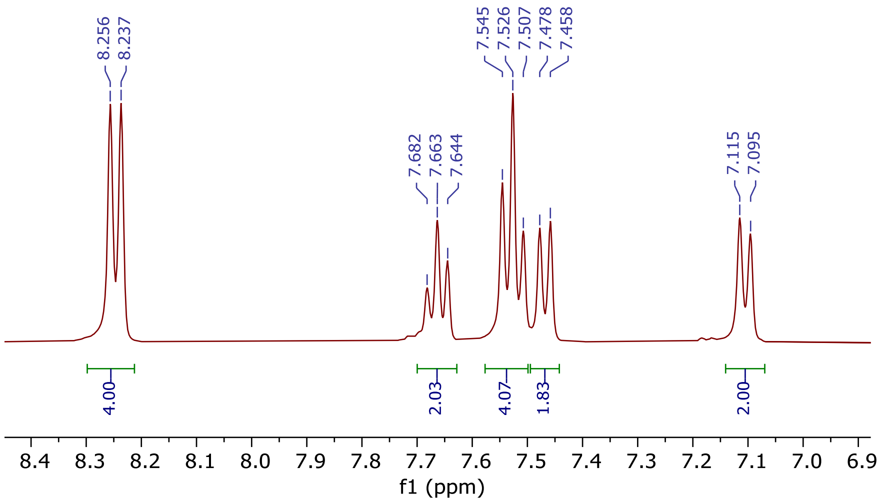

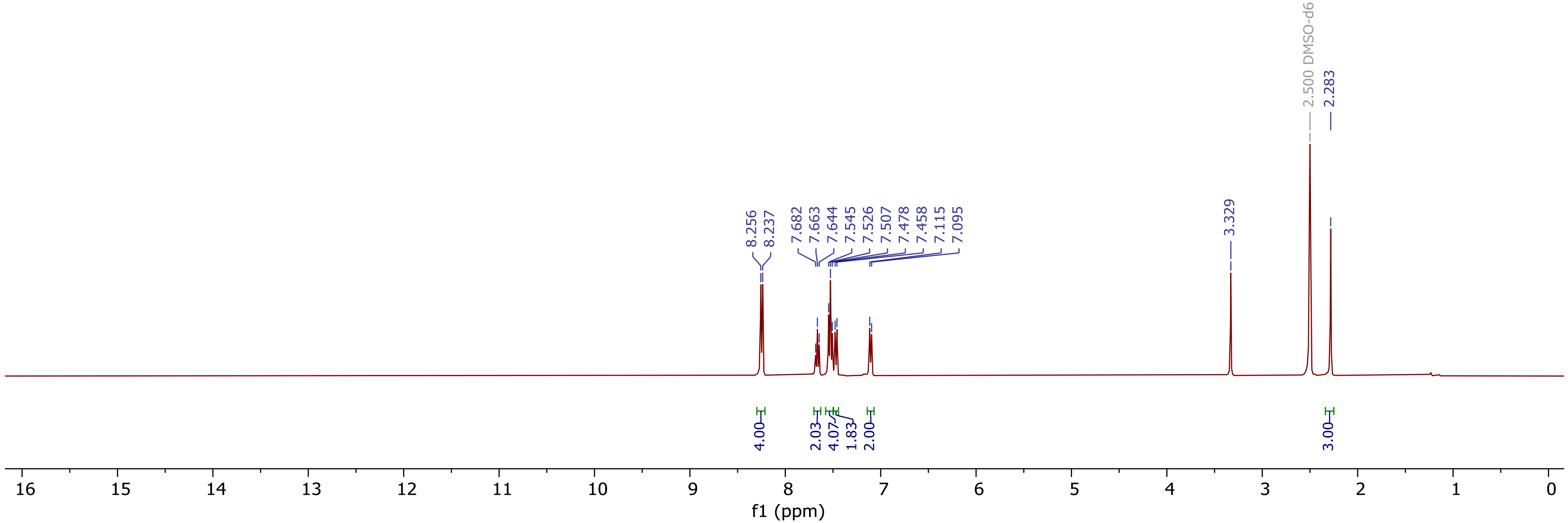

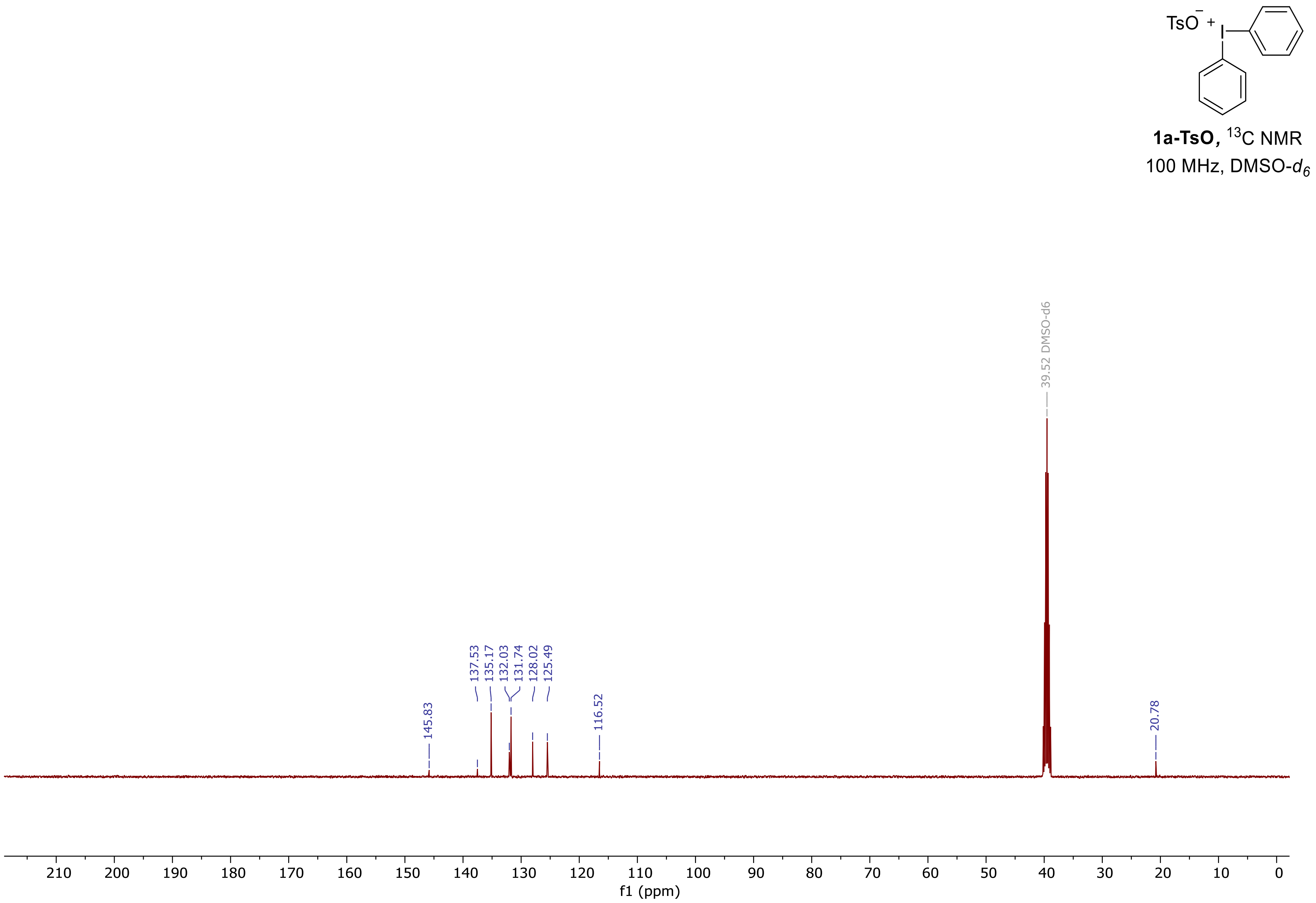
TsO⁻ +I
1a-TsO, 13C NMR
100 MHz, DMSO-d6
145.83
137.53
135.17
132.03
131.74
128.02
125.49
116.52
39.52 DMSO-d6
20.78
210
200
190
180
170
160
150
140
130
120
110
100
90
80
70
60
50
40
30
20
10
0
f1 (ppm)

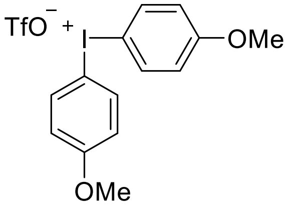

1b, ^{1}H NMR
400 MHz, DMSO-d_6

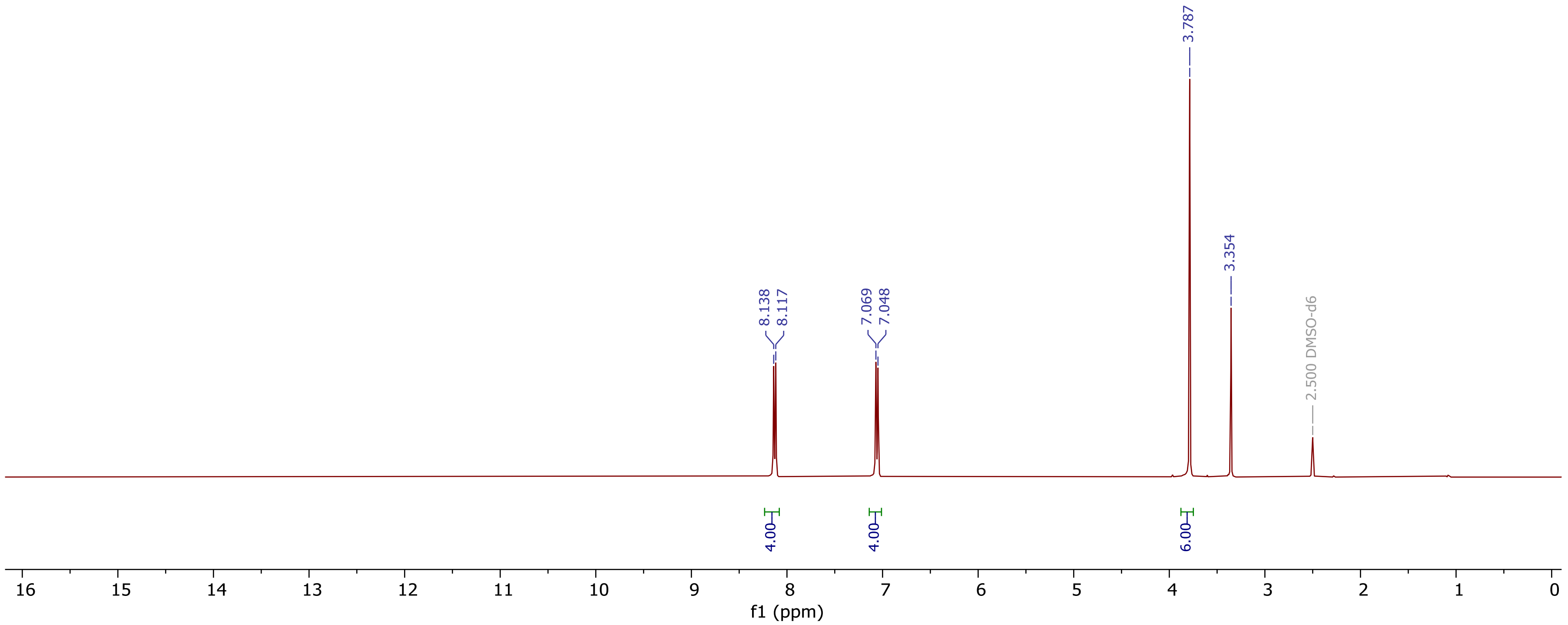

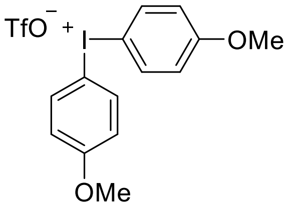

1b, ^{13}C NMR
100 MHz, DMSO-d_6

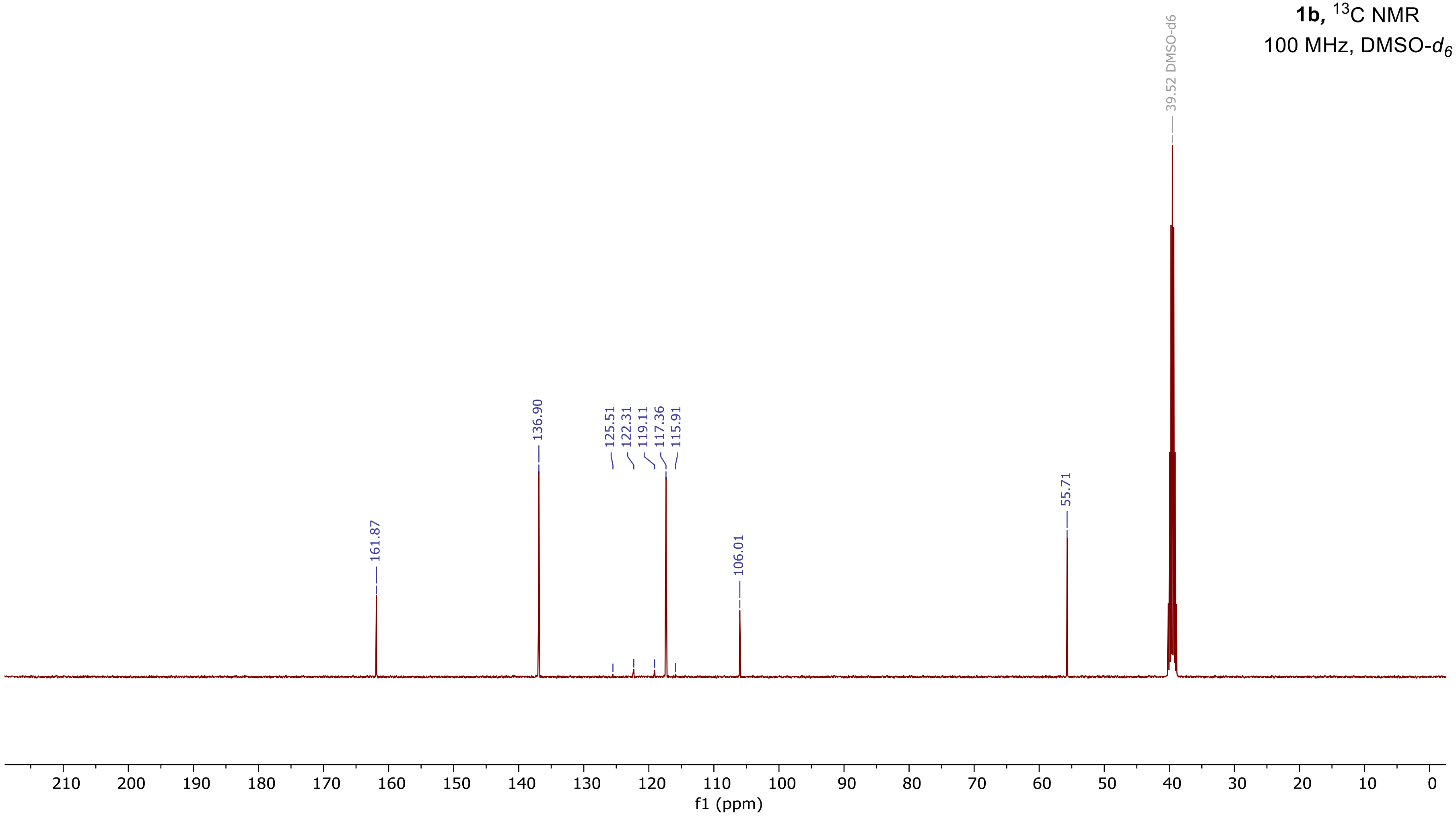

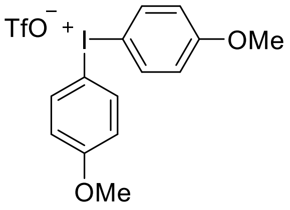

1b, ^{19}F NMR
376 MHz, DMSO-d_6

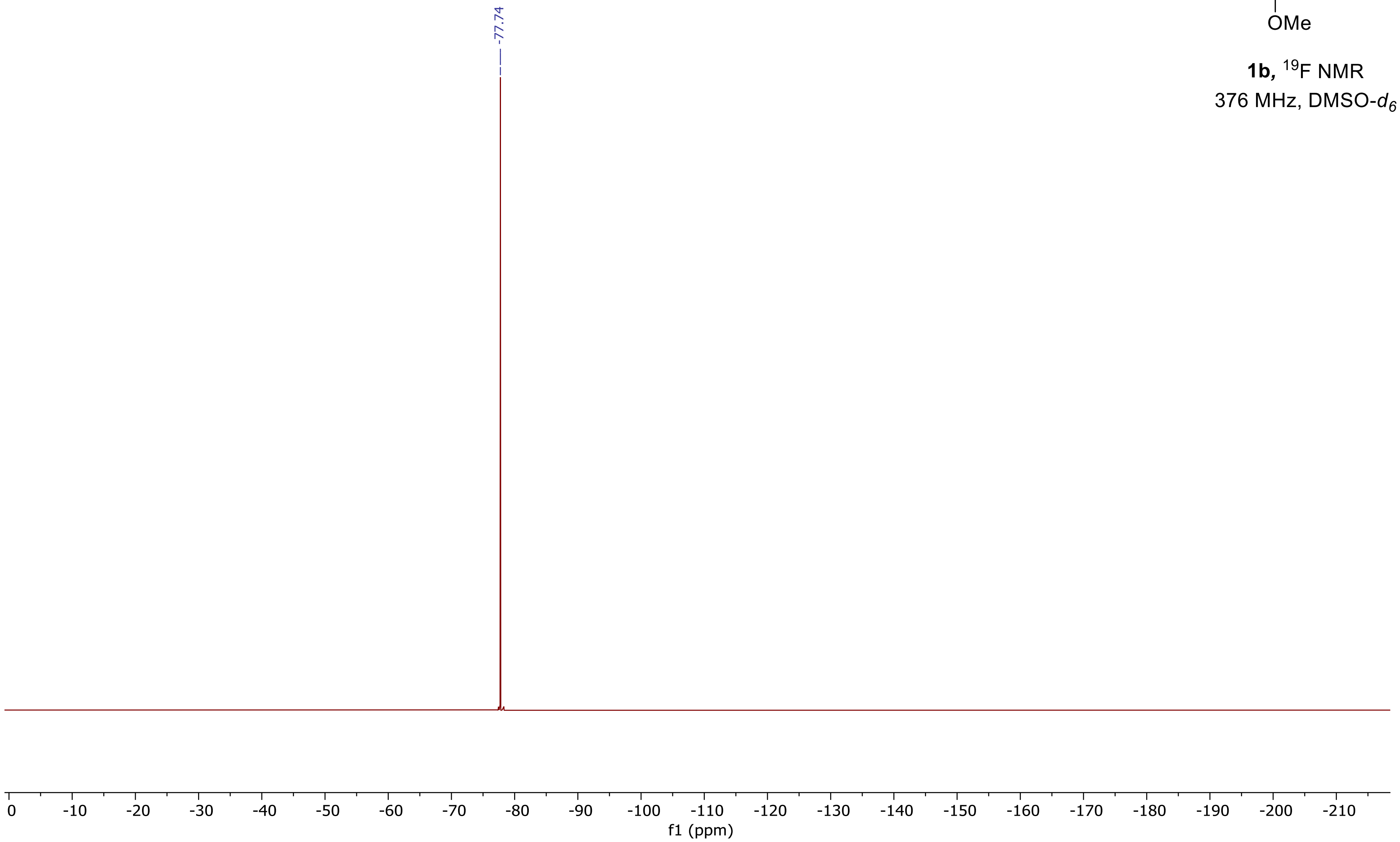

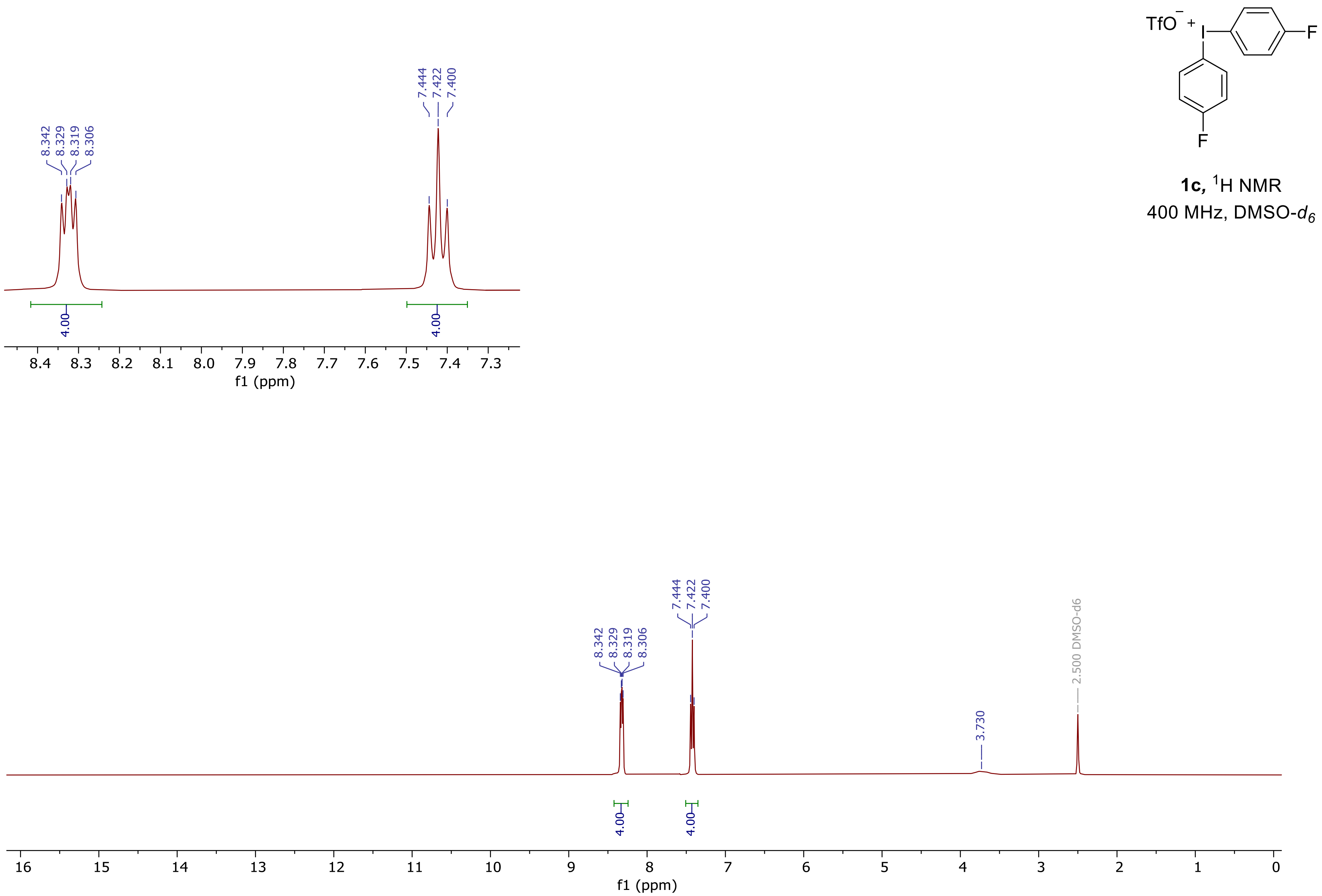

1c, ^{1}H NMR
400 MHz, DMSO-d_6

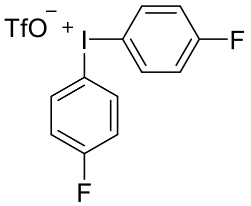

1c, ^{13}C NMR
100 MHz, DMSO-d_6

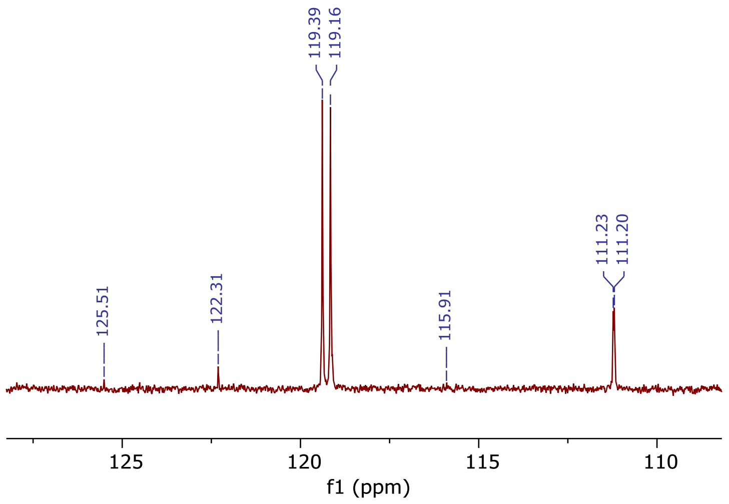

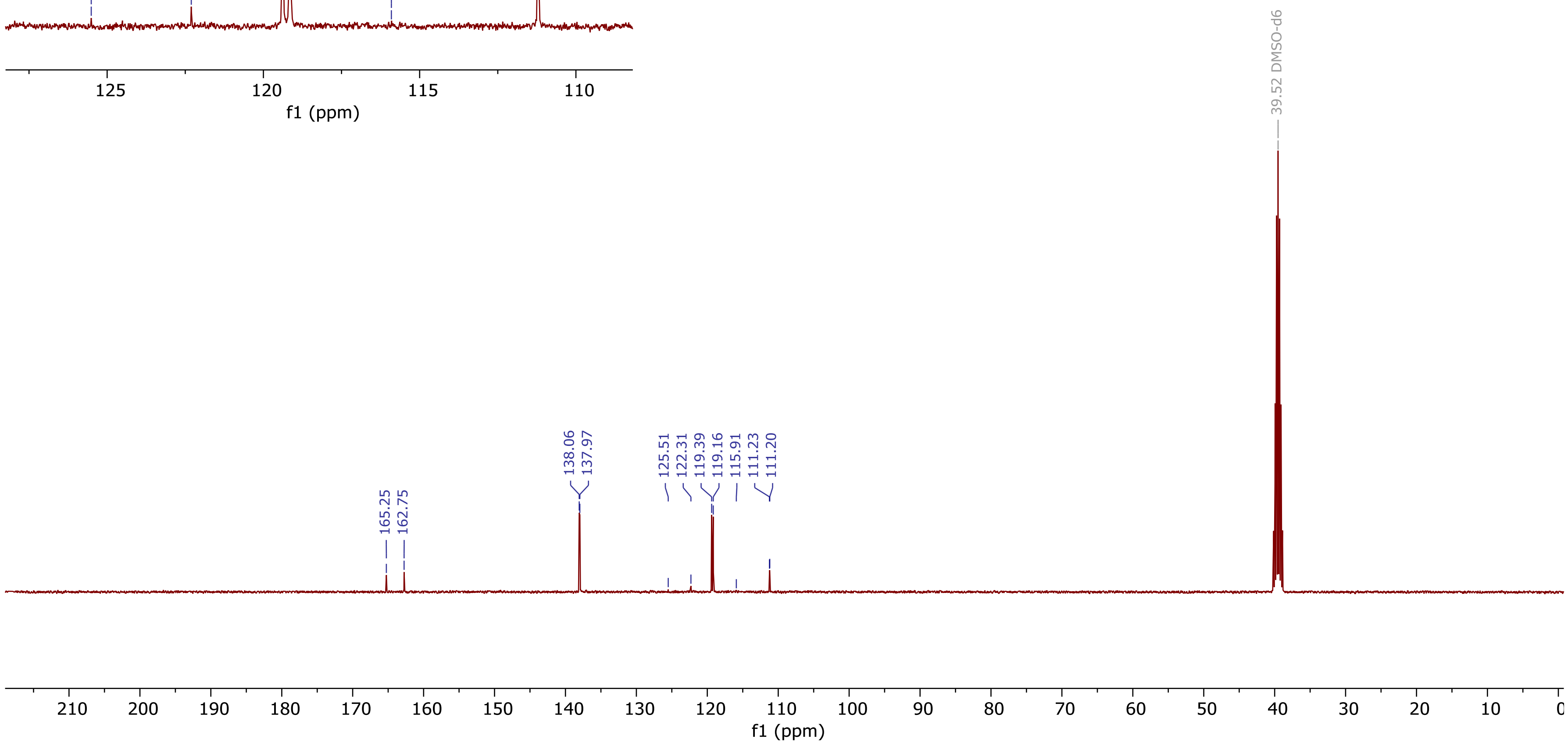

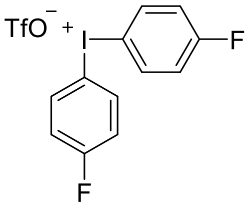

1c, ^{19}F NMR
376 MHz, DMSO-d_6

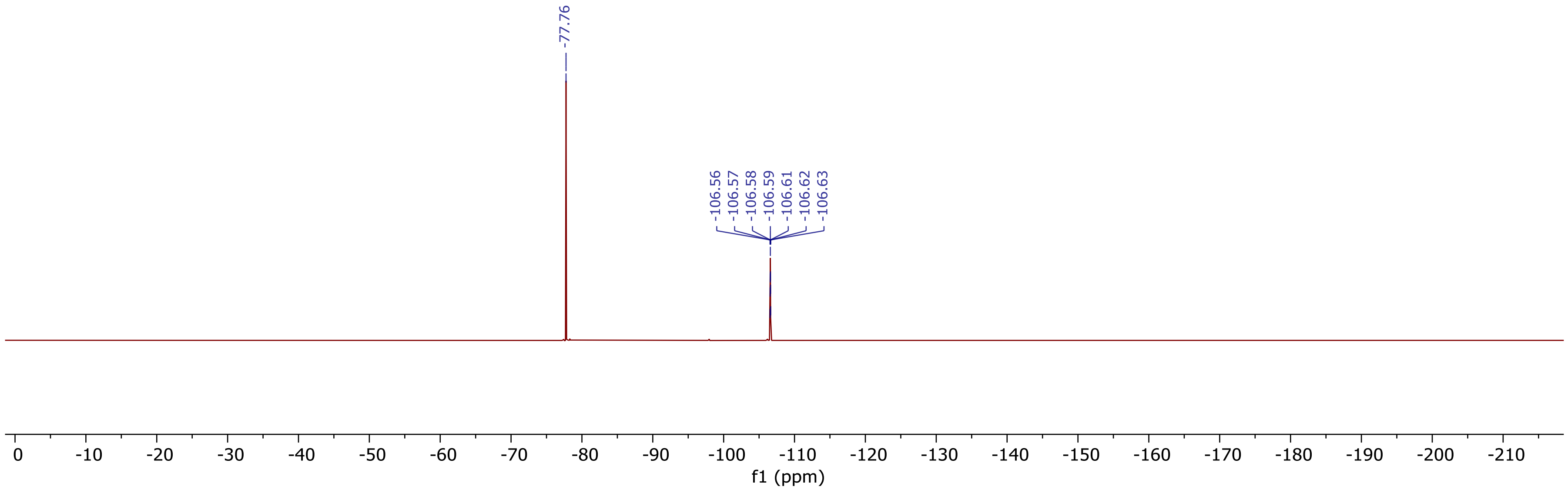

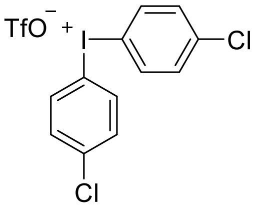

1d, ^{1}H NMR
400 MHz, DMSO-d_6

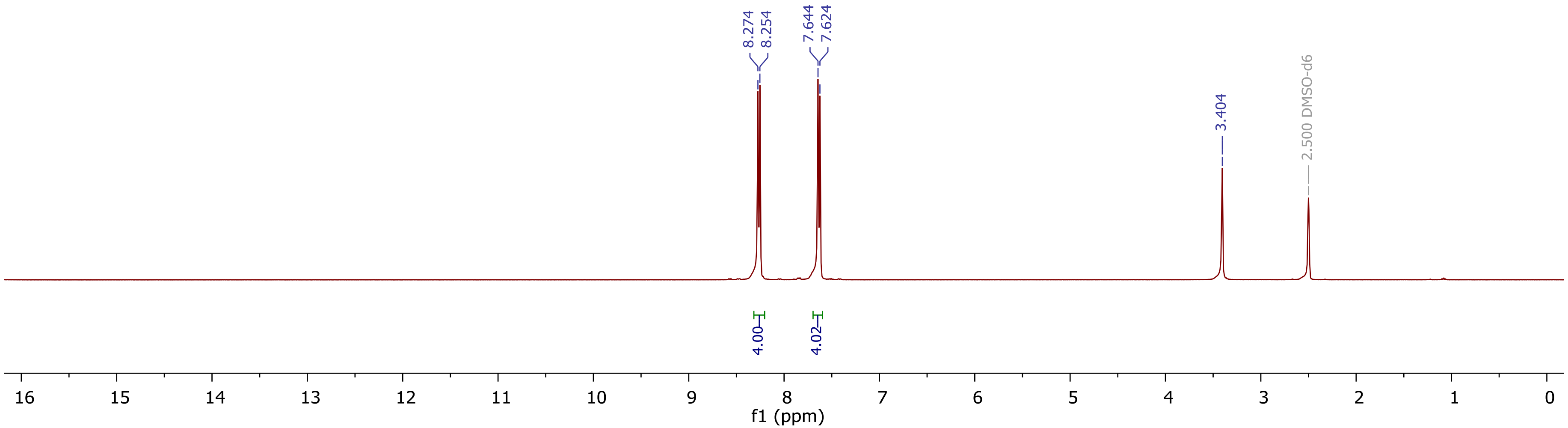

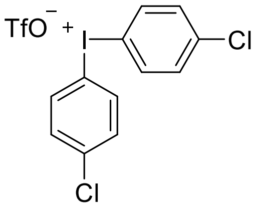

1d, ^{13}C NMR
100 MHz, DMSO-d_6

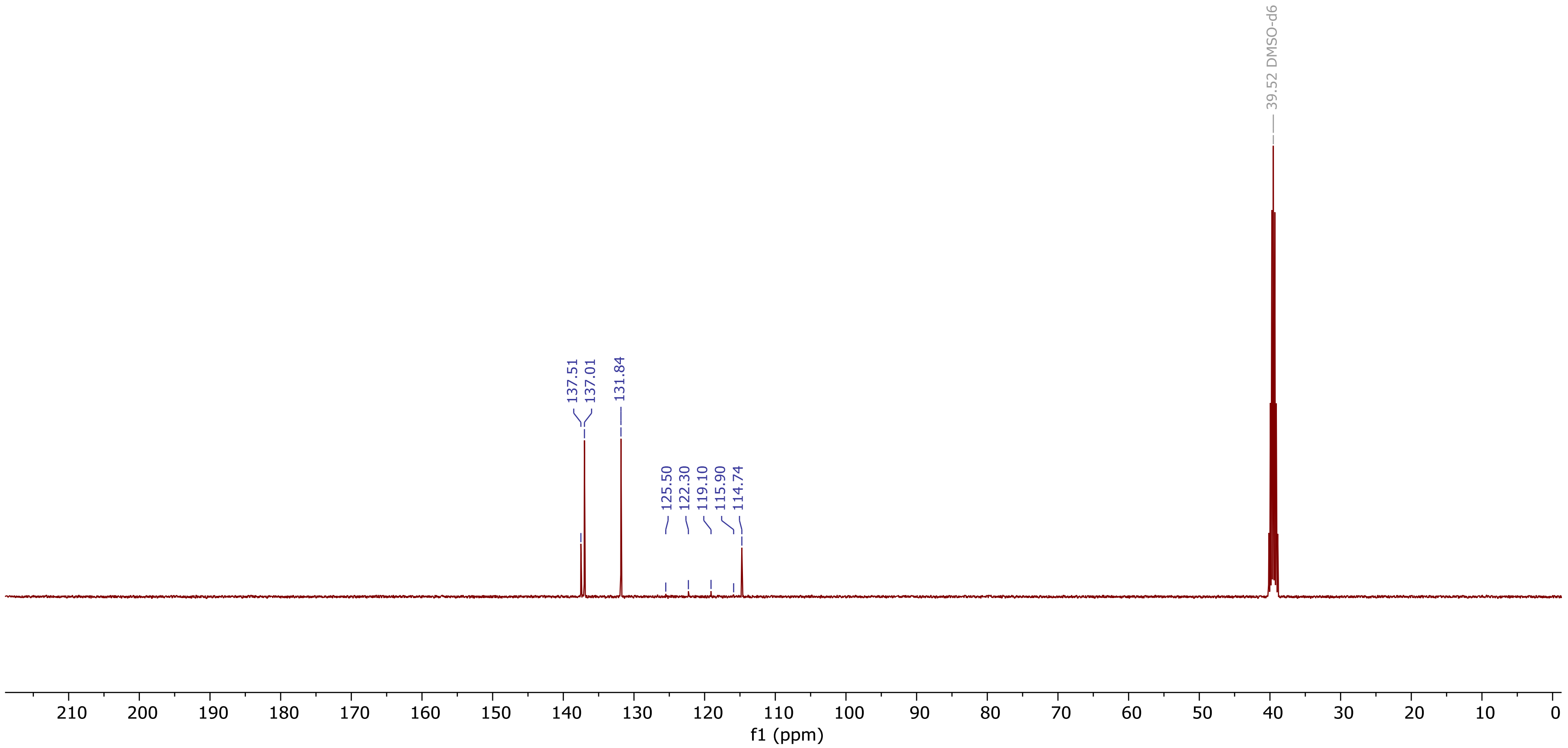

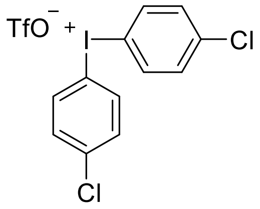

1d, ^{19}F NMR
376 MHz, DMSO-d_6

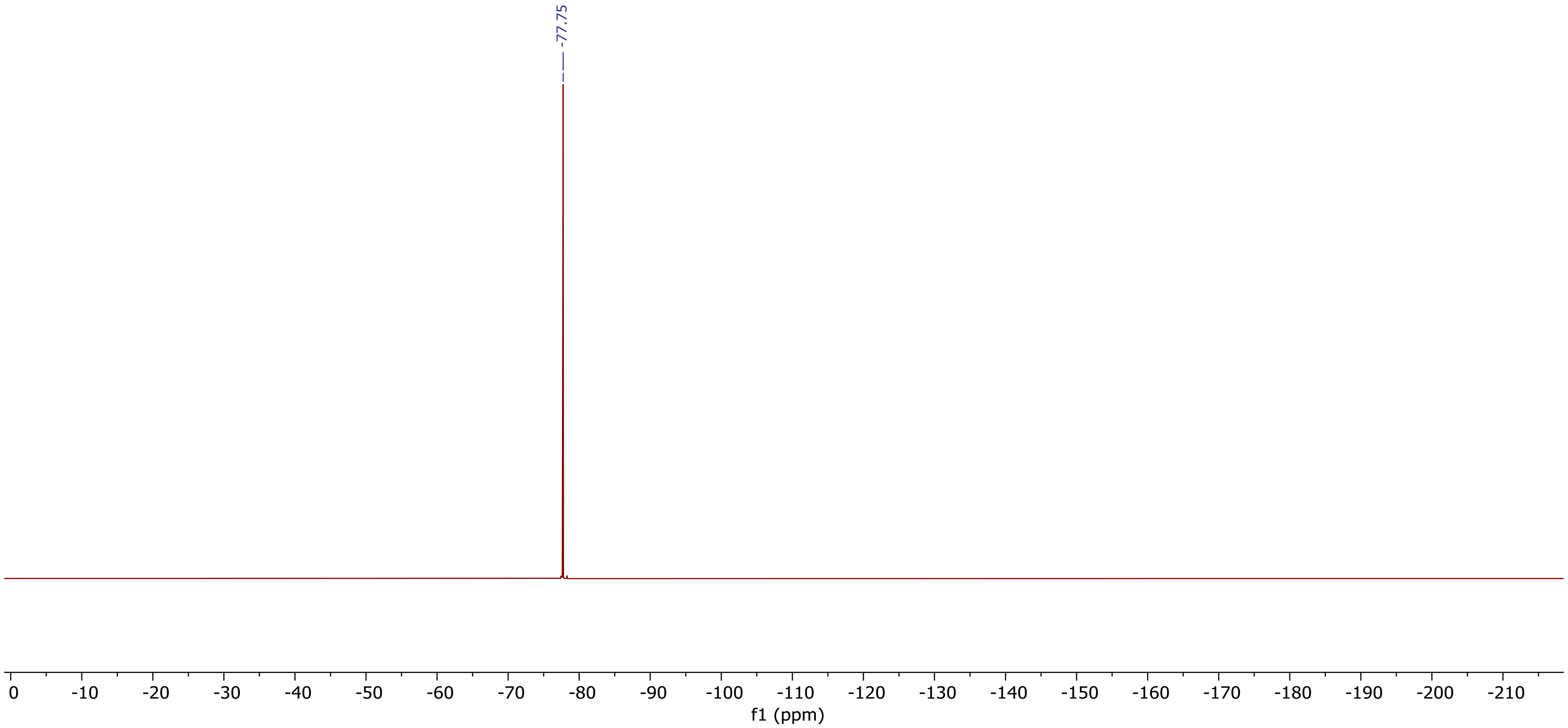

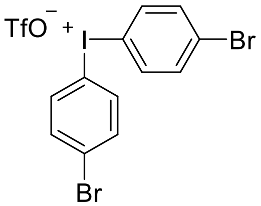

1e, ^{1}H NMR
400 MHz, DMSO-d_6

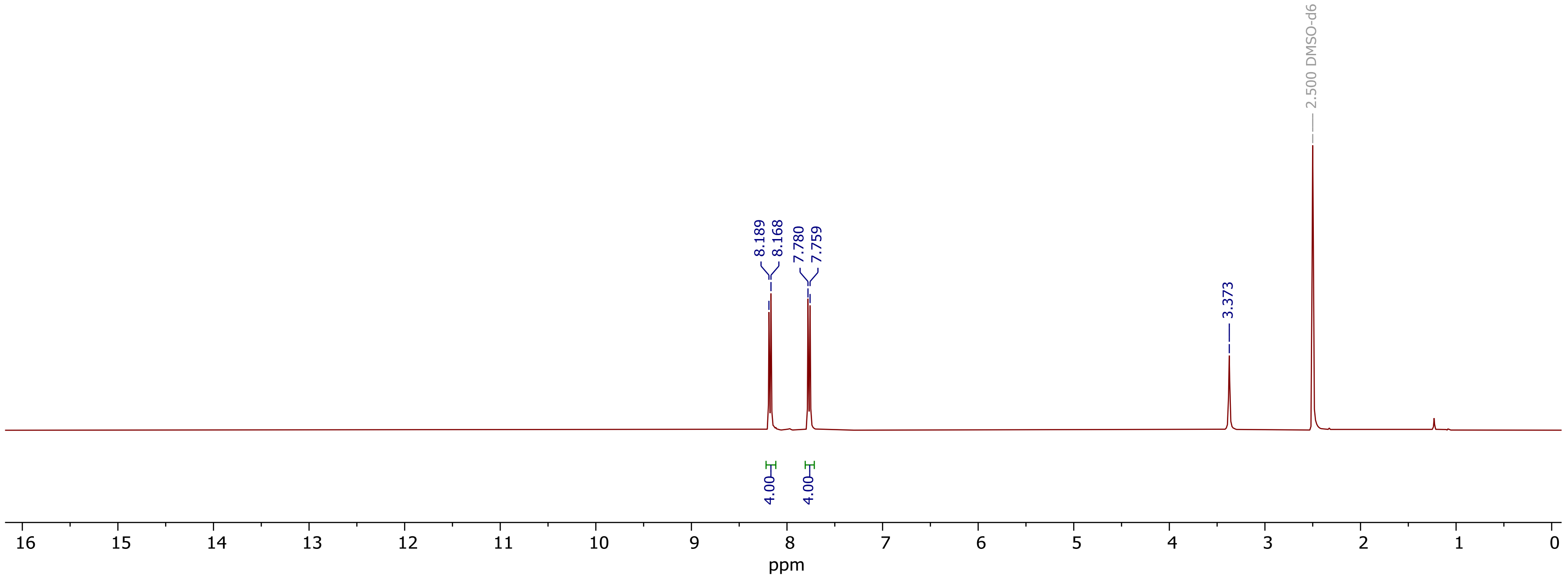

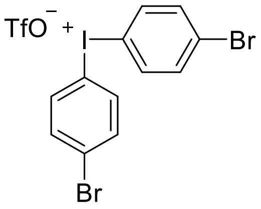

1e, ^{13}C NMR
100 MHz, DMSO-d_6

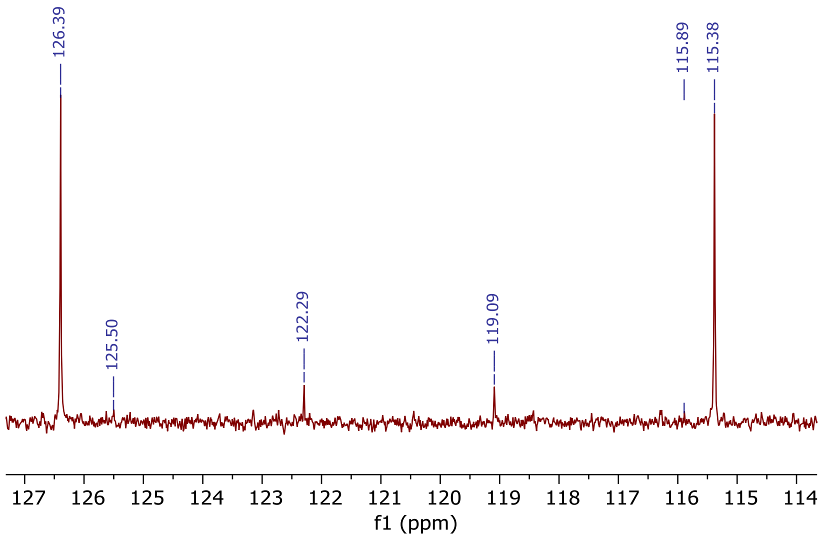

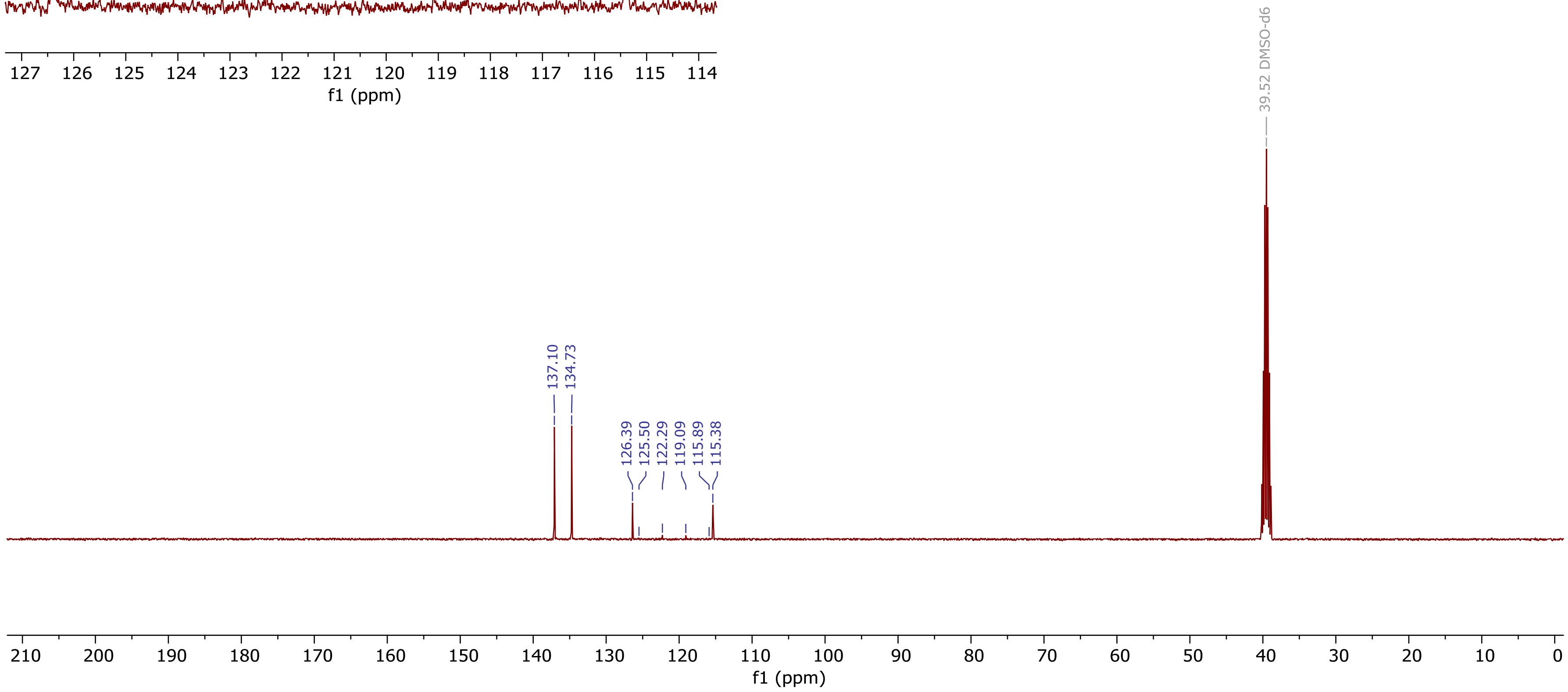

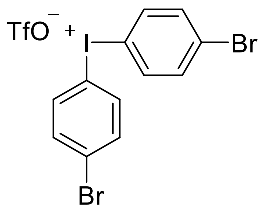

1e, ^{19}F NMR
376 MHz, DMSO-d_6

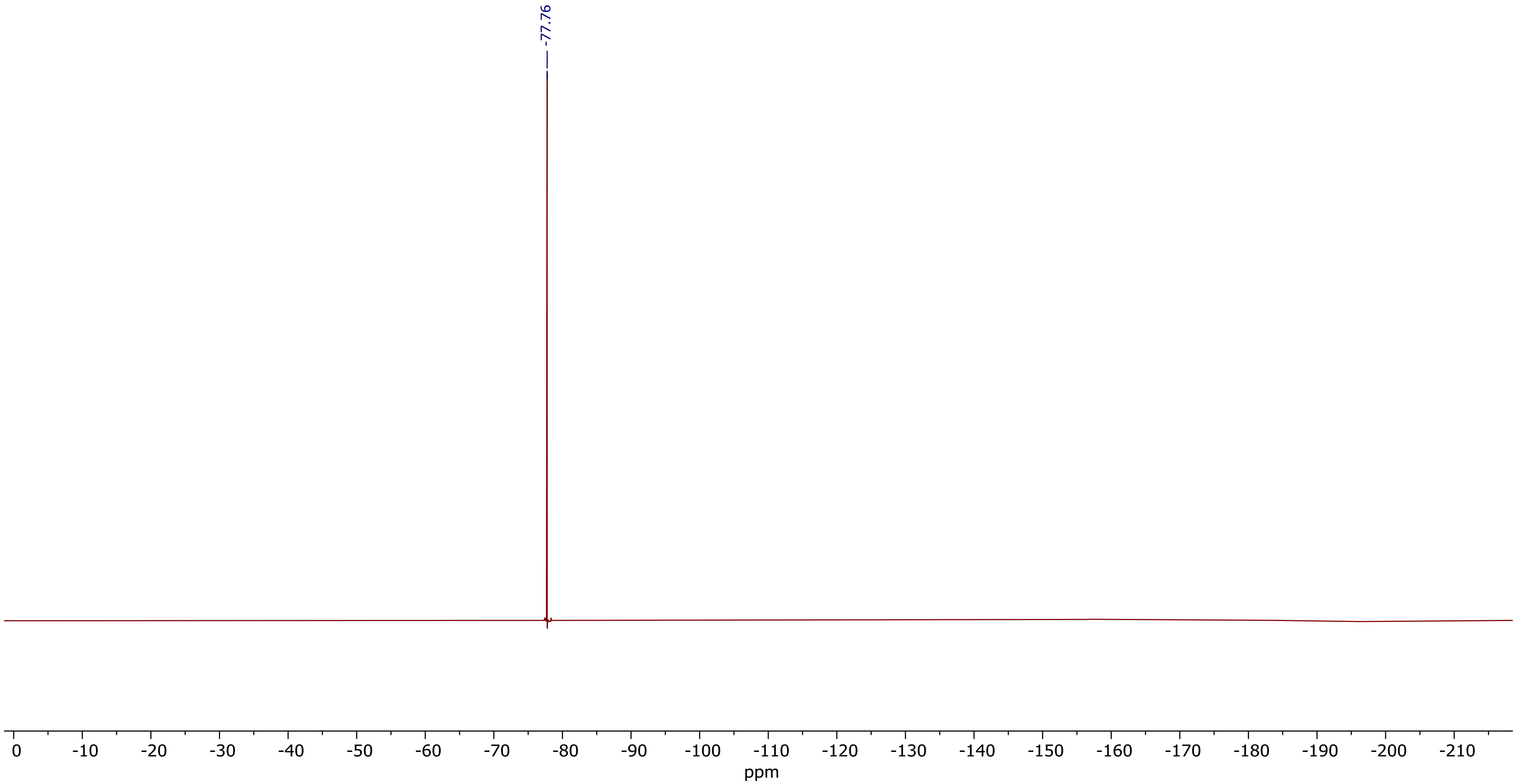

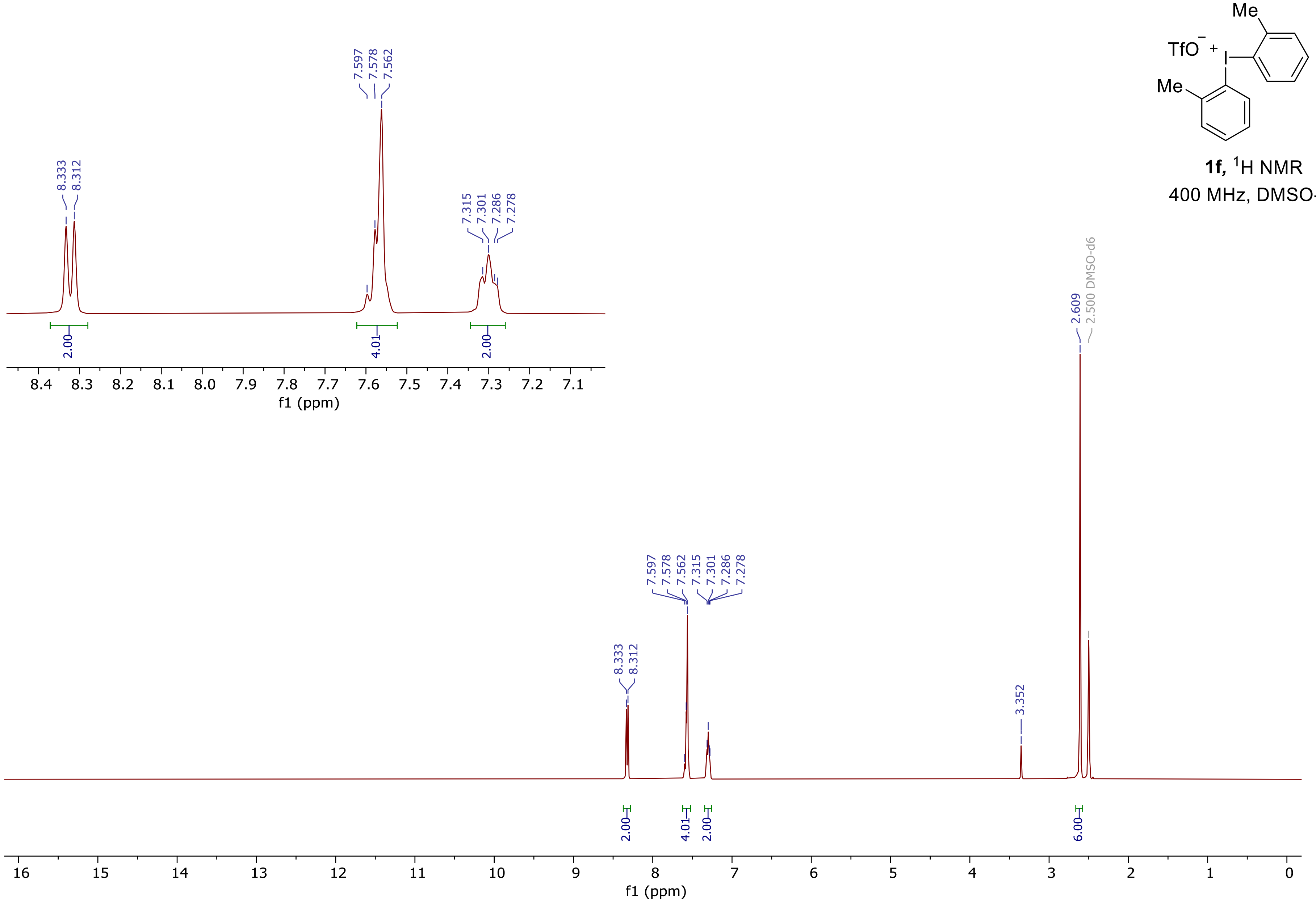

1f, ^{1}H NMR
400 MHz, DMSO-d_6

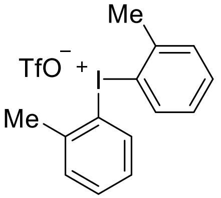

1f, ^{13}C NMR
100 MHz, DMSO-d_6

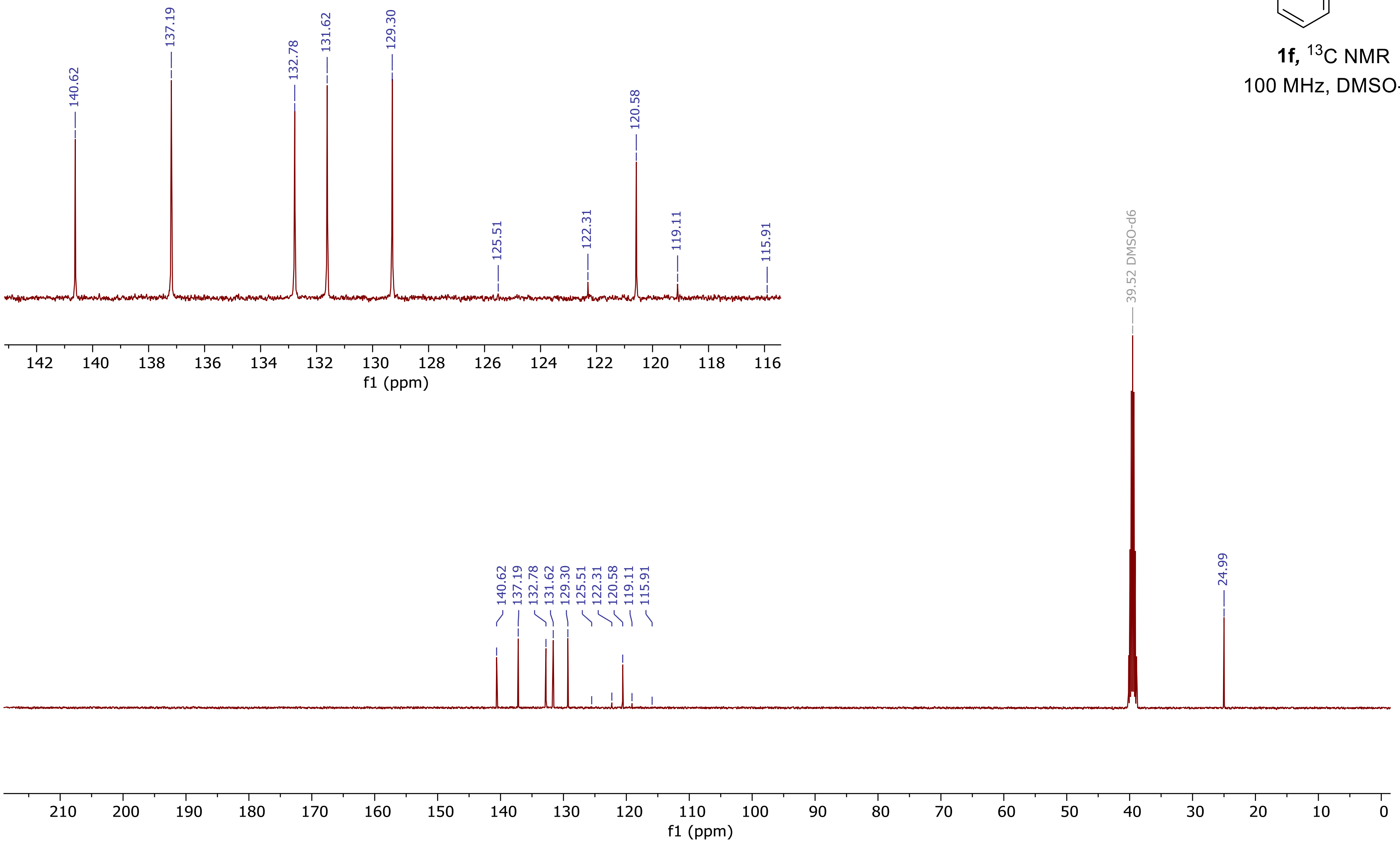

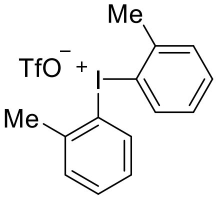

1f, ^{19}F NMR
376 MHz, DMSO-d_6

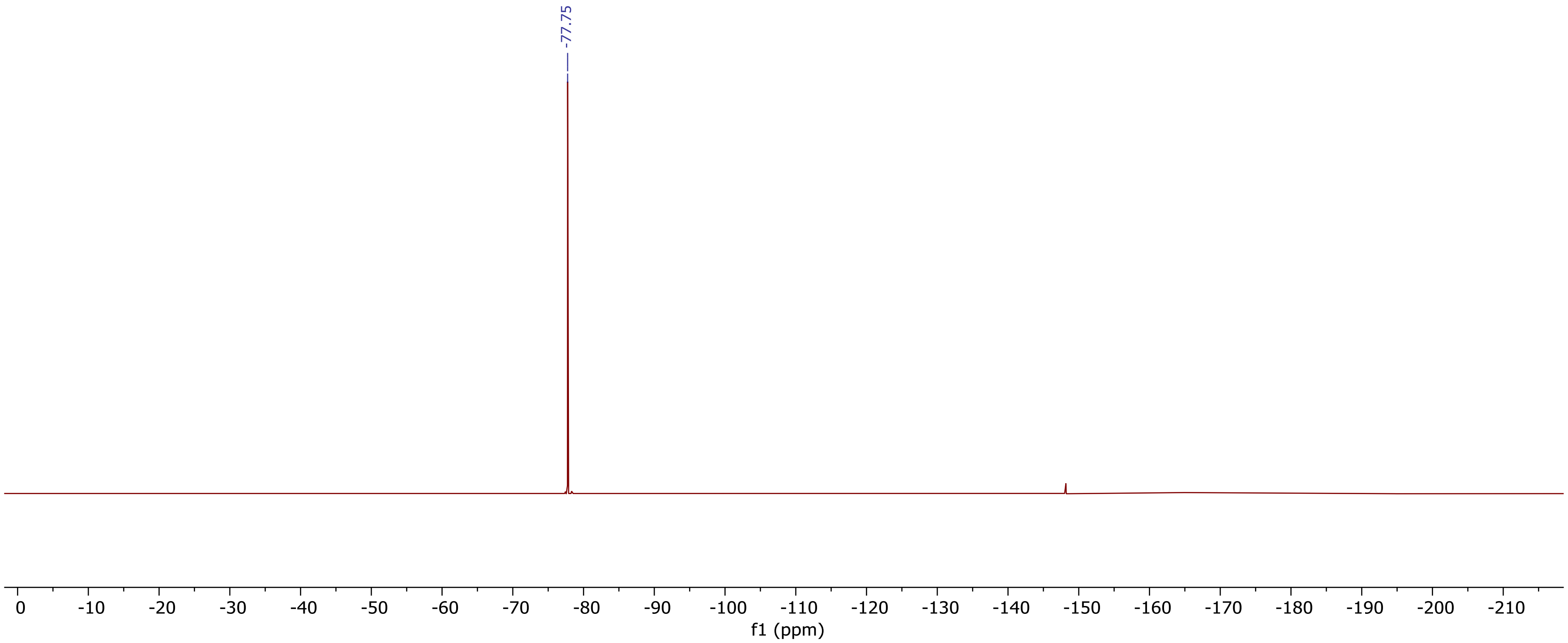

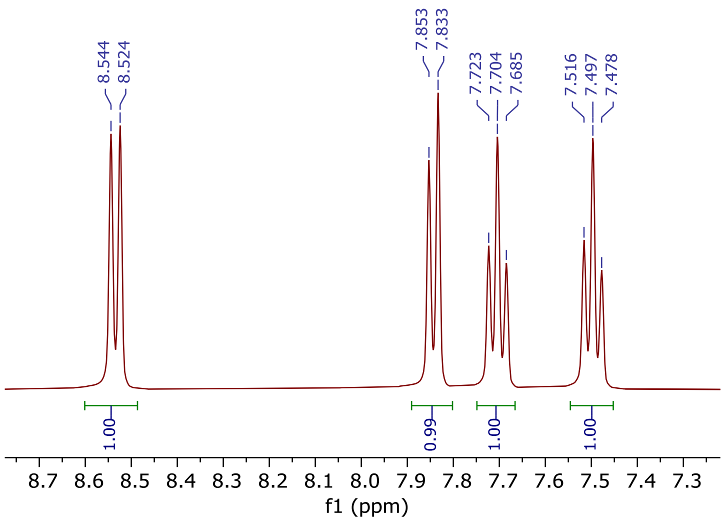

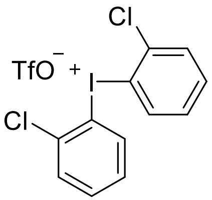

1g, ^{1}H NMR
400 MHz, DMSO-d_6

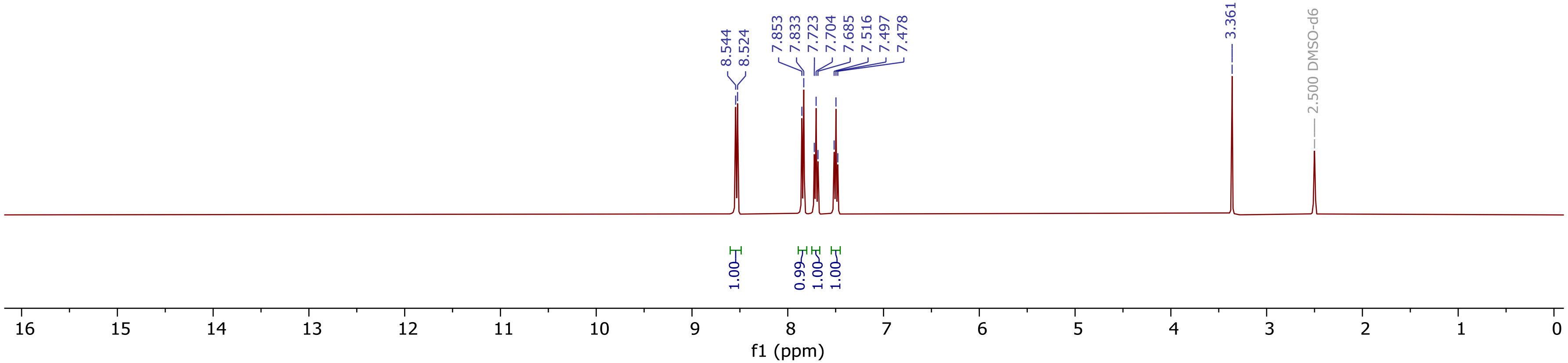

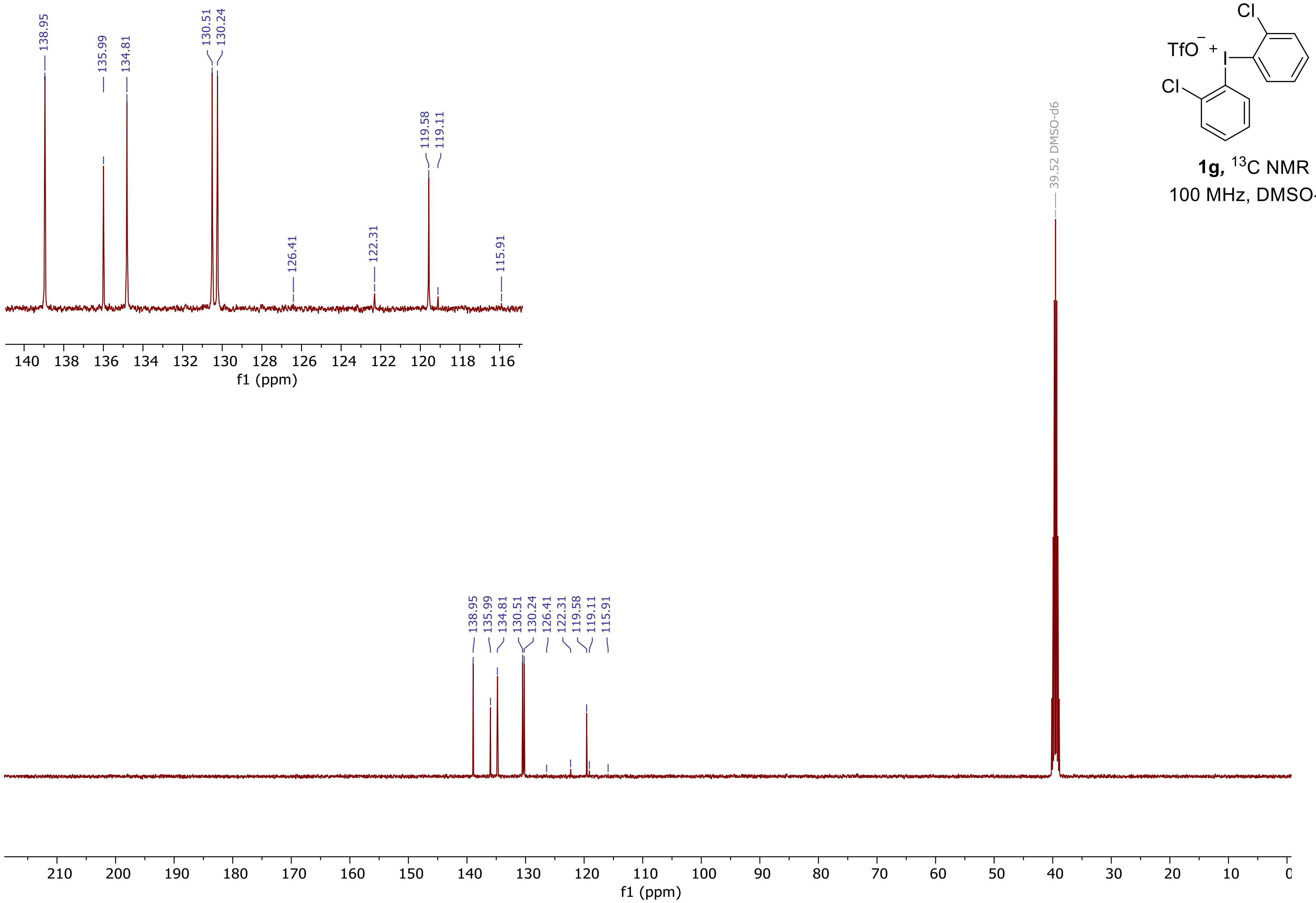

1g, ^{13}C NMR
100 MHz, DMSO-d_6

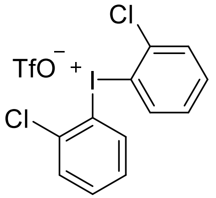

1g, ^{19}F NMR
376 MHz, DMSO-d_6

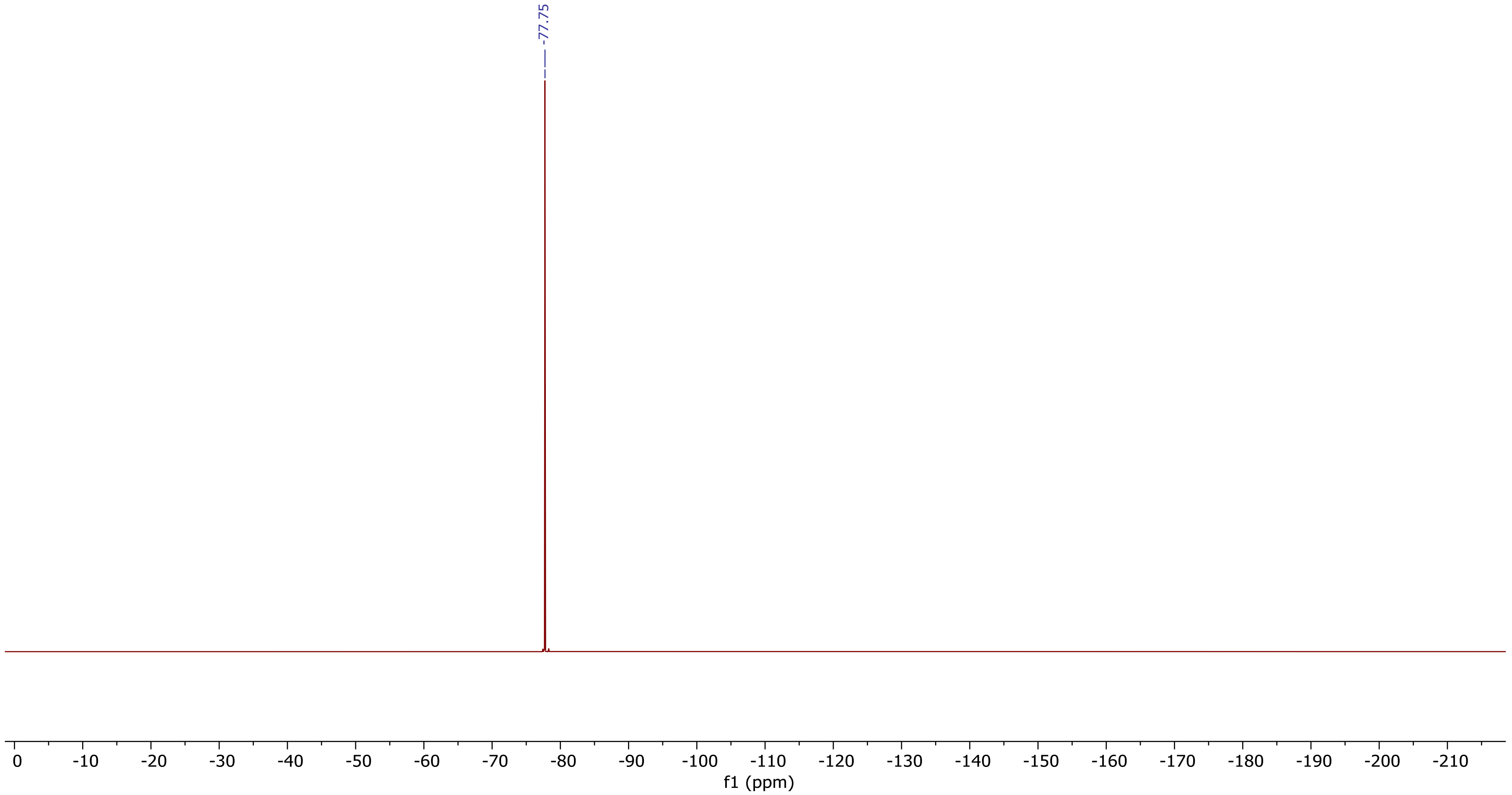

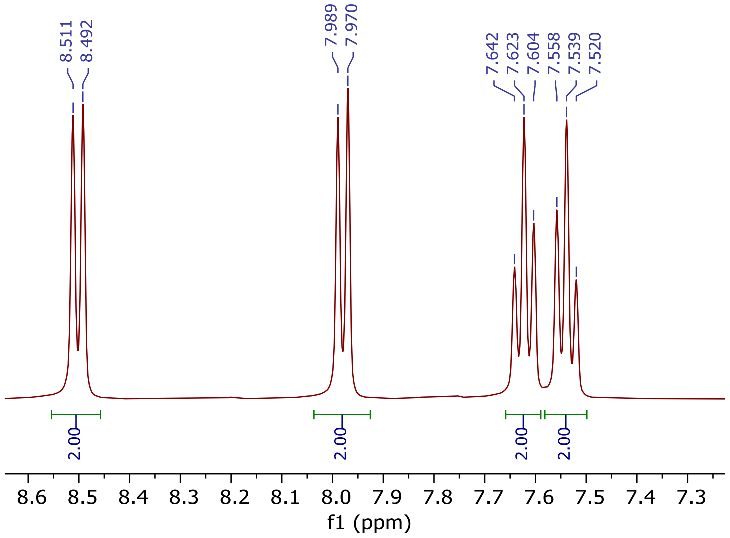

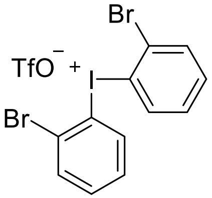

1h, ^{1}H NMR
400 MHz, DMSO-d_6

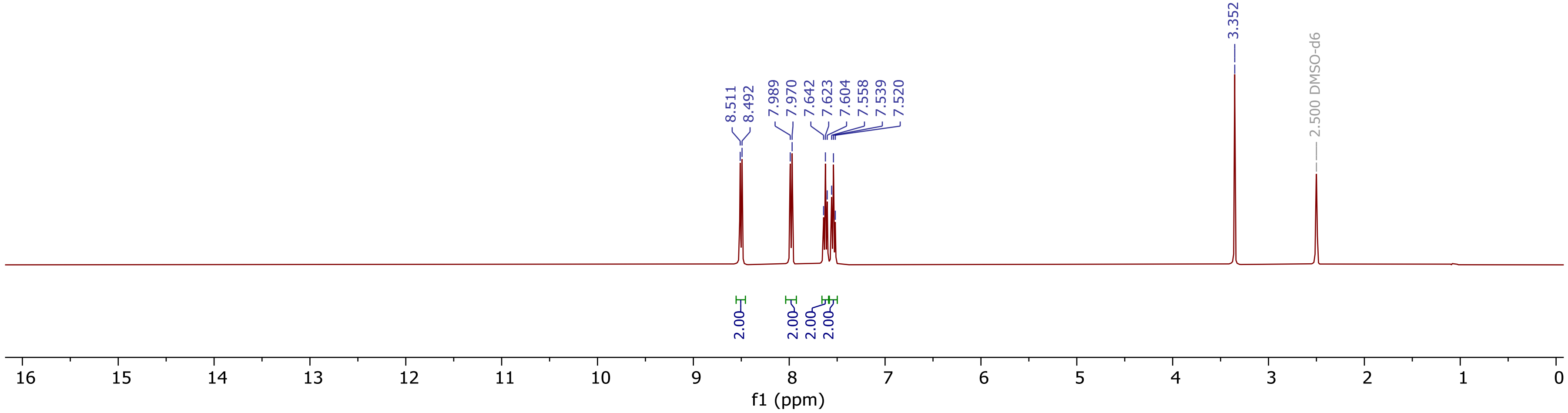

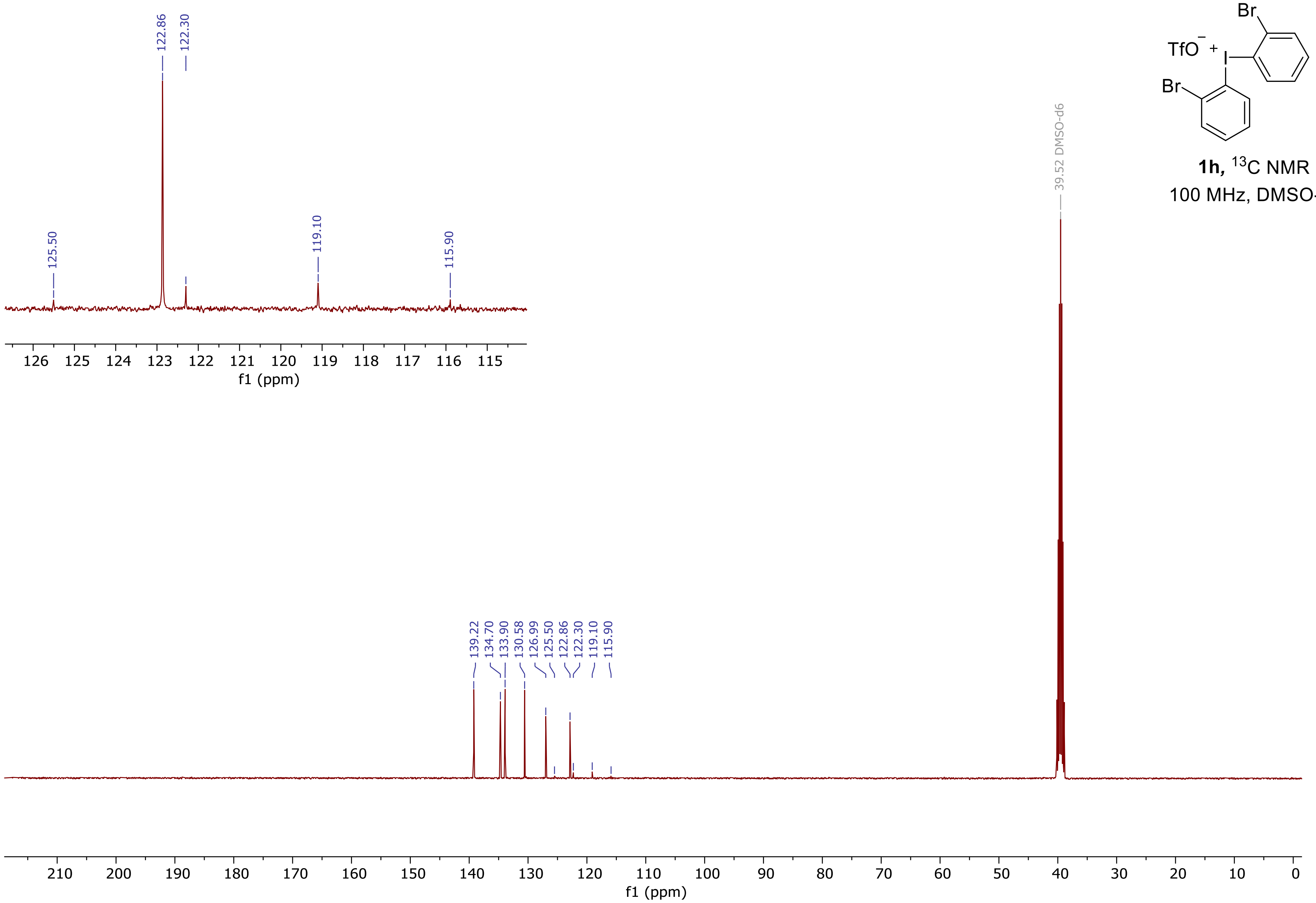

1h, ^{13}C NMR
100 MHz, DMSO-d_6

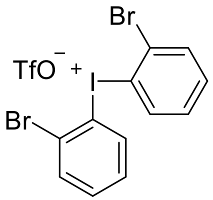

1h, ^{19}F NMR
376 MHz, DMSO-d_6

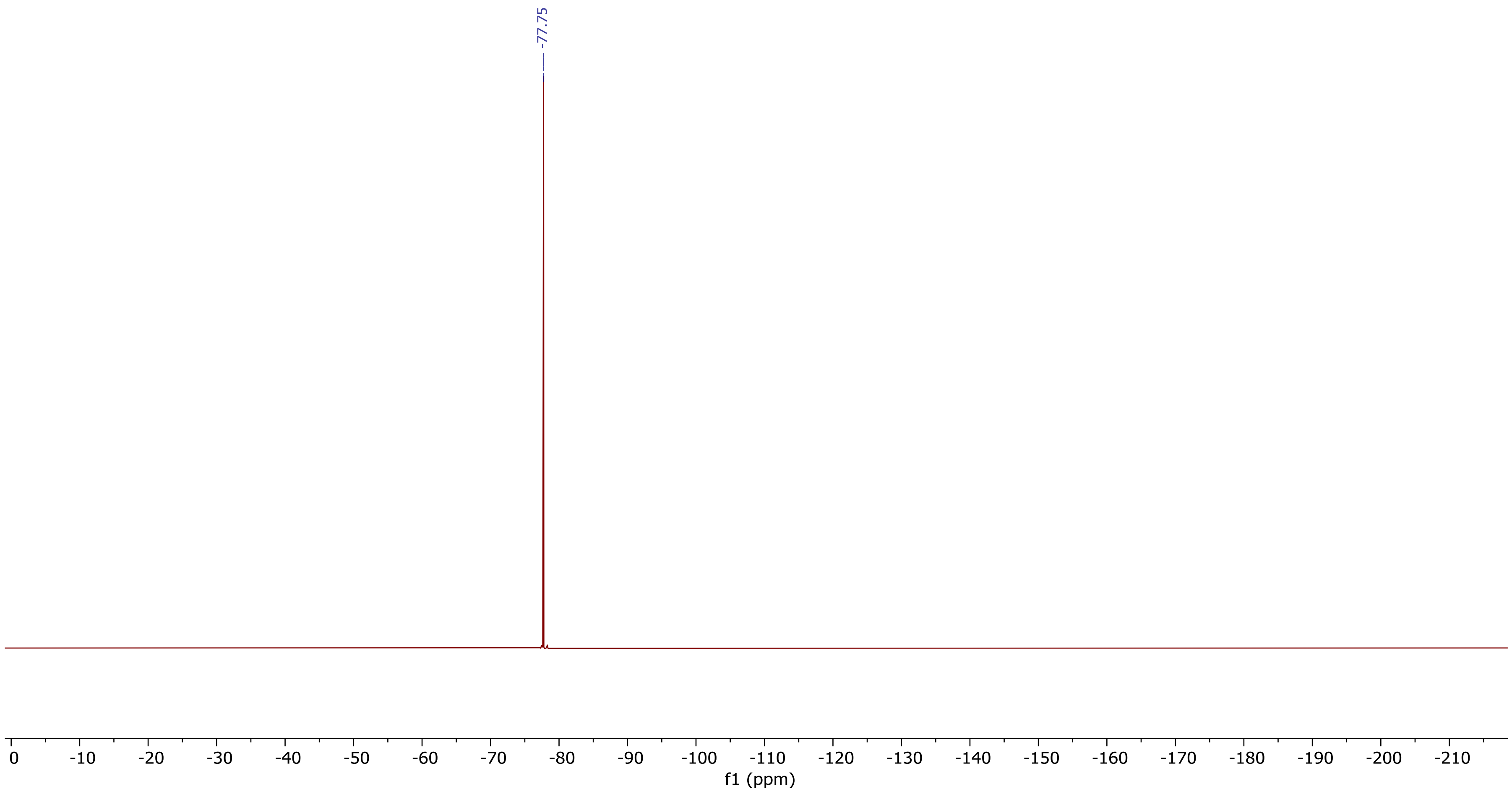

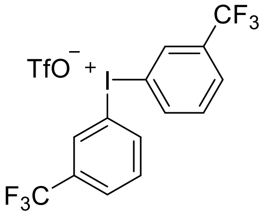

1i, ^{1}H NMR
400 MHz, DMSO-d_6

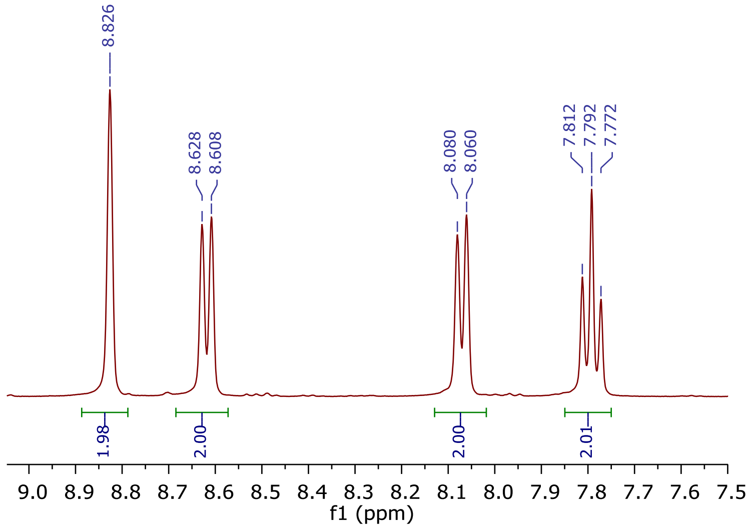

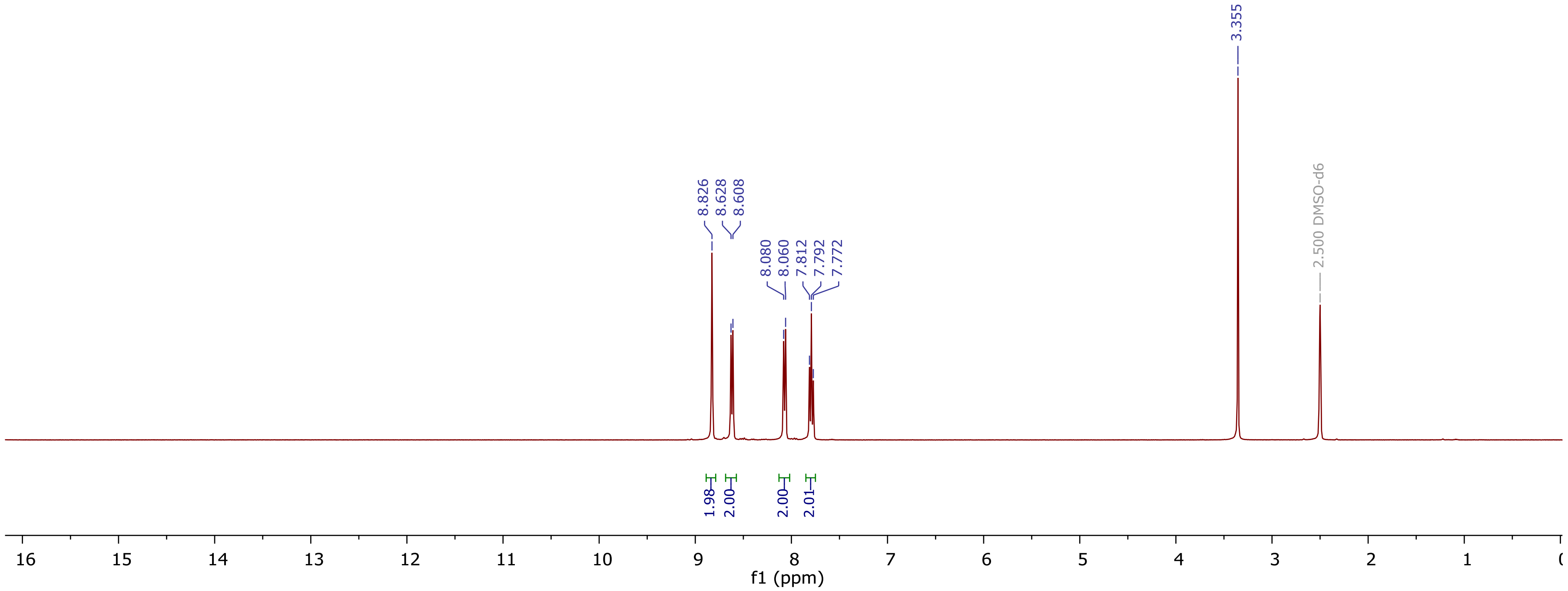

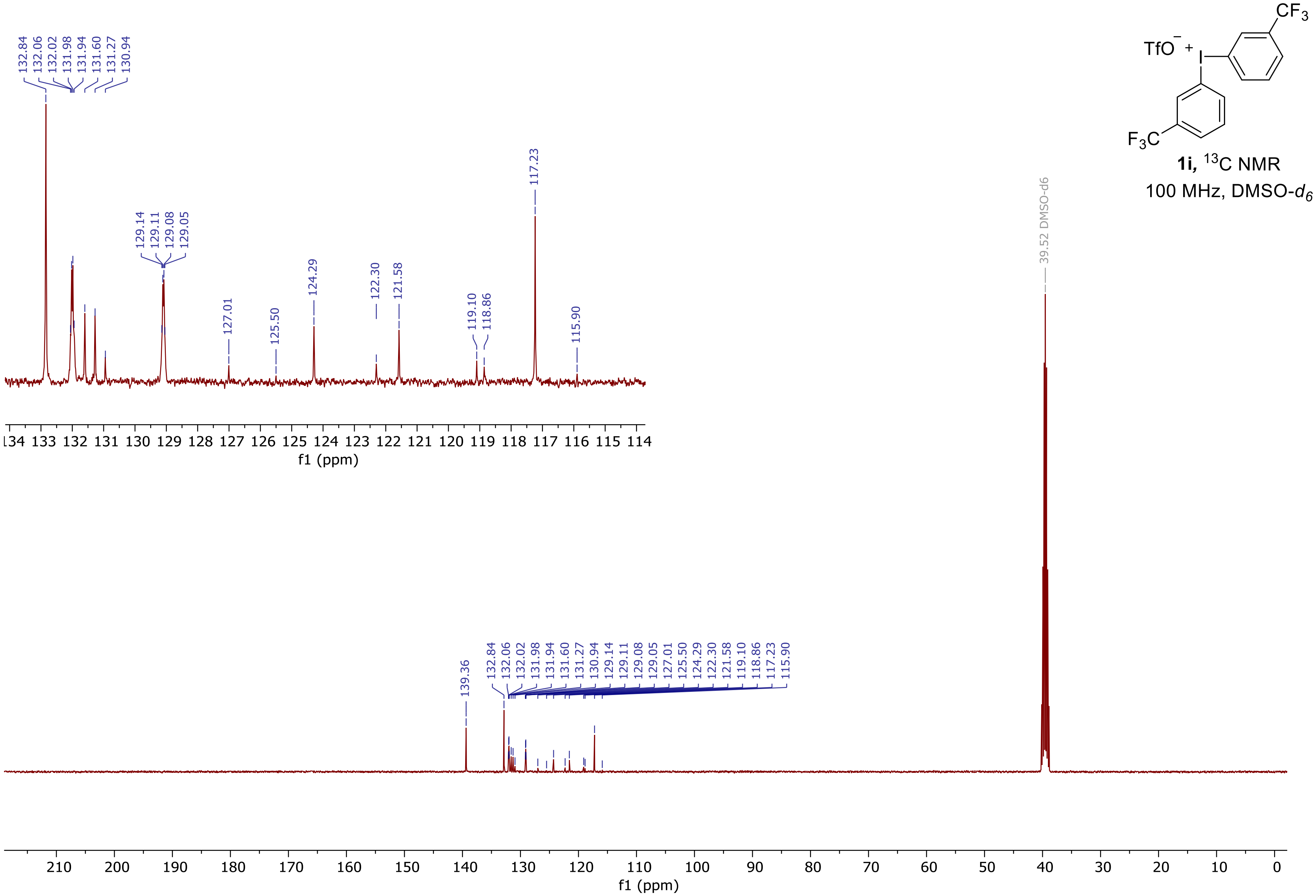

1i, ^{13}C NMR
100 MHz, DMSO-d_6

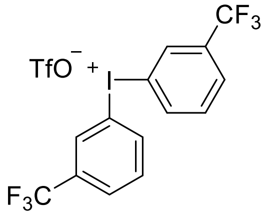

1i, ^{19}F NMR
376 MHz, DMSO-d_6

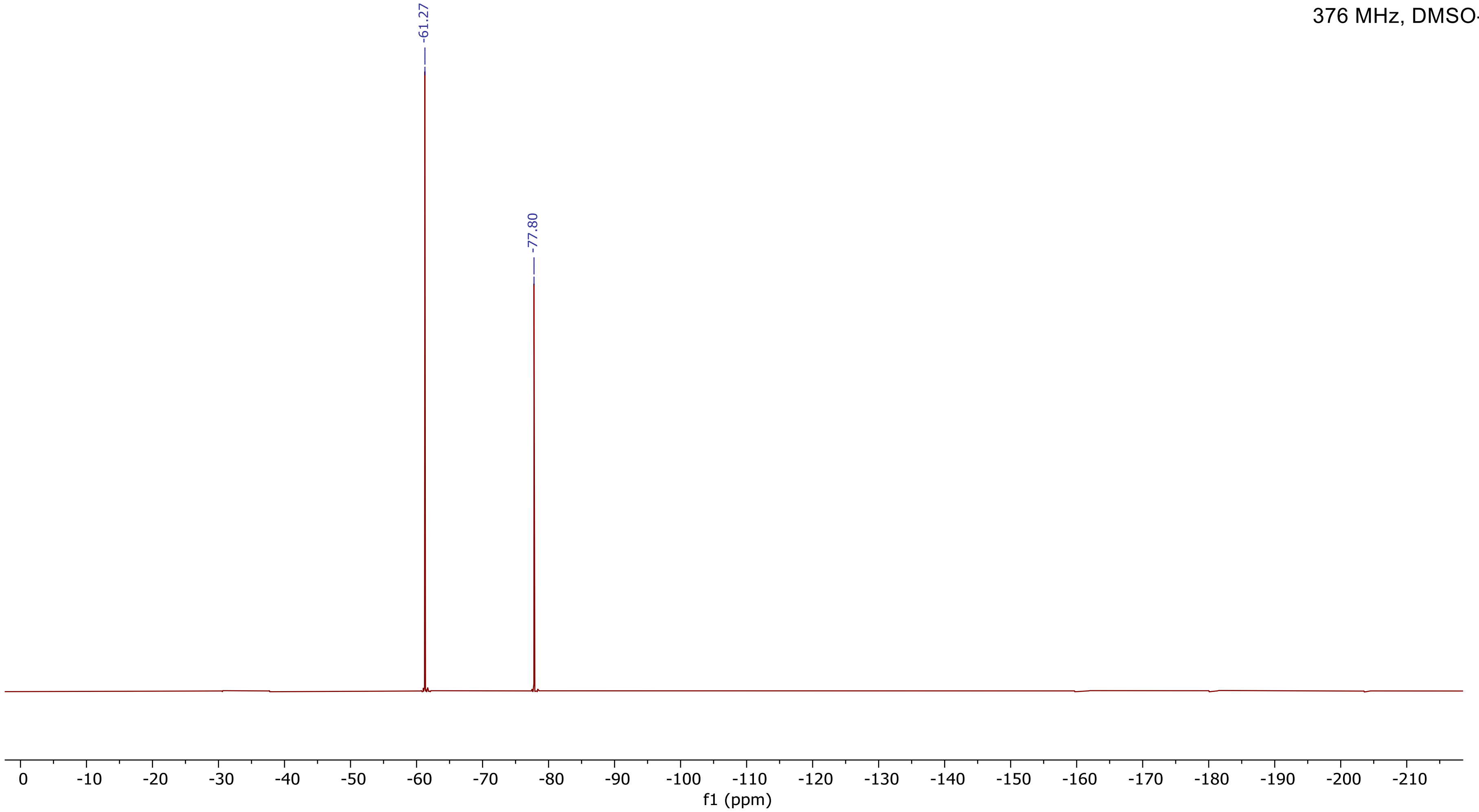

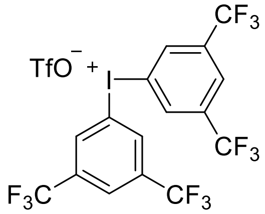

1j, ^{1}H NMR
400 MHz, Acetone-d_6

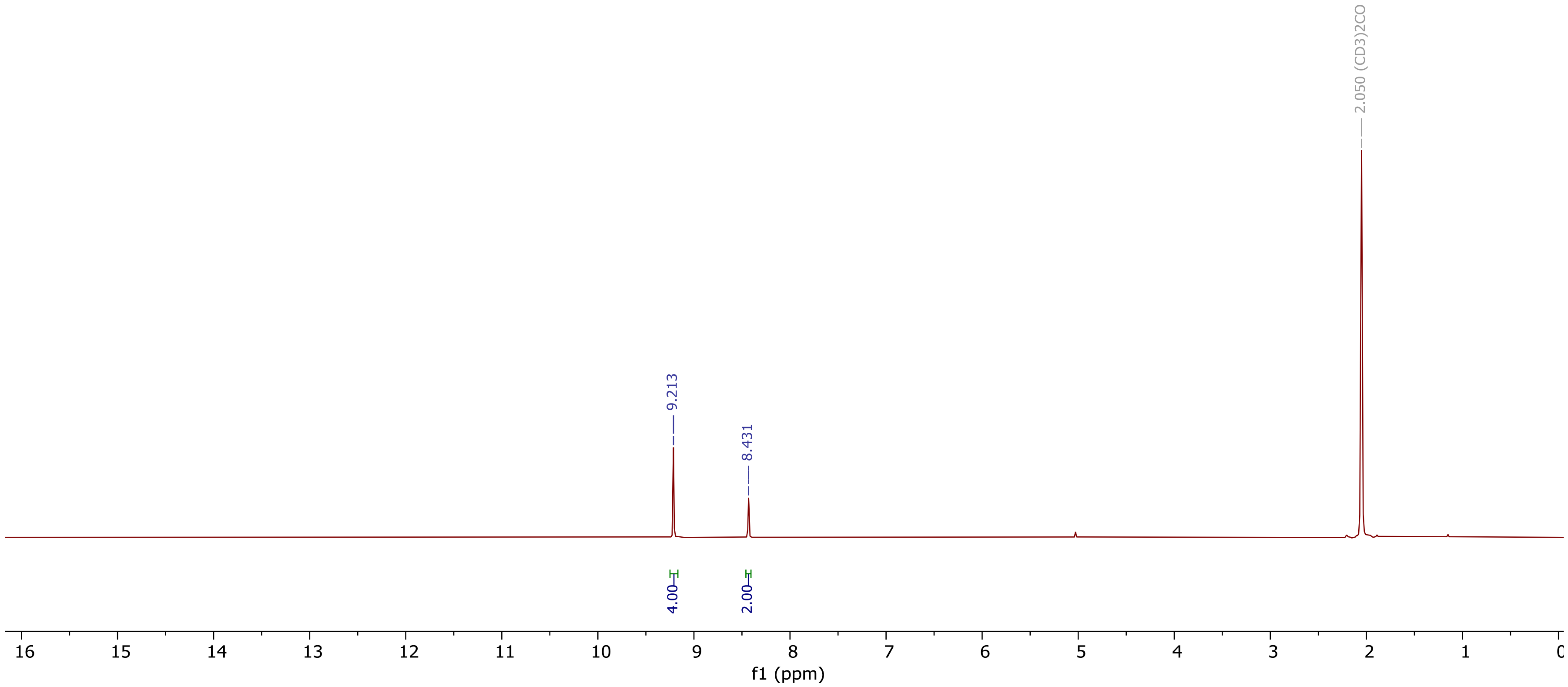

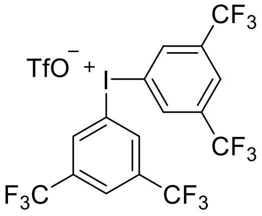

1j, ^{13}C NMR
100 MHz, Acetone-d_6

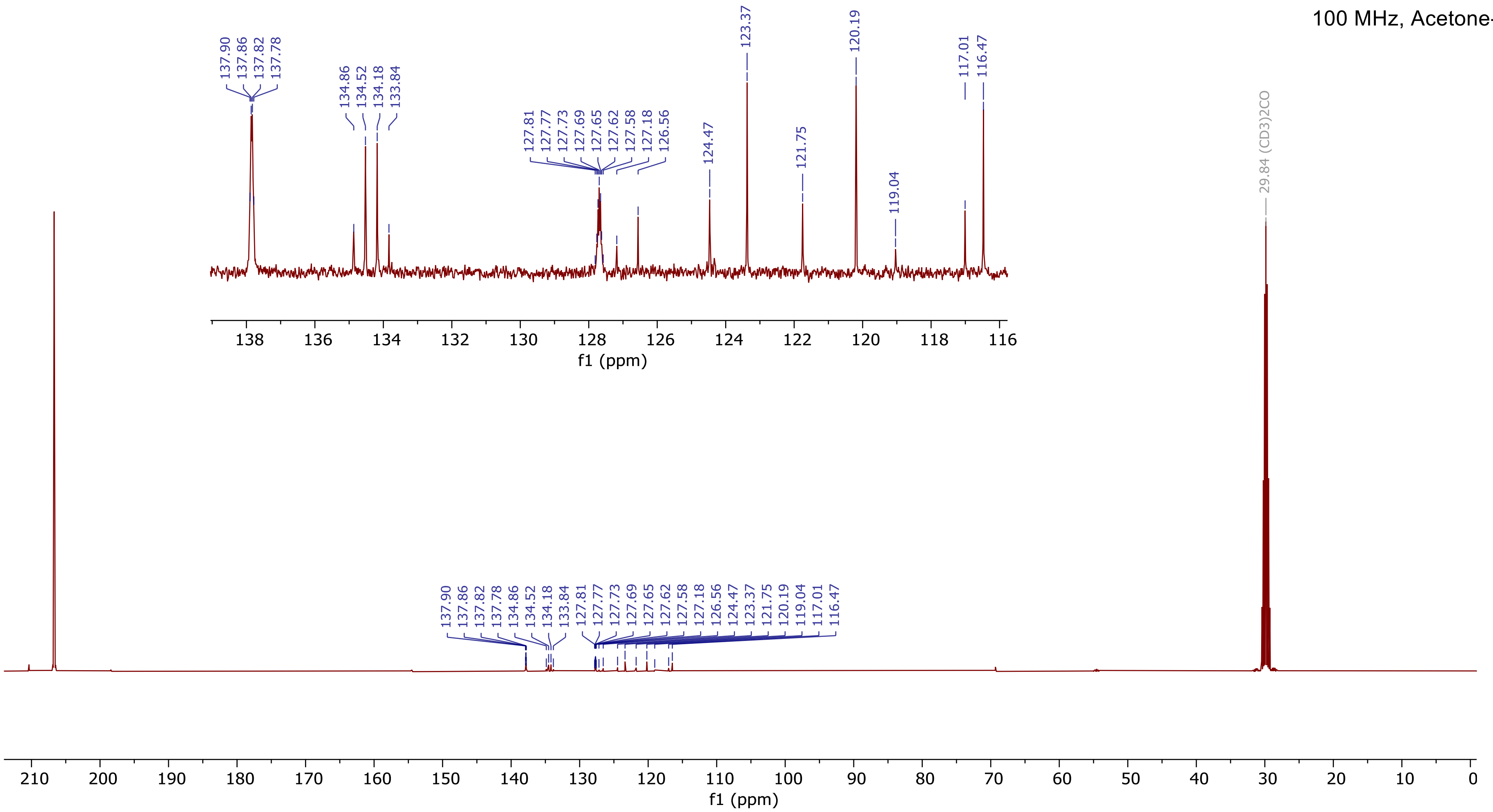

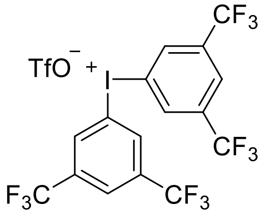

1j, ^{19}F NMR
376 MHz, Acetone-d_6

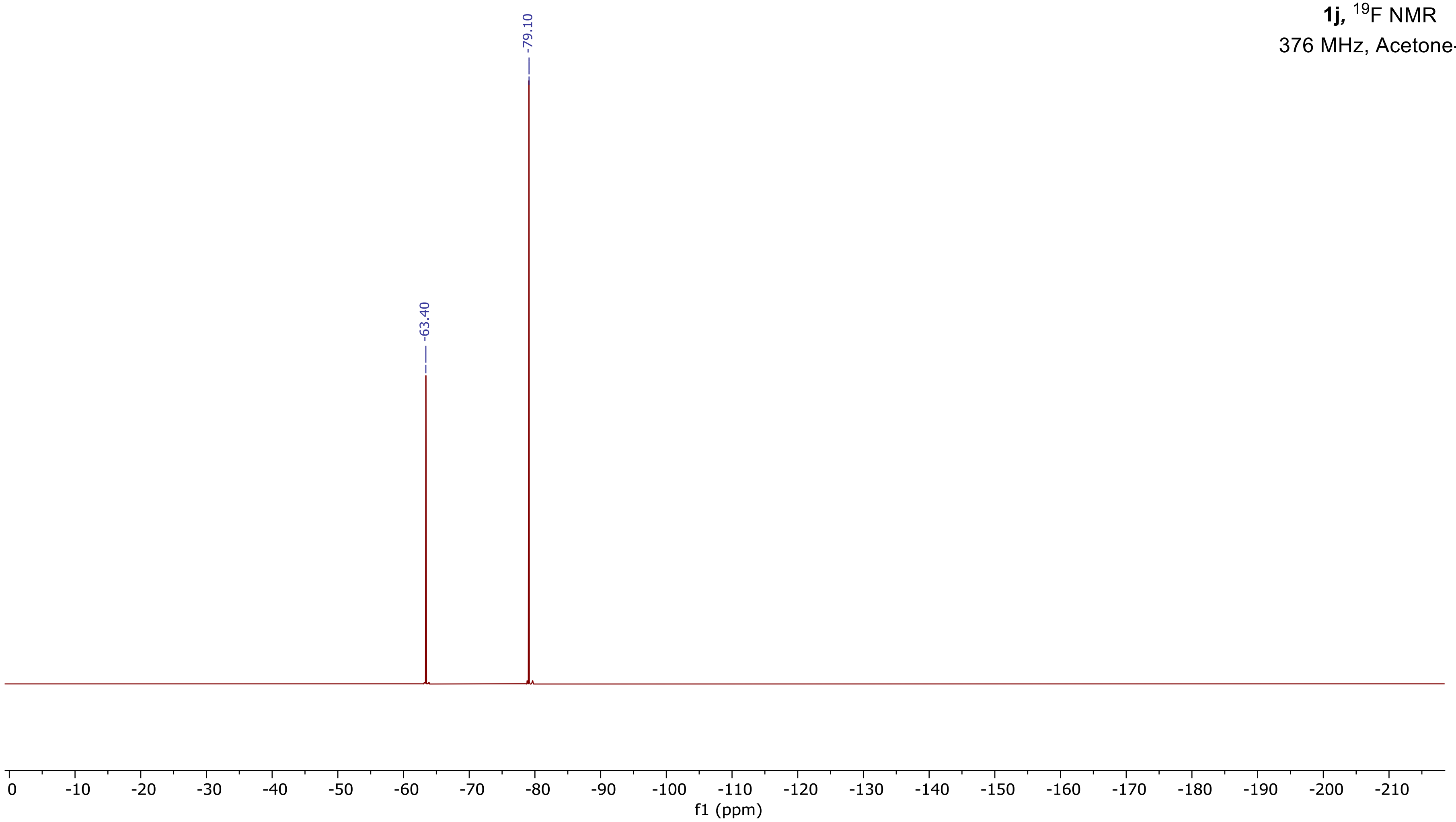

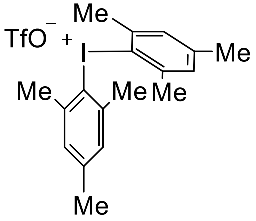

1k, ^{1}H NMR
400 MHz, DMSO-d_6

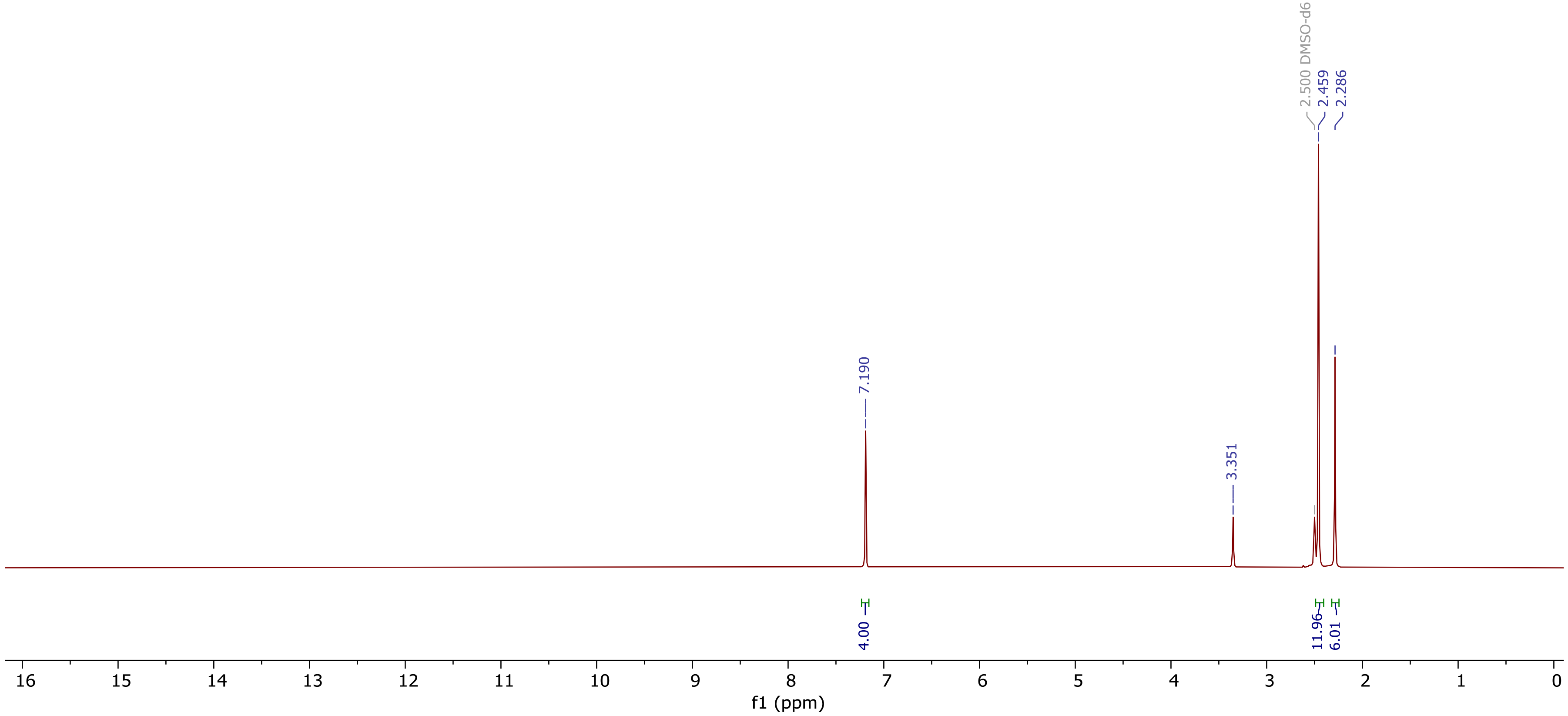

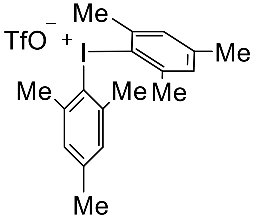

1k, ^{13}C NMR
100 MHz, DMSO-d_6

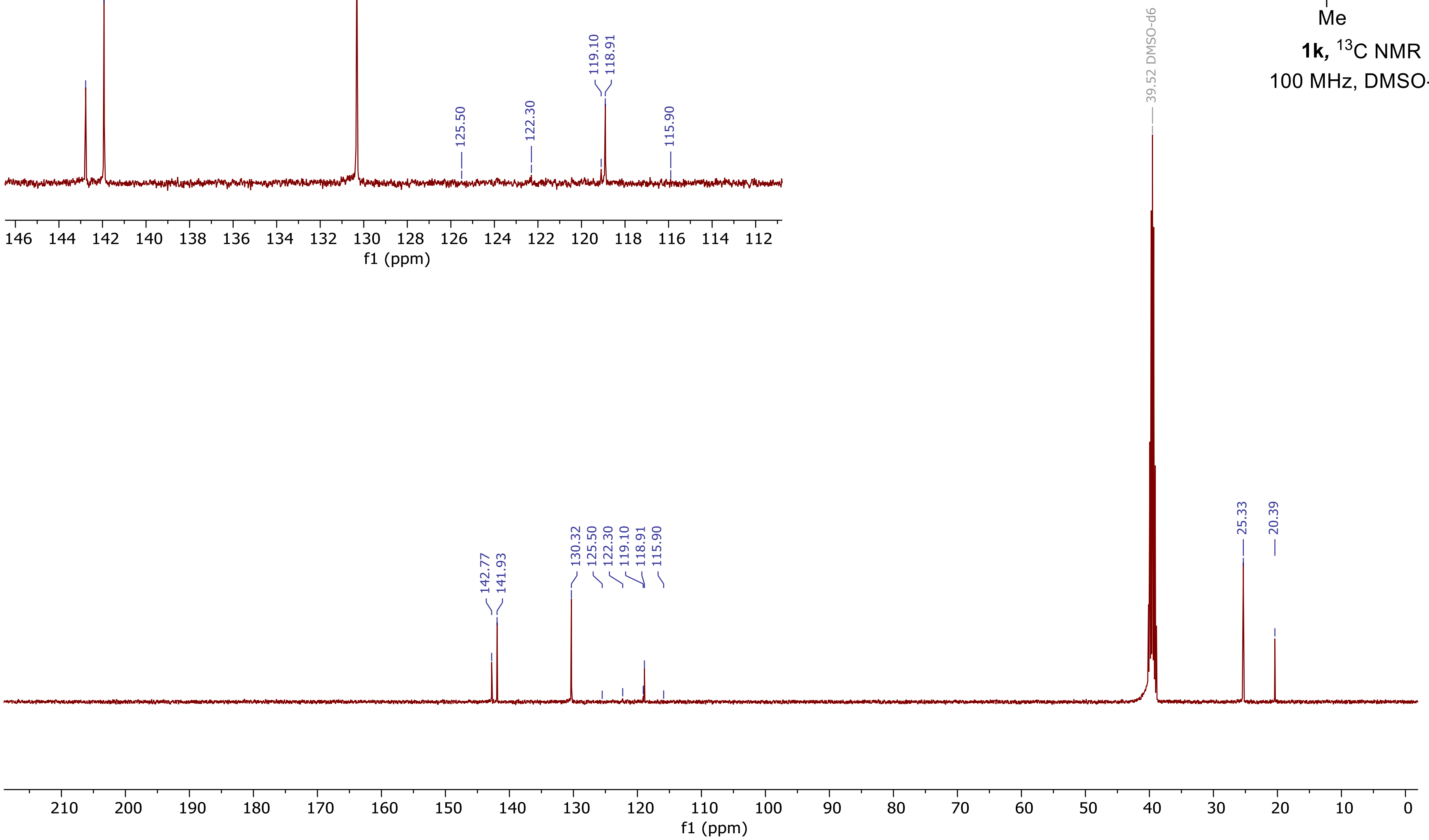

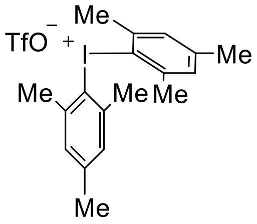

1k, ^{19}F NMR
376 MHz, DMSO-d_6

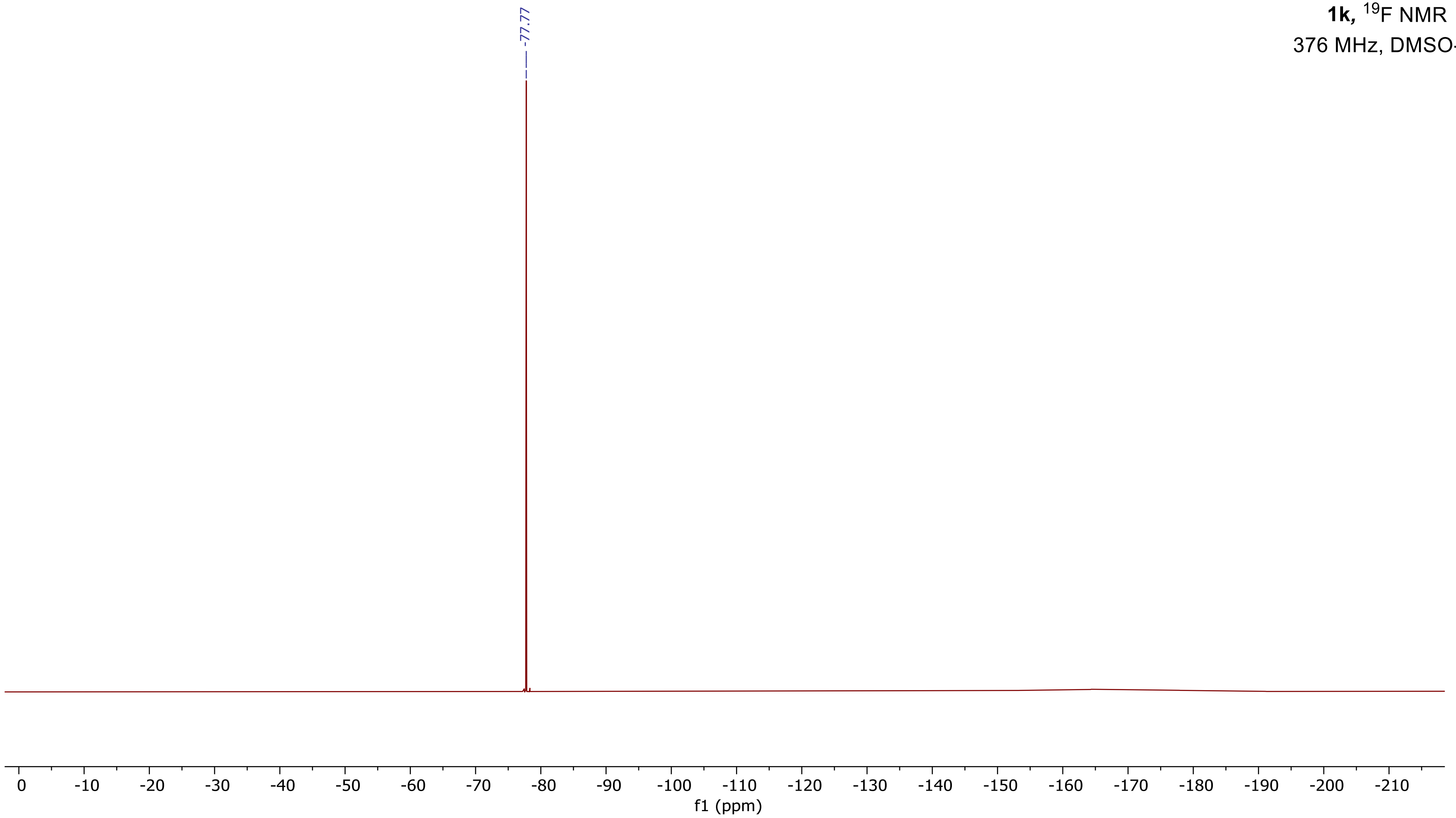

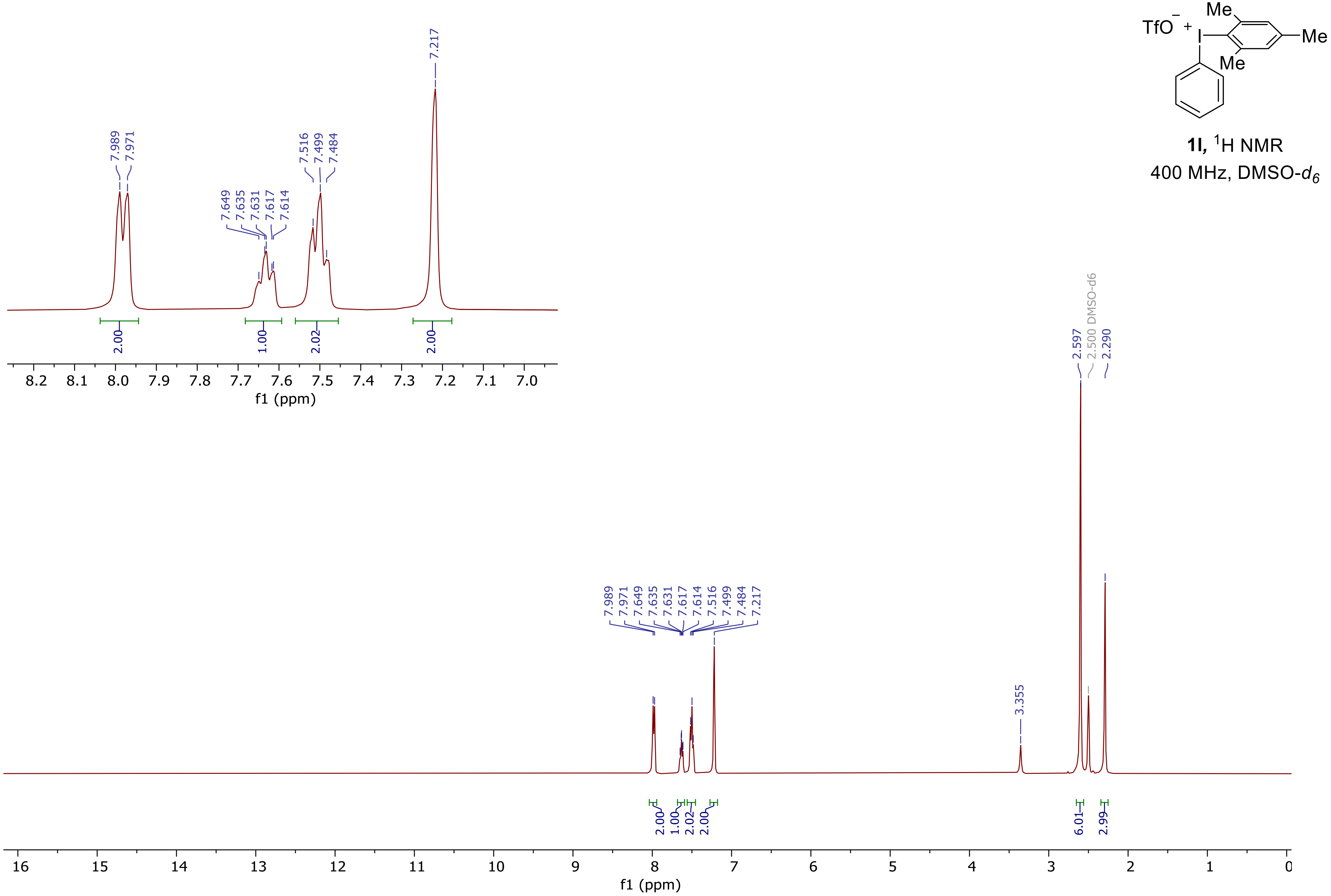
TfO⁻ +I Me Me Me
1l, ¹H NMR
400 MHz, DMSO-d6
7.989 7.971 7.649 7.635 7.631 7.617 7.614 7.516 7.499 7.484 7.217
2.00 1.00 2.02 2.00
f1 (ppm)
3.355
2.597 2.500 DMSO-d6 2.290
6.01 2.99
f1 (ppm)

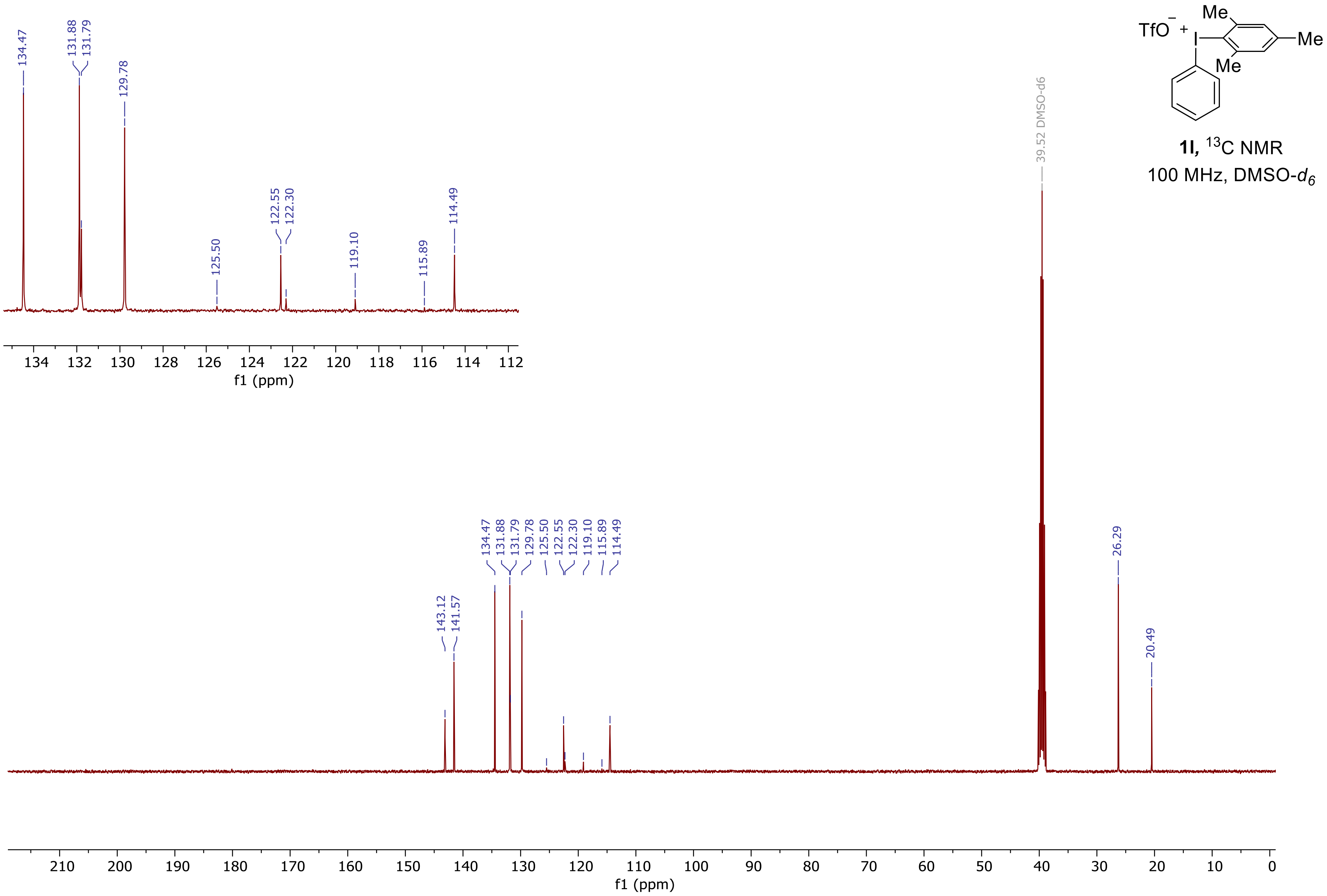

1l, ^{13}C NMR
100 MHz, DMSO-d_6

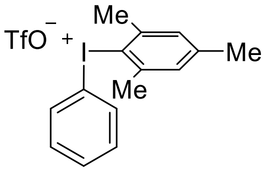

1l, ^{19}F NMR
376 MHz, DMSO-d_6

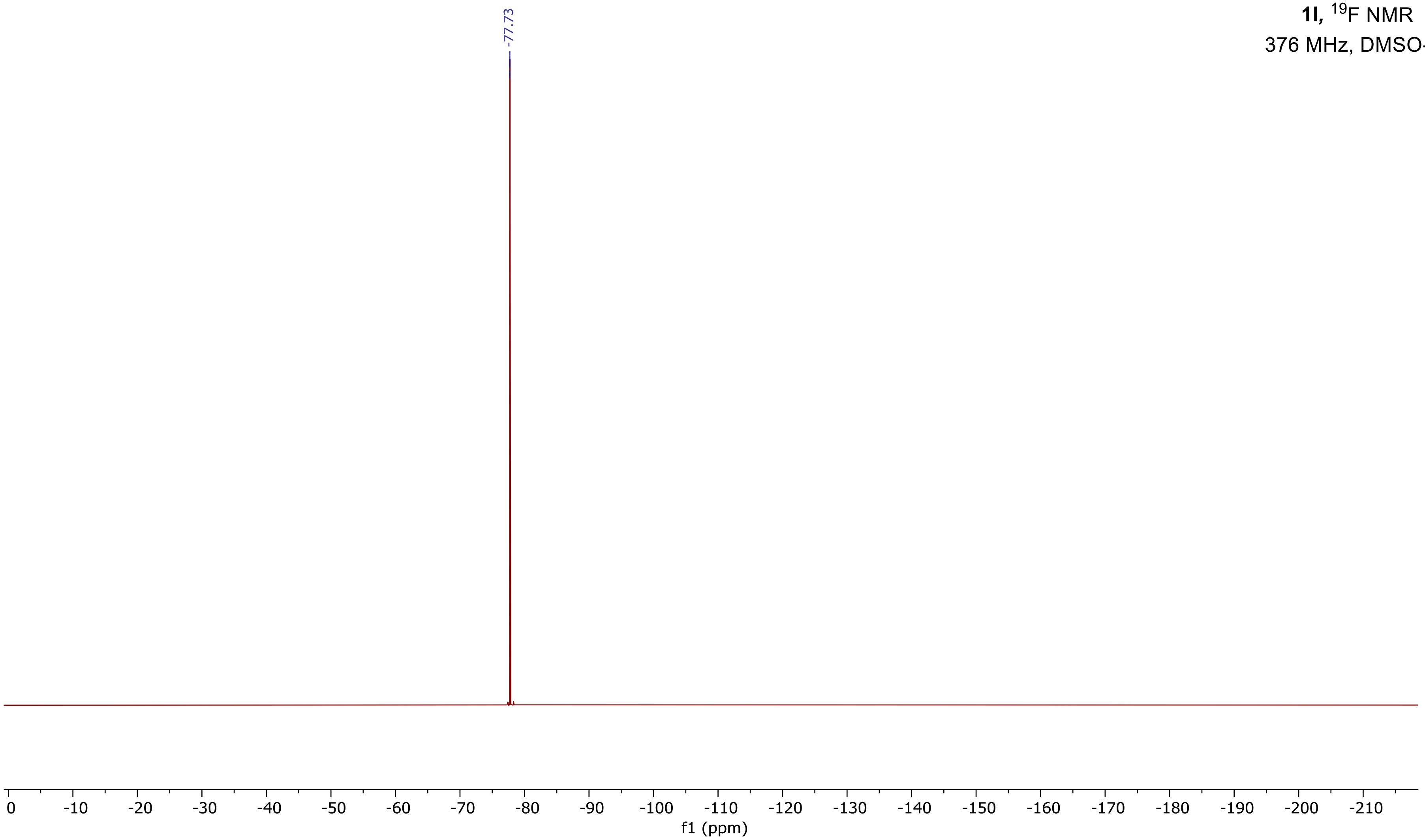

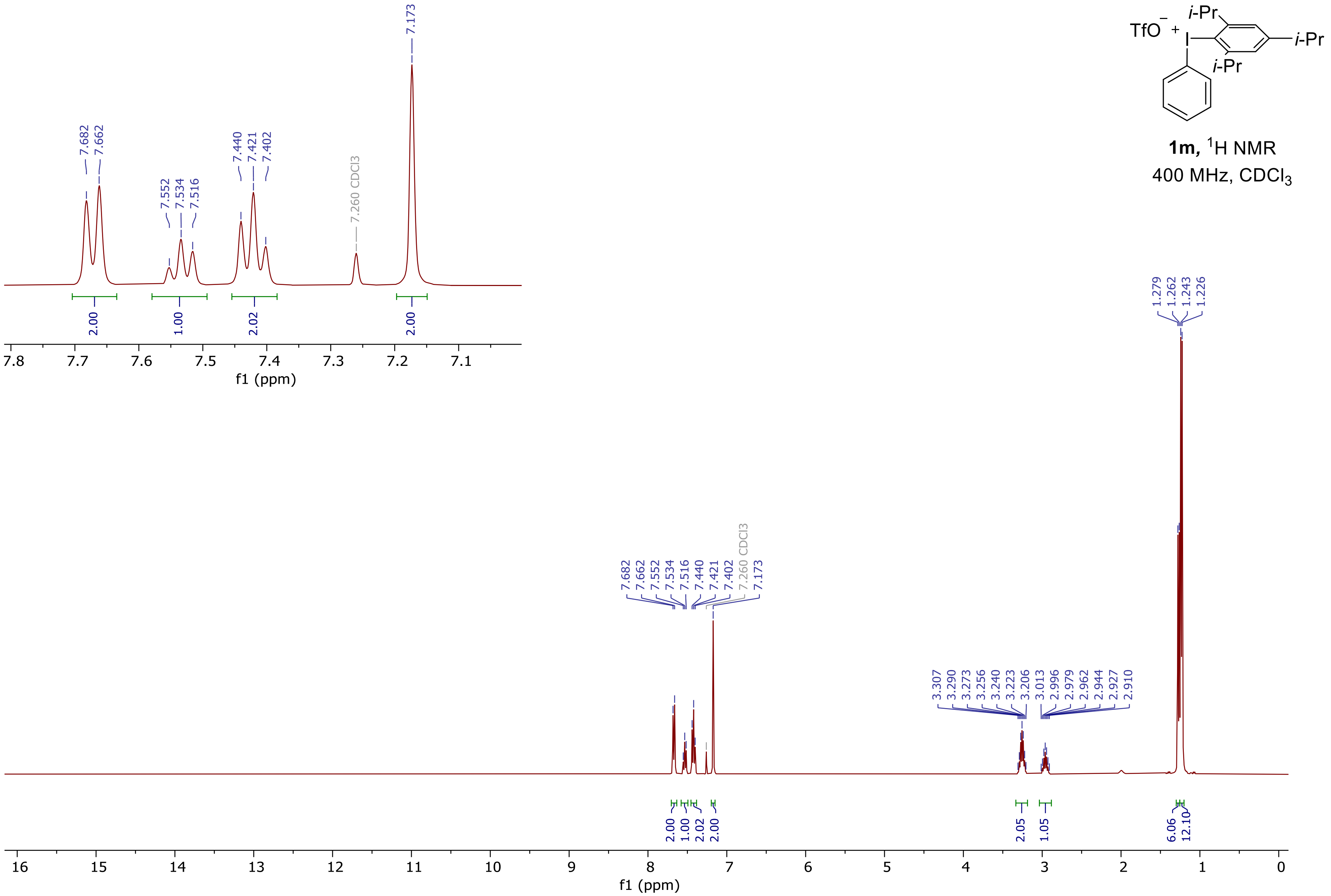
TfO⁻ ⁺I
i-Pr
i-Pr
i-Pr
1m, ¹H NMR
400 MHz, CDCl3
7.682 7.662 7.552 7.534 7.516 7.440 7.421 7.402 7.260 CDCl3 7.173
2.00 1.00 2.02 2.00
f1 (ppm)
3.307 3.290 3.273 3.256 3.240 3.223 3.206 3.013 2.996 2.979 2.962 2.944 2.927 2.910
1.279 1.262 1.243 1.226
2.05 1.05 6.06 12.10

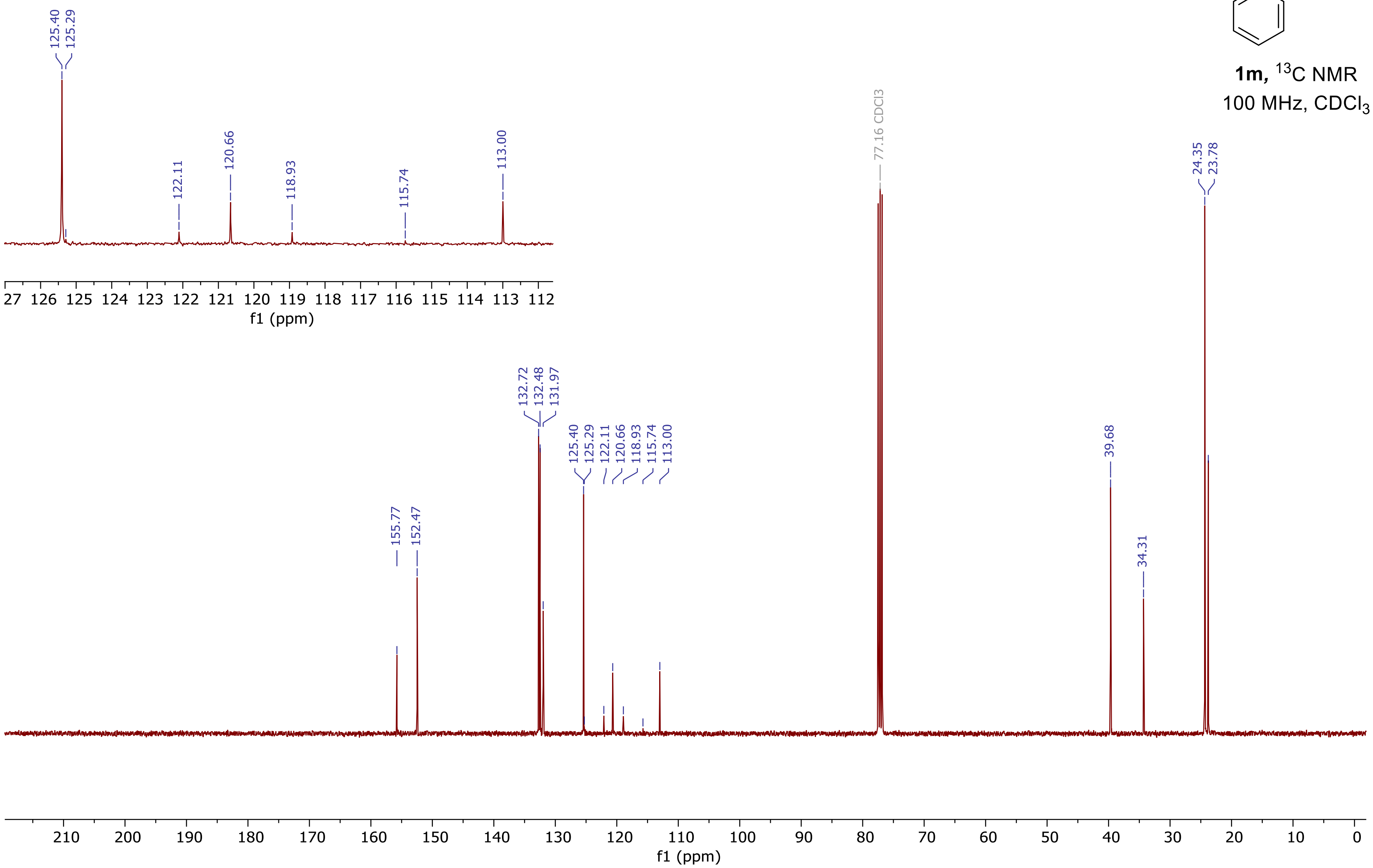

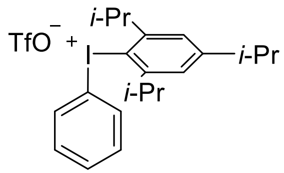

1m, ^{13}C NMR

100 MHz, $CDCl_3$

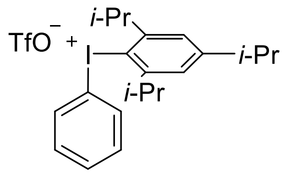

1m, ^{19}F NMR
376 MHz, $CDCl_3$

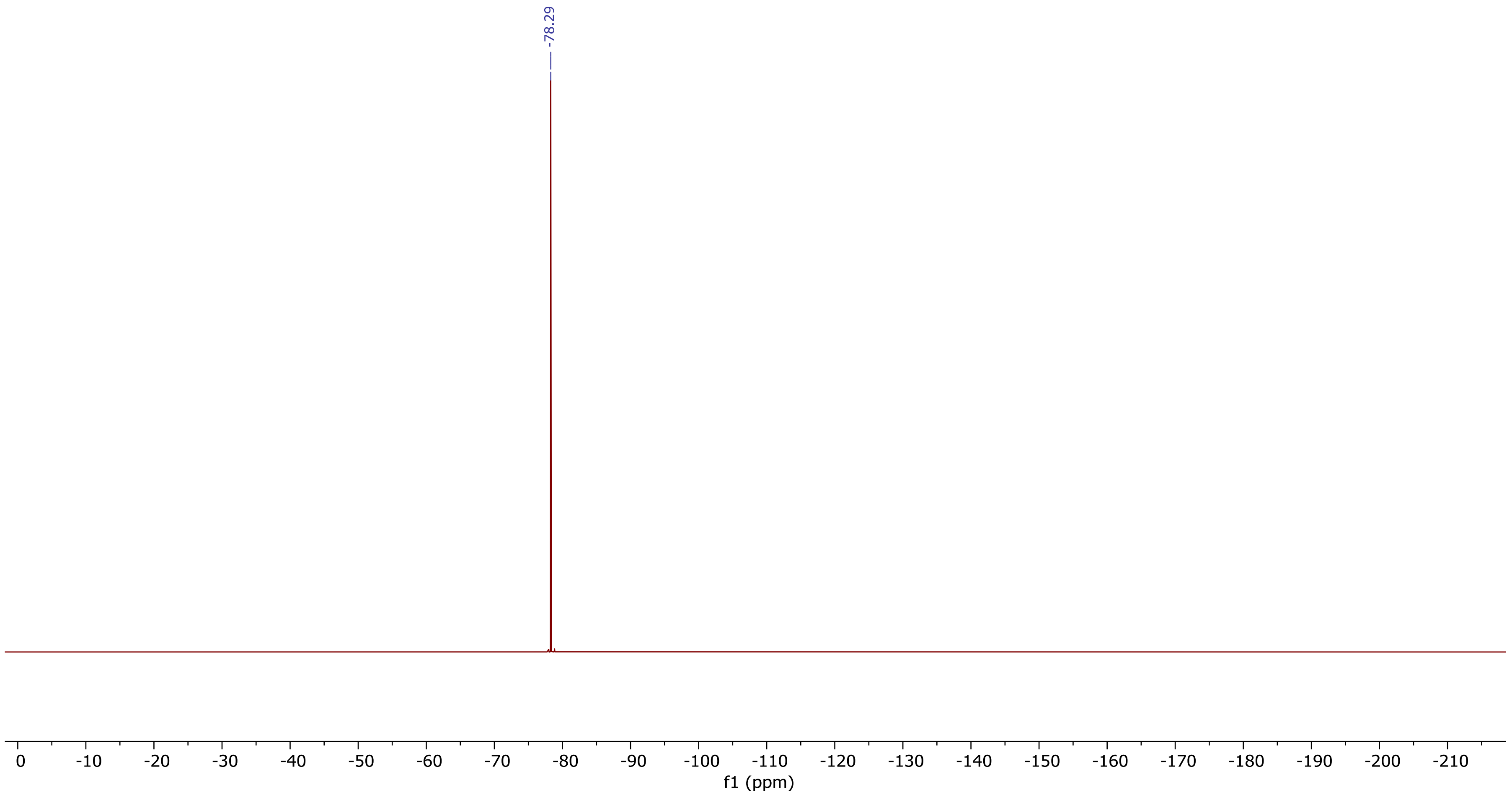

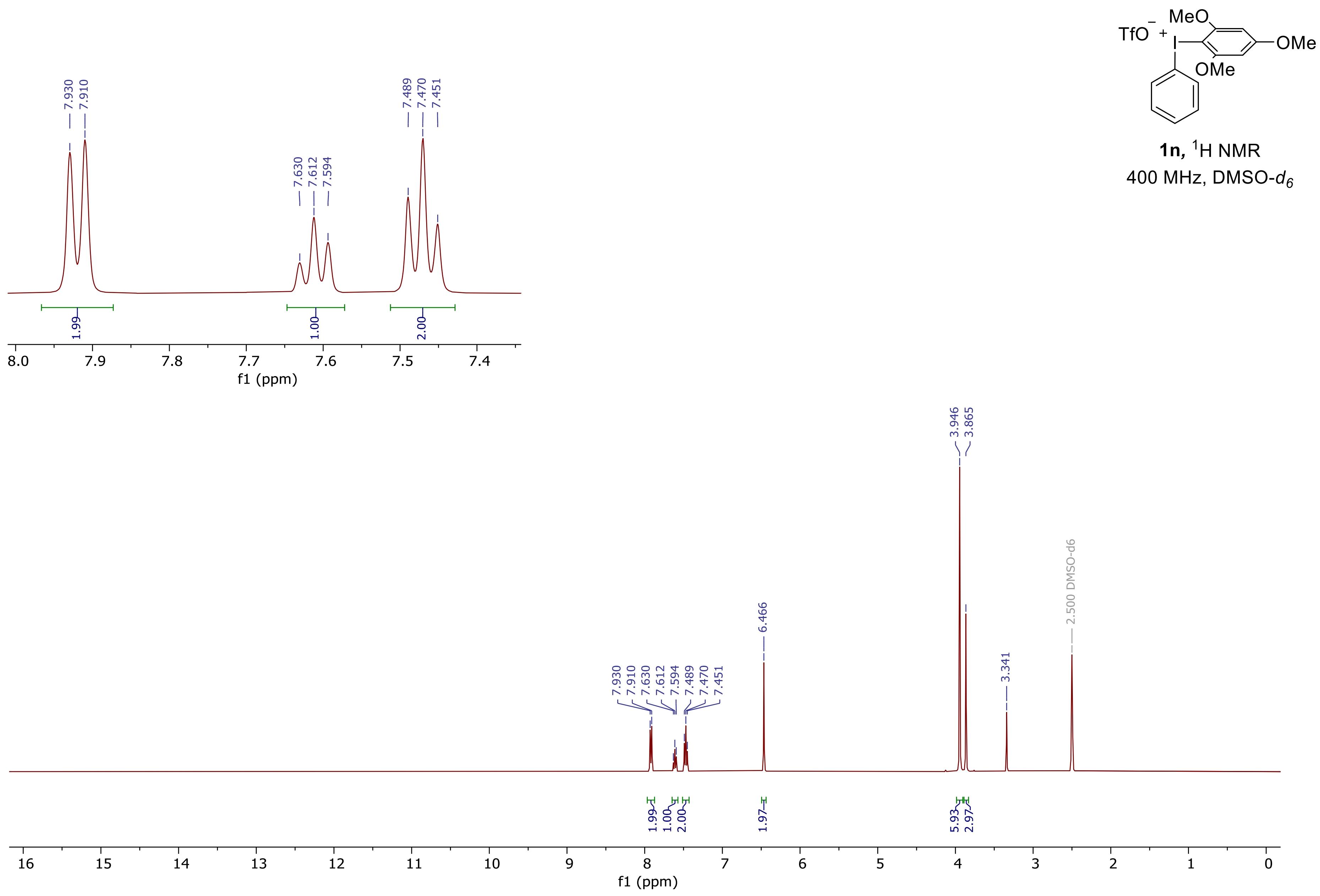
TfO⁻ ⁺I MeO OMe OMe
1n, ¹H NMR
400 MHz, DMSO-d6
7.930
7.910
7.630
7.612
7.594
7.489
7.470
7.451
1.99
1.00
2.00
f1 (ppm)
6.466
3.946
3.865
3.341
2.500 DMSO-d6
1.97
5.93
2.97

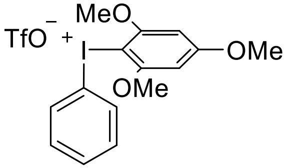

1n, ^{13}C NMR
100 MHz, DMSO-d_6

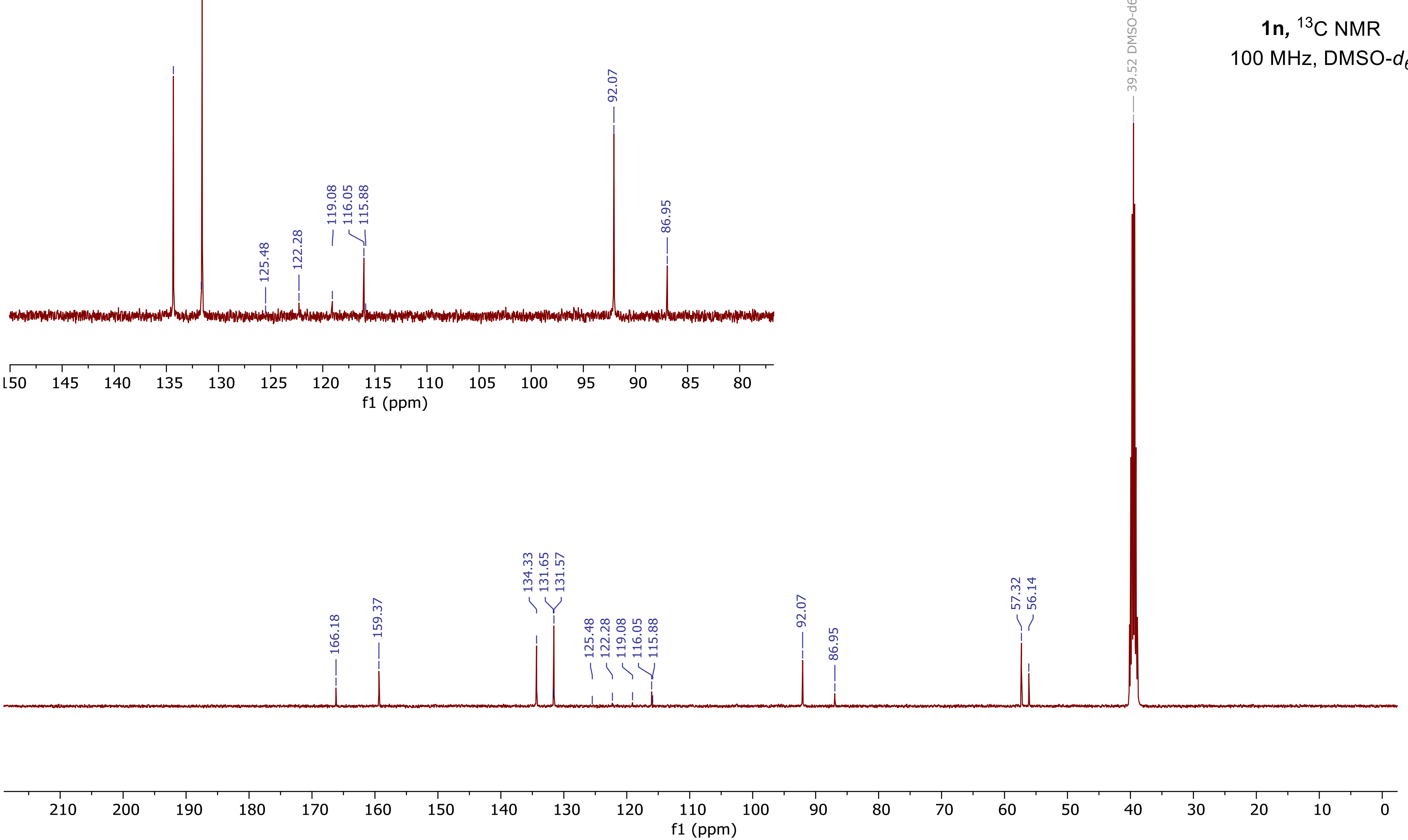

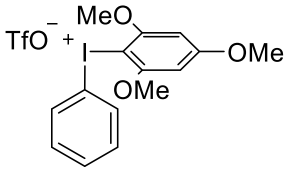

1n, ^{19}F NMR
376 MHz, DMSO-d_6

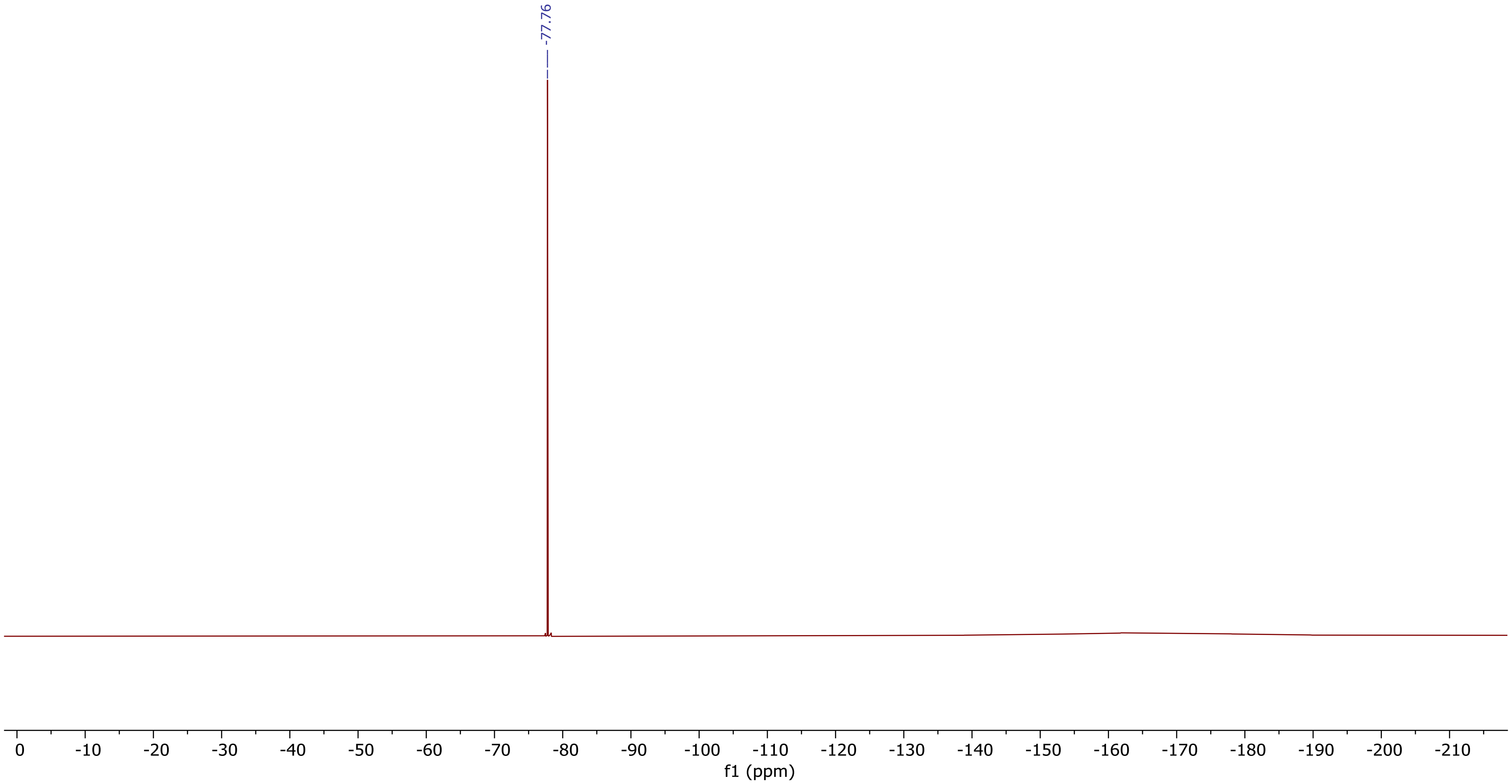

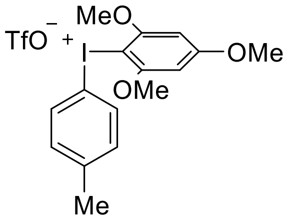

1o, ^{1}H NMR
400 MHz, DMSO-d_6

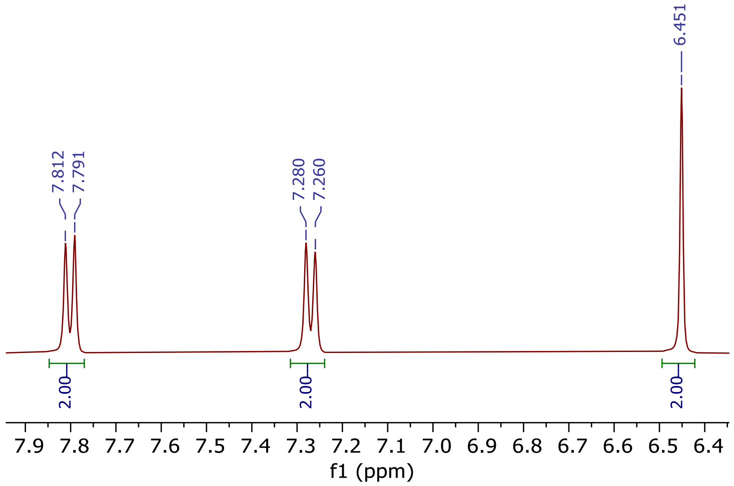

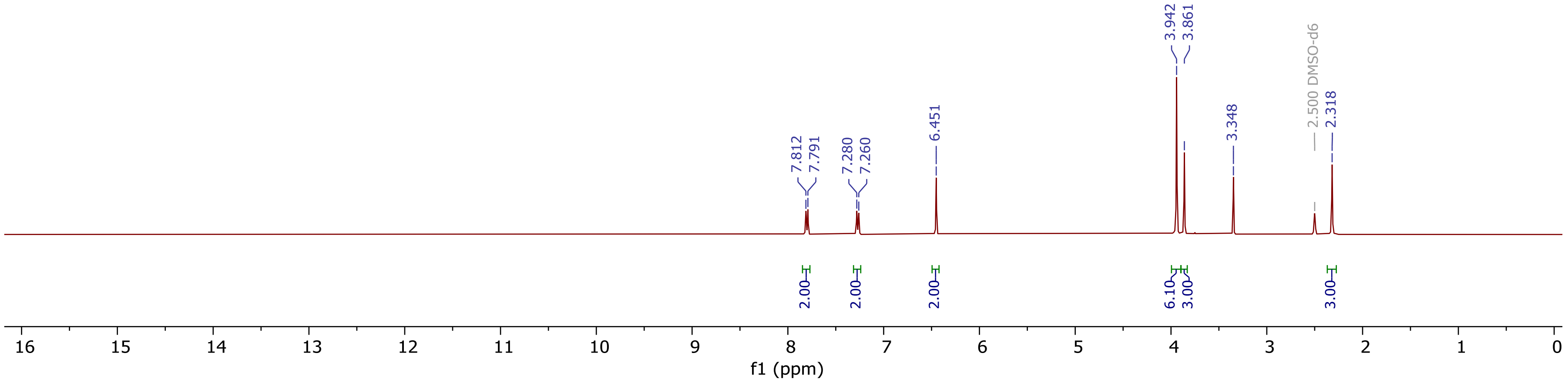

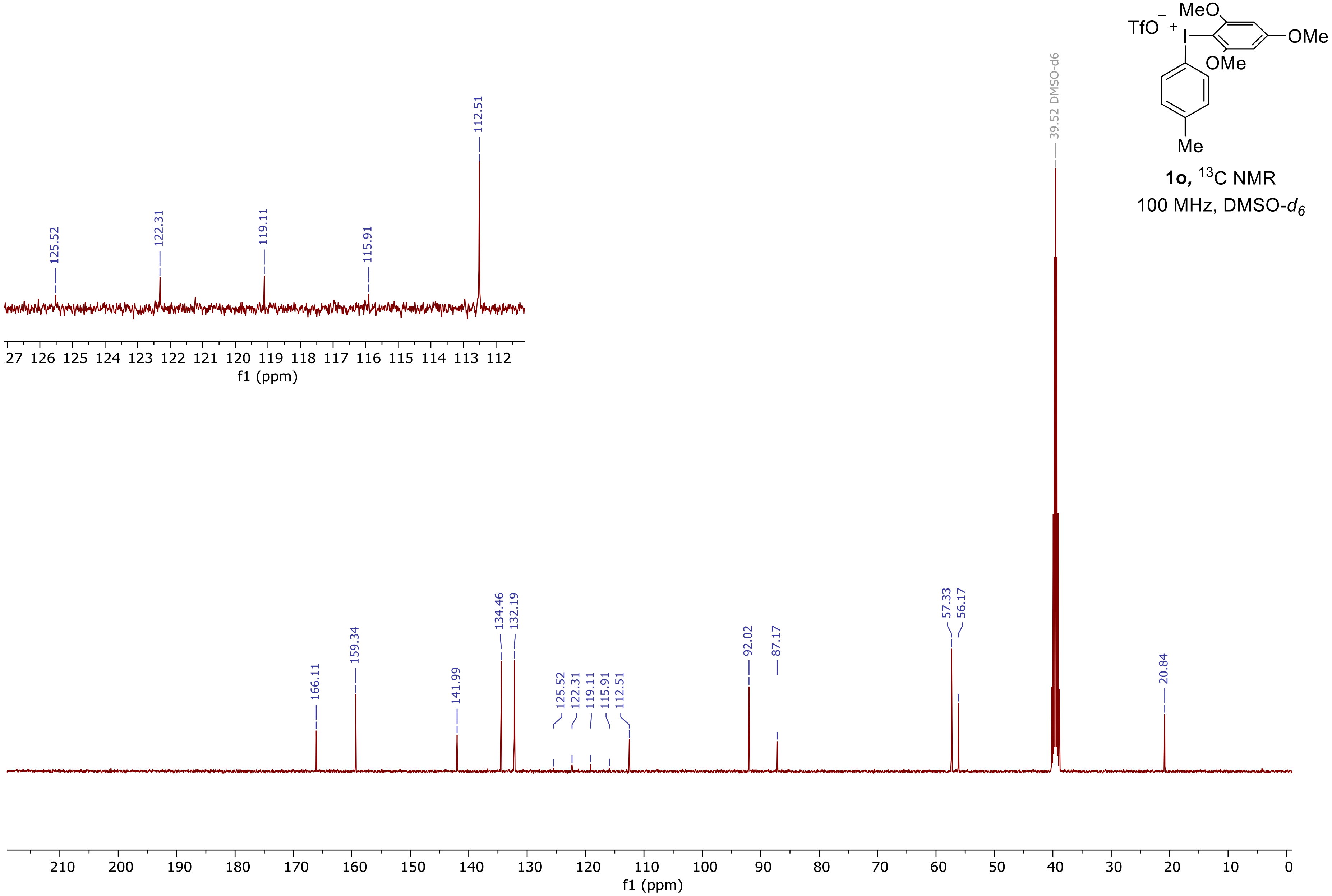

TfO⁻ +I MeO OMe OMe Me
1o, 13C NMR
100 MHz, DMSO-d6
39.52 DMSO-d6
166.11
159.34
141.99
134.46
132.19
125.52
122.31
119.11
115.91
112.51
92.02
87.17
57.33
56.17
20.84
f1 (ppm)

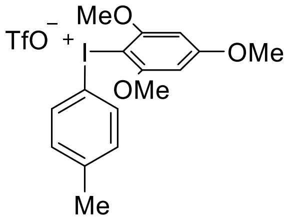

1o, ^{19}F NMR
376 MHz, DMSO-d_6

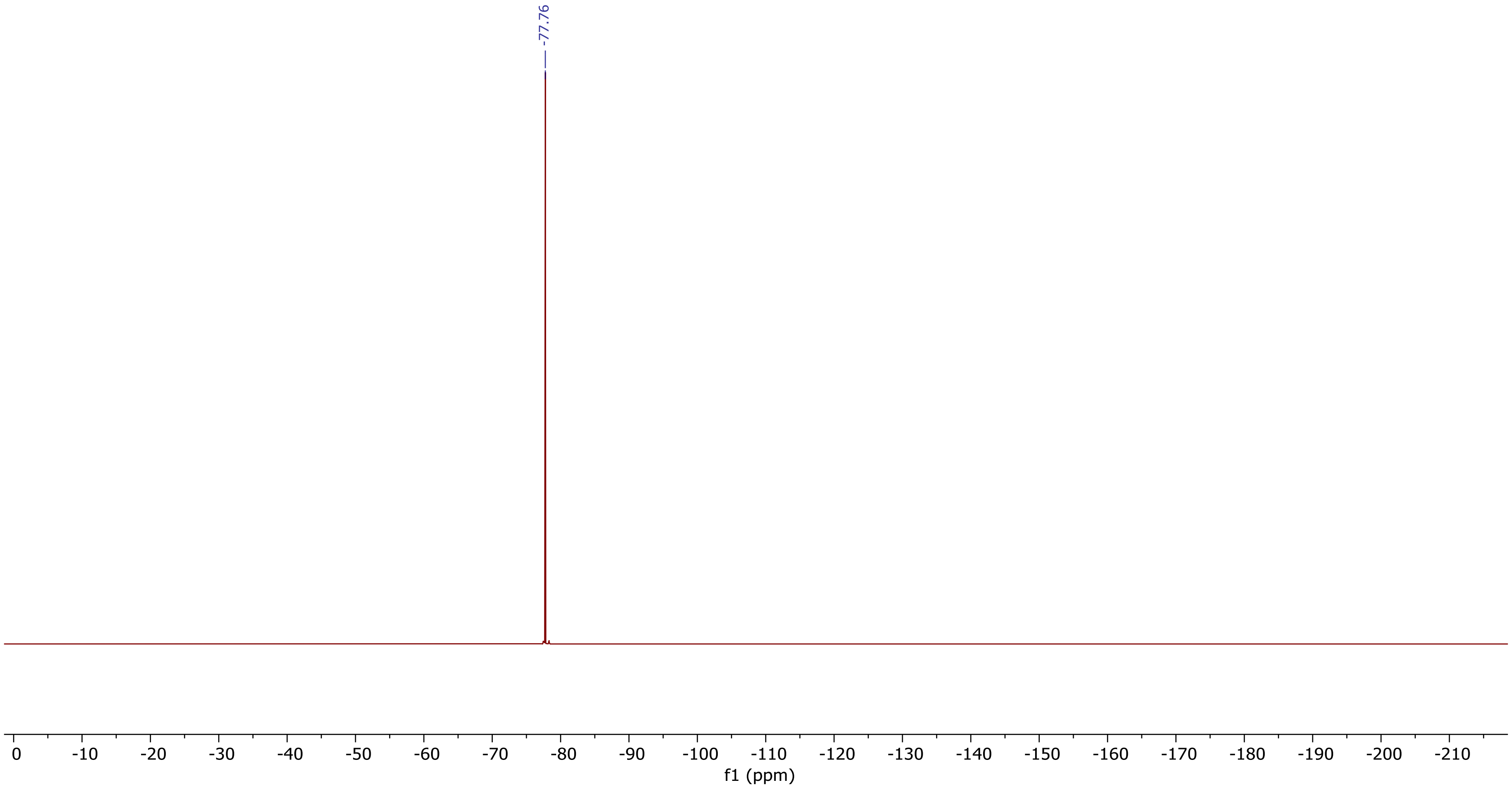

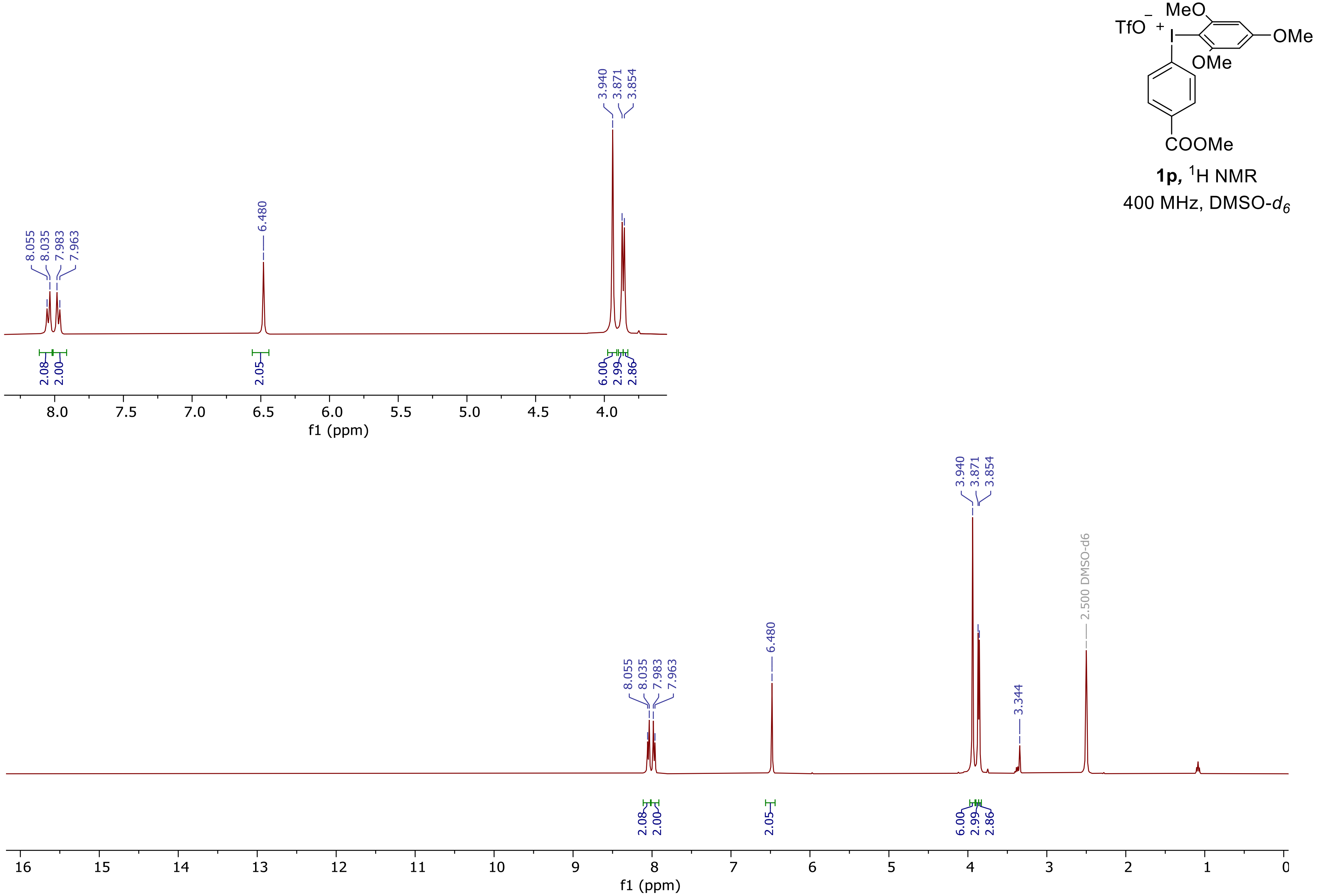

1p, ^{1}H NMR
400 MHz, DMSO-d_6

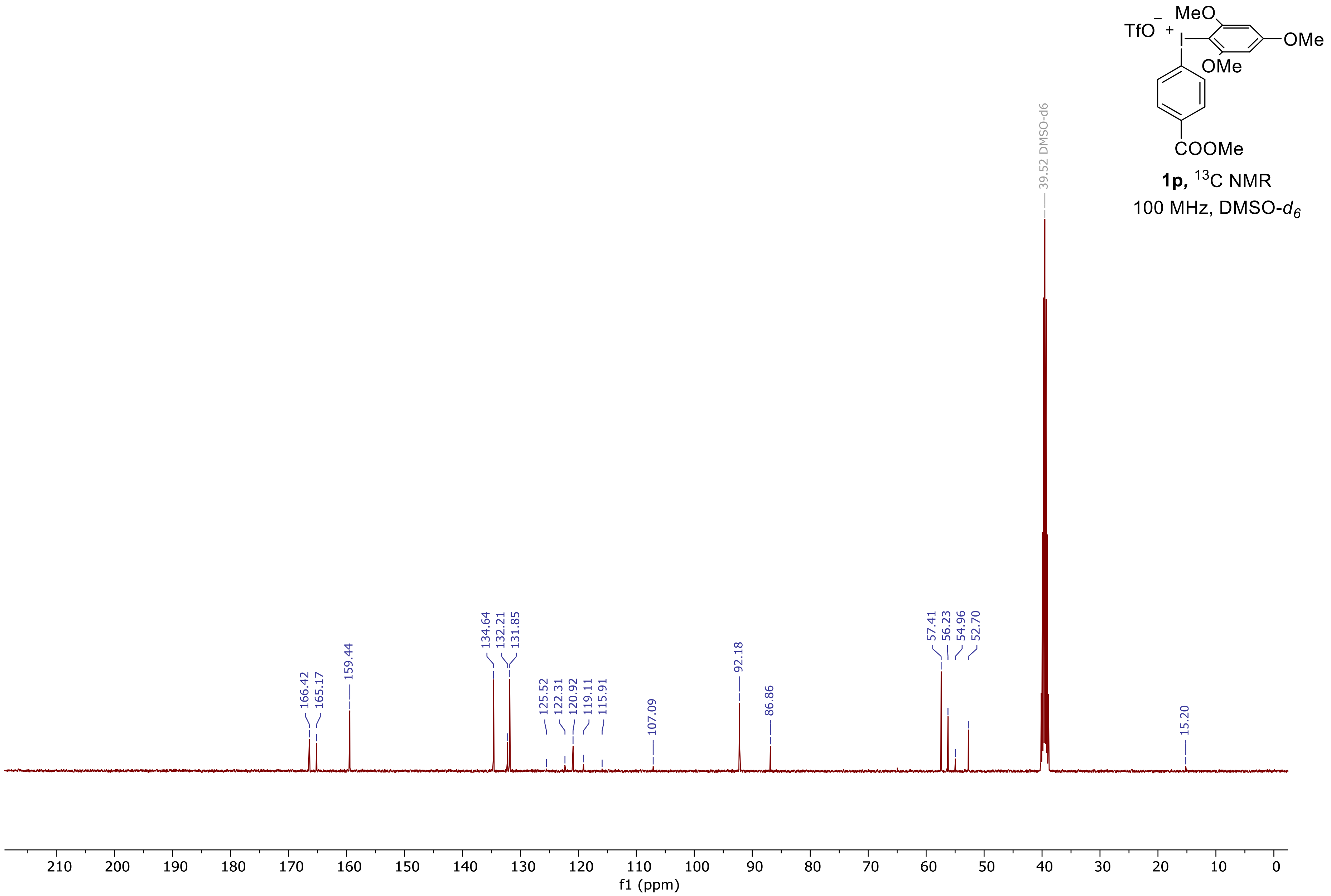
TfO⁻ ⁺I
MeO
OMe
OMe
COOMe
1p, ¹³C NMR
100 MHz, DMSO-d6
166.42
165.17
159.44
134.64
132.21
131.85
125.52
122.31
120.92
119.11
115.91
107.09
92.18
86.86
57.41
56.23
54.96
52.70
39.52 DMSO-d6
15.20
210
200
190
180
170
160
150
140
130
120
110
100
90
80
70
60
50
40
30
20
10
0
f1 (ppm)

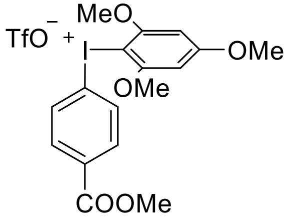

1p, ^{19}F NMR
376 MHz, DMSO-d_6

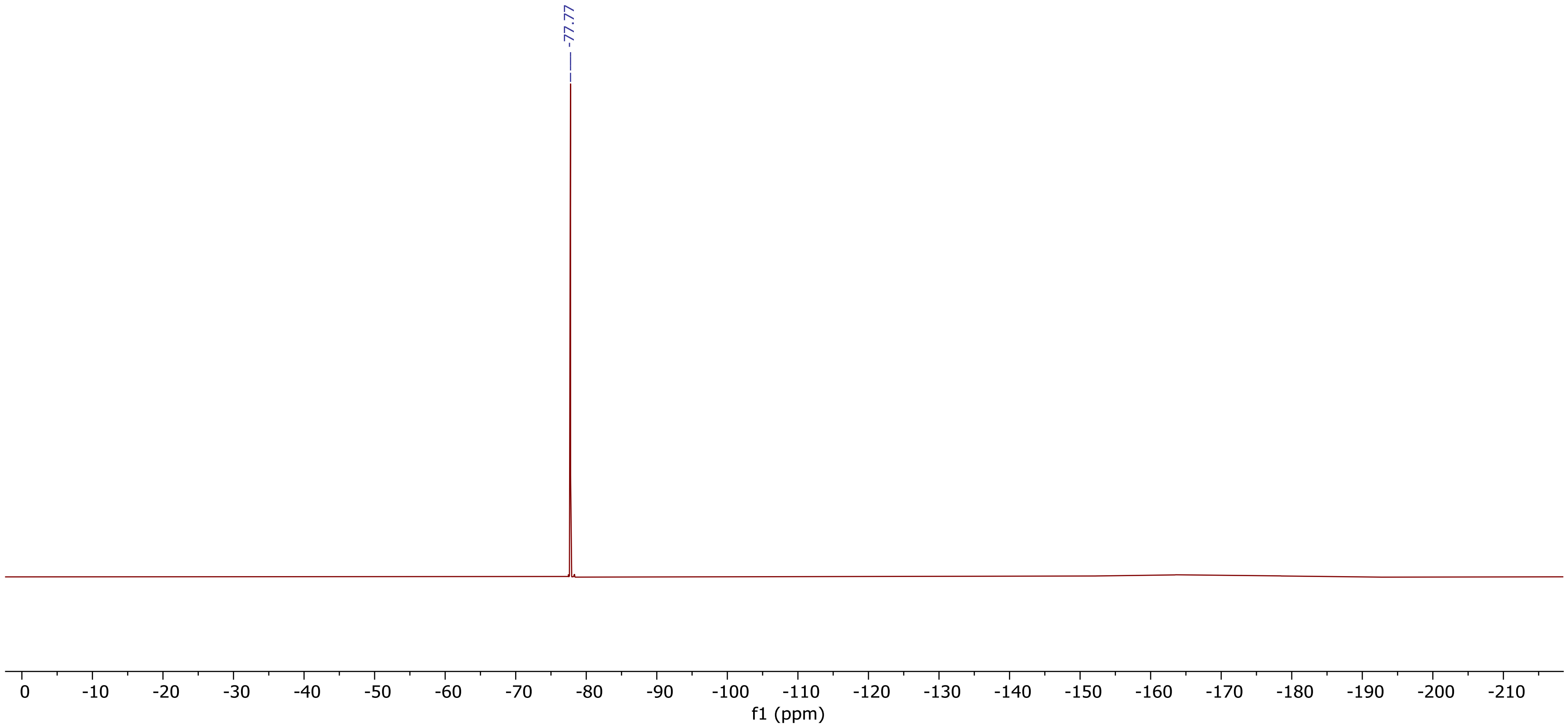

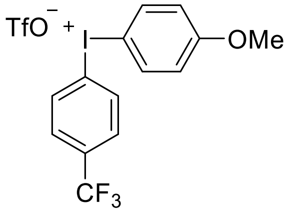

1q, ^{1}H NMR
400 MHz, DMSO-*d*$_6$

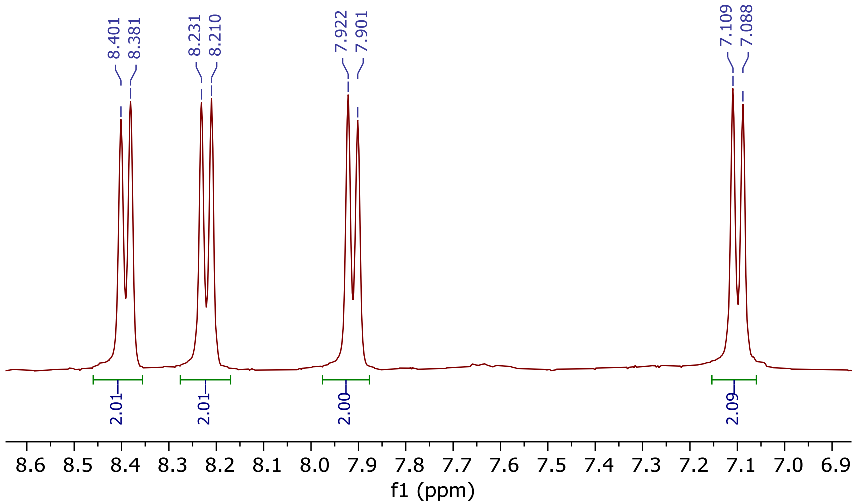

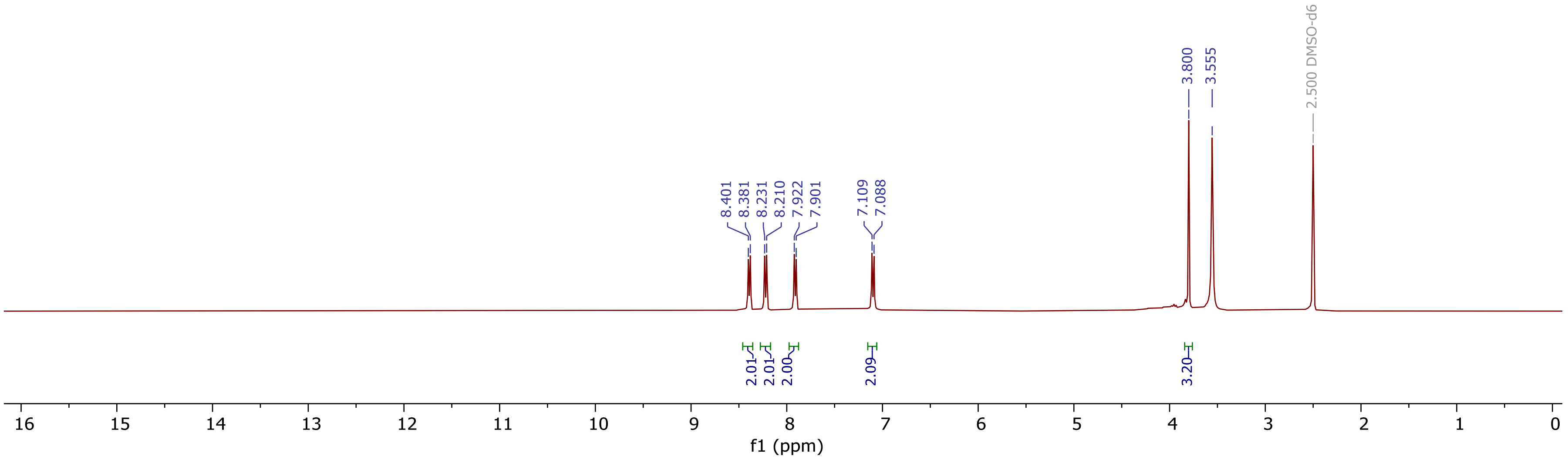

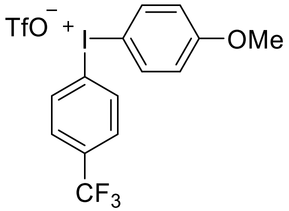

1q, ^{13}C NMR
100 MHz, DMSO-d_6

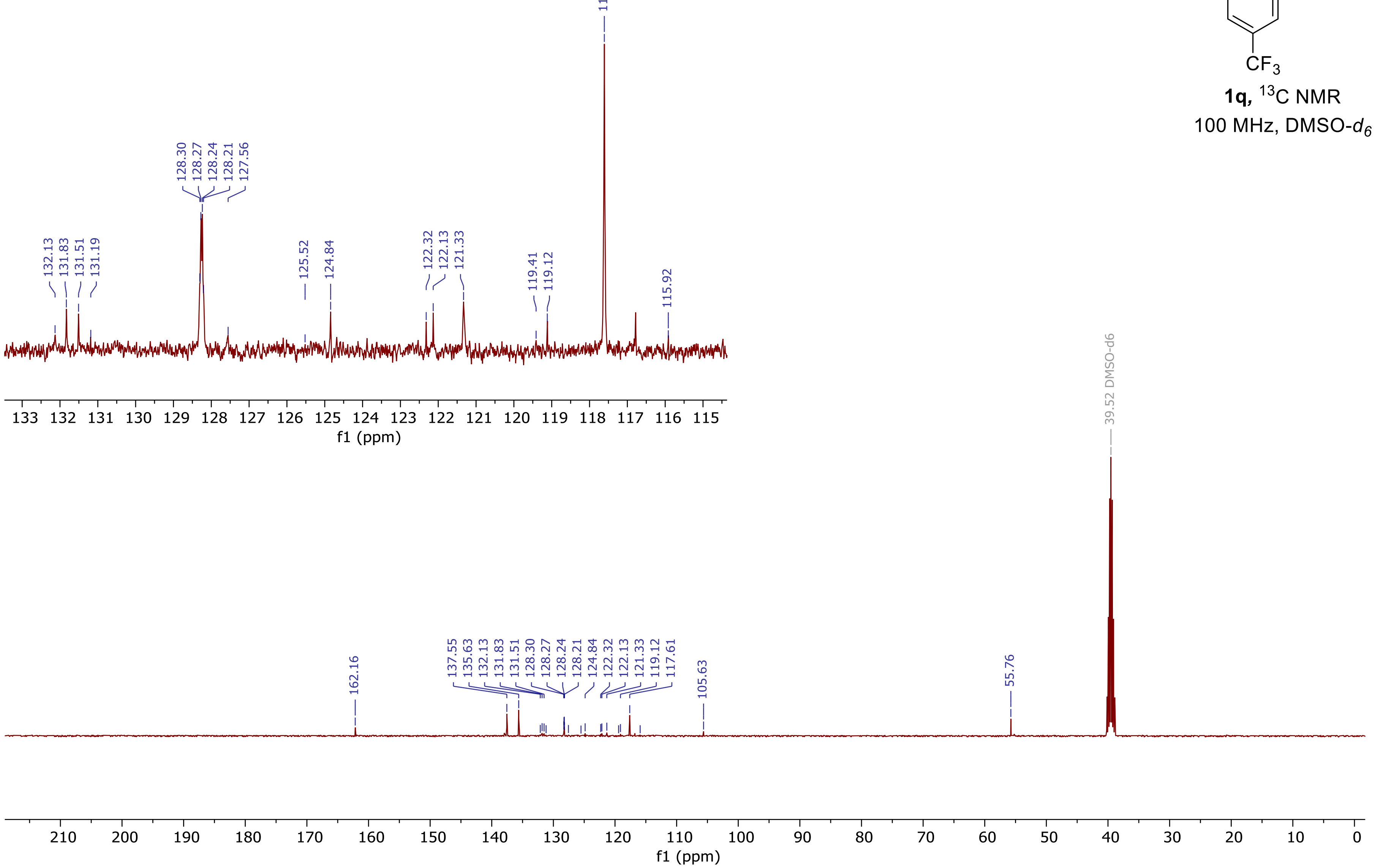

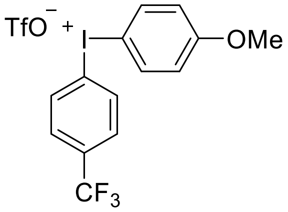

1q, ^{19}F NMR
376 MHz, DMSO-d_6

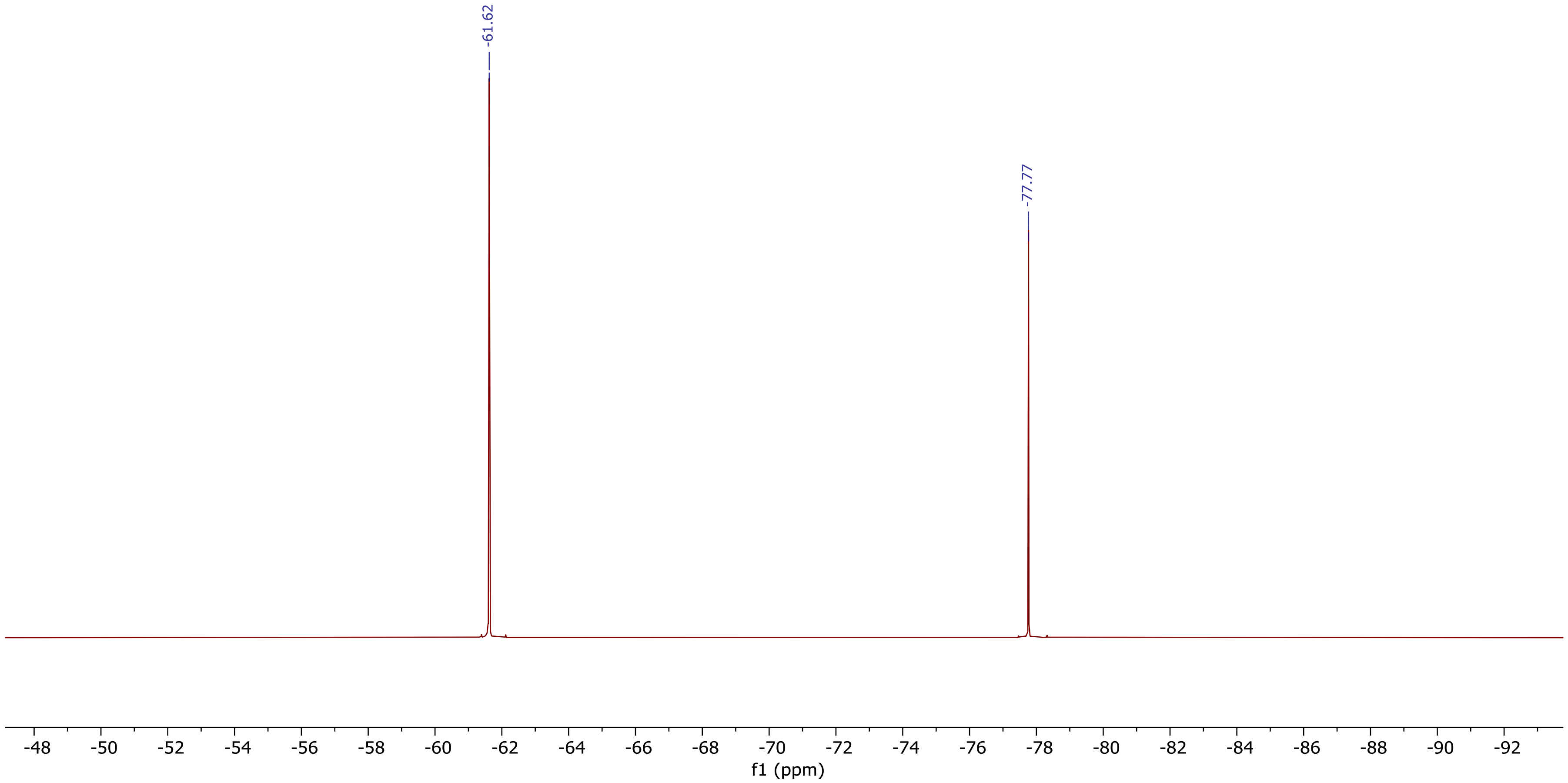

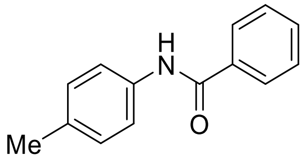

2aa, ^{1}H NMR
400 MHz, $CDCl_3$

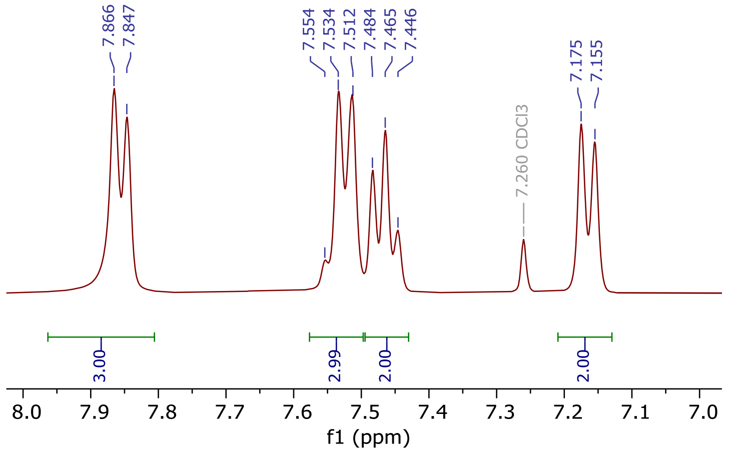

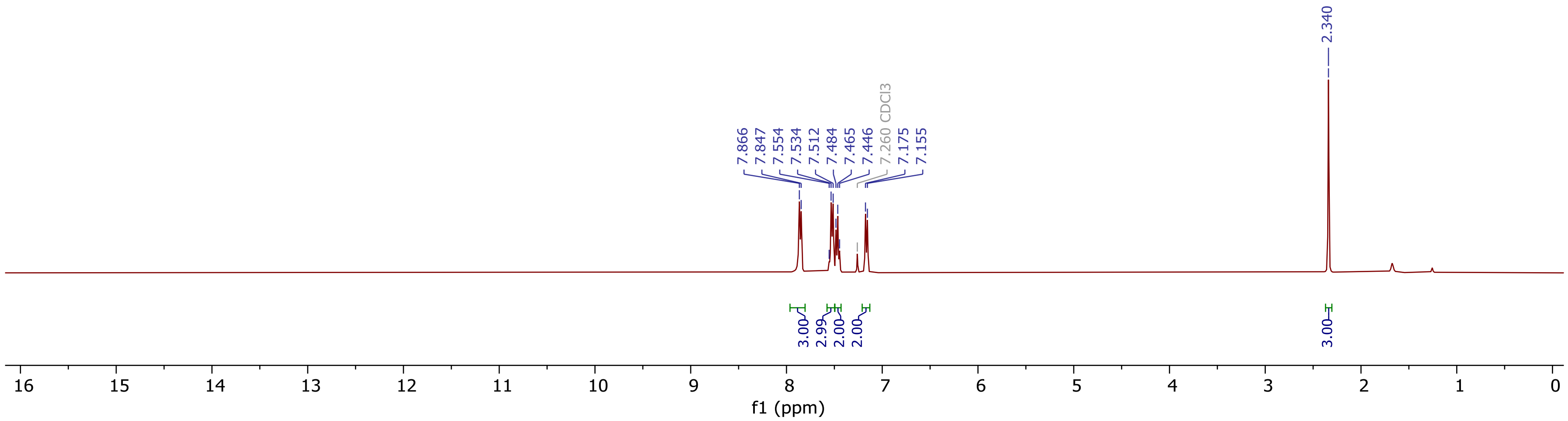

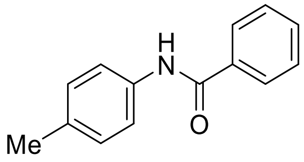

2aa, ^{13}C NMR
100 MHz, $CDCl_3$

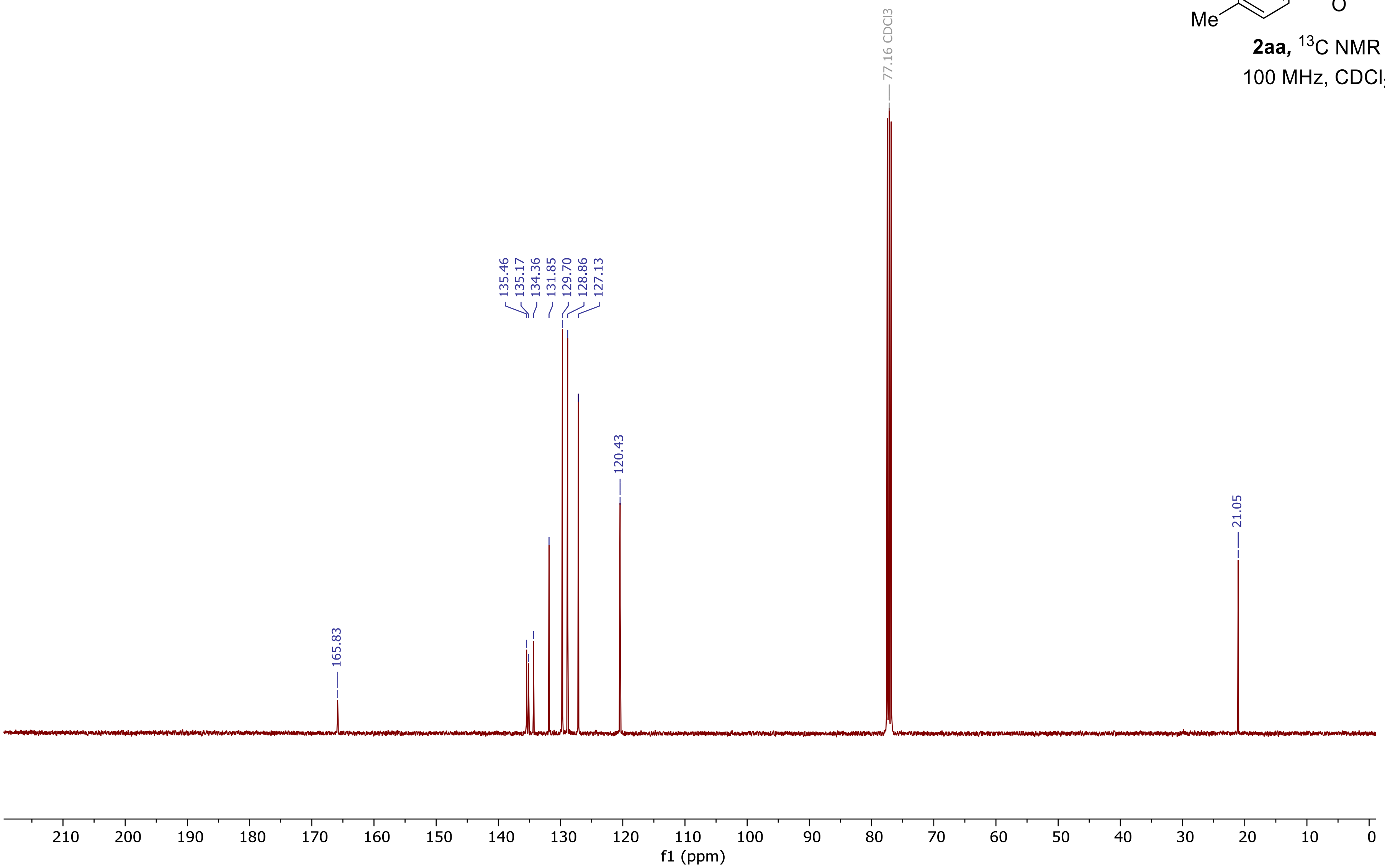

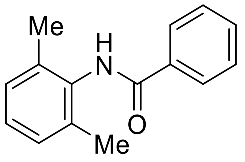

2ba, ^{1}H NMR
400 MHz, $CDCl_3$

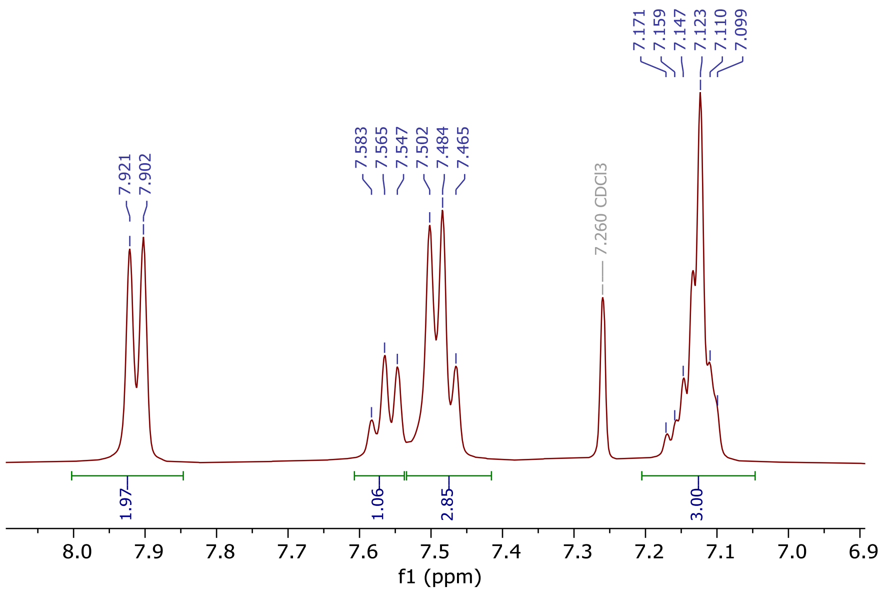

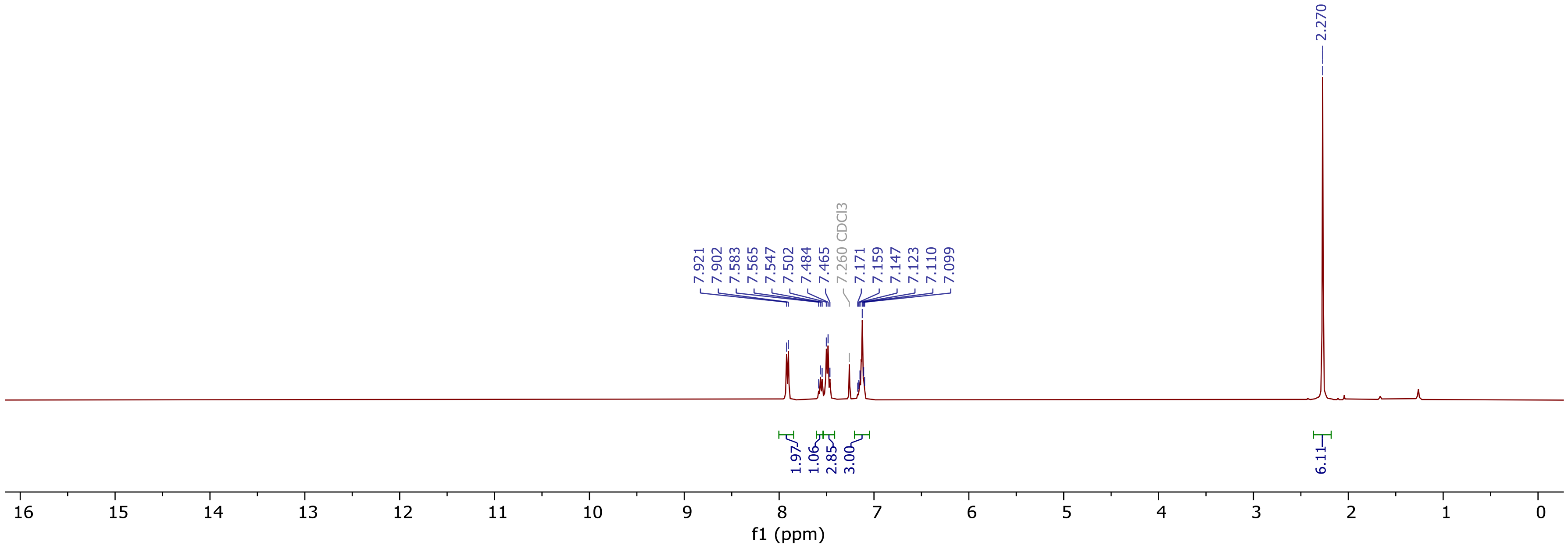

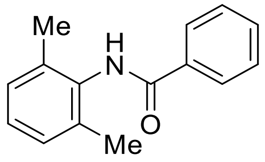

2ba, ^{13}C NMR
100 MHz, $CDCl_3$

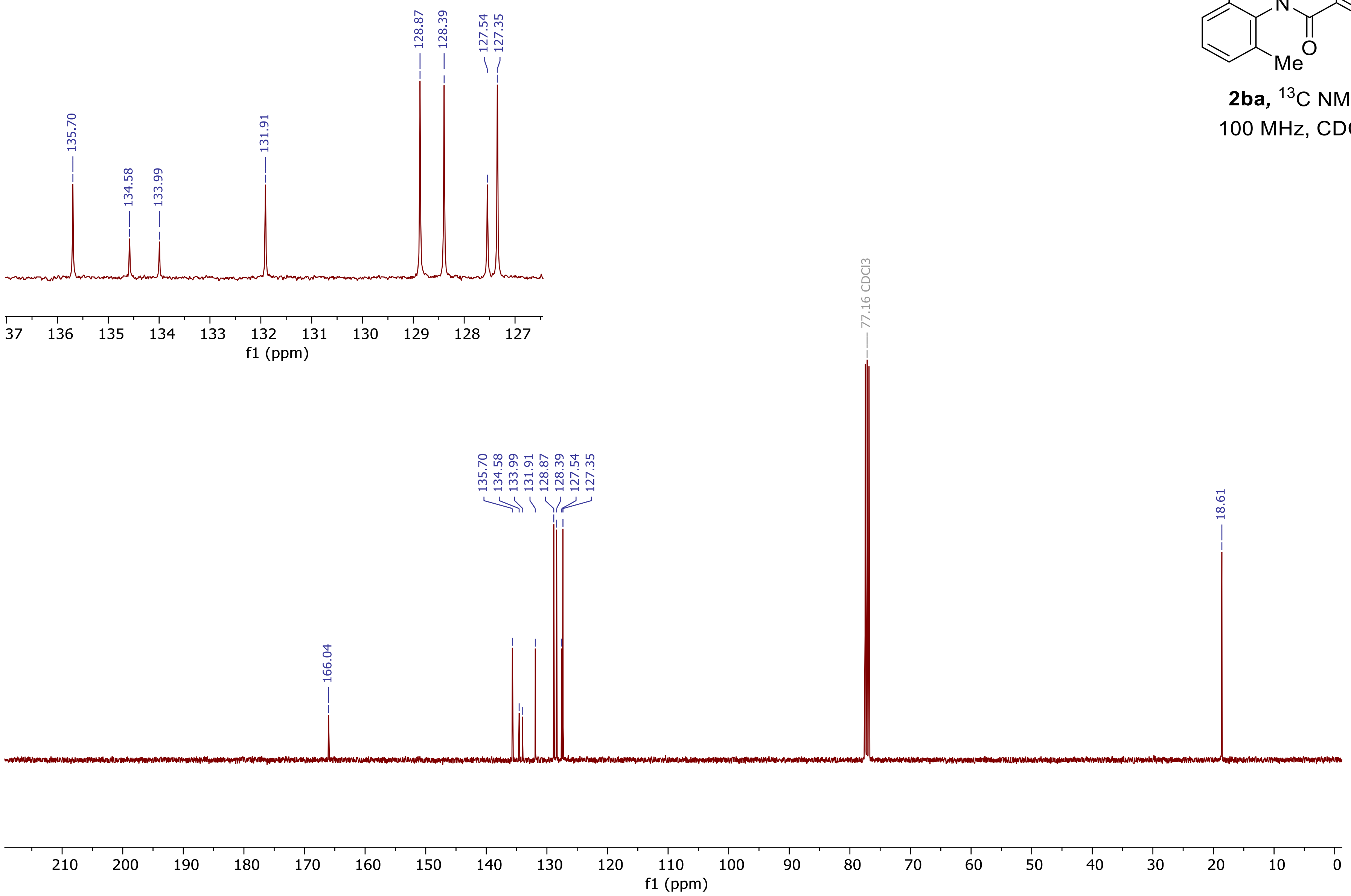

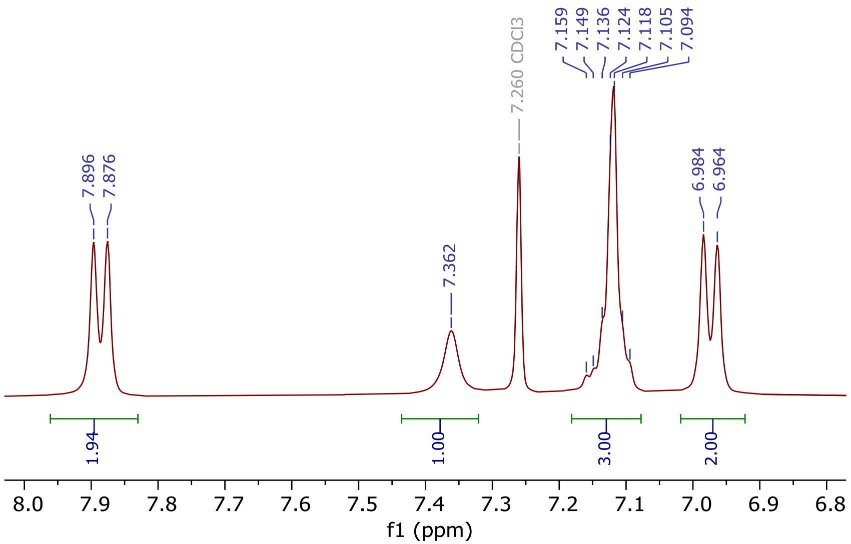

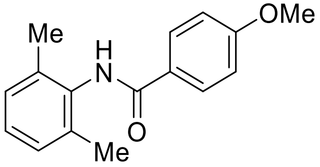

2bb, ^{1}H NMR
400 MHz, $CDCl_3$

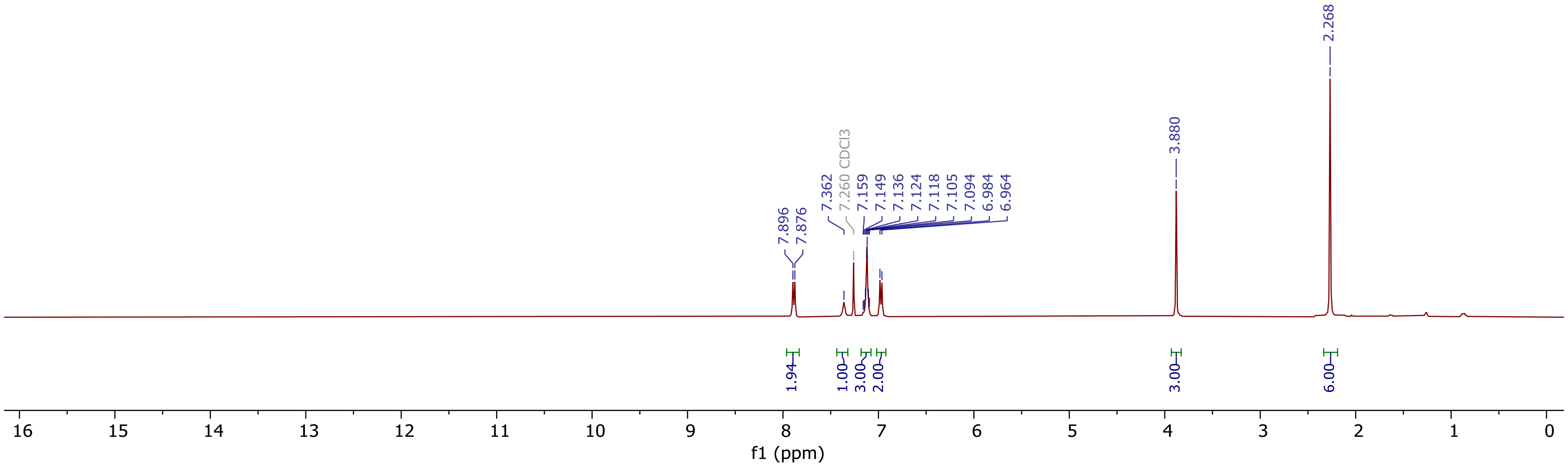

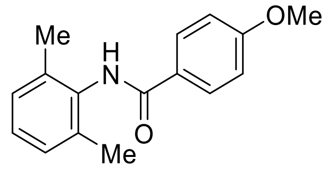

2bb, ^{13}C NMR
100 MHz, $CDCl_3$

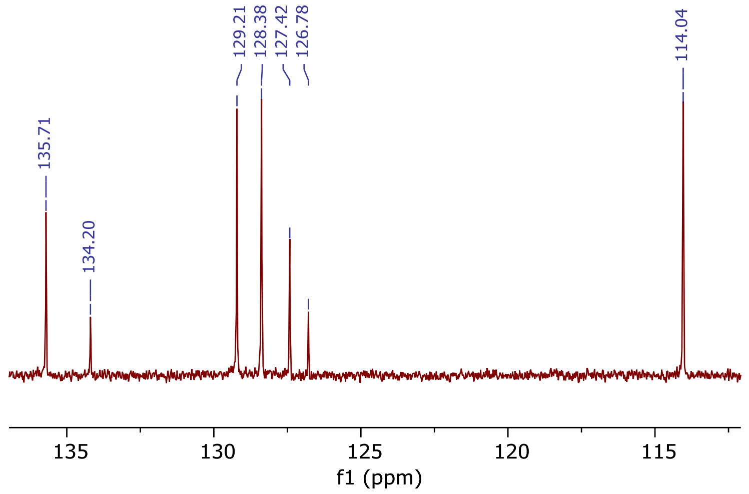

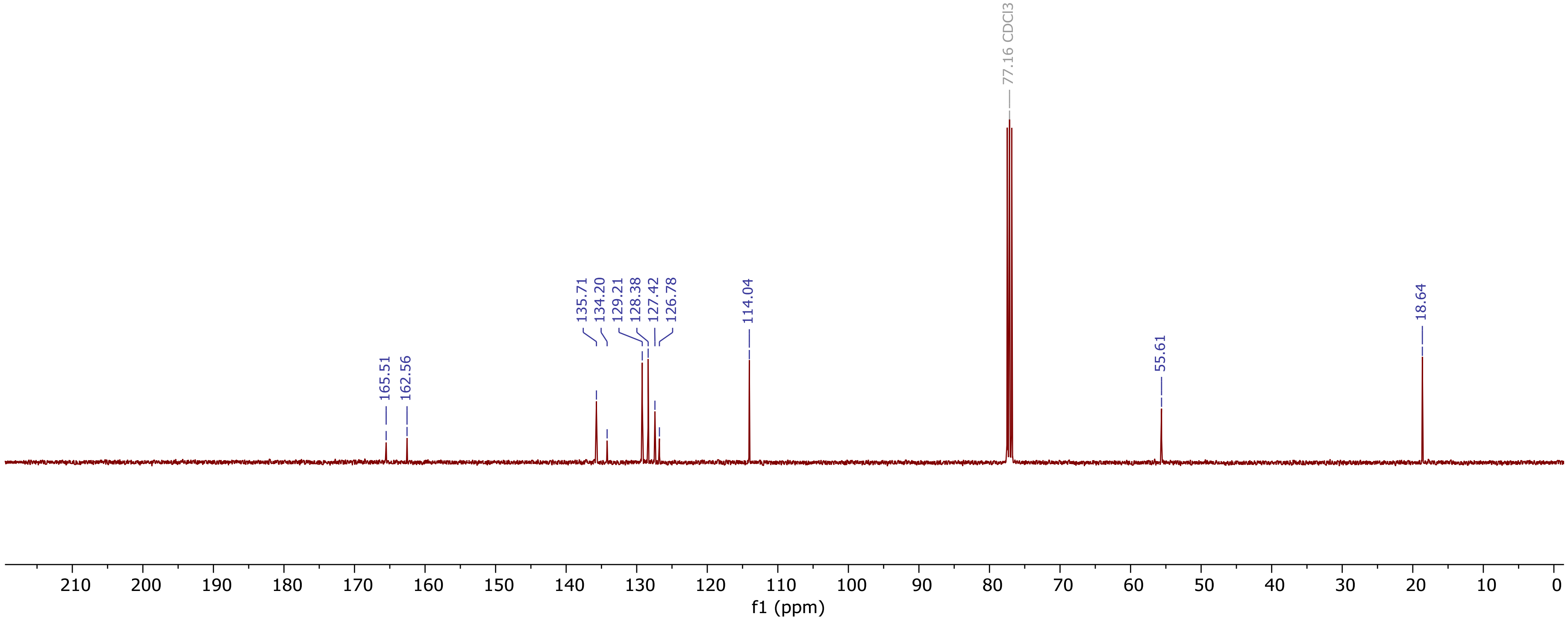

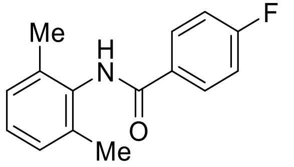

2bc, ^{1}H NMR
400 MHz, $CDCl_3$

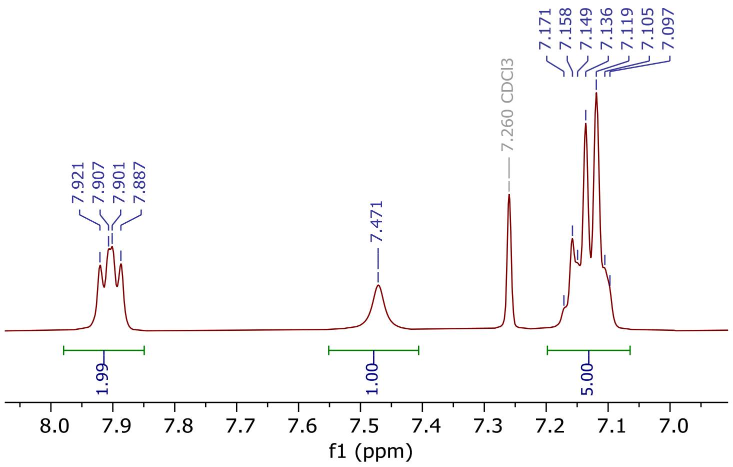

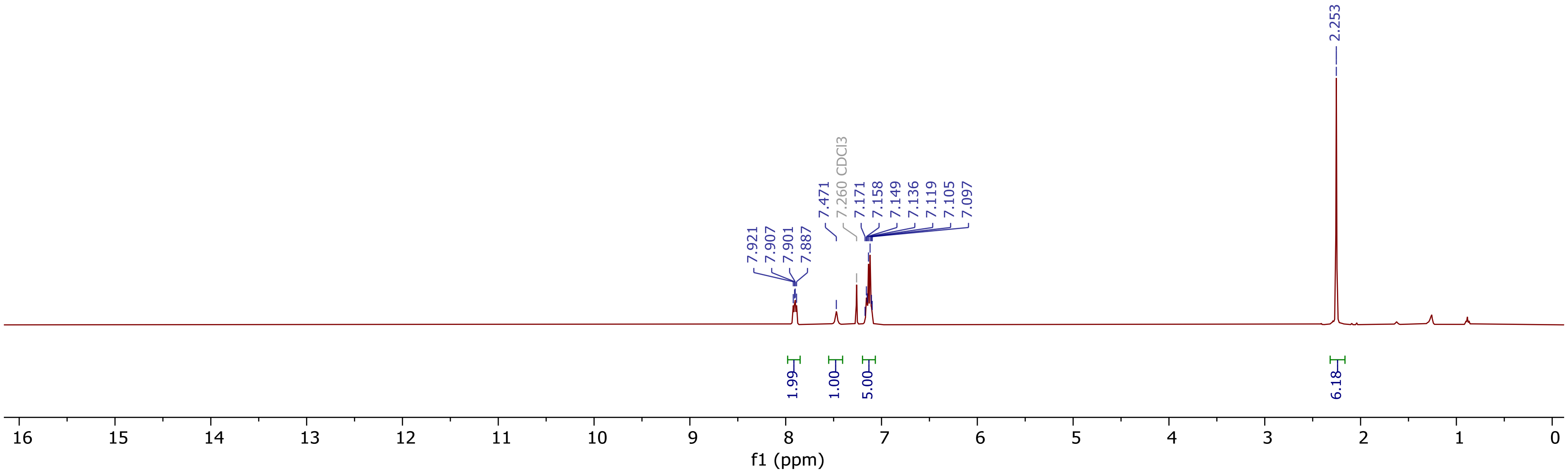

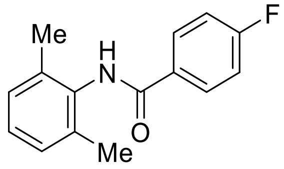

2bc, ^{13}C NMR
100 MHz, $CDCl_3$

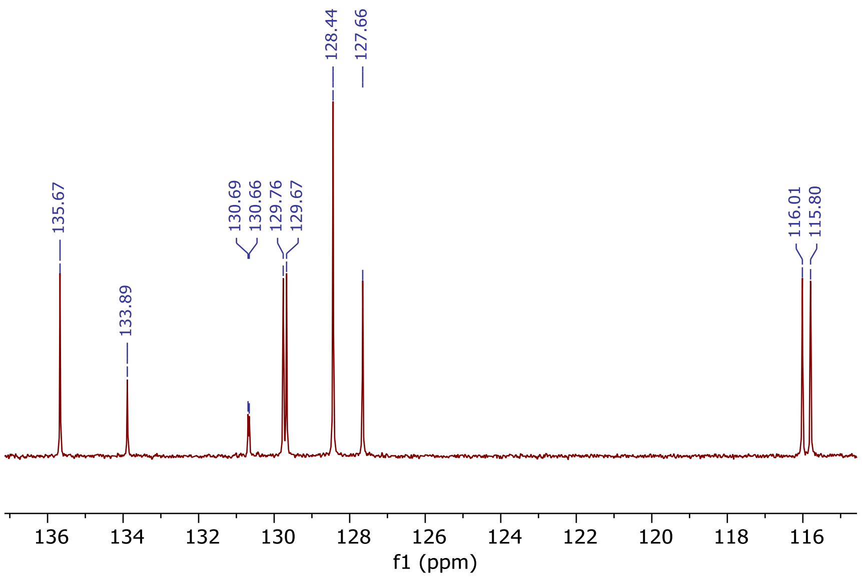

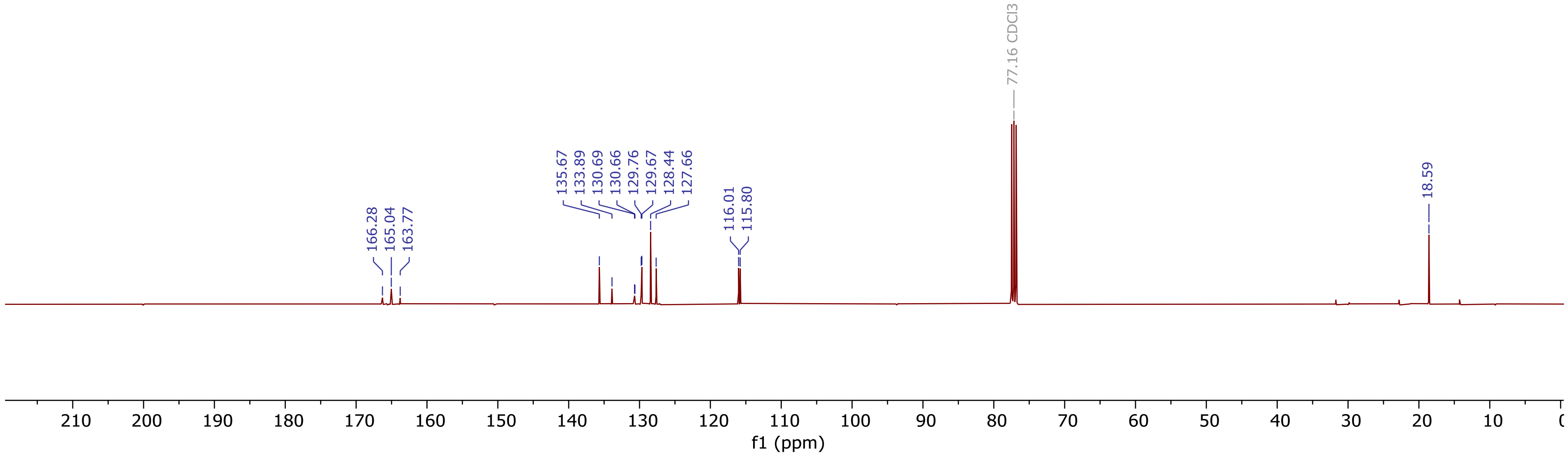

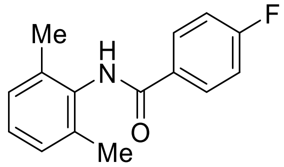

2bc, ^{19}F NMR
376 MHz, $CDCl_3$

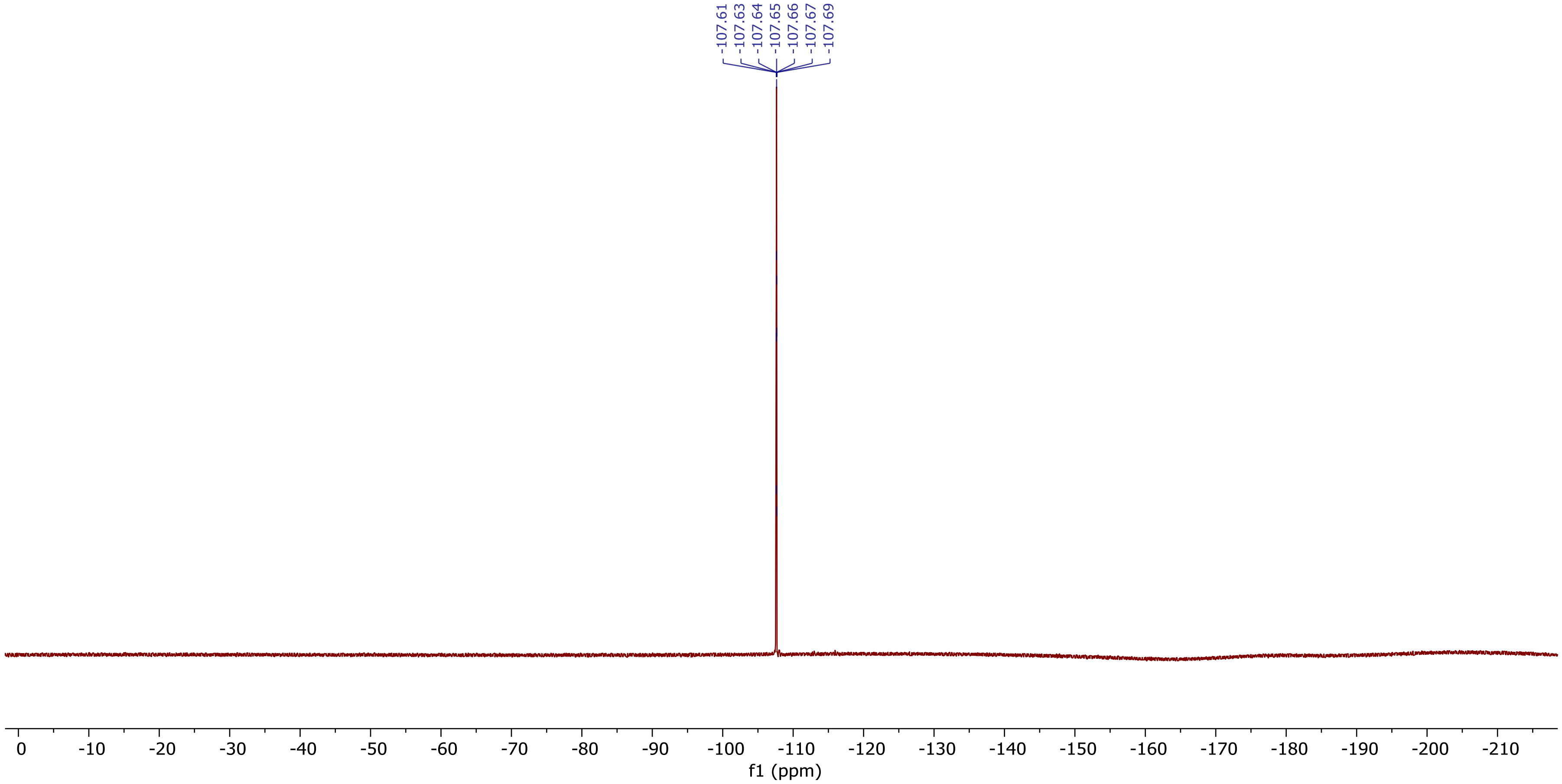

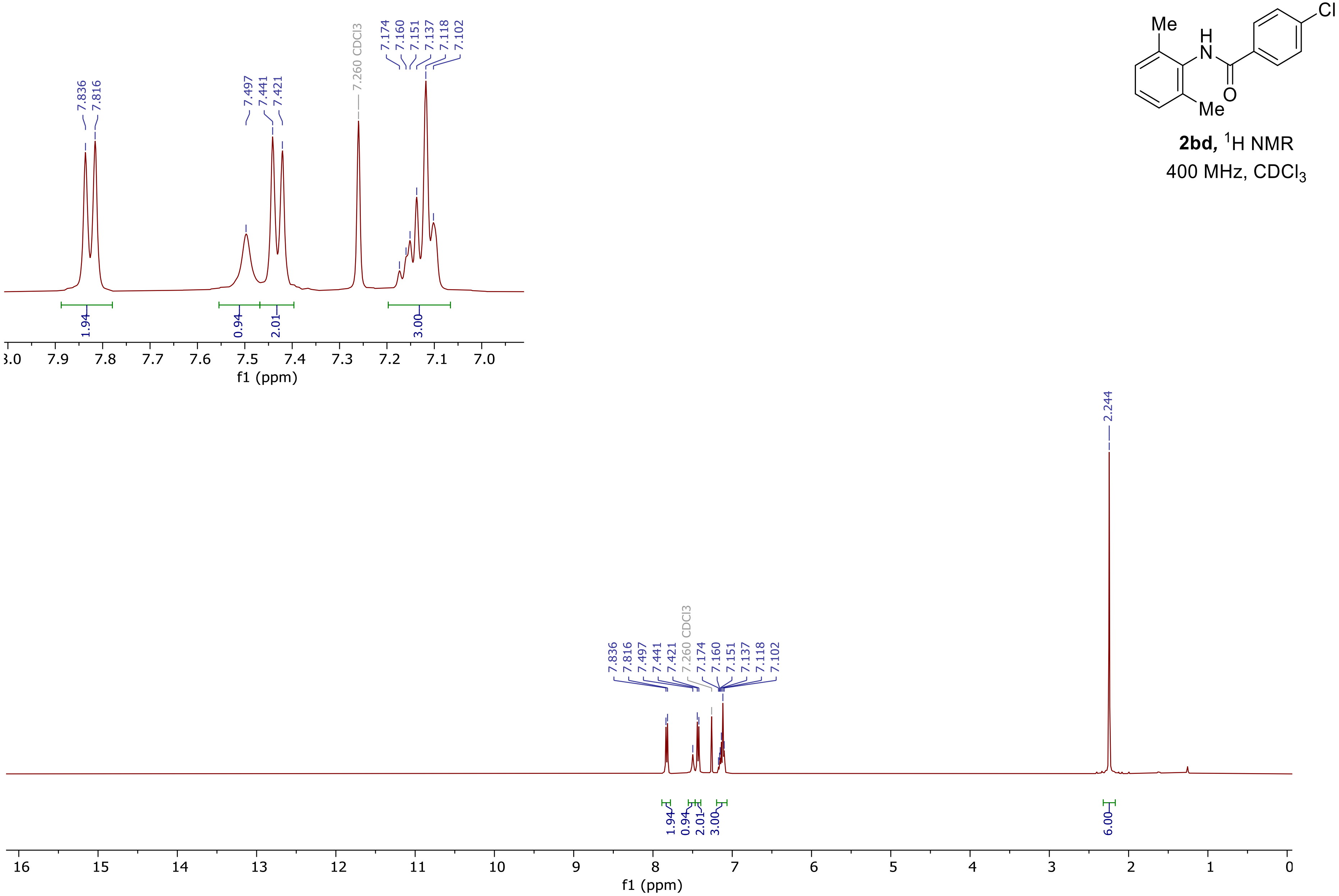

2bd, ^{1}H NMR
400 MHz, $CDCl_3$

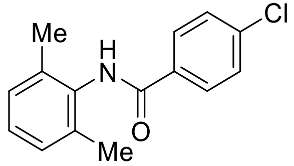

2bd, ^{13}C NMR
100 MHz, $CDCl_3$

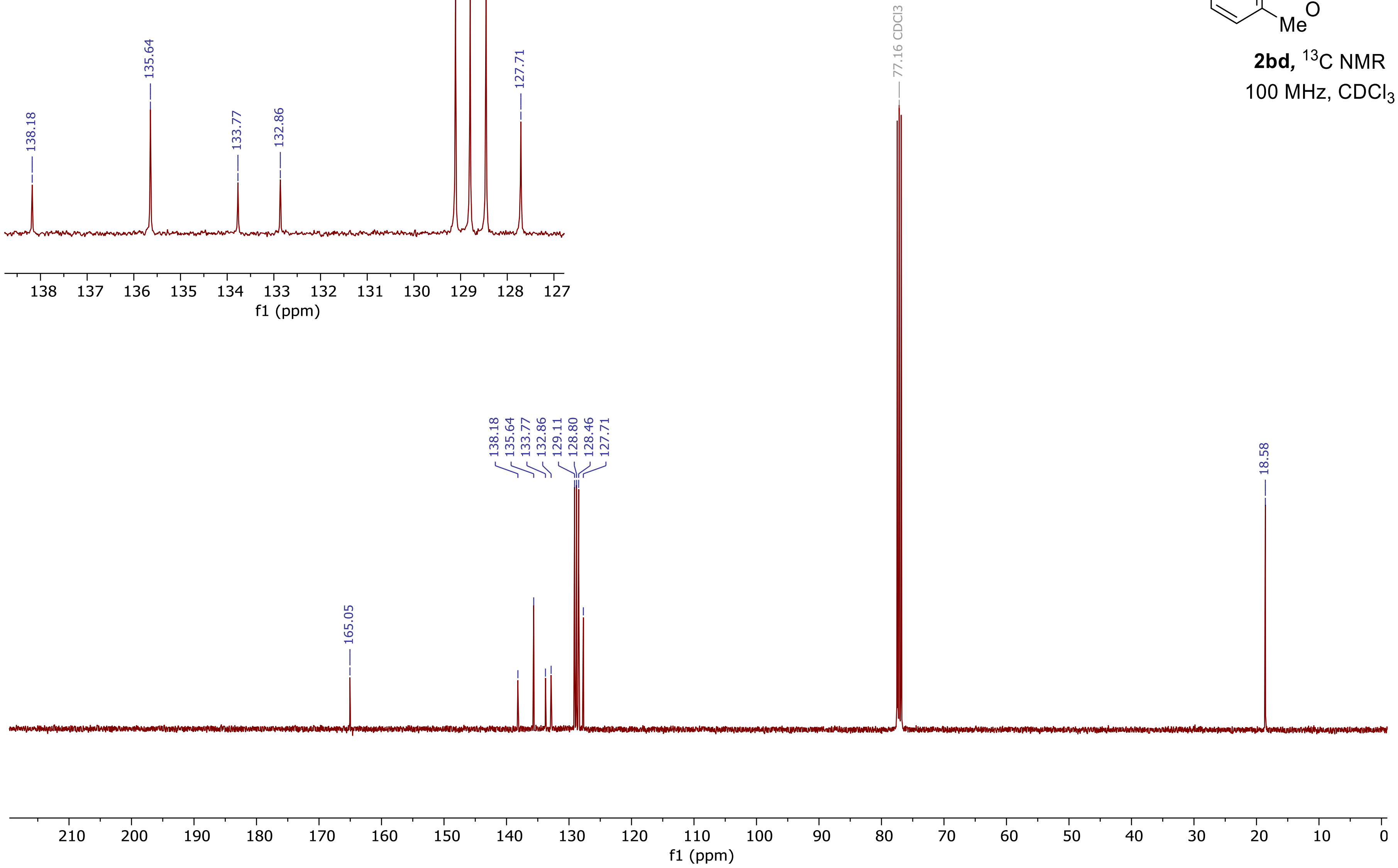

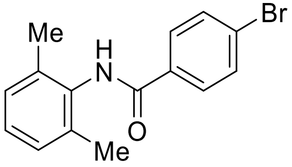

2be, ^{1}H NMR
400 MHz, $CDCl_3$

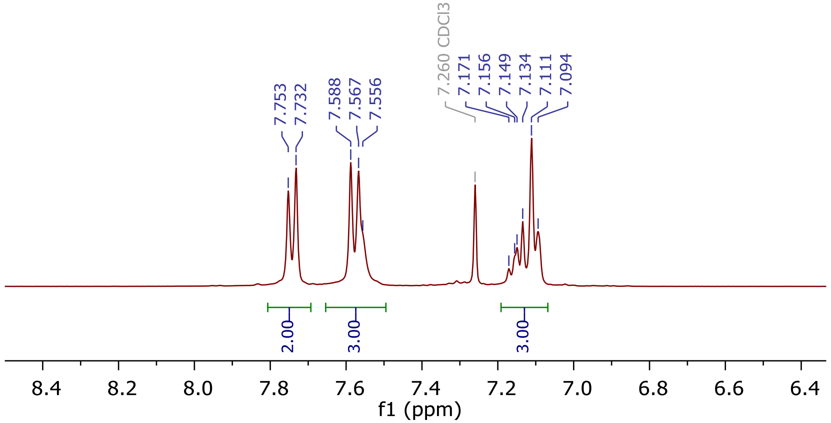

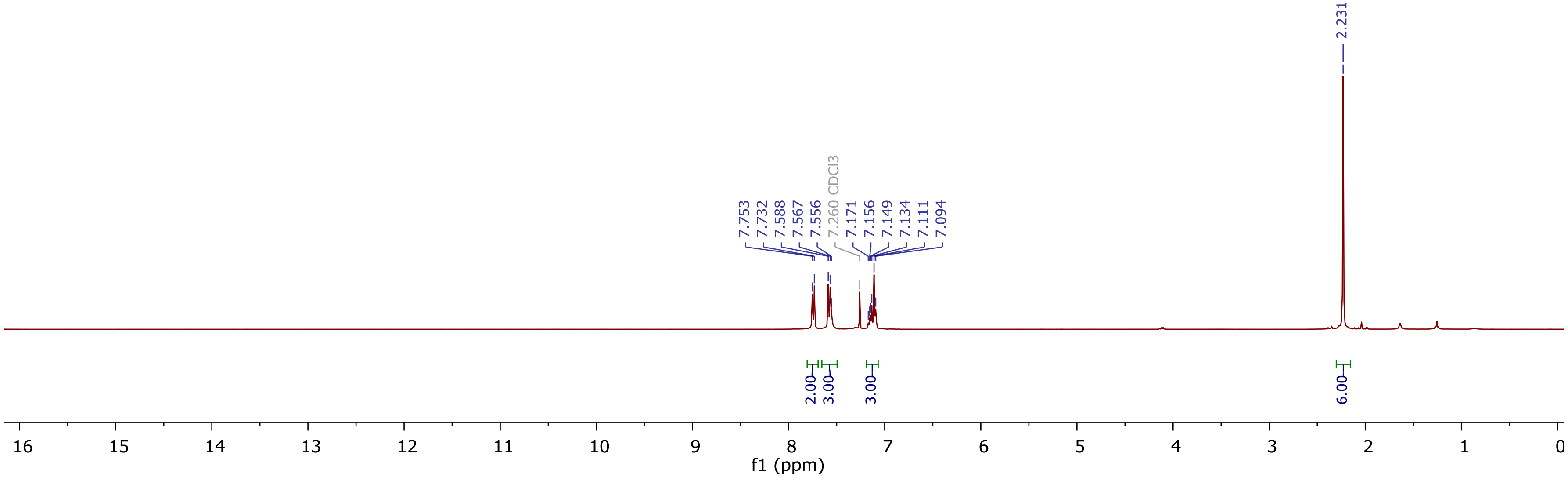

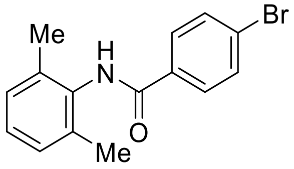

2be, ^{13}C NMR
100 MHz, $CDCl_3$

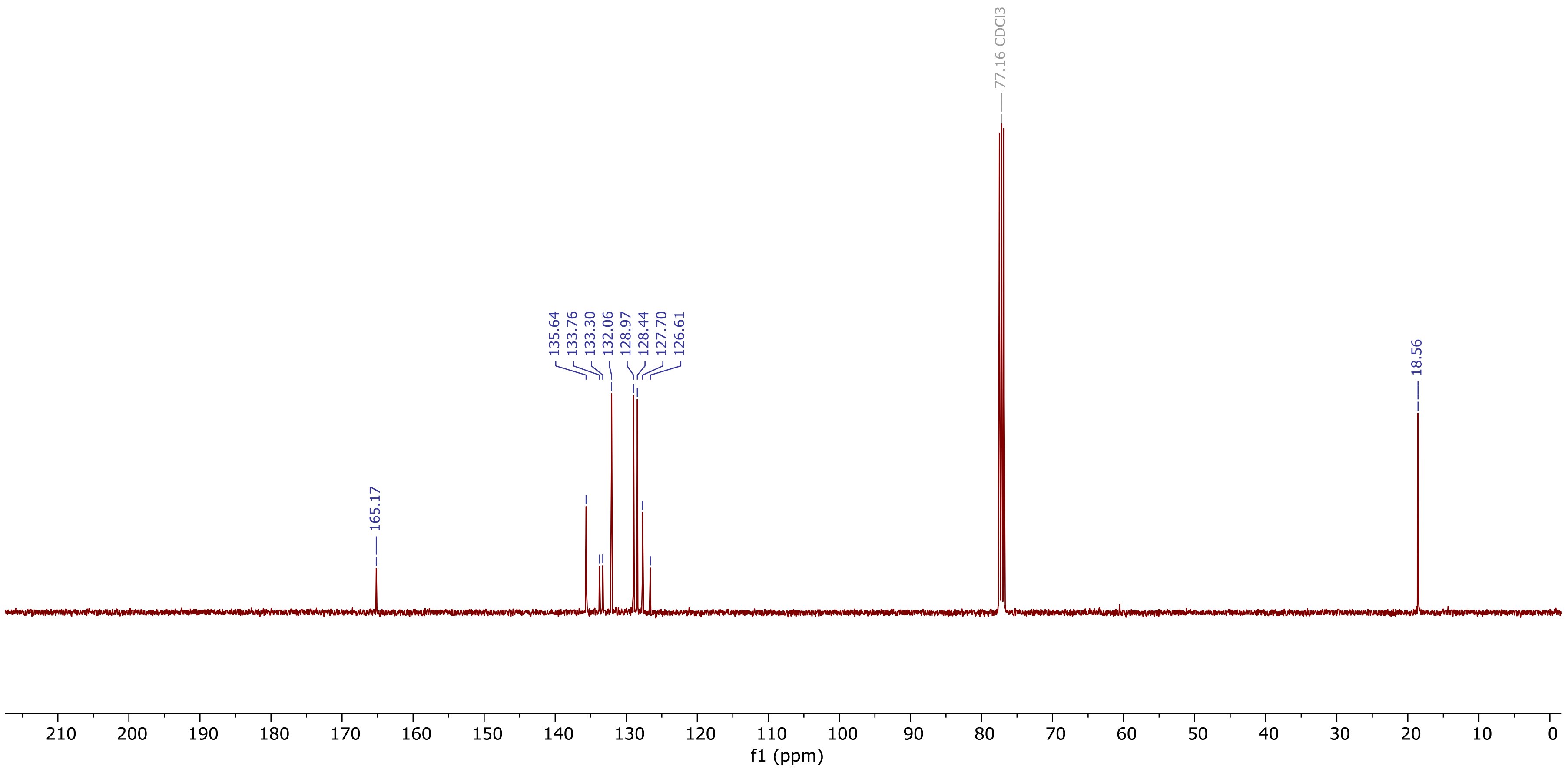

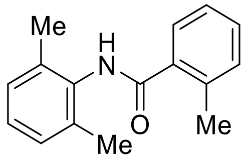

2bf, ^{1}H NMR
400 MHz, $CDCl_3$

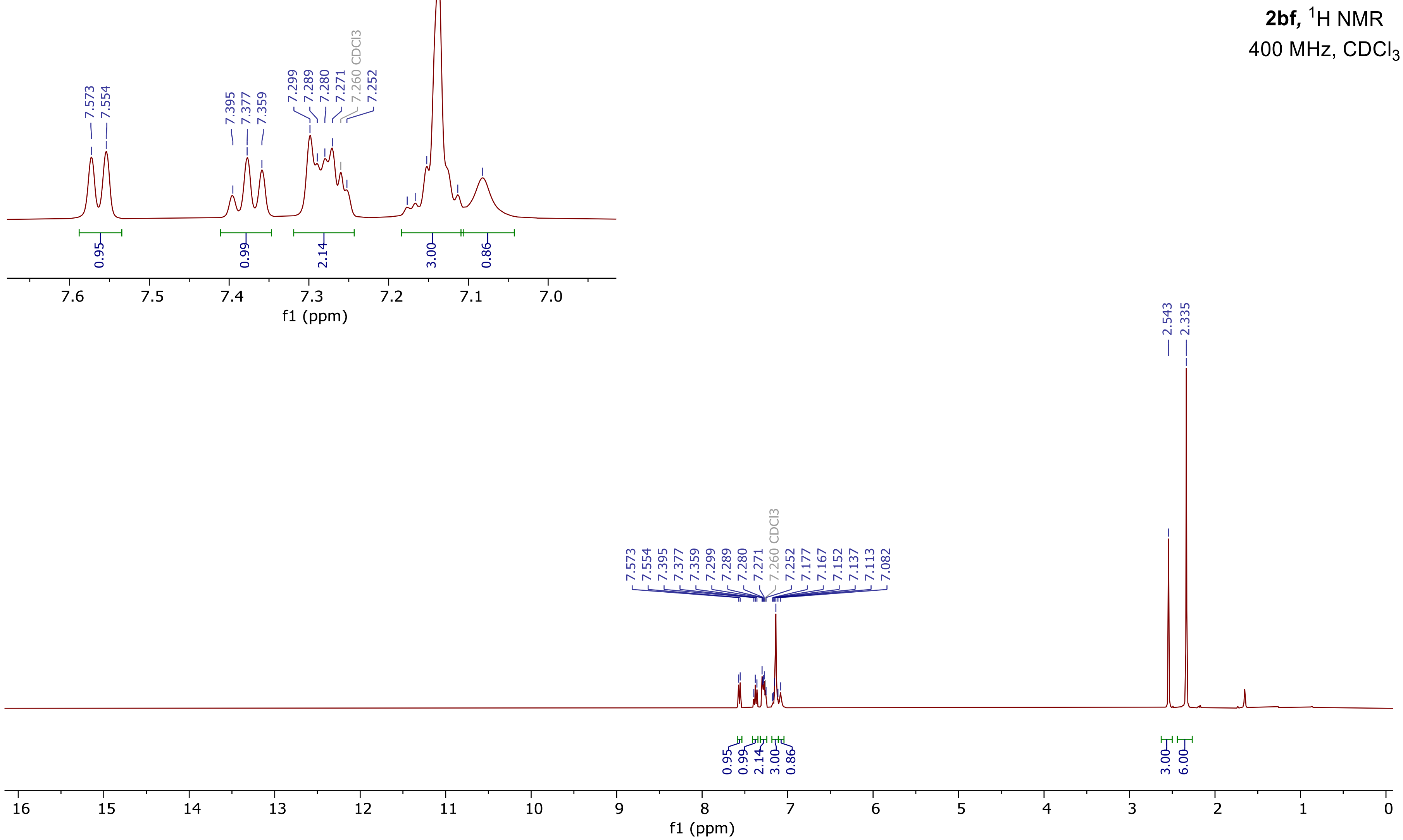

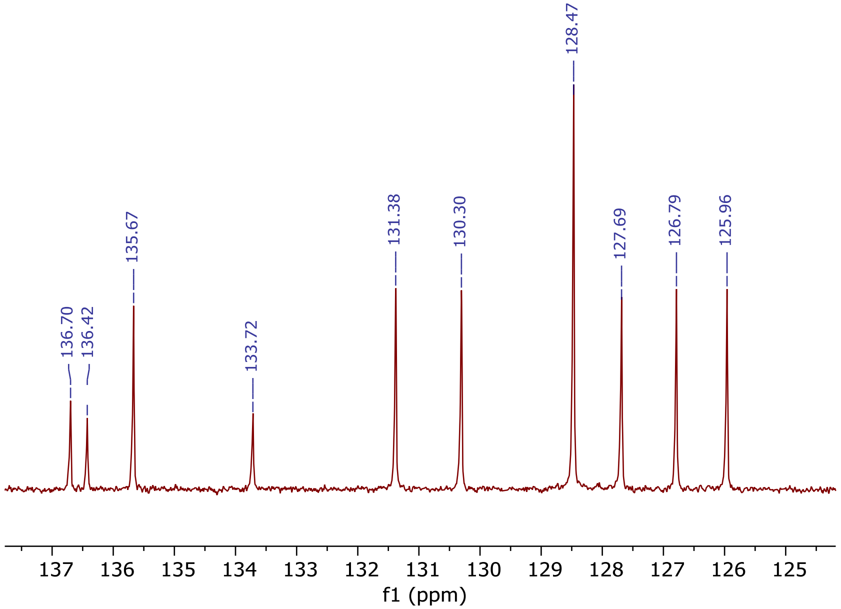

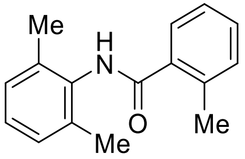

2bf, ^{13}C NMR
100 MHz, $CDCl_3$

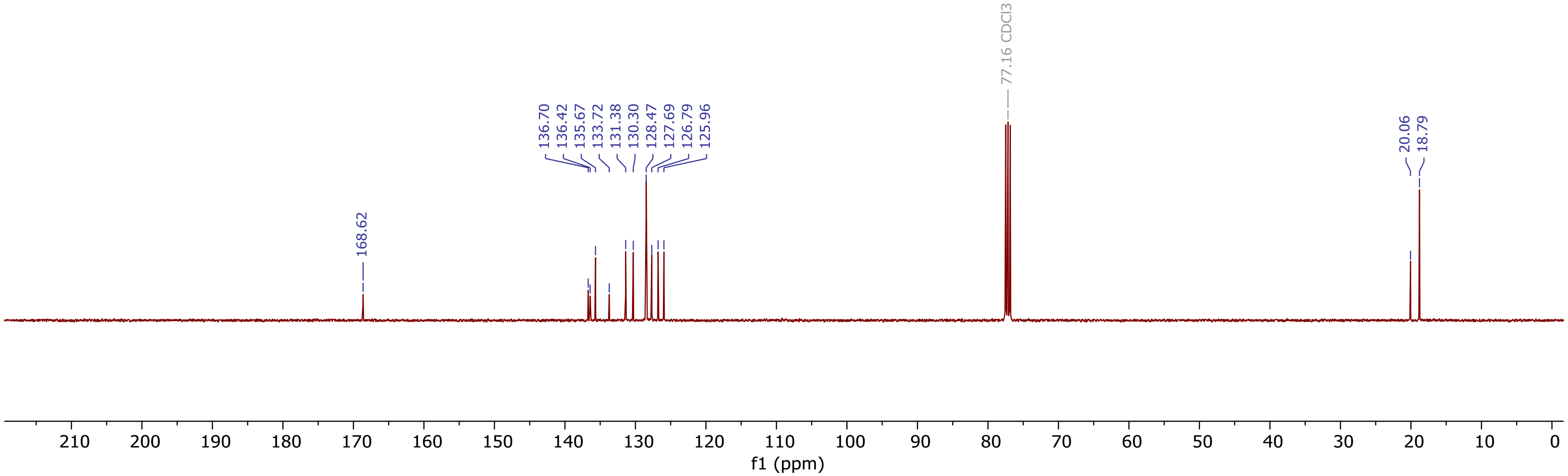

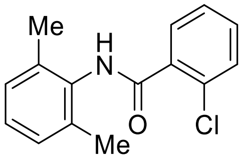

2bg, ^{1}H NMR
400 MHz, $CDCl_3$

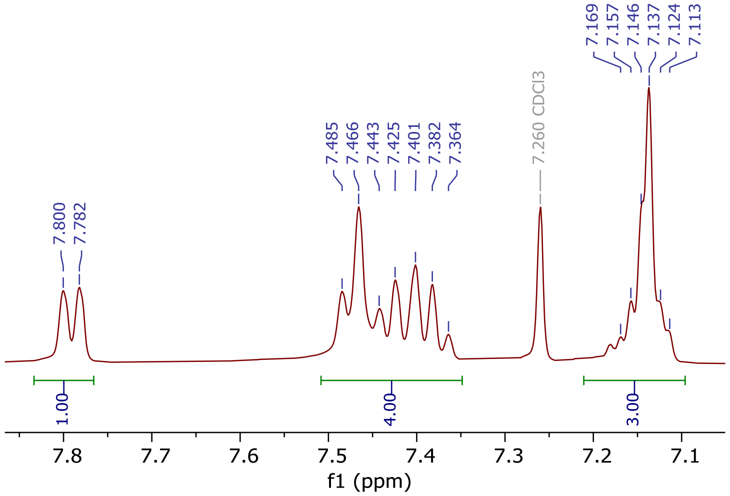

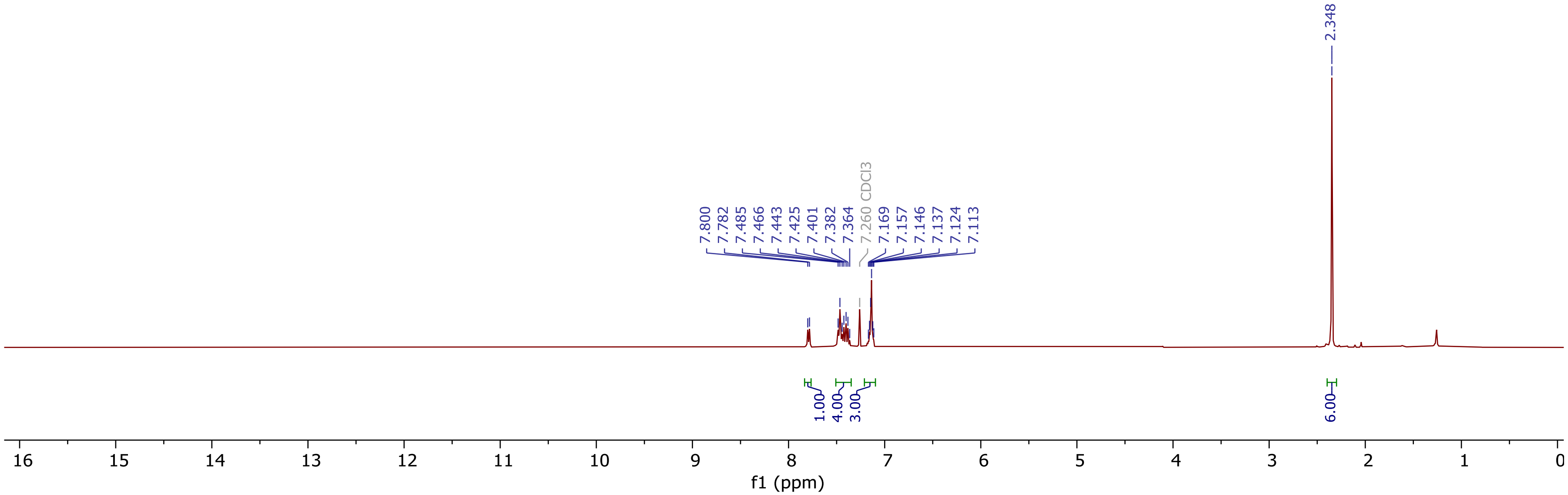

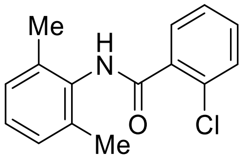

2bg, ^{13}C NMR
100 MHz, $CDCl_3$

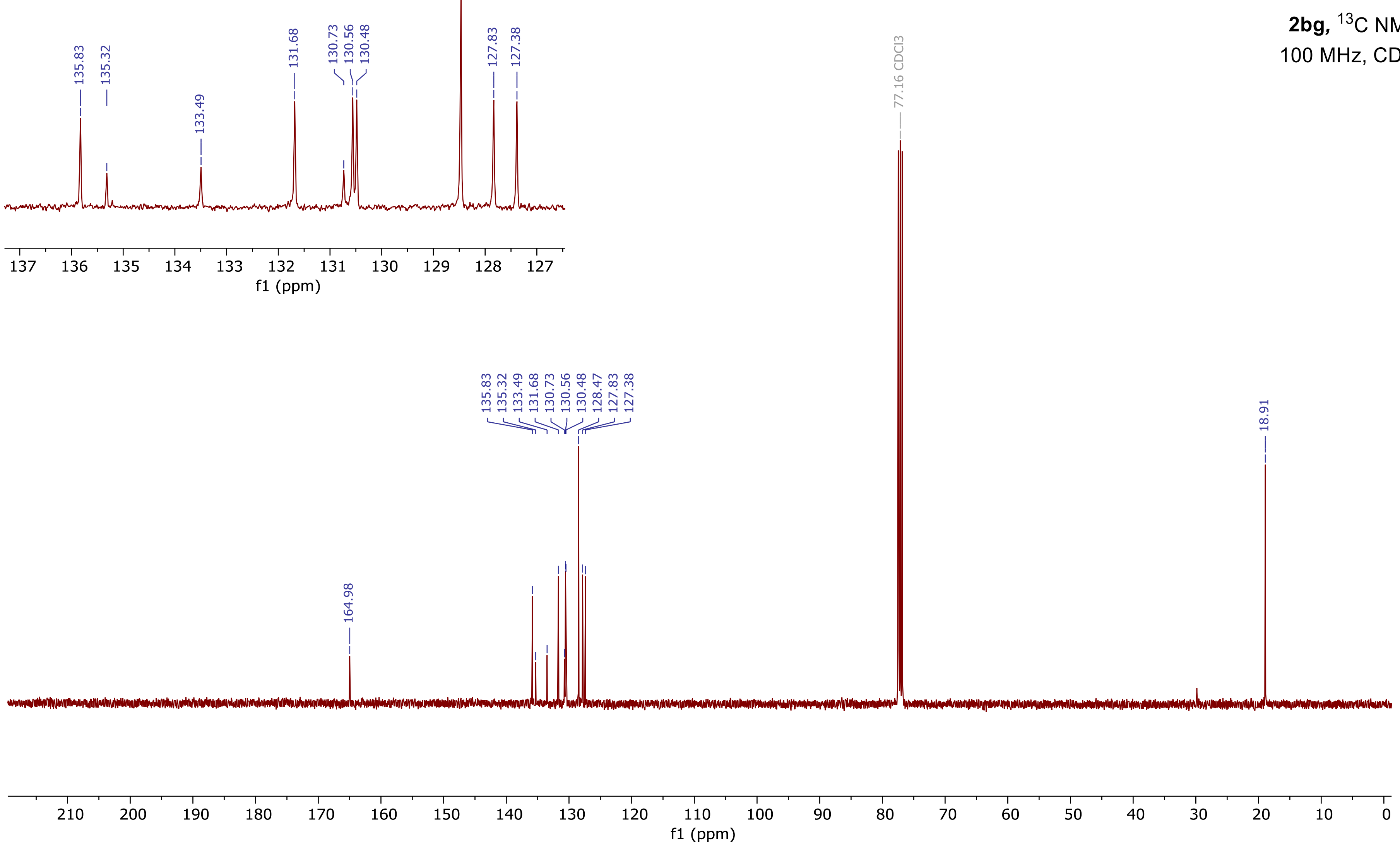

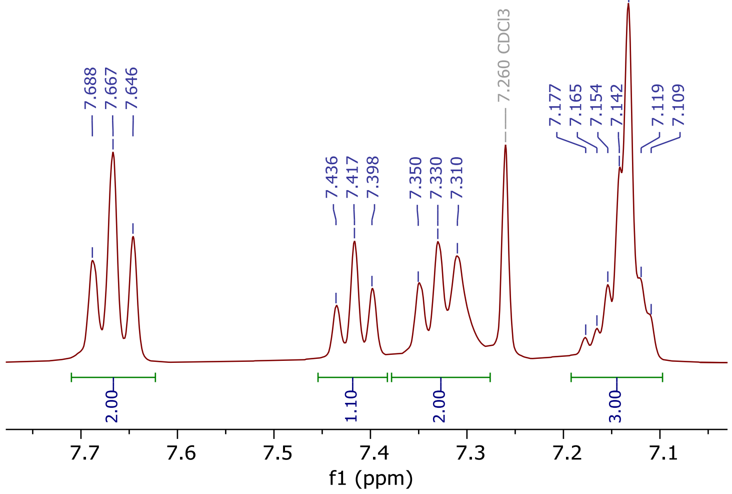

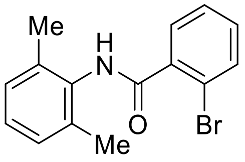

2bh, ^{1}H NMR
400 MHz, $CDCl_3$

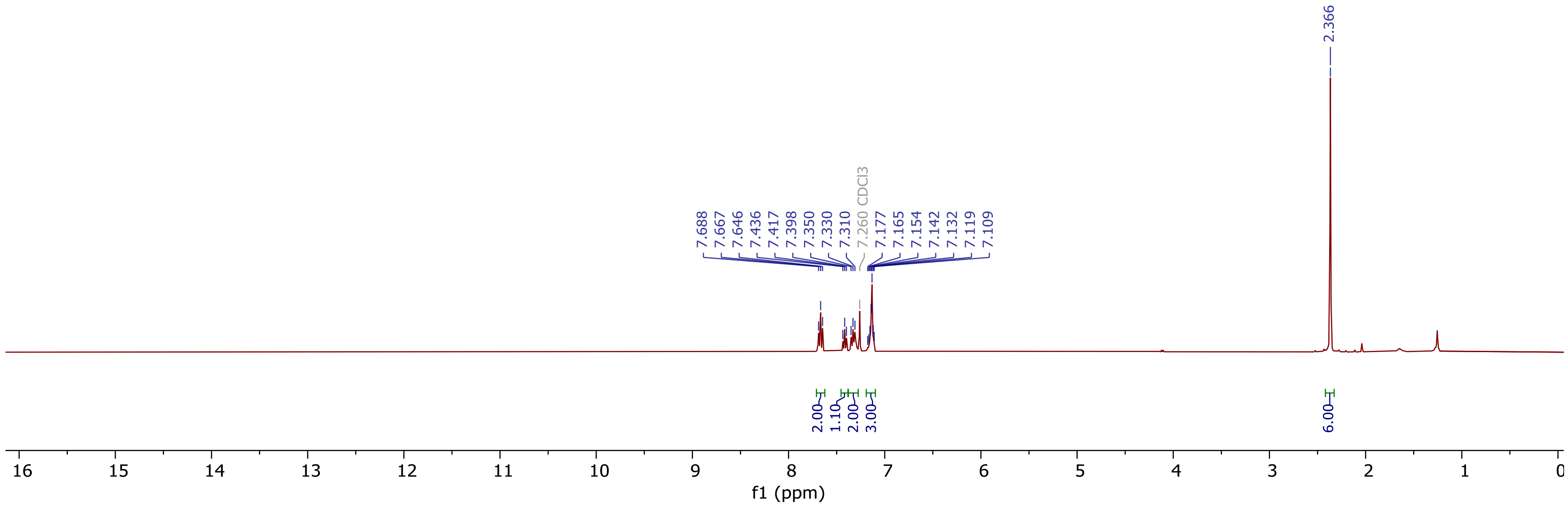

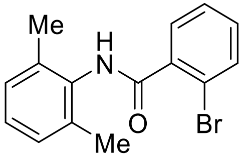

2bh, ^{13}C NMR

100 MHz, $CDCl_3$

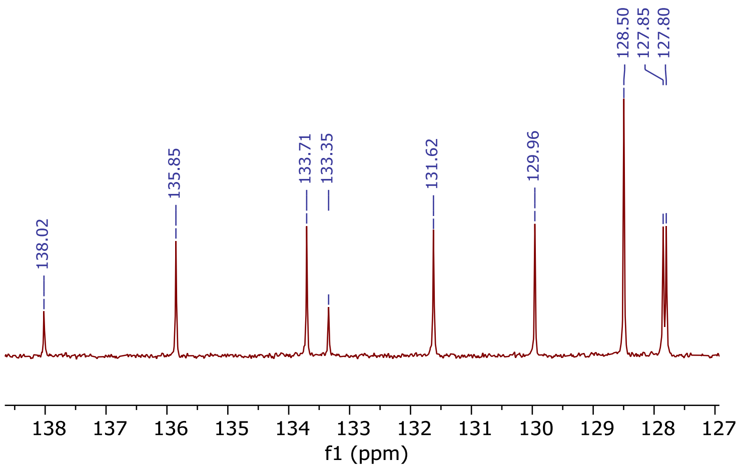

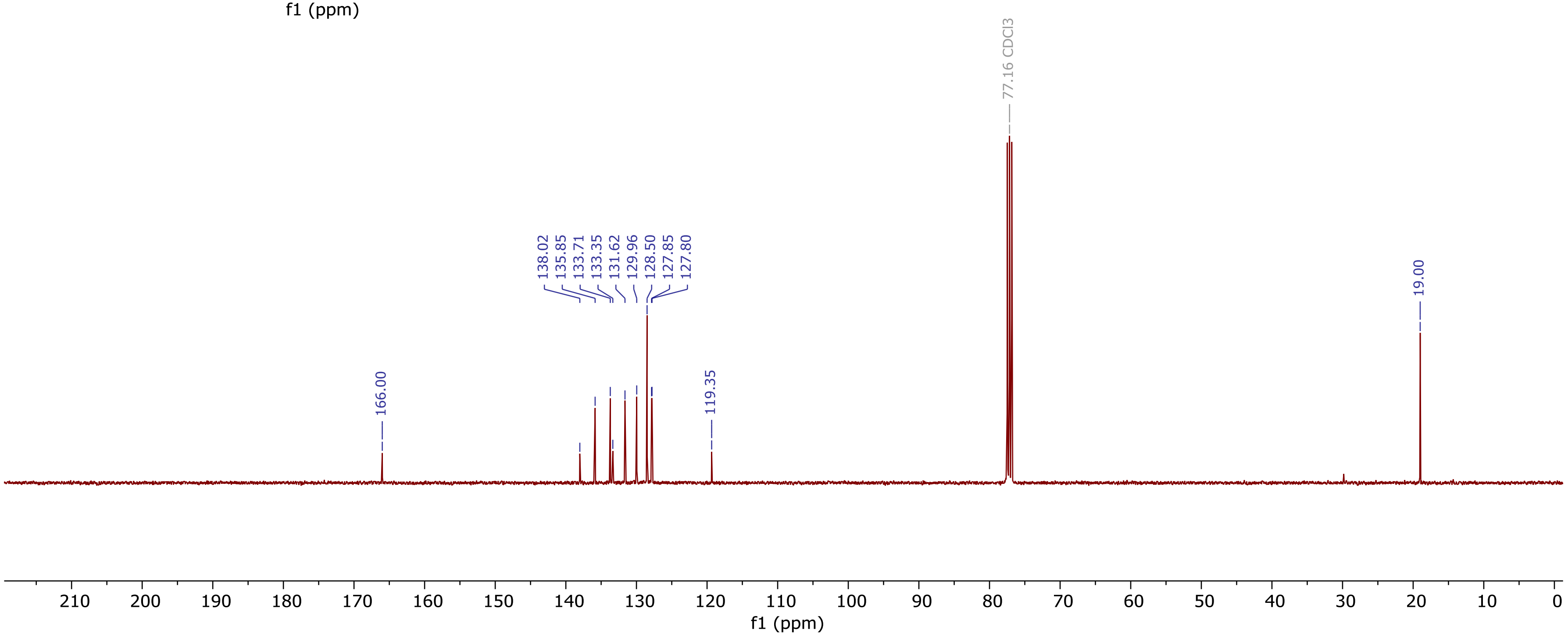

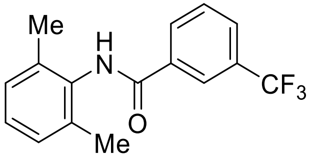

2bi, ^{1}H NMR
400 MHz, $CDCl_3$

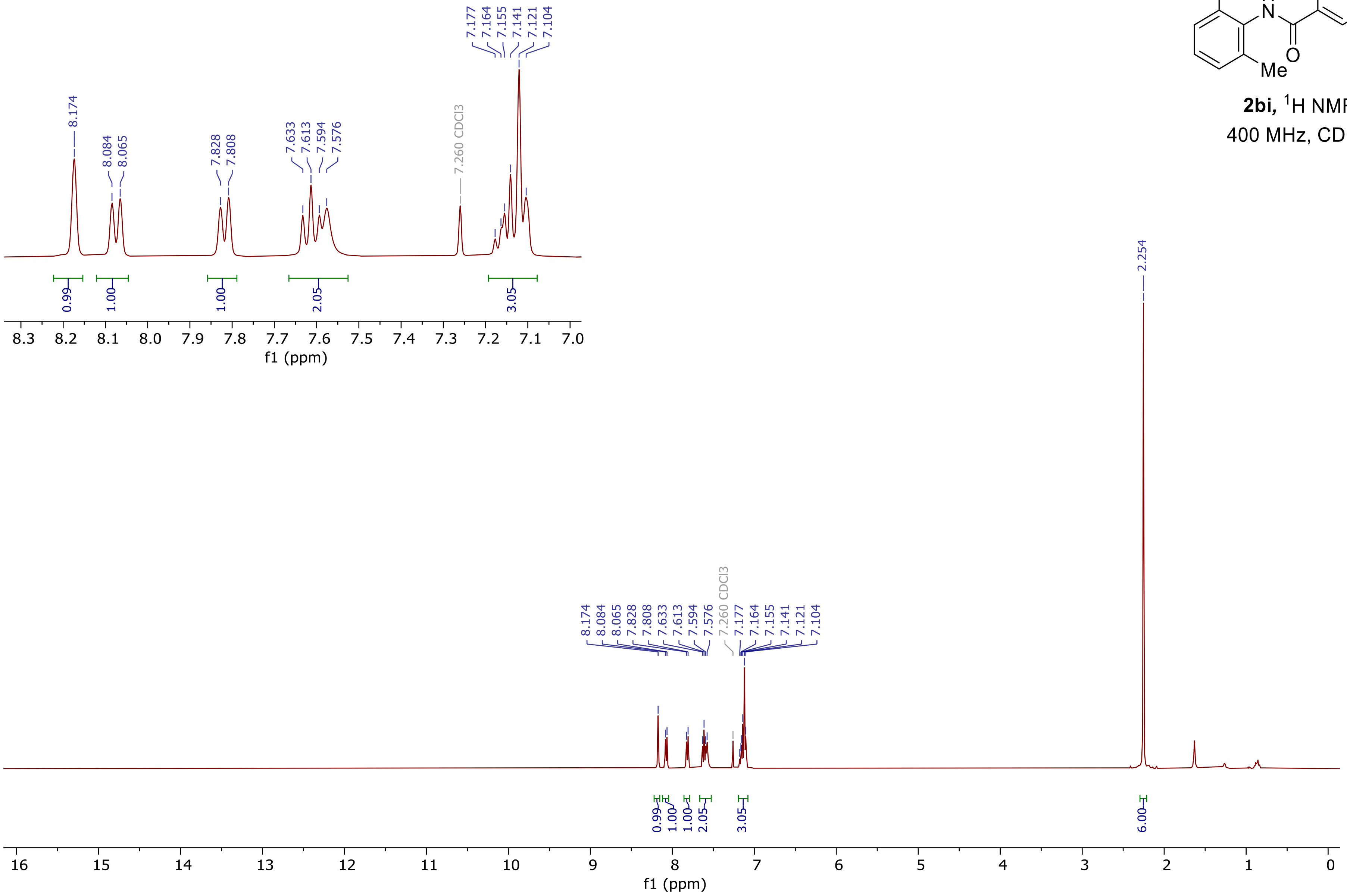

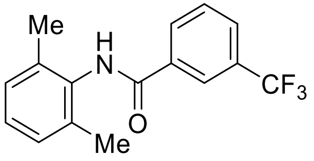

2bi, ^{13}C NMR
100 MHz, $CDCl_3$

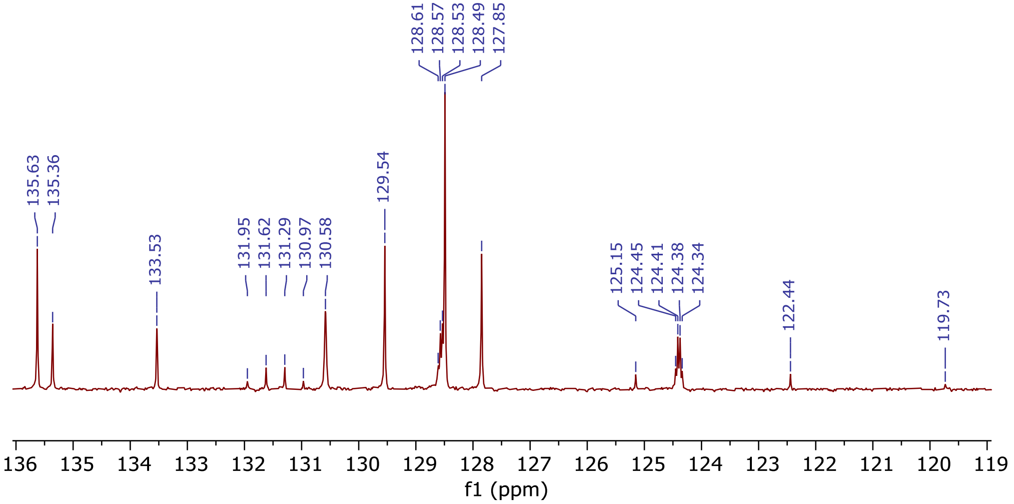

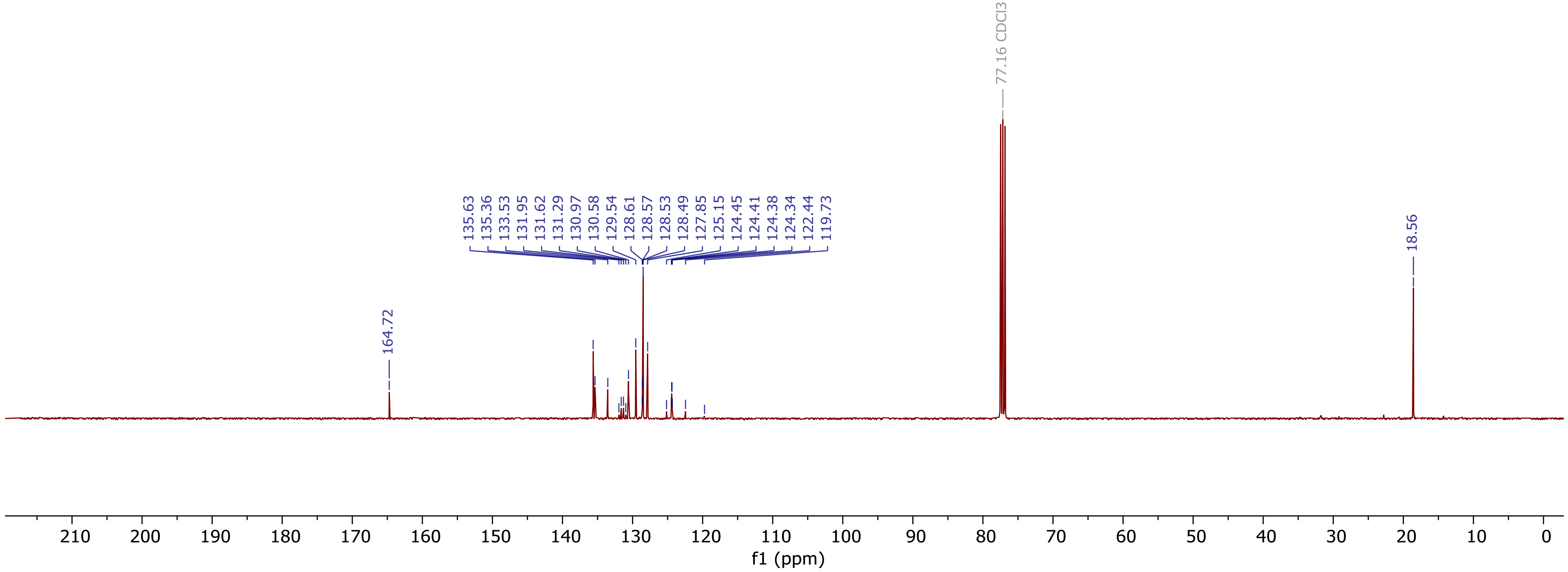

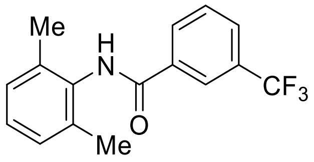

2bi, ^{19}F NMR
376 MHz, $CDCl_3$

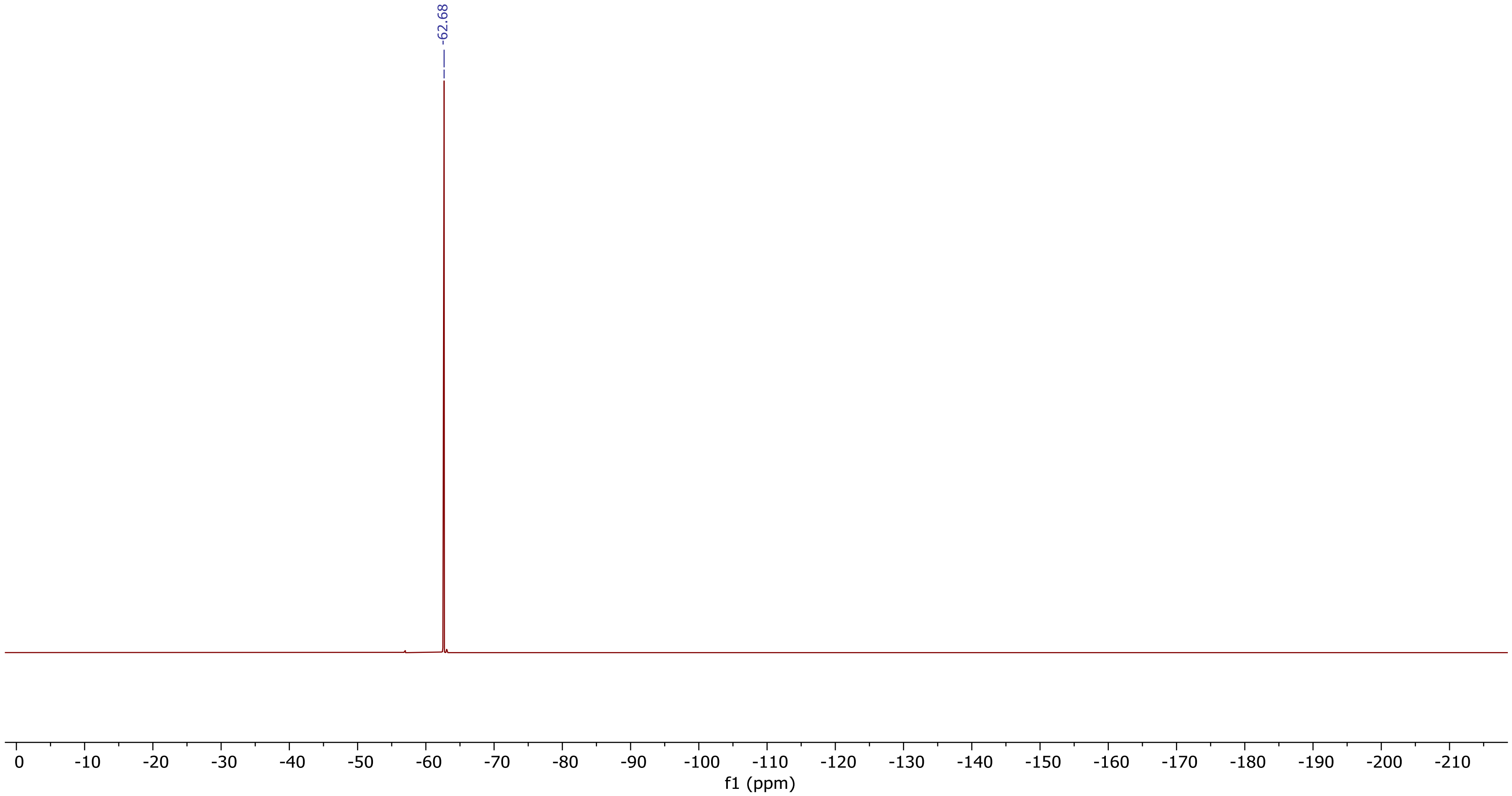

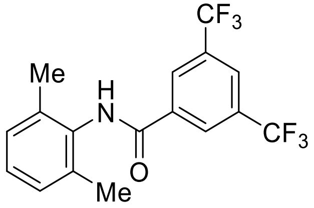

2bj, ^{1}H NMR
400 MHz, $CDCl_3$

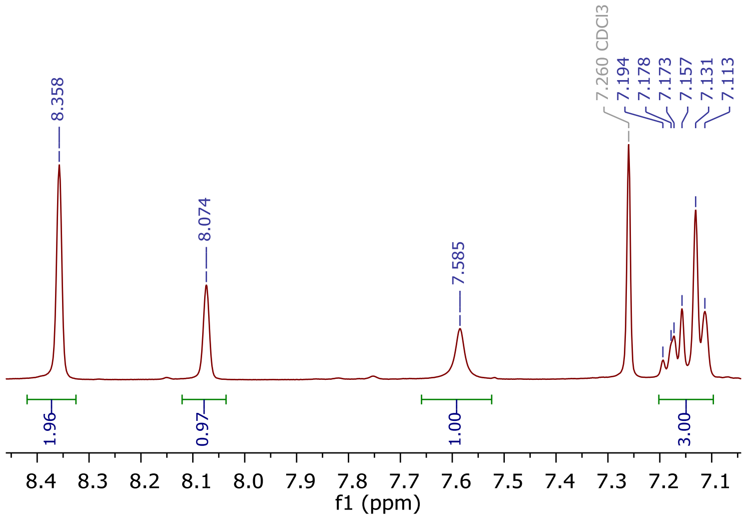

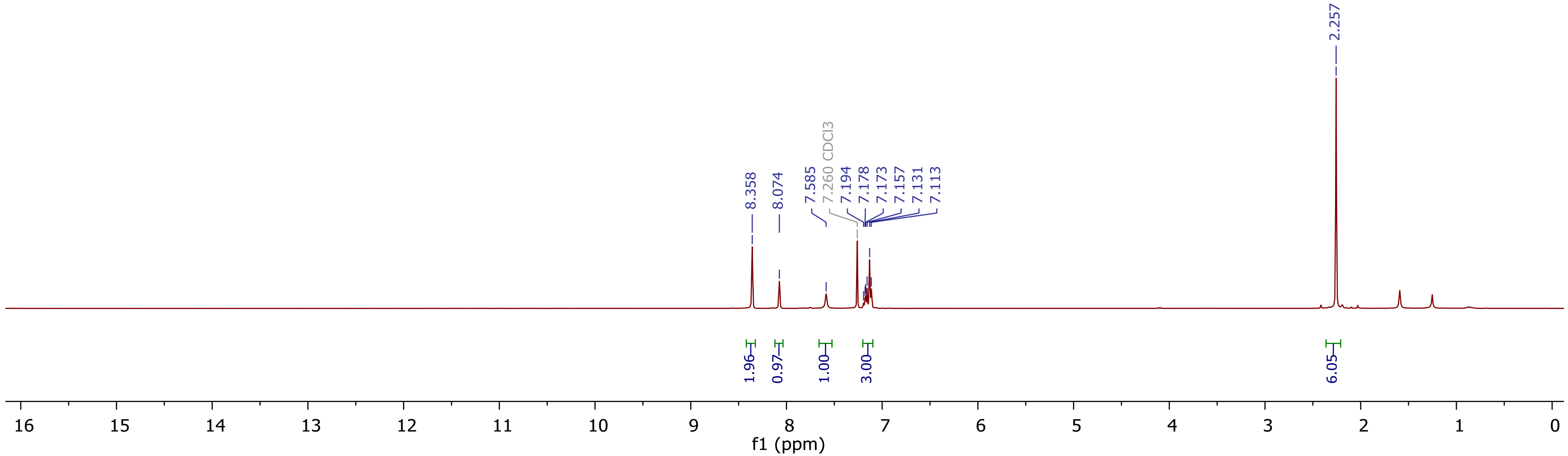

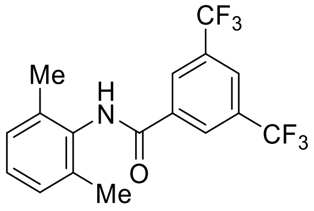

2bj, ^{13}C NMR
100 MHz, $CDCl_3$

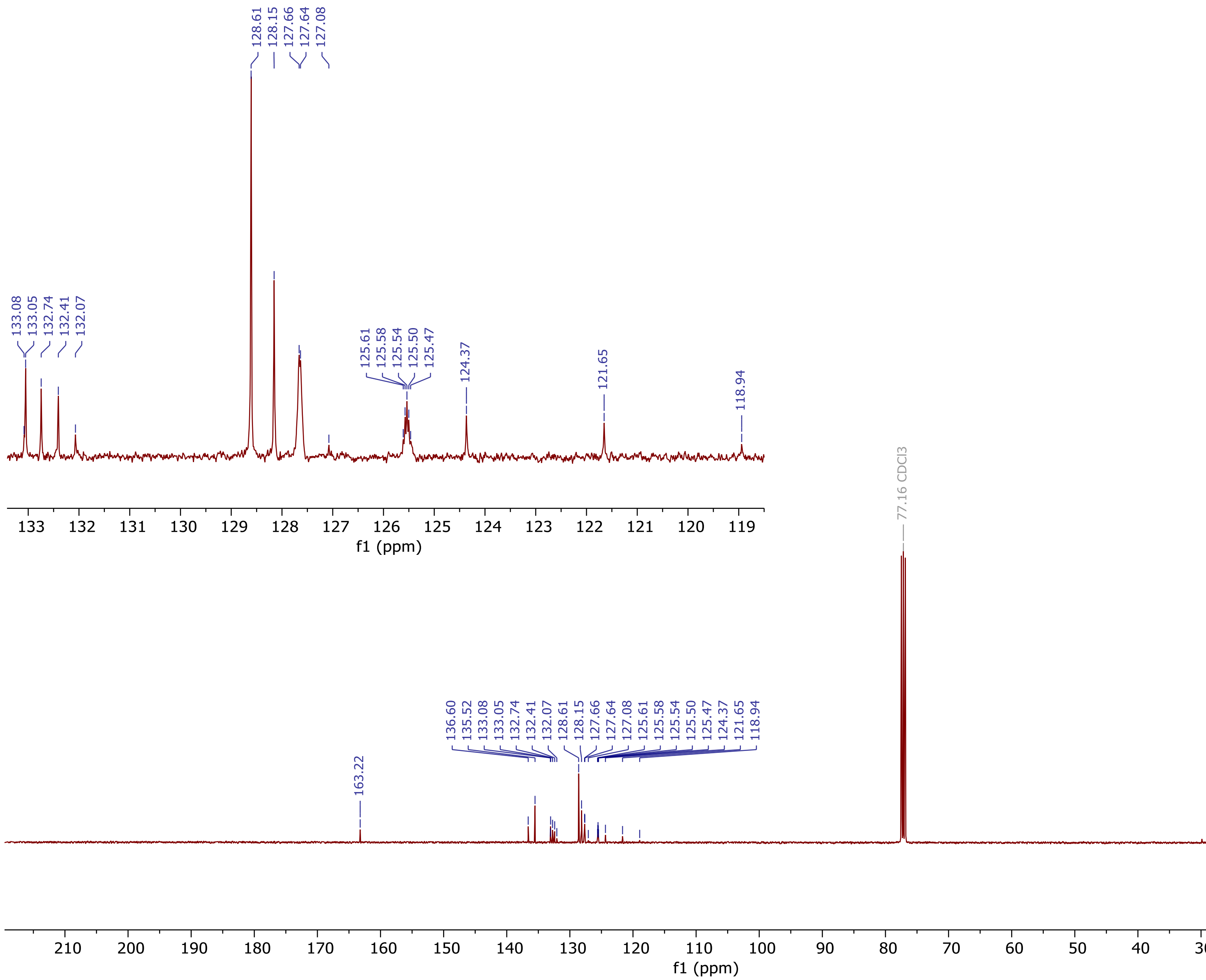

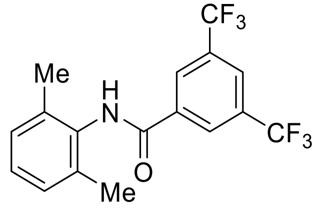

2bj, ^{19}F NMR
376 MHz, $CDCl_3$

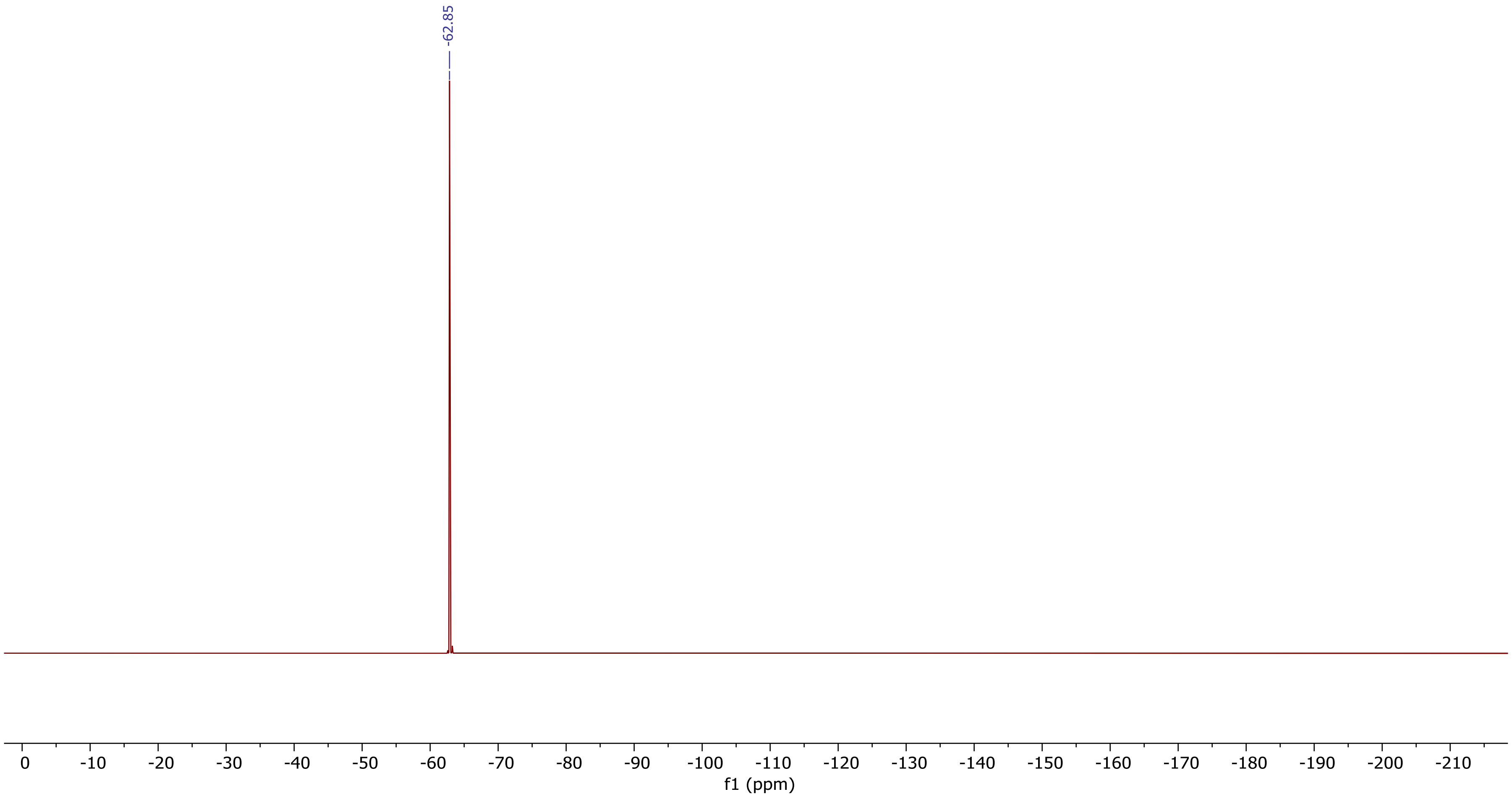

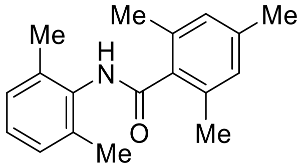

2bk, ^{1}H NMR
400 MHz, $CDCl_3$

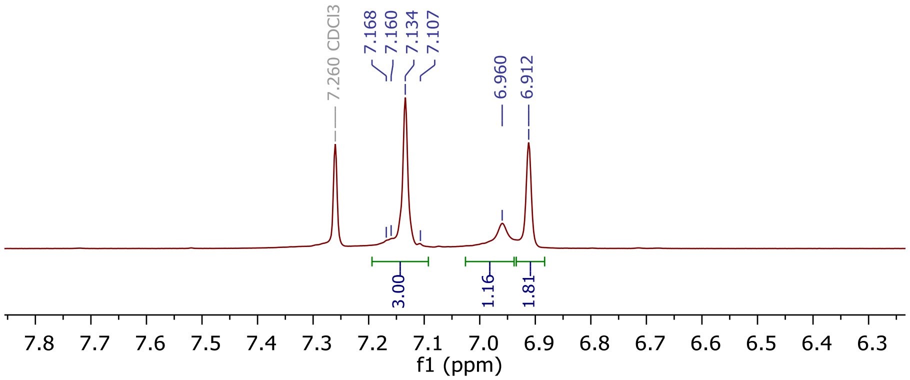

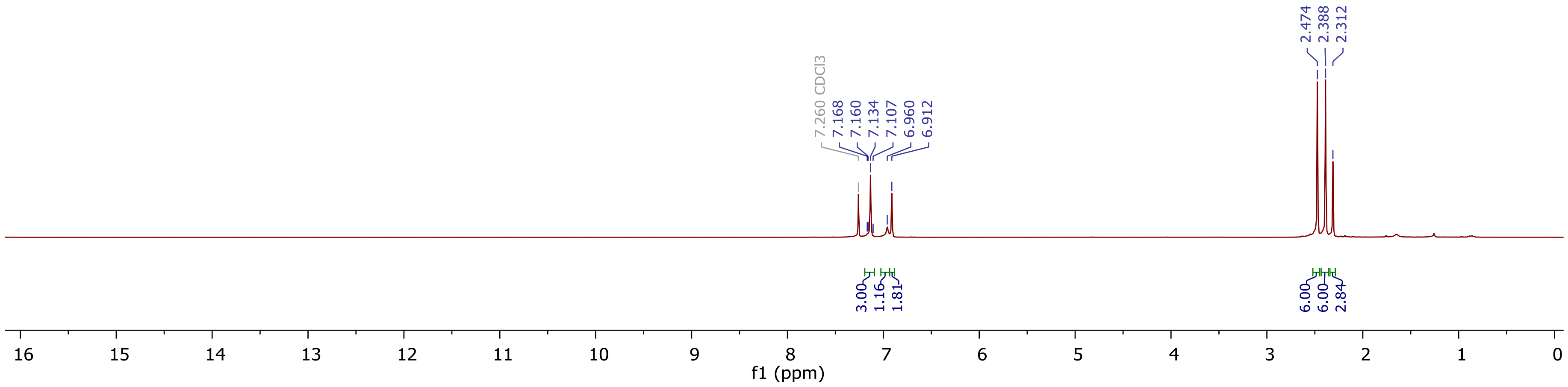

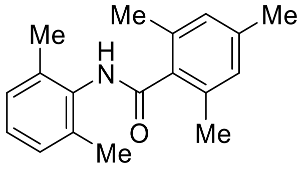

2bk, ^{13}C NMR
100 MHz, $CDCl_3$

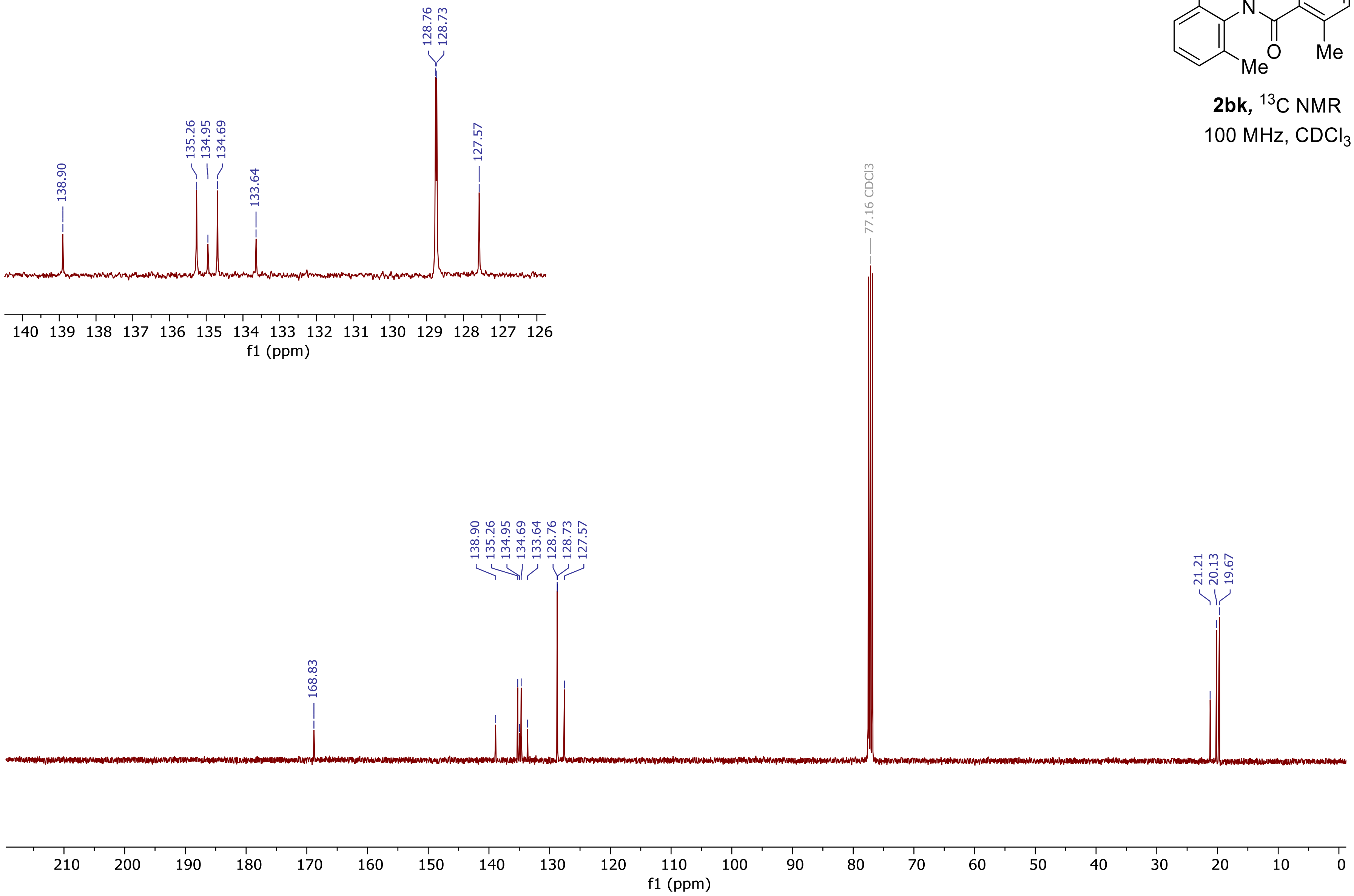

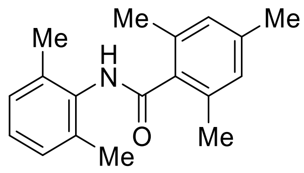

2bk, ^{1}H NMR
400 MHz, DMSO-d_6

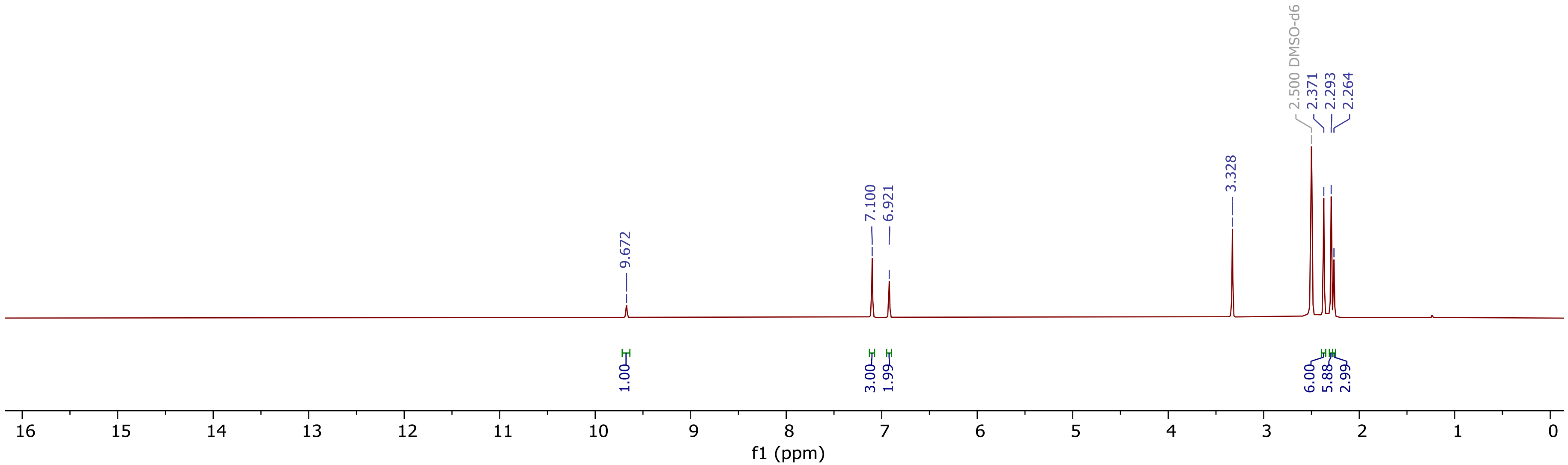

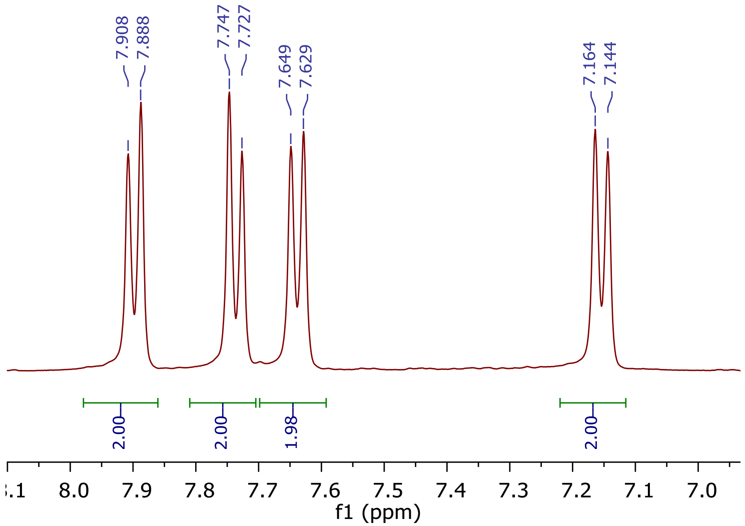

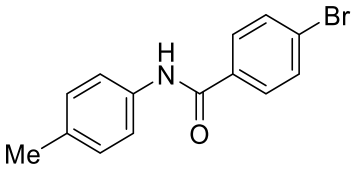

2ce, ^{1}H NMR
400 MHz, DMSO-d_6

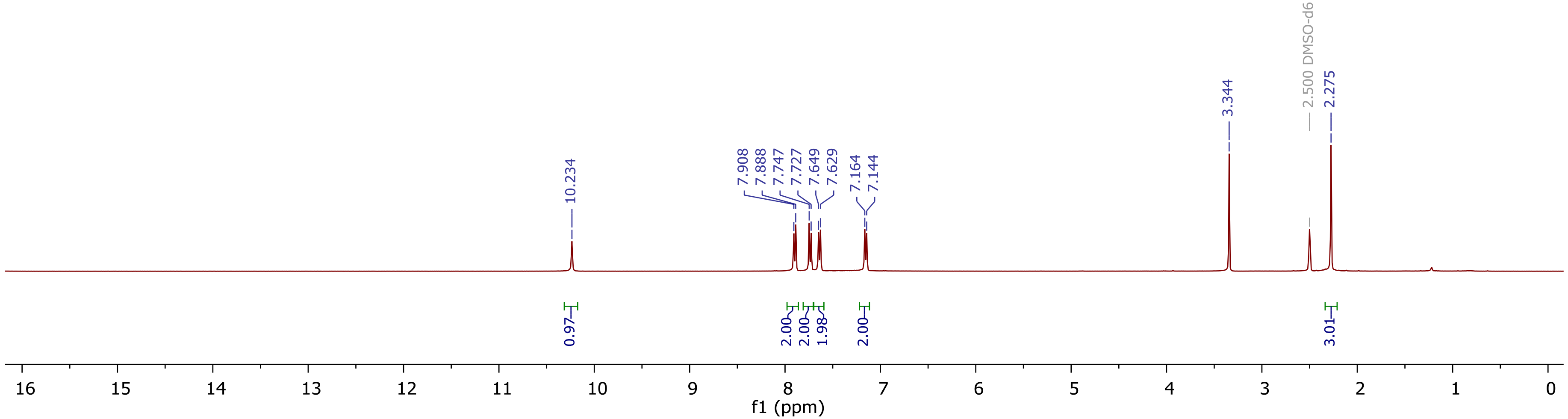

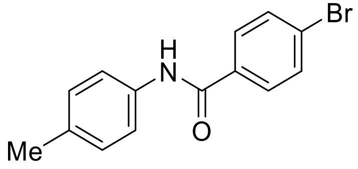

2ce, ^{13}C NMR
100 MHz, DMSO-d_6

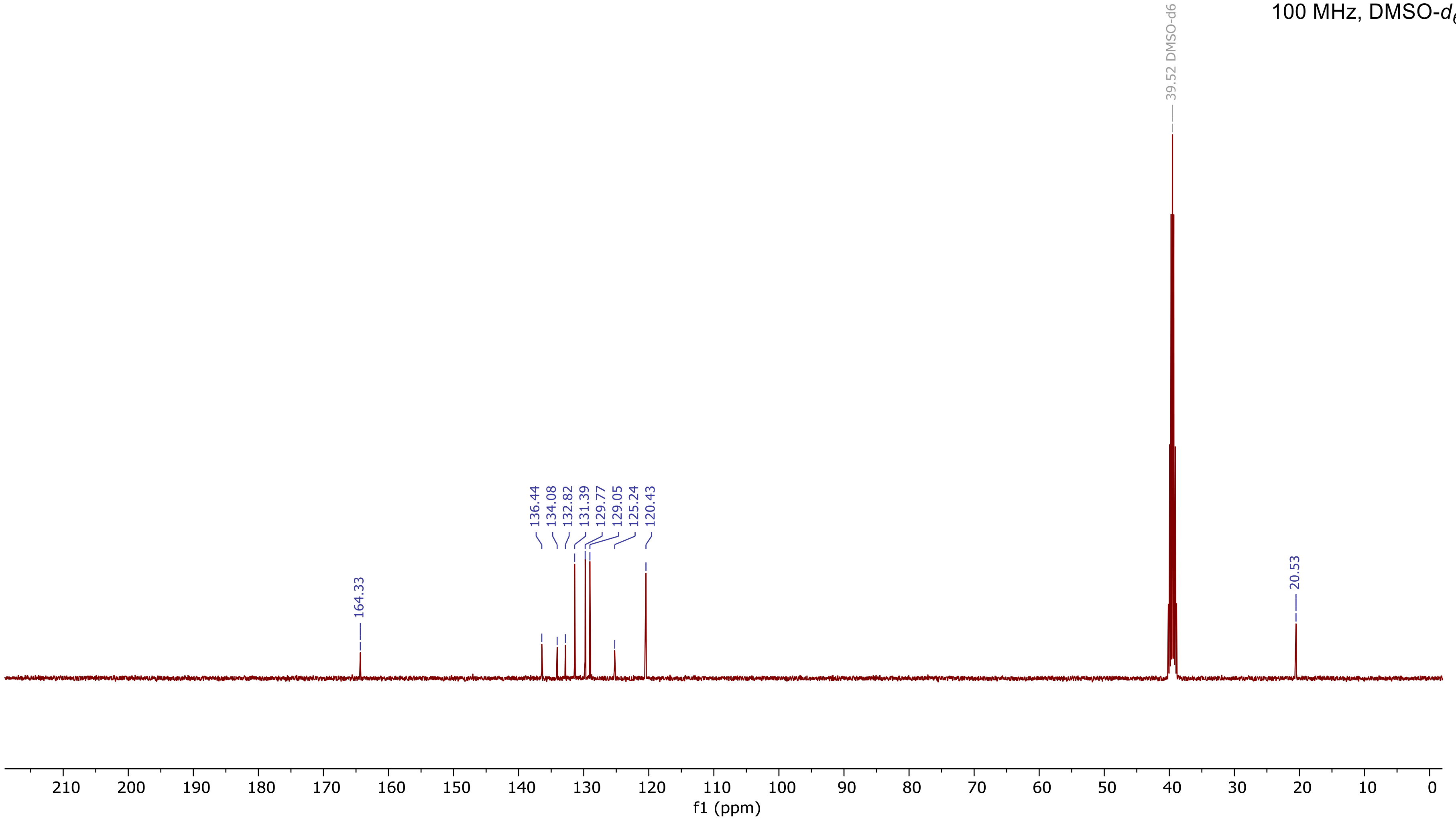

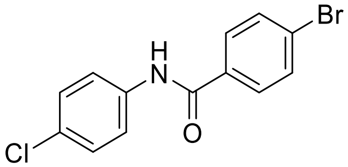

2de, ^{1}H NMR

400 MHz, $CDCl_3$

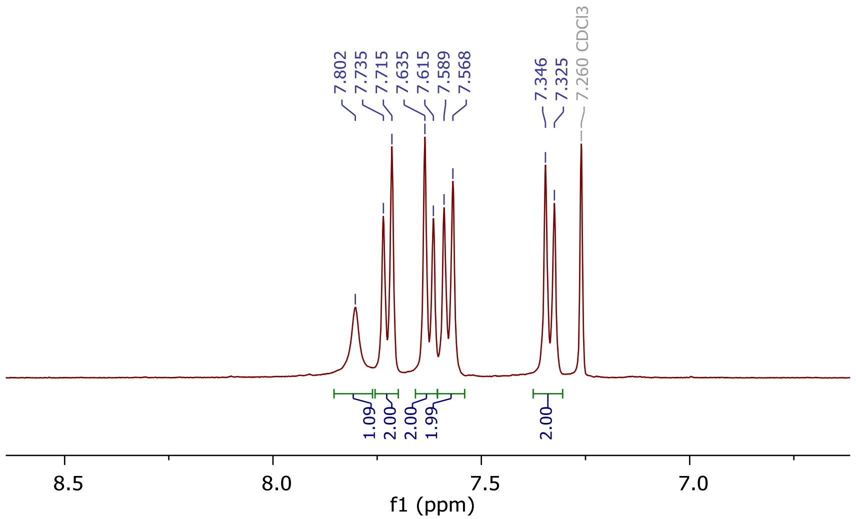

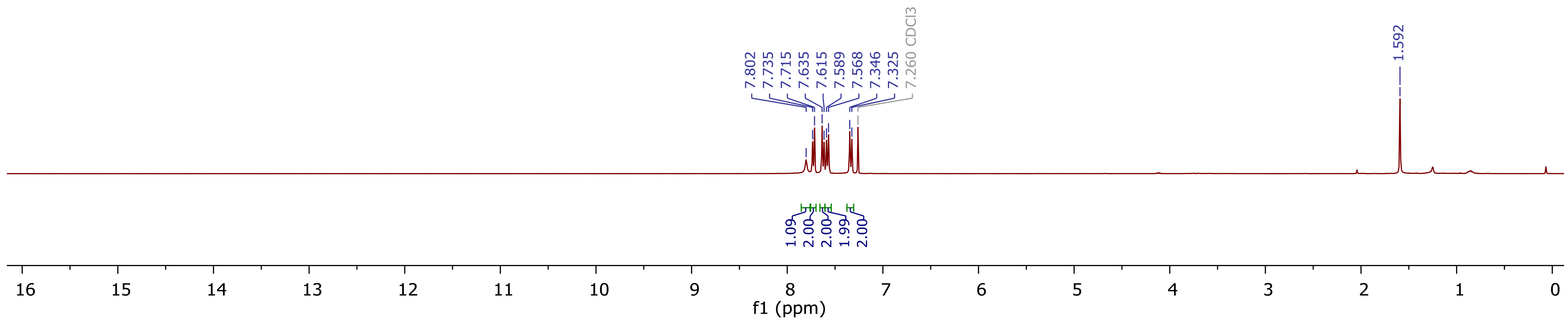

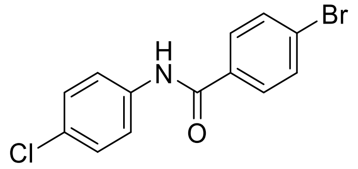

2de, ^{13}C NMR
100 MHz, $CDCl_3$

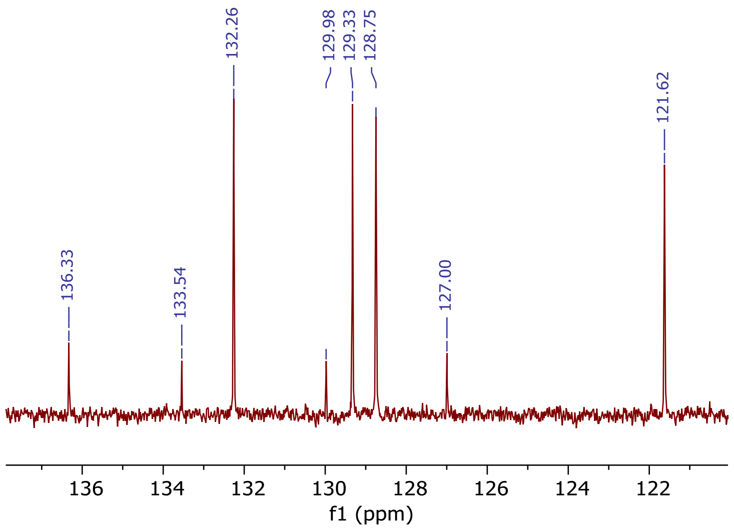

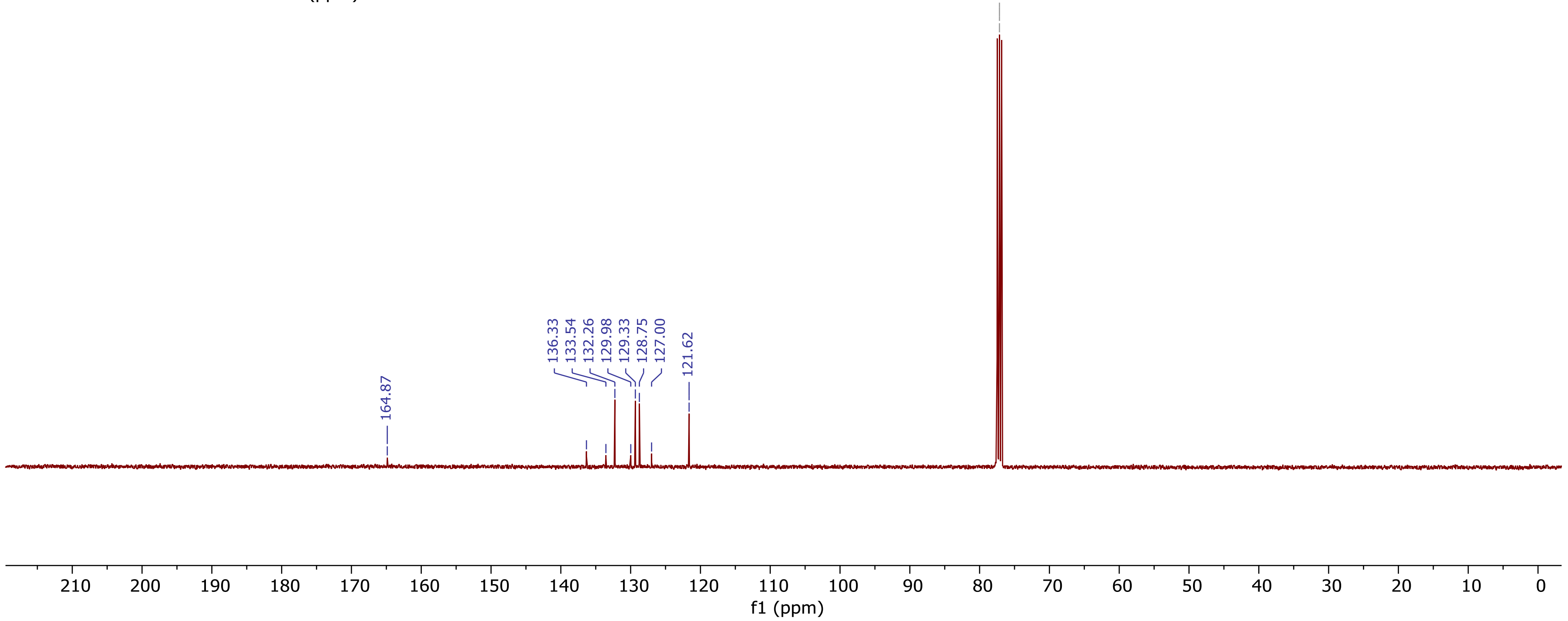

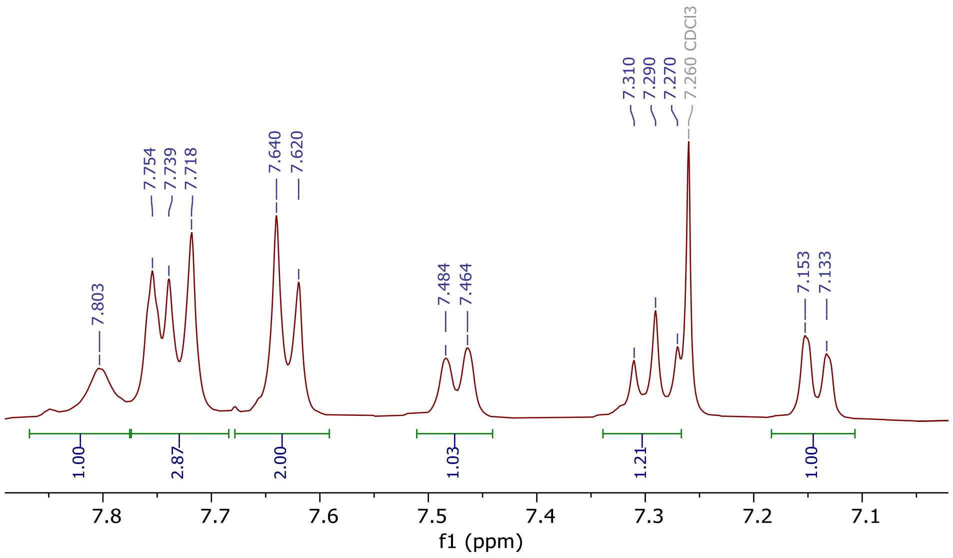

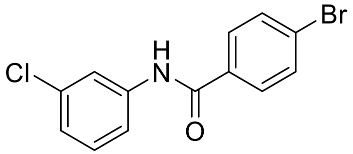

2ee, ^{1}H NMR
400 MHz, $CDCl_3$

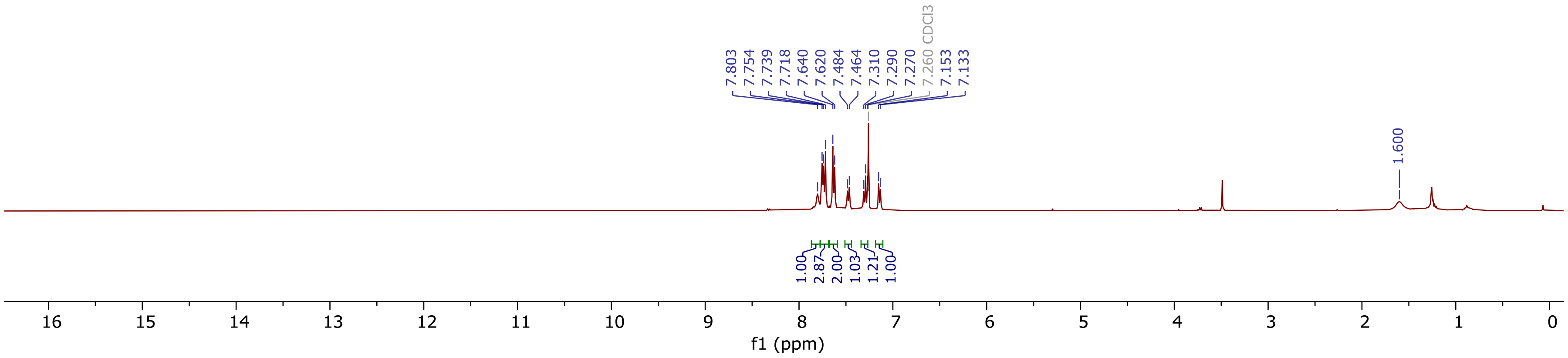

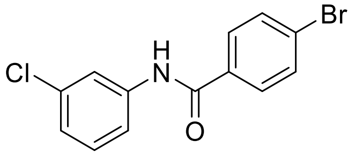

2ee, ^{13}C NMR
100 MHz, $CDCl_3$

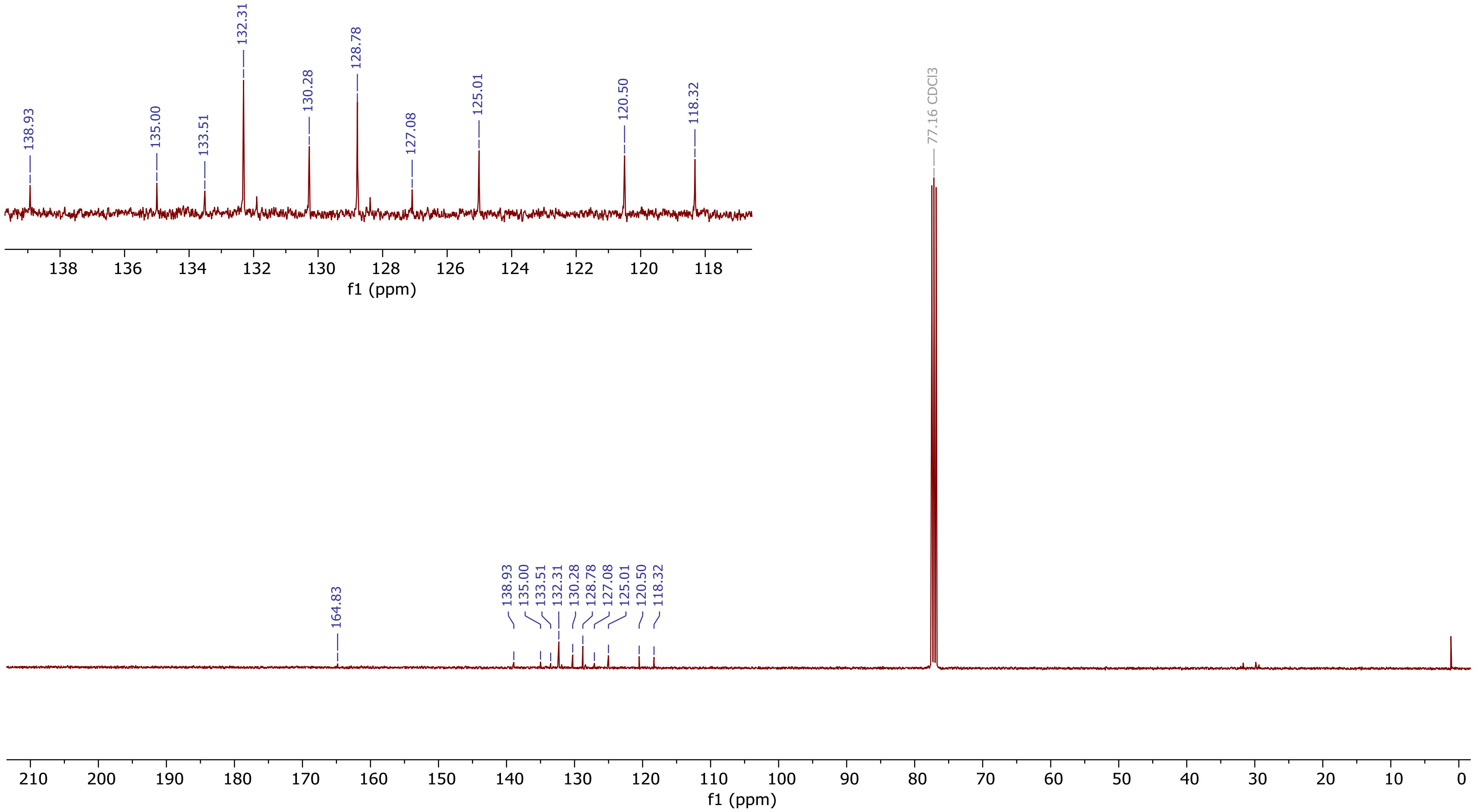

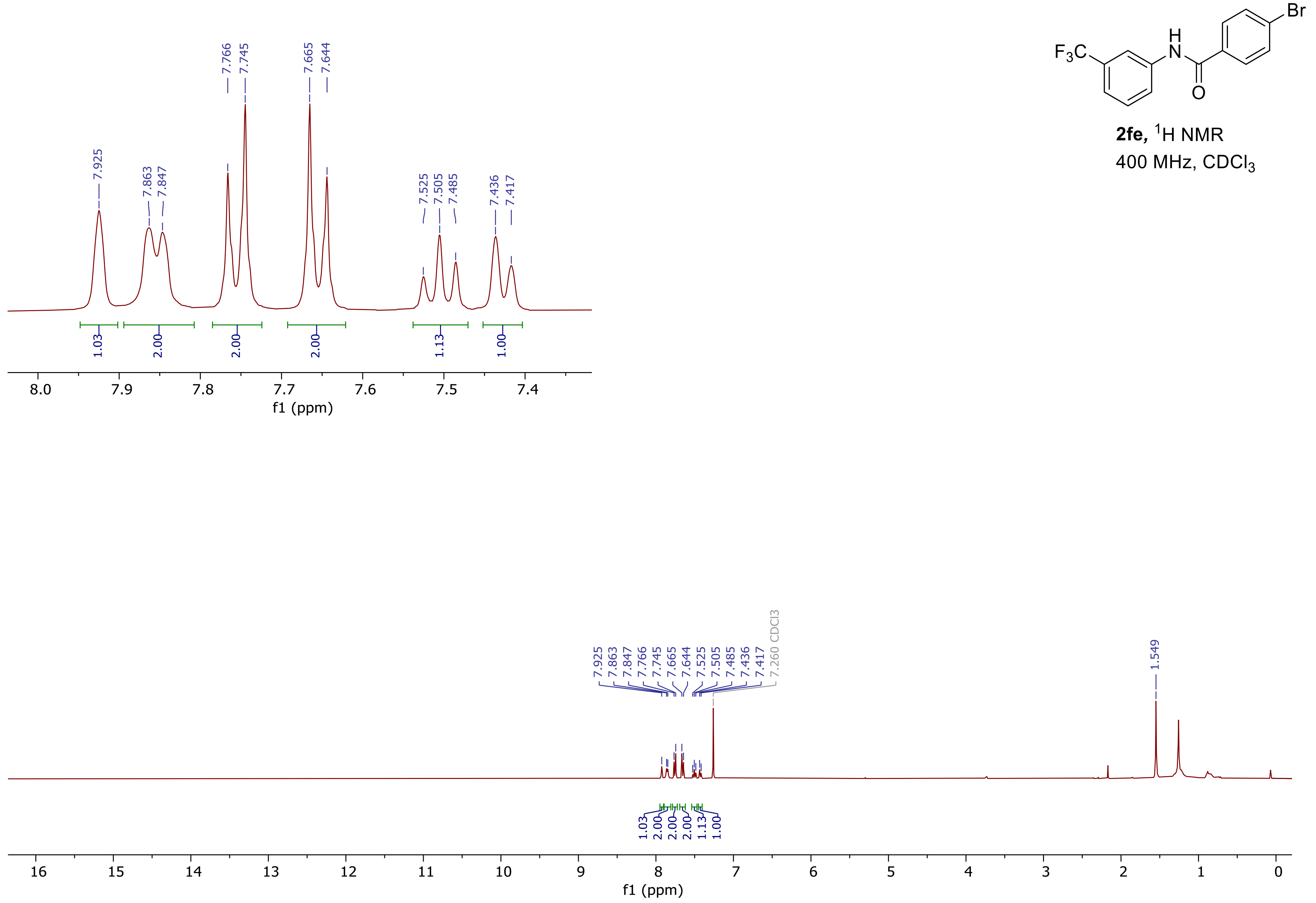
Br
H
N
F3C
O
2fe, 1H NMR
400 MHz, CDCl3
7.925
7.863
7.847
7.766
7.745
7.665
7.644
7.525
7.505
7.485
7.436
7.417
7.260 CDCl3
1.549
1.03
2.00
2.00
2.00
1.13
1.00
f1 (ppm)

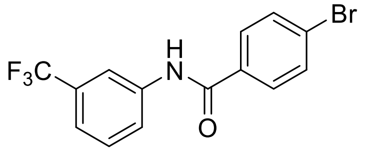

2fe, ^{13}C NMR
125 MHz, $CDCl_3$

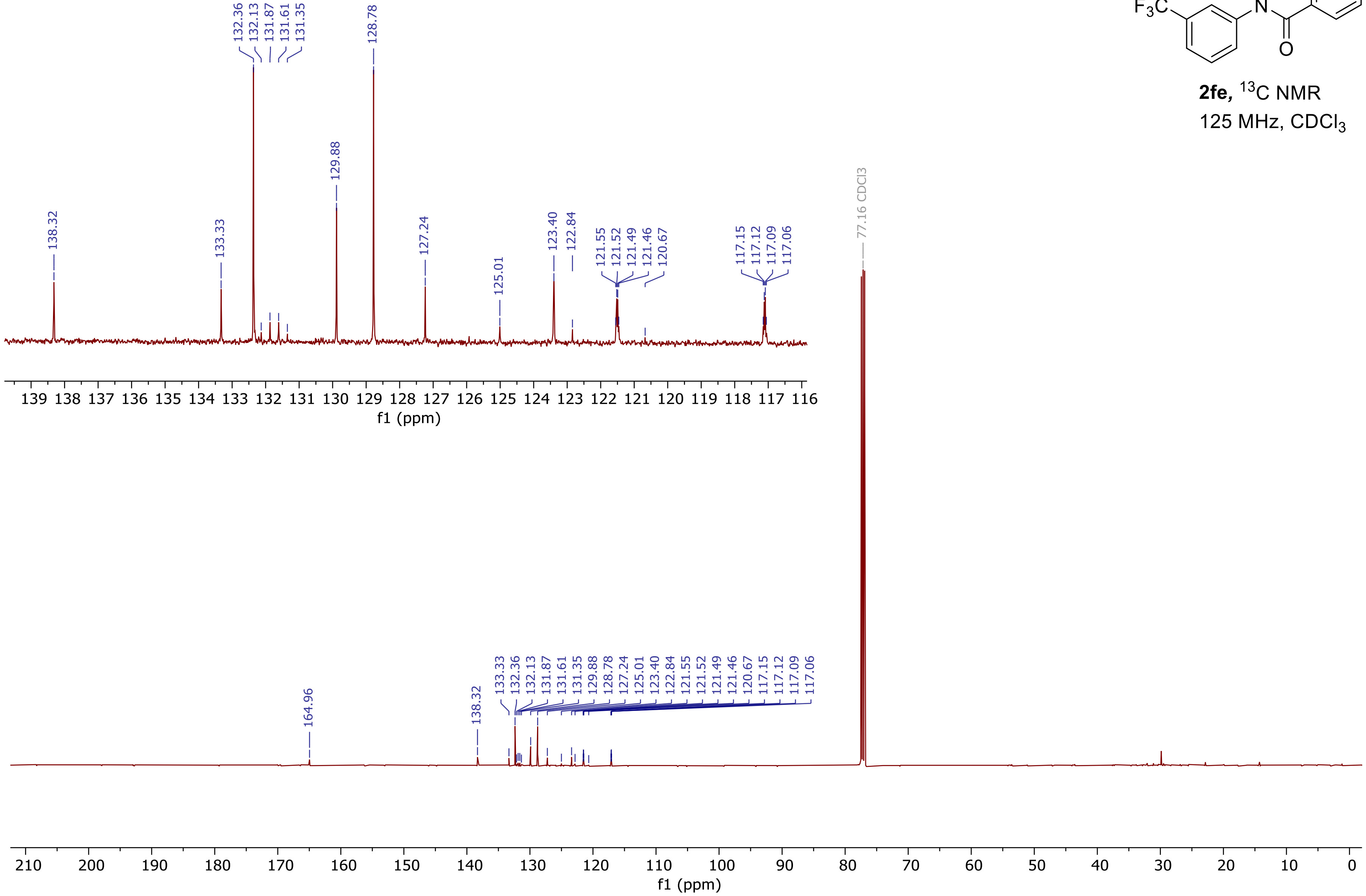

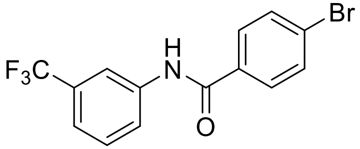

2fe, ^{19}F NMR
376 MHz, $CDCl_3$

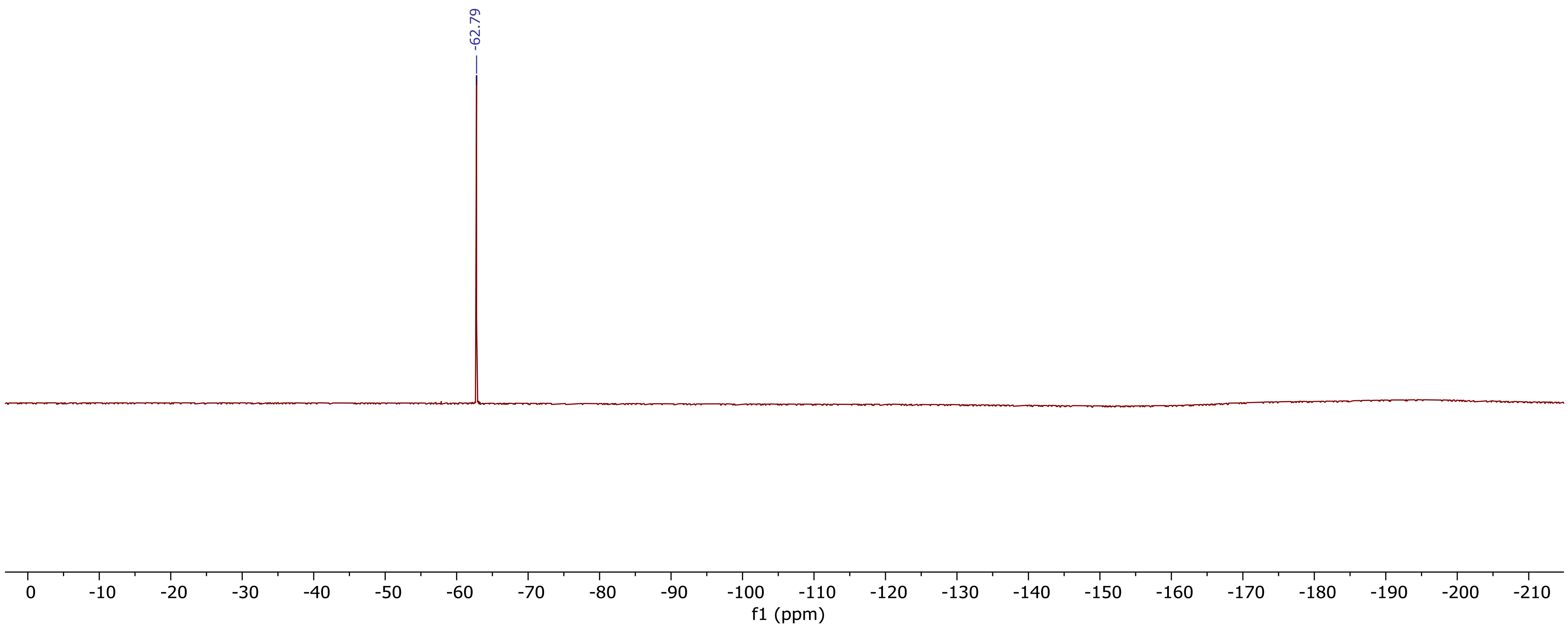

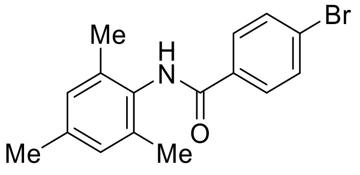

2ge, ^{1}H NMR
400 MHz, $CDCl_3$

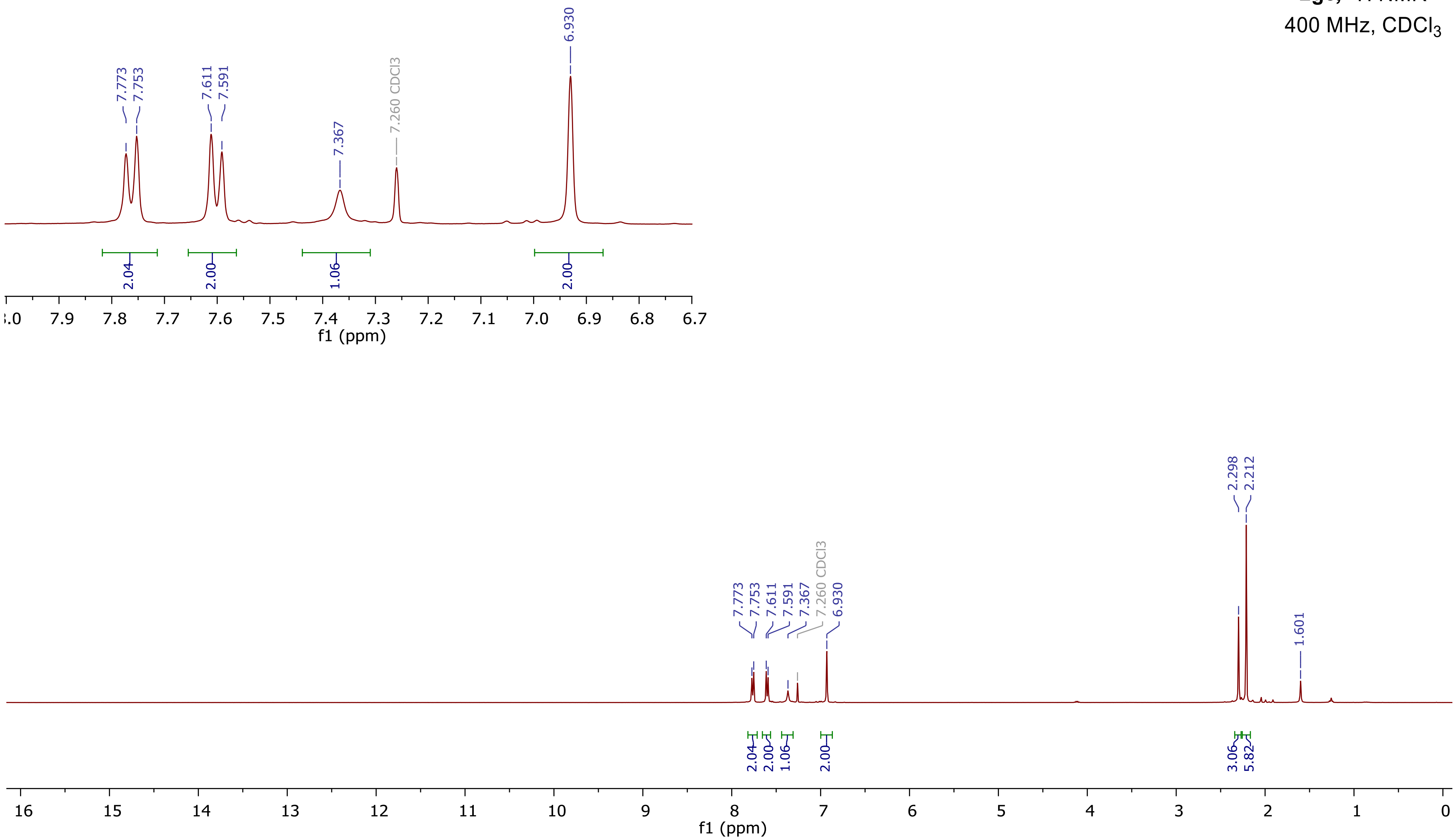

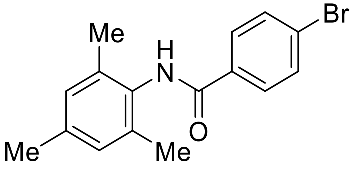

2ge, ^{13}C NMR
100 MHz, $CDCl_3$

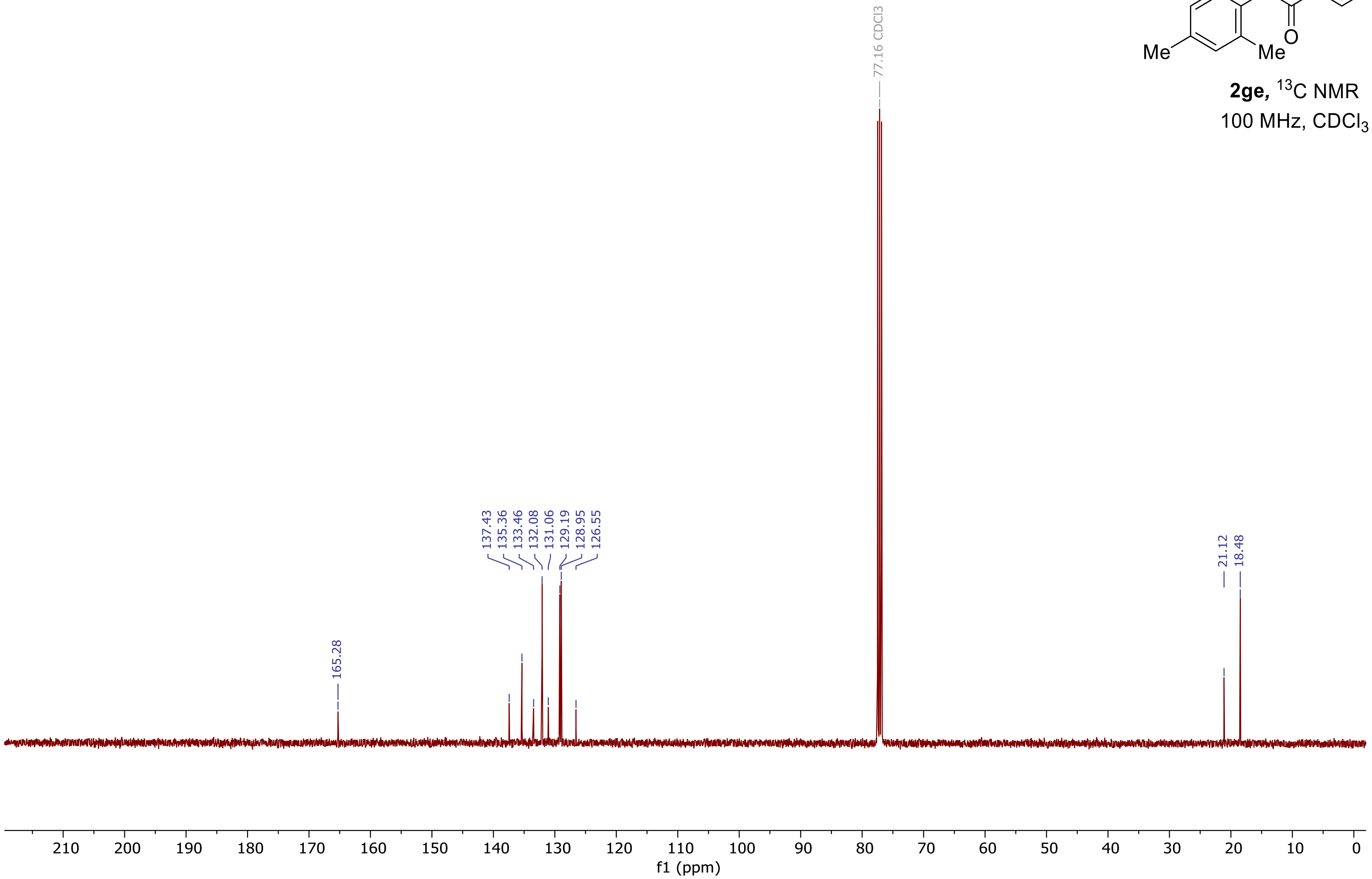

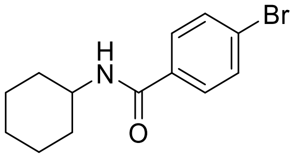

2he, ^{1}H NMR
400 MHz, $CDCl_3$

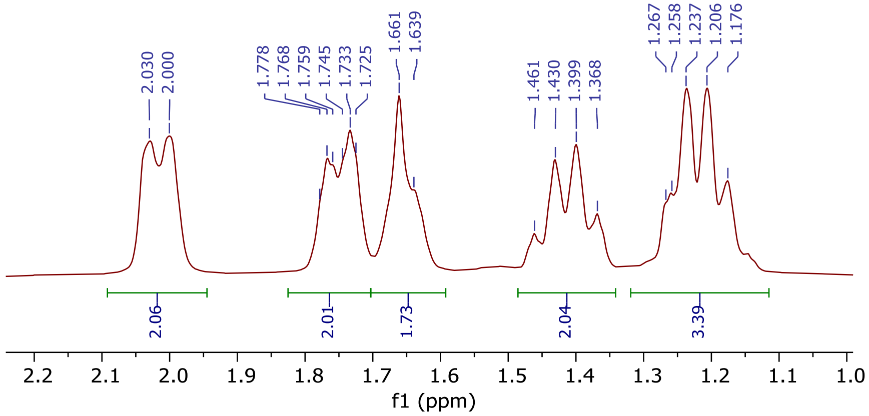

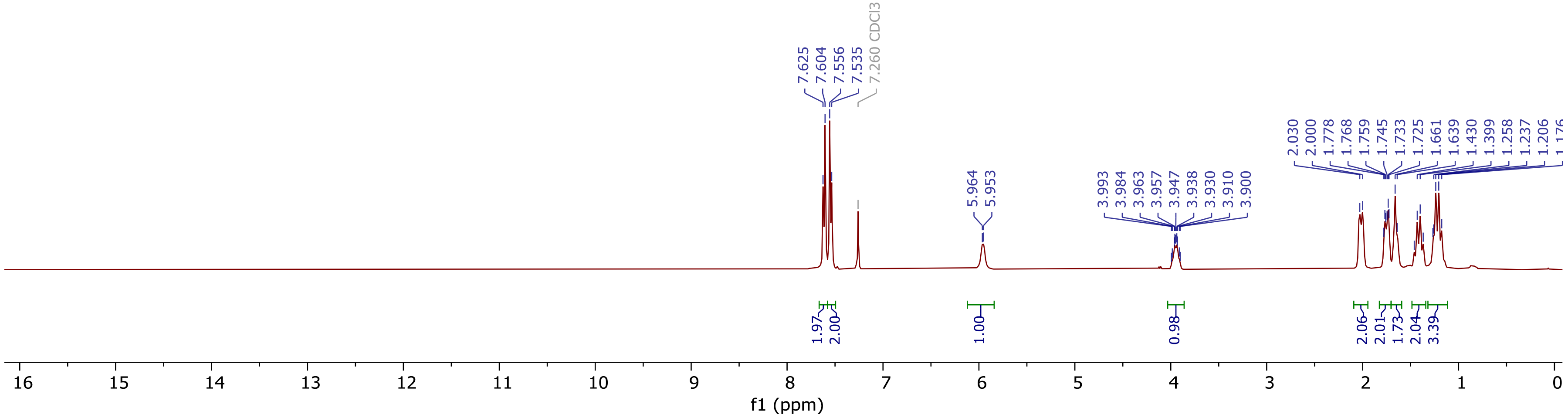

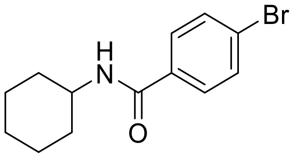

2he, ^{13}C NMR
100 MHz, $CDCl_3$

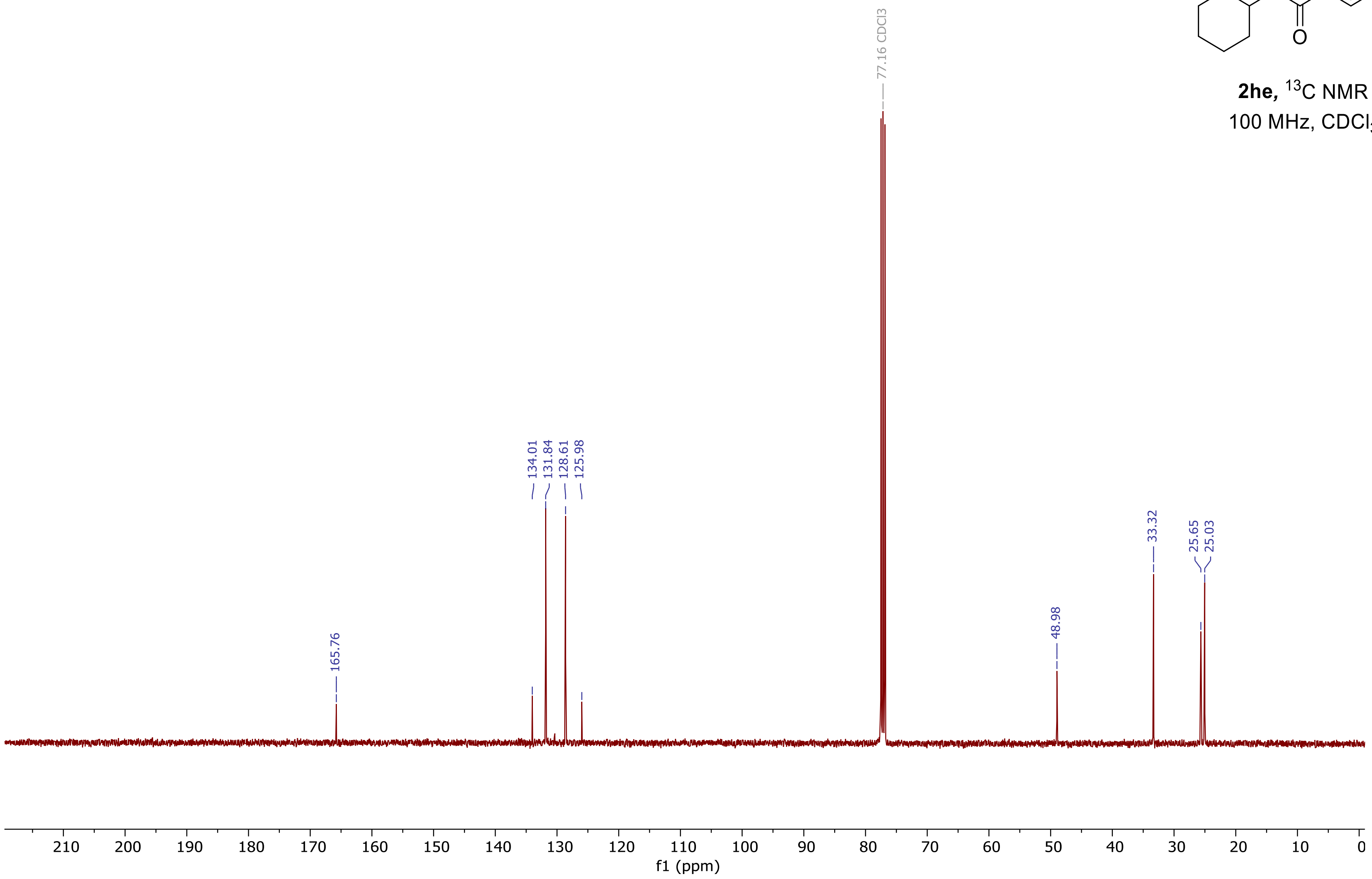

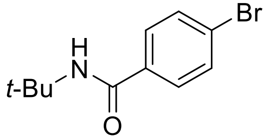

2ie, ^{1}H NMR
400 MHz, $CDCl_3$

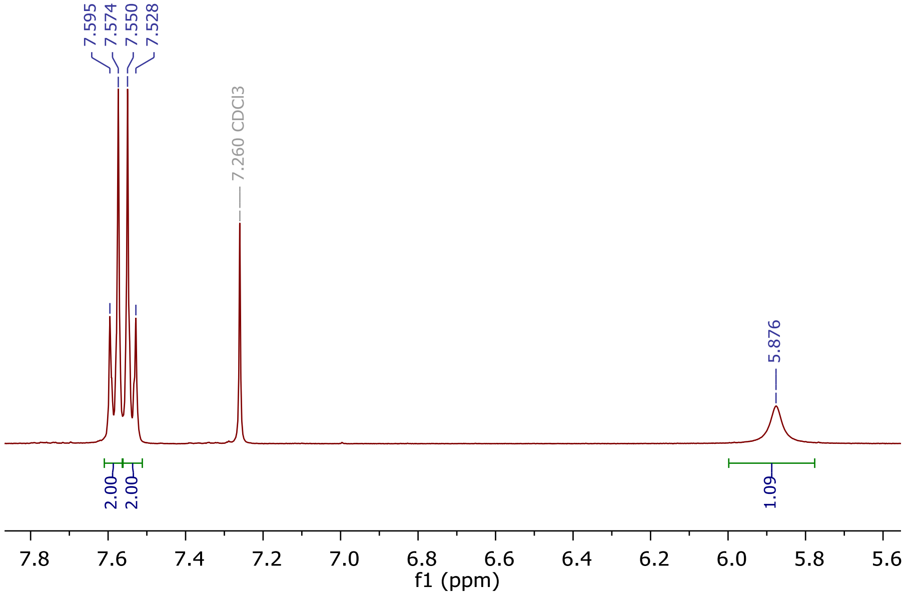

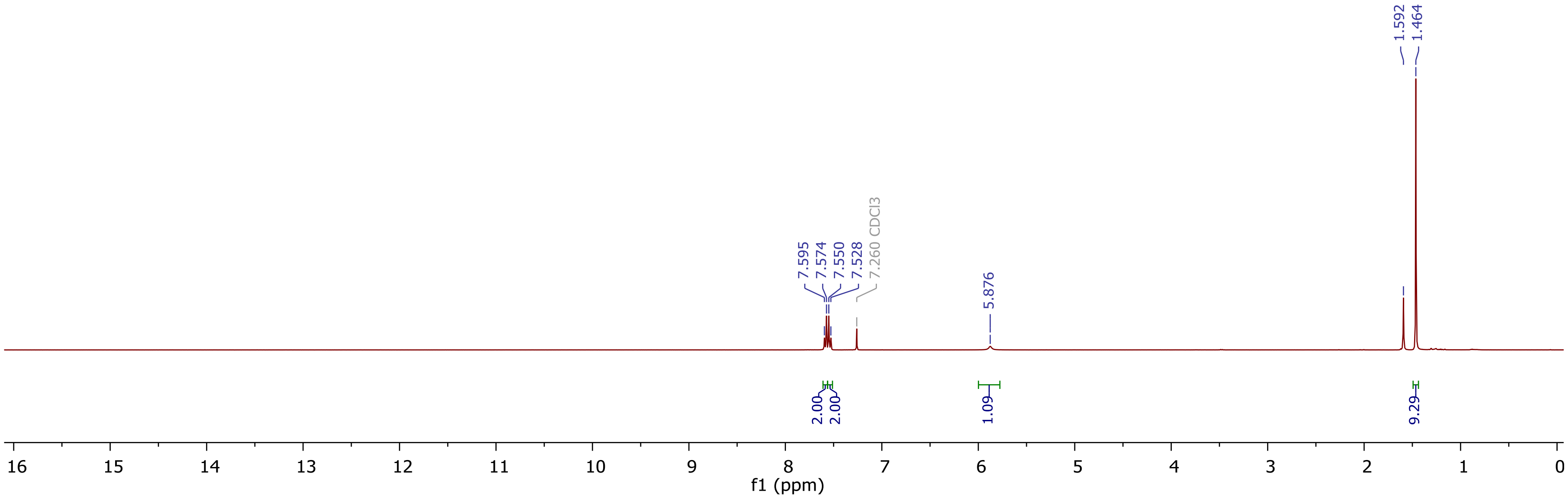

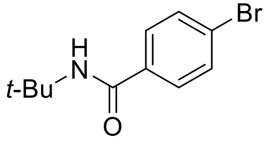

2ie, ^{13}C NMR
100 MHz, $CDCl_3$

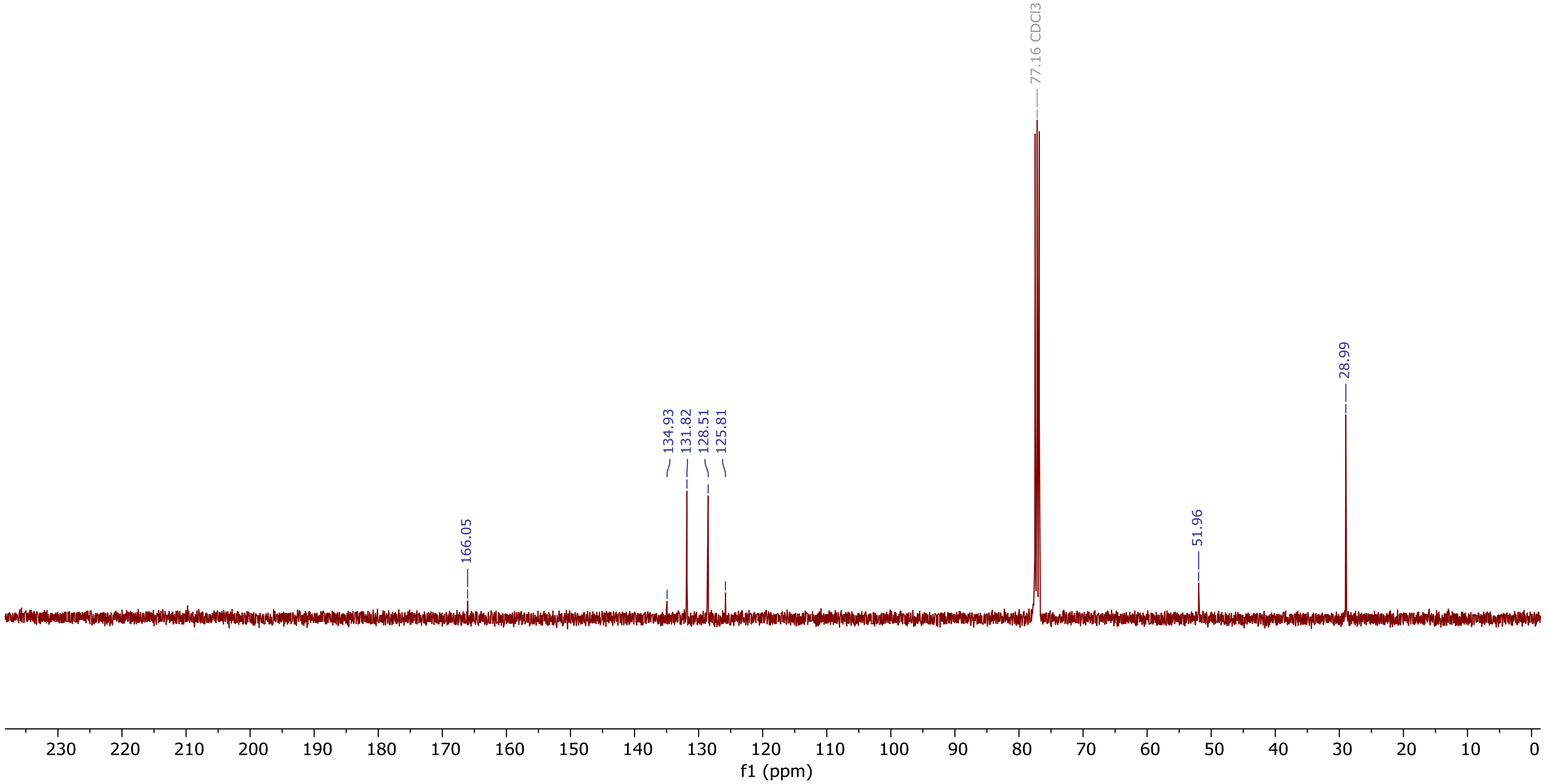

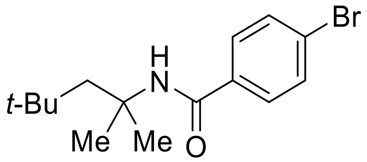

2je, ^{1}H NMR
400 MHz, $CDCl_3$

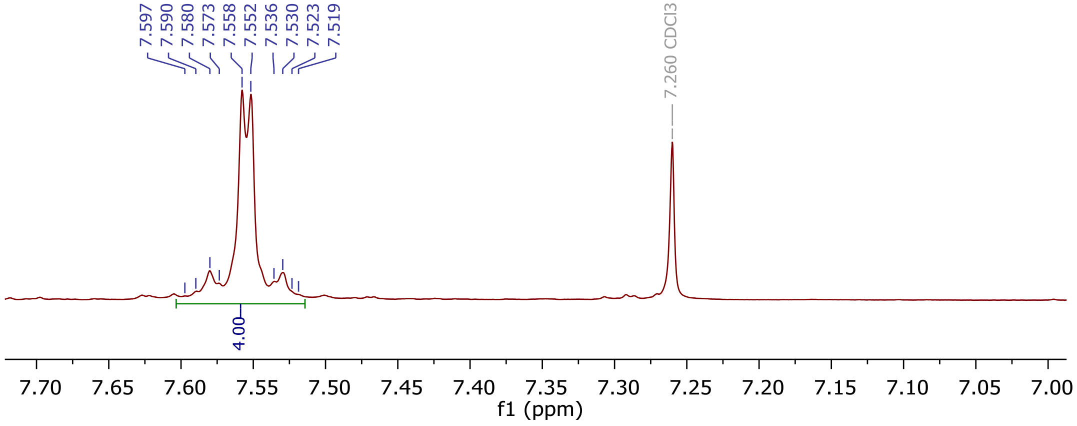

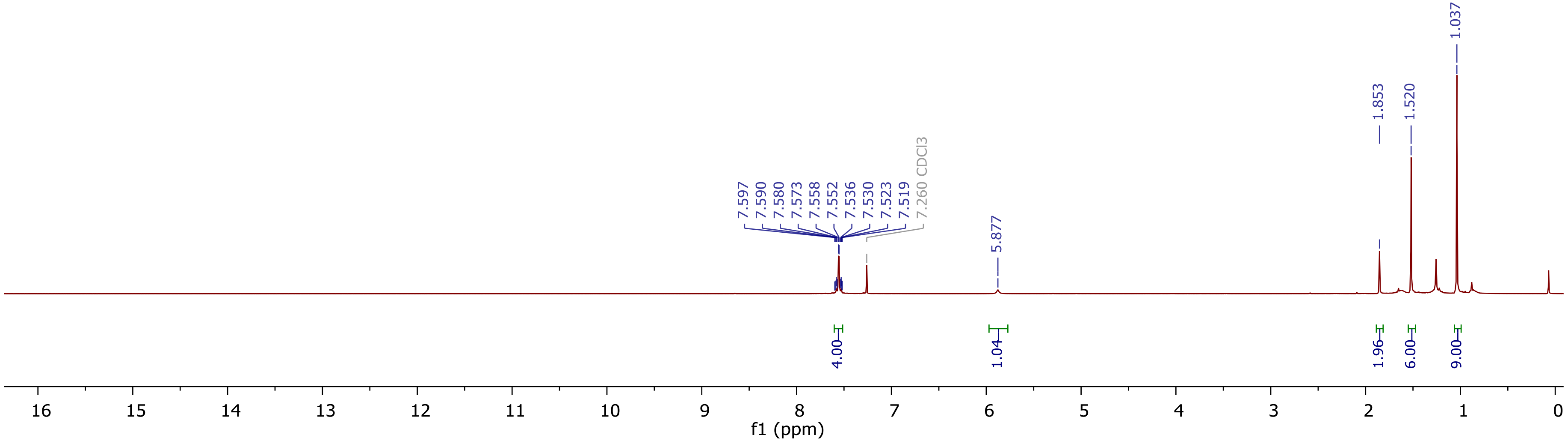

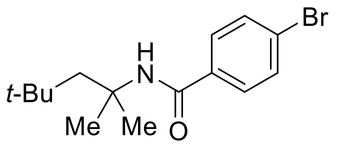

2je, ^{13}C NMR
125 MHz, $CDCl_3$

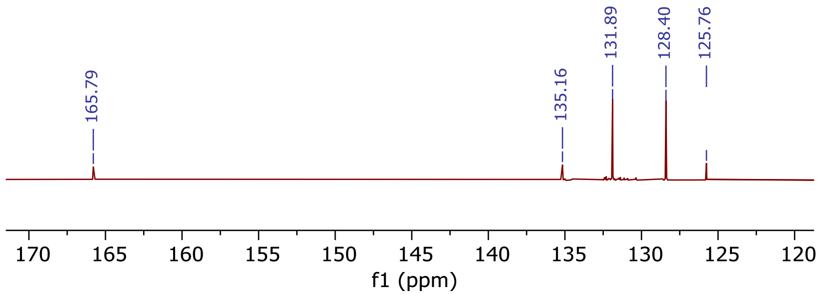

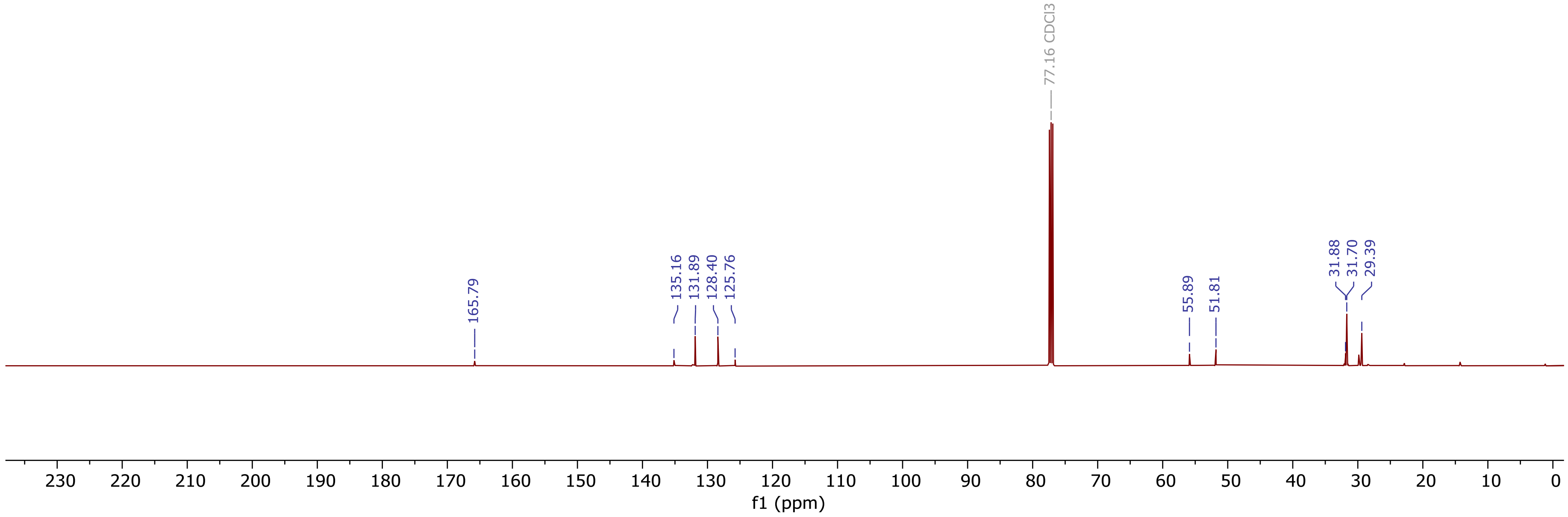

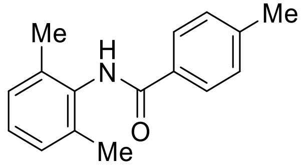

2bo, ^{1}H NMR
400 MHz, $CDCl_3$

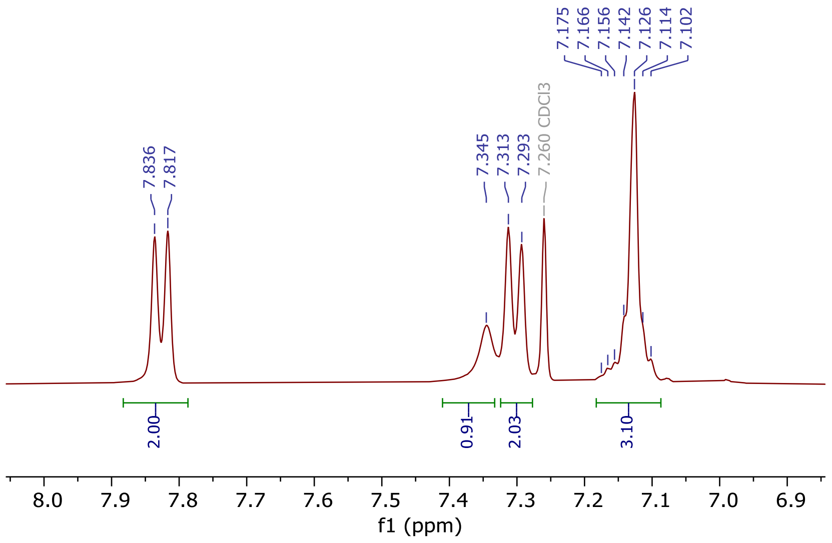

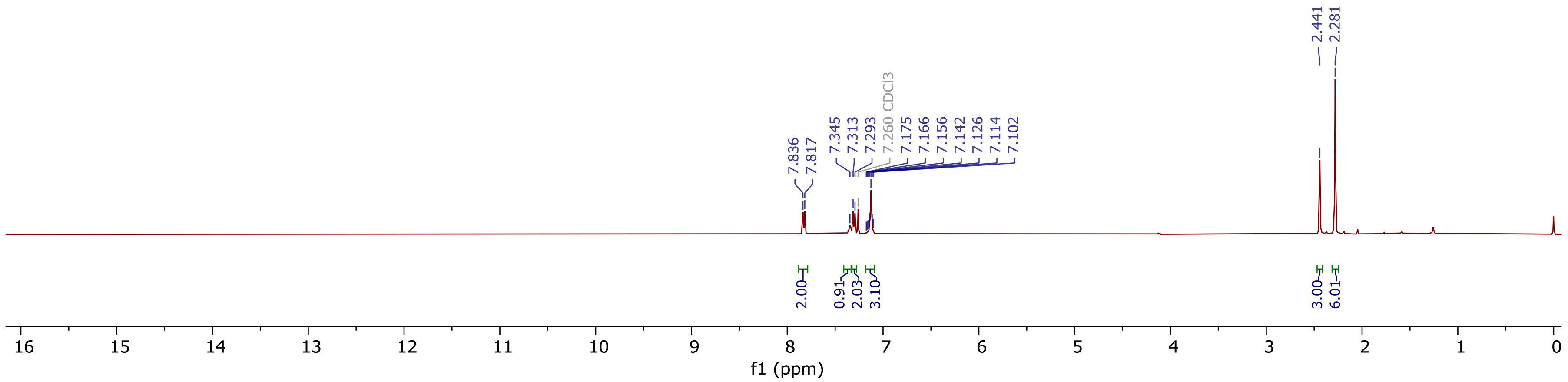

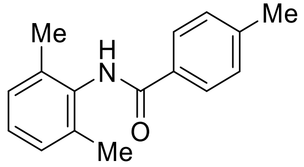

2bo, ^{13}C NMR

100 MHz, $CDCl_3$

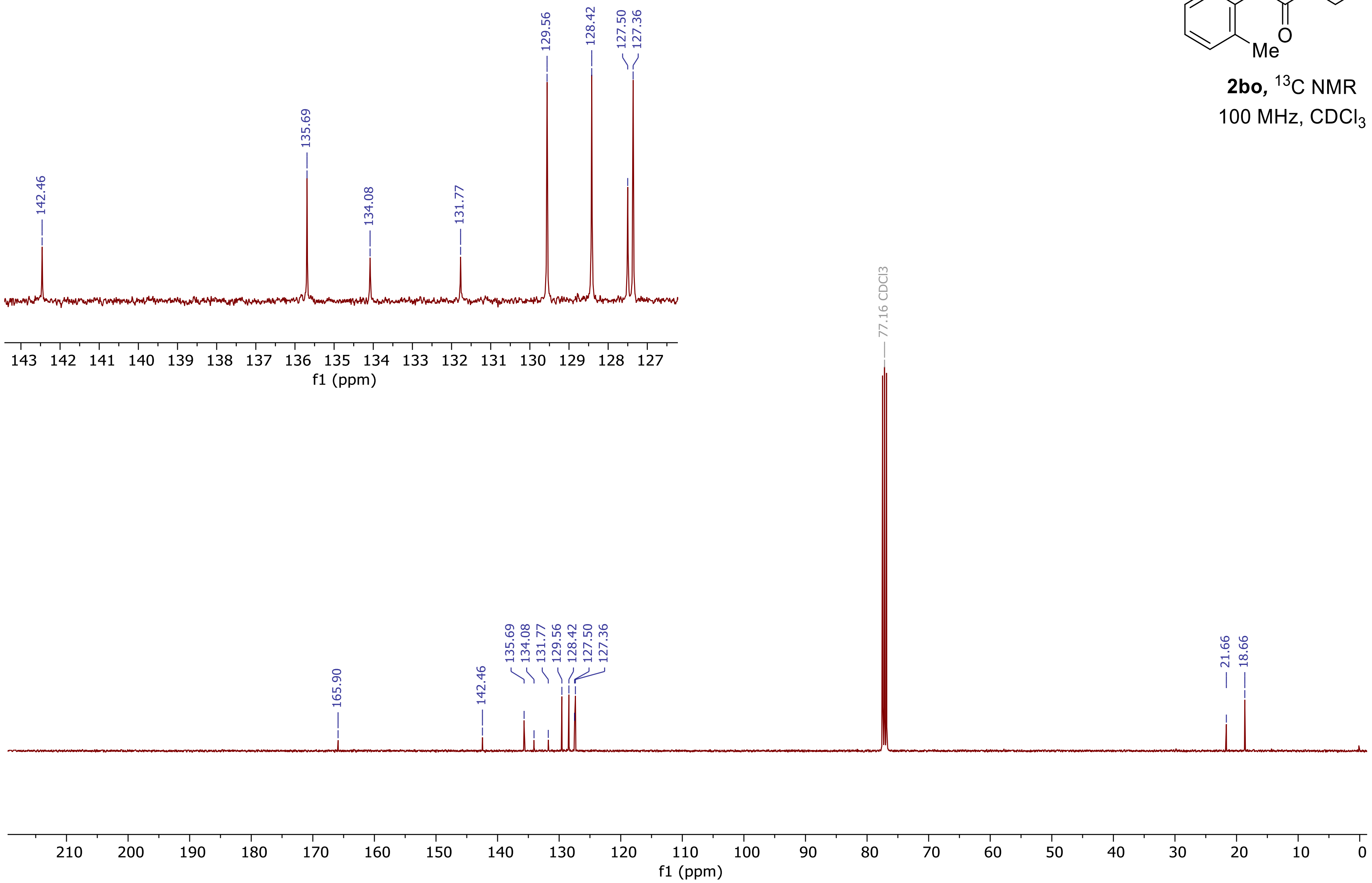

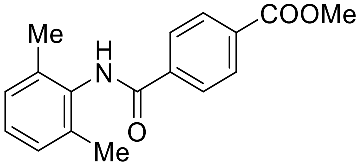

2bp, ^{1}H NMR
400 MHz, $CDCl_3$

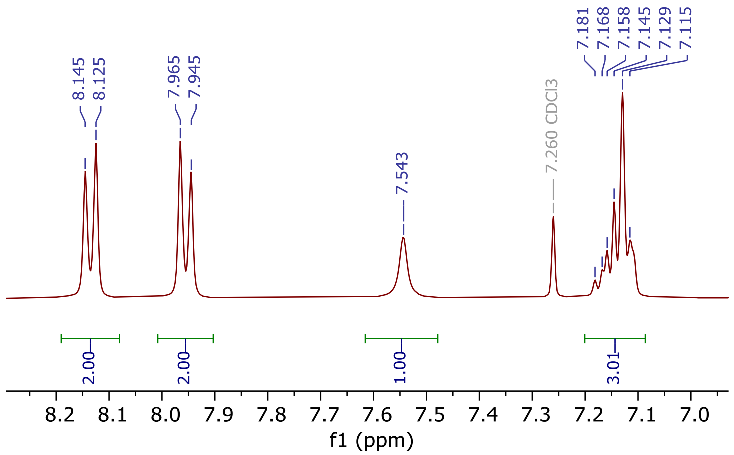

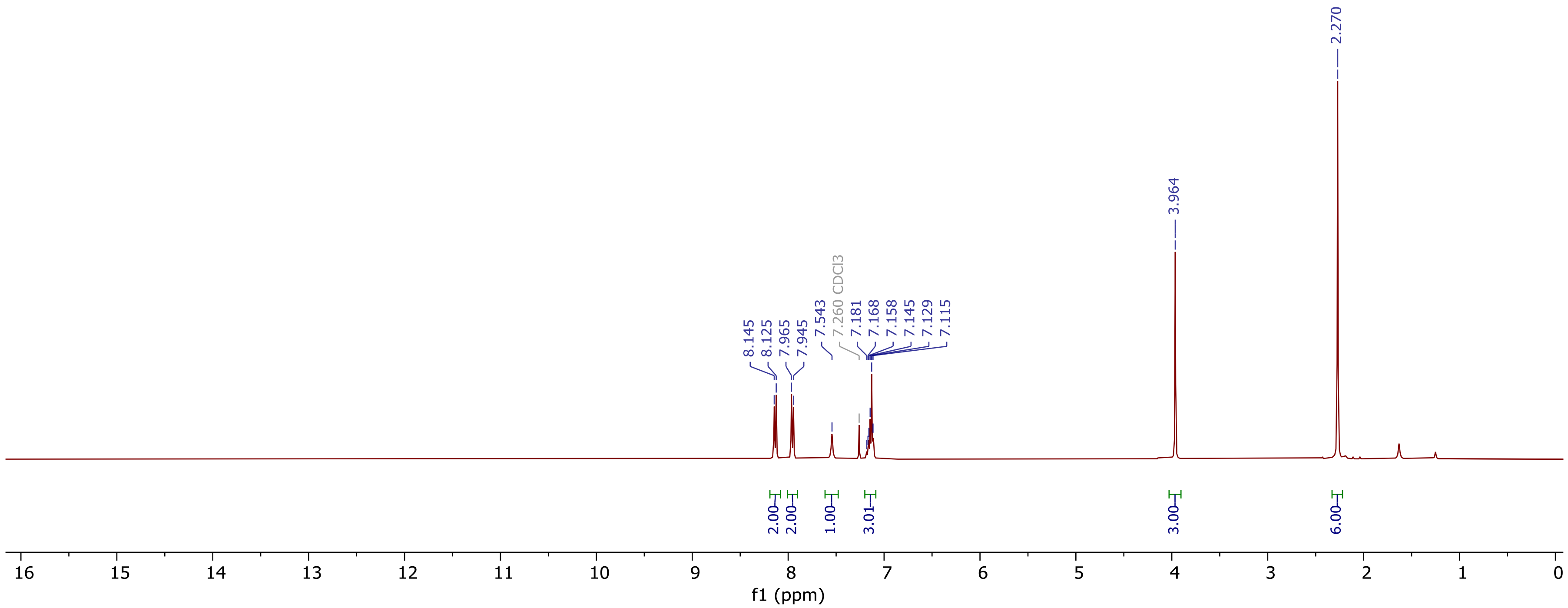

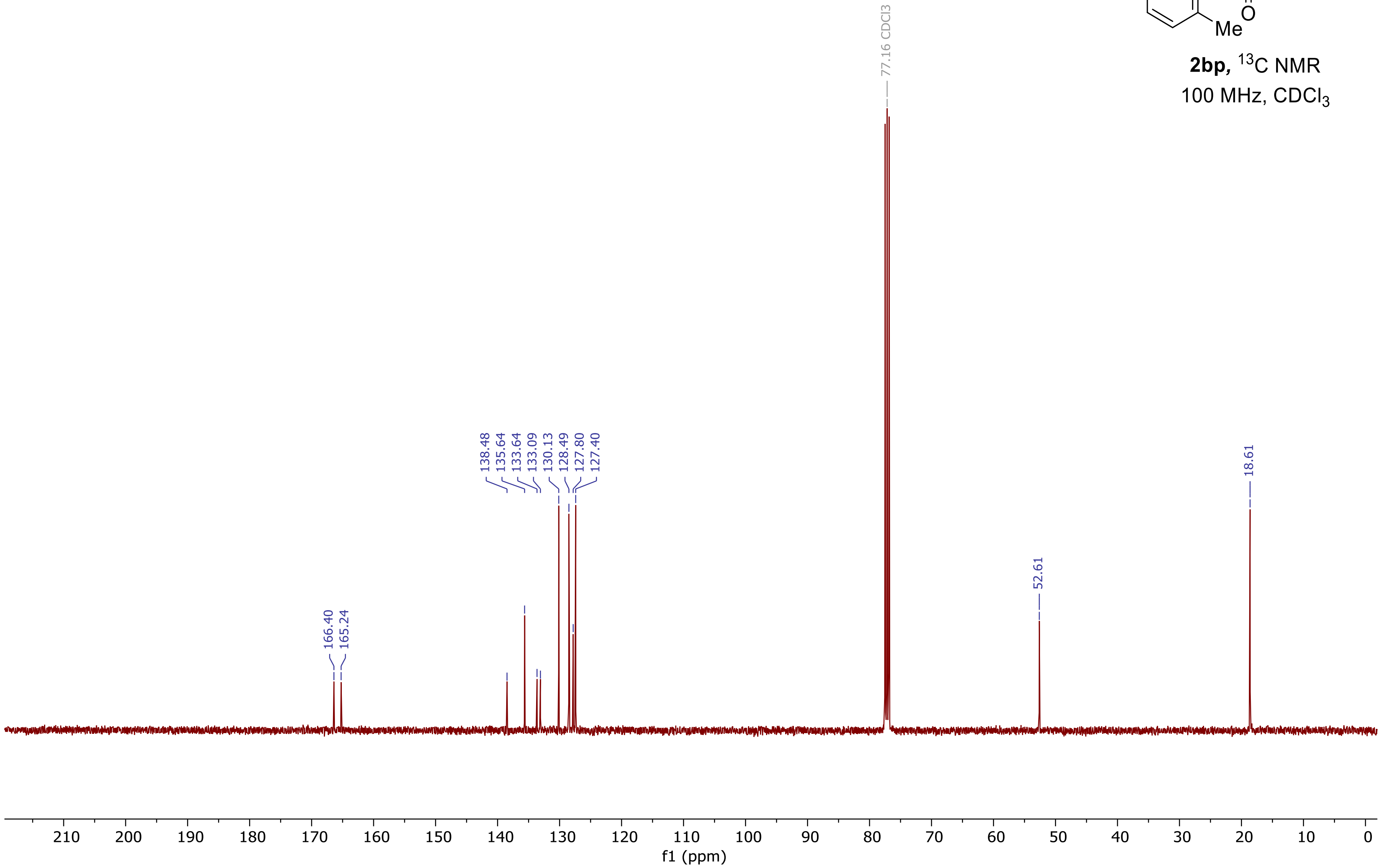

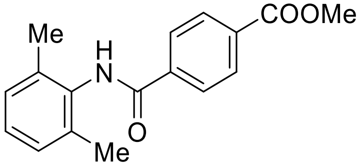

2bp, ^{13}C NMR
100 MHz, $CDCl_3$

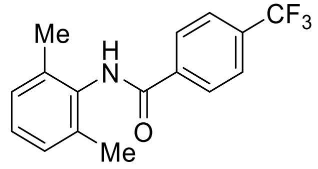

2bq, ^{1}H NMR
400 MHz, $CDCl_3$

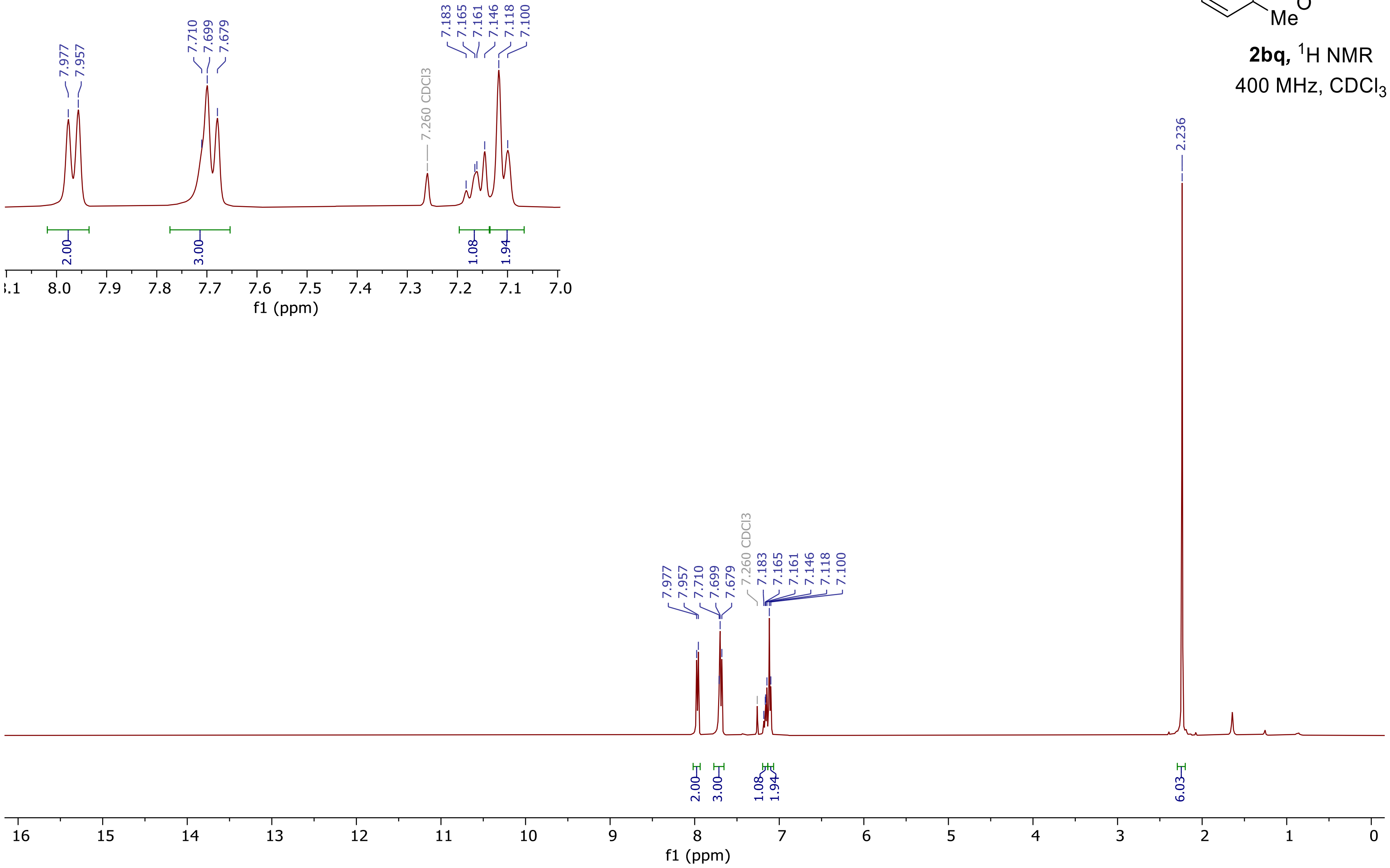

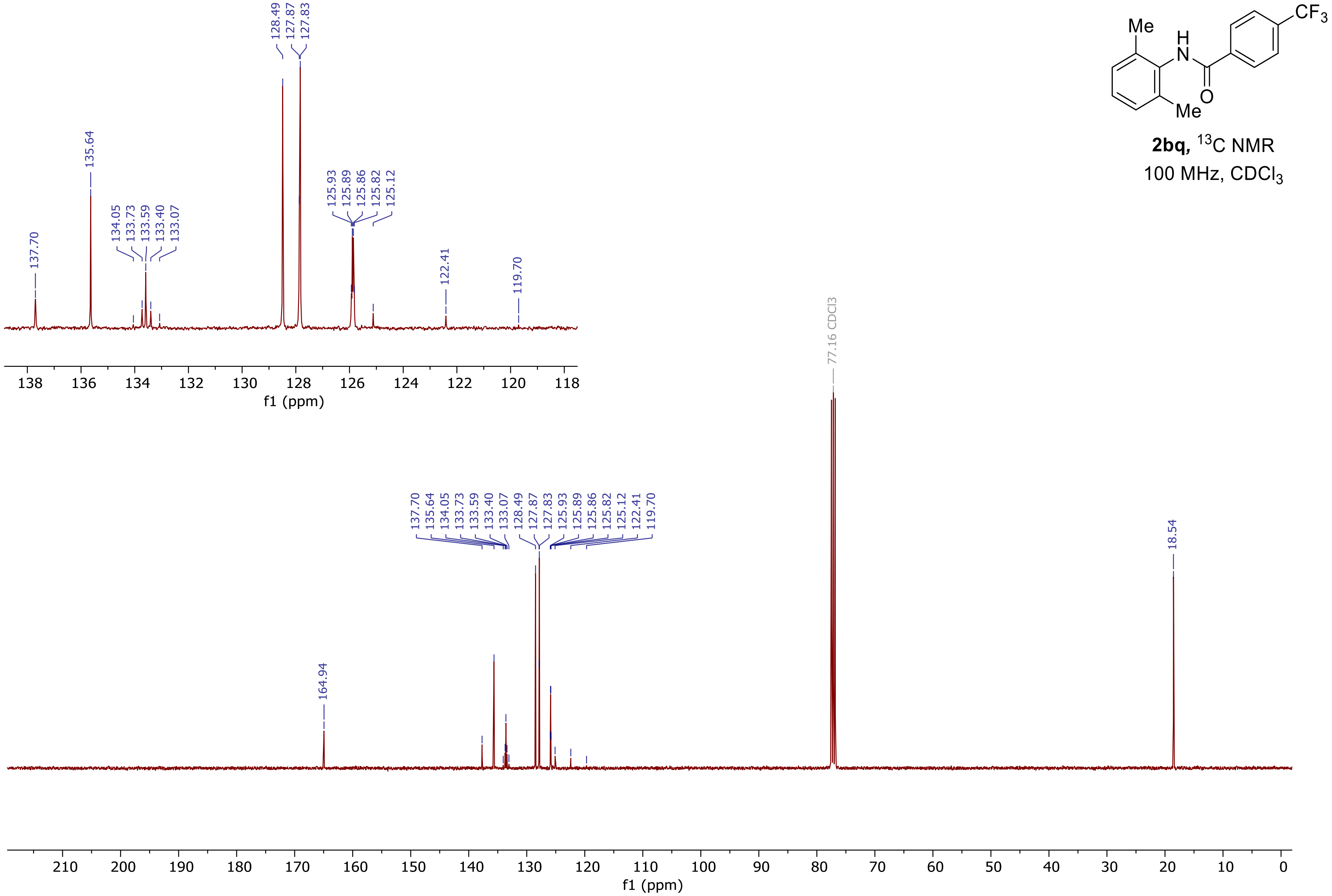

2bq, ^{13}C NMR
100 MHz, $CDCl_3$

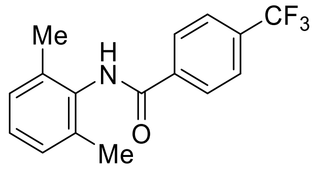

2bq, ^{19}F NMR
376 MHz, $CDCl_3$

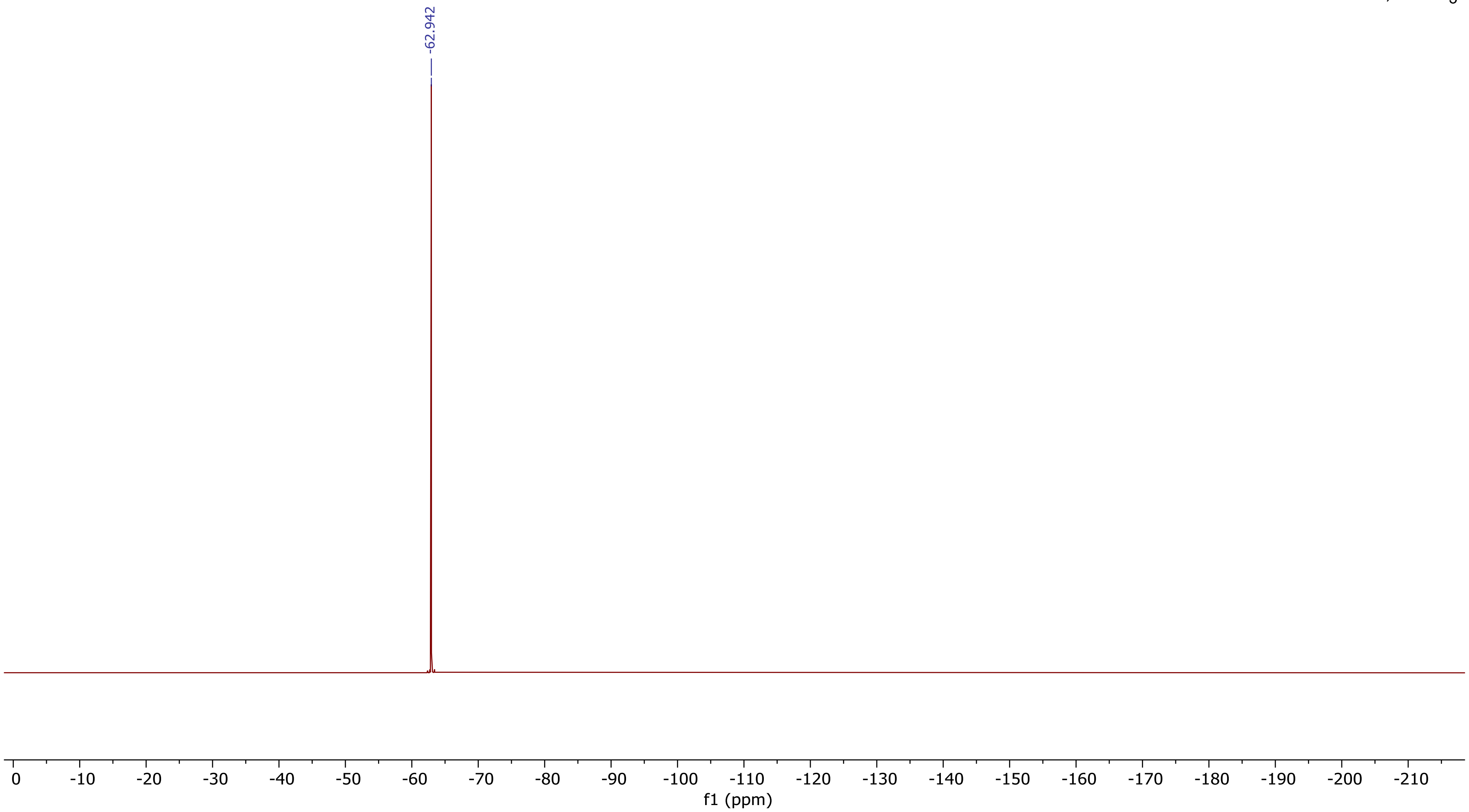

Supplement: File 1 — Experimental section, characterization data and control experiments. [file Beilstein_J_Org_Chem-21-1480-s001.pdf]
